# Single-cell transcriptomes identify human islet cell signatures and reveal cell-type–specific expression changes in type 2 diabetes

**DOI:** 10.1101/gr.212720.116

**Published:** 2017-02

**Authors:** Nathan Lawlor, Joshy George, Mohan Bolisetty, Romy Kursawe, Lili Sun, V. Sivakamasundari, Ina Kycia, Paul Robson, Michael L. Stitzel

**Affiliations:** 1 The Jackson Laboratory for Genomic Medicine, Farmington, Connecticut 06032, USA;; 2 Institute for Systems Genomics, University of Connecticut, Farmington, Connecticut 06032, USA;; 3 Department of Genetics & Genome Sciences, University of Connecticut, Farmington, Connecticut 06032, USA

## Abstract

Blood glucose levels are tightly controlled by the coordinated action of at least four cell types constituting pancreatic islets. Changes in the proportion and/or function of these cells are associated with genetic and molecular pathophysiology of monogenic, type 1, and type 2 (T2D) diabetes. Cellular heterogeneity impedes precise understanding of the molecular components of each islet cell type that govern islet (dys)function, particularly the less abundant delta and gamma/pancreatic polypeptide (PP) cells. Here, we report single-cell transcriptomes for 638 cells from nondiabetic (ND) and T2D human islet samples. Analyses of ND single-cell transcriptomes identified distinct alpha, beta, delta, and PP/gamma cell-type signatures. Genes linked to rare and common forms of islet dysfunction and diabetes were expressed in the delta and PP/gamma cell types. Moreover, this study revealed that delta cells specifically express receptors that receive and coordinate systemic cues from the leptin, ghrelin, and dopamine signaling pathways implicating them as integrators of central and peripheral metabolic signals into the pancreatic islet. Finally, single-cell transcriptome profiling revealed genes differentially regulated between T2D and ND alpha, beta, and delta cells that were undetectable in paired whole islet analyses. This study thus identifies fundamental cell-type–specific features of pancreatic islet (dys)function and provides a critical resource for comprehensive understanding of islet biology and diabetes pathogenesis.

Pancreatic islets of Langerhans are clusters of at least four different hormone-secreting endocrine cell types that elicit coordinated—but distinct—responses to maintain glucose homeostasis. As such, they are central to diabetes pathophysiology. On average, human islets consist mostly of beta (54%), alpha (35%), and delta (11%) cells; up to a few percent gamma/pancreatic polypeptide (PP) cells; and very few epsilon cells (Brissova et al. 2005; Cabrera et al. 2006; Blodgett et al. 2015). Human islet composition is neither uniform nor static but varies between individuals and across regions of the pancreas (Brissova et al. 2005; Cabrera et al. 2006; Blodgett et al. 2015). Cellular heterogeneity complicates molecular studies of whole human islets and may mask important role(s) for less common cells in the population (Dorrell et al. 2011b; Bramswig et al. 2013; Nica et al. 2013; Blodgett et al. 2015; Liu and Trapnell 2016). Moreover, it complicates attempts to identify epigenetic and transcriptional signatures distinguishing diabetic from nondiabetic (ND) islets, leading to inconsistent reports of genes and pathways affected (Gunton et al. 2005; Marselli et al. 2010; Taneera et al. 2012; Dayeh et al. 2014). Conventional sorting and enrichment techniques are unable to specifically purify each human islet cell type (Dorrell et al. 2008; Nica et al. 2013; Bramswig et al. 2013; Hrvatin et al. 2014; Blodgett et al. 2015), thus a precise understanding of the transcriptional repertoire governing each cell type's identity and function is lacking. Identifying the cell-type**–**specific expression programs that contribute to islet dysfunction and type 2 diabetes (T2D) should reveal novel targets and approaches to prevent, monitor, and treat T2D.

In this study, we sought to decipher the transcriptional repertoire of each islet cell type in an agnostic and precise manner by capturing and profiling pancreatic single cells from ND and T2D individuals. From these profiles, we identified transcripts uniquely important for each islet cell type's identity and function. Finally, we compared T2D and ND individuals to identify islet cell-type**–**specific expression changes that were otherwise masked by islet cellular heterogeneity. The insights and data from this study provide an important foundation to guide future genomics-based interrogation of islet dysfunction and diabetes.

## Results

### Islet single-cell transcriptomes accurately recapitulate those of intact islets

**Figure 1.**
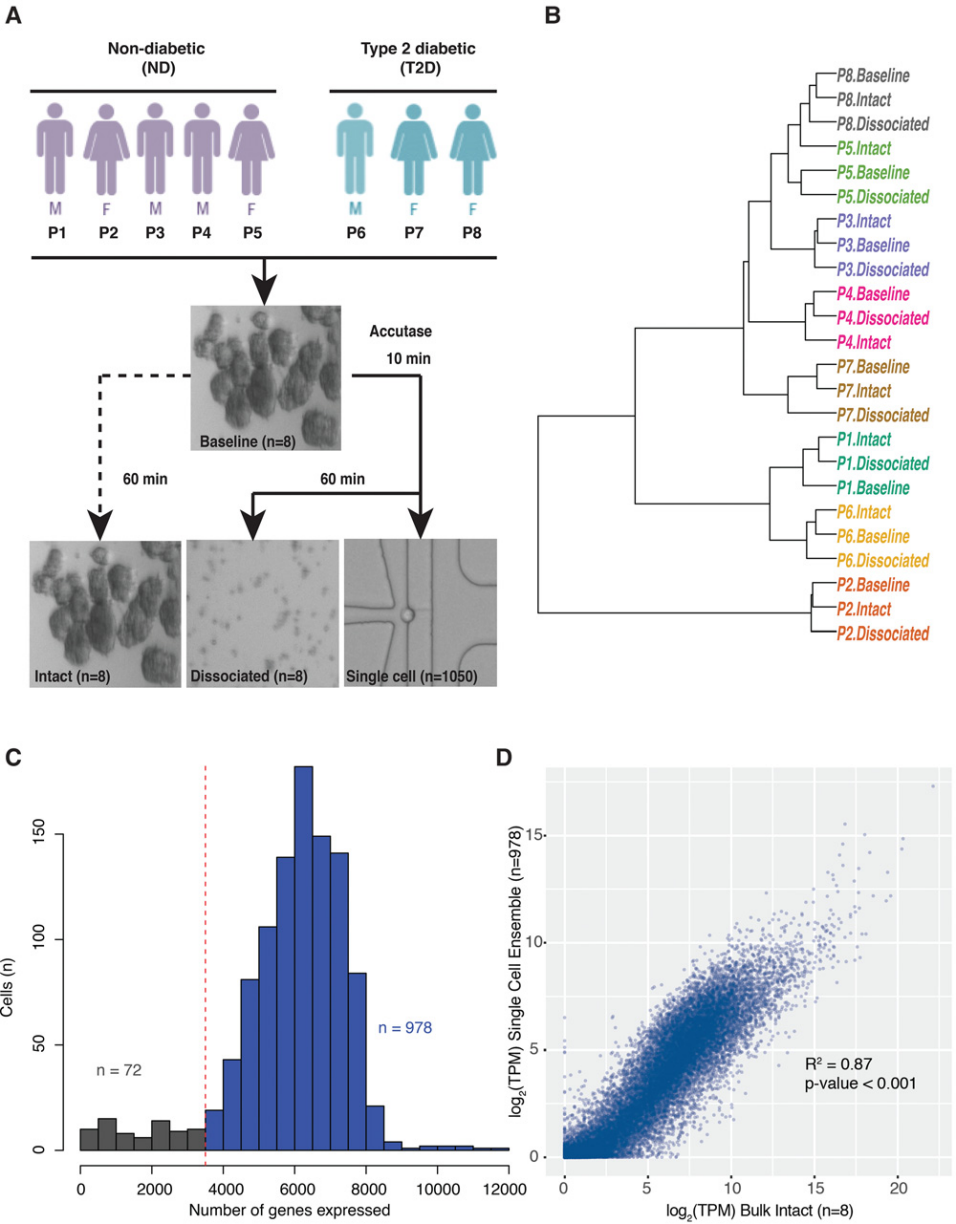
Single-cell transcriptomes reflect those of paired intact islets. (*A*) Schematic of experimental workflow. Islets from each donor sample (*n* = 8 individuals) were dissociated using Accutase, and single-cell transcriptomes were synthesized from 1050 cells captured using 11 Fluidigm C1 chips. In parallel, “bulk” RNA-seq libraries were prepared from remaining dissociated single cells (dissociated) and from intact islets either flash frozen (baseline) or incubated/processed (intact). (*B*) Unsupervised hierarchical clustering of baseline, intact, and dissociated islet transcriptomes demonstrates clustering by person and not by processing/experimental condition. (*C*) Histogram demonstrating the number of genes detected in each single cell. Cells expressing less than 3500 genes (*n* = 72) were removed from downstream analyses. (*D*) Scatter plot comparing intact islet bulk RNA-seq (*n* = 8) and ensemble single-cell RNA-seq (*n* = 978) data demonstrates high correlation. (*R*^2^) Pearson's *R*-squared; (TPM) transcripts per million; (P) person.

Pancreatic islets (>85% purity and >90% viability) were obtained from eight human cadaveric organ donors (five ND, three T2D) (Fig. 1A; Supplemental Table S1). Each islet sample was processed to generate single-cell RNA-seq libraries (Fig. 1A; single cell) and paired bulk RNA-seq libraries at three different stages of islet processing (Fig. 1A; baseline, intact, and dissociated). All RNA-seq methods employed SMARTer chemistry (Methods), and bulk islet cDNA libraries were sequenced to an average approximate depth of 34 million reads (Supplemental Table S2). Baseline, intact, and dissociated transcriptomes from each person were highly correlated (Supplemental Fig. S1). Transcriptomes clustered by donor and not by processing condition or incubation time (Fig. 1B), strongly suggesting that islet processing did not significantly alter islet transcriptomes.

A total of 1050 islet cells (622 ND and 428 T2D) were captured on 11 Fluidigm C1 chips. cDNA libraries were constructed from captured cells and barcoded, fragmented, pooled, and sequenced to an average depth of 3 million reads (Supplemental Table S2). Two separate library preparations from the same amplified cDNA for each of 83 single cells demonstrated remarkable correlation, suggesting minimal batch effects resulting from the cDNA processing and sequencing steps. Resequenced samples are highlighted in Supplemental Table S2 but were not included in subsequent analyses. Transcript coverage is indicated in Supplemental Figure S2. Approximately 81% (21,484/26,616) of protein-coding genes and long intergenic noncoding RNAs (lincRNAs) were detected in at least one cell from the collection. On average, each single cell expressed 5944 genes (Fig. 1C). Cells expressing less than 3500 genes (*n* = 72) also exhibited high mitochondrial alignment rates and other reported transcriptional metrics of cell death and/or poor quality (Ilicic et al. 2016; Xin et al. 2016) and were removed from subsequent analyses (Fig. 1C).

We next assessed the extent to which the remaining 978 single-cell transcriptomes represent the expression patterns observed in intact islets. Single-cell transcriptome ensembles from each person were highly correlated (Pearson's *R*^2^ ranged from 0.91–0.98) (Supplemental Fig. S3), regardless of disease state. Pearson's *R*^2^ values between individuals’ single-cell ensembles and corresponding “bulk” transcriptomes ranged from 0.75–0.86 (Supplemental Fig. S4) and did not differ substantially between ND (*R*^2^ = 0.87) and T2D (*R*^2^ = 0.85) samples (Supplemental Fig. S5). Overall, ensemble/aggregate single-cell transcriptome profiles correlated well with those of pooled bulk islet transcriptomes from all individuals (Fig. 1D, *R*^2^ = 0.87). These results suggest that the islet single-cell transcriptomes are high quality and effectively reflect bulk islet transcriptomes.

### Single-cell profiling captures transcriptomes of major and minor pancreatic endocrine and exocrine cell types

Five islet endocrine cell types have been assigned based on exclusive and robust expression of the peptide hormone genes *INS* (beta), *GCG* (alpha), *SST* (delta), *PPY* (PP/gamma), and *GHRL* (epsilon) (Baetens et al. 1979; Nussey and Whitehead 2001; Zhao et al. 2008; Li et al. 2016; Xin et al. 2016; Wang et al. 2016). The pancreas also contains three exocrine cell types—acinar, stellate, and ductal—that critically support digestion through synthesis and transport of digestive enzymes (Pandol 2011; Reichert and Rustgi 2011). Each also has been identified by specific marker gene expression, including the serine peptidase gene *PRSS1* (acinar) (Dabbs 2013), the extracellular matrix protein gene *COL1A1* (stellate) (Mathison et al. 2010), and the structural keratin gene *KRT19* (ductal) (Dorrell et al. 2008, 2011a,b; Reichert and Rustgi 2011). We used these marker genes to determine the representation of each islet cell type among our 978 profiled single cells.

**Figure 2.**
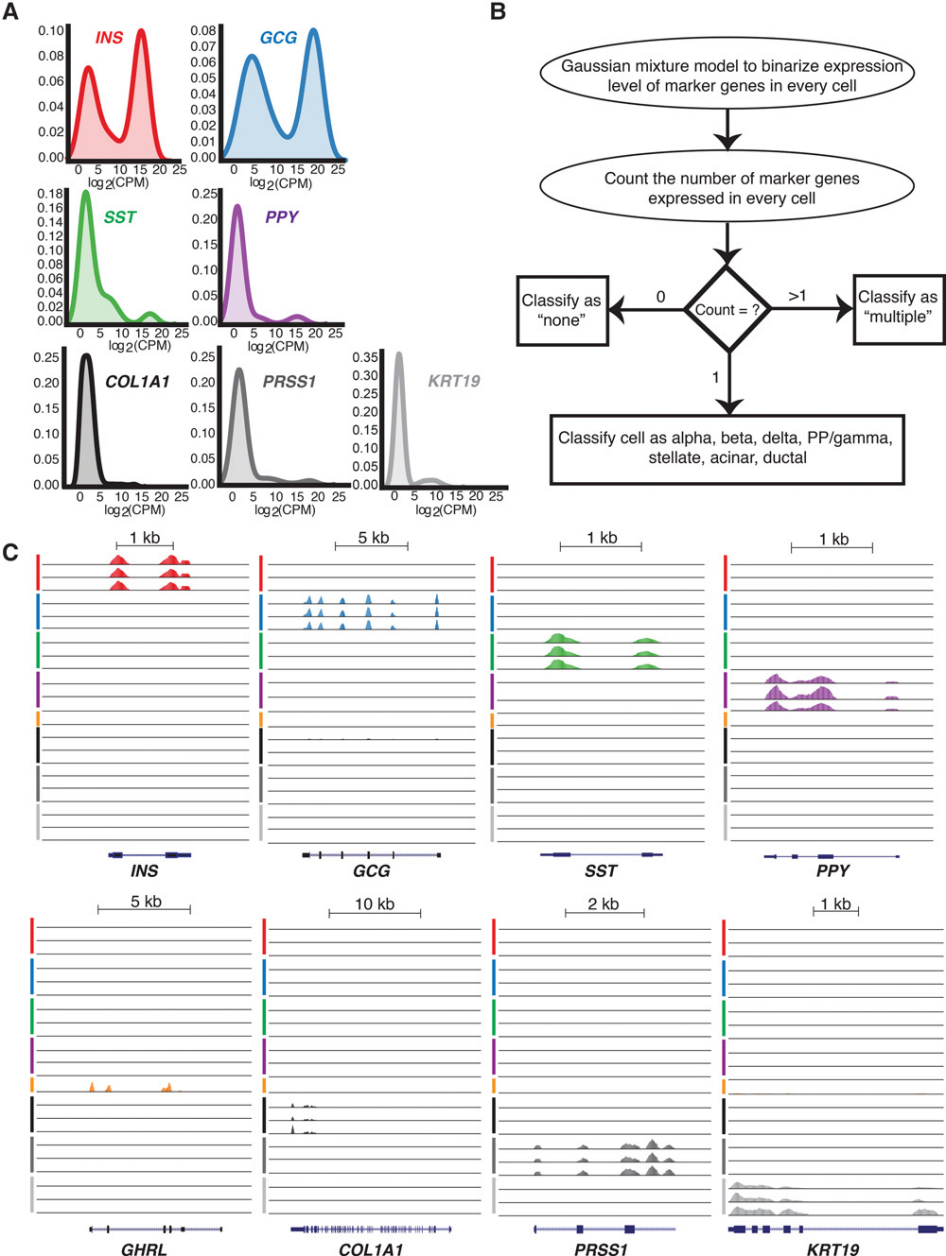
Cell-type classification based on marker gene expression. (*A*) Density plots demonstrating endocrine and exocrine marker gene expression across all single cells. (*B*) Schematic of the Gaussian mixture model method applied to assign cell-type identity based on marker gene expression. (*C*) UCSC Genome Browser views of representative single-cell expression profiles of *INS*, *GCG*, *SST*, *PPY*, and *GHRL* genes encoding beta, alpha, delta, PP/gamma, and epsilon cell hormones of the endocrine pancreas, respectively, and marker genes for stellate (*COL1A1*), acinar (*PRSS1*), and ductal (*KRT19*) cells of the exocrine pancreas. Line colors indicate putative beta (red), alpha (blue), delta (green), PP/gamma (purple), epsilon (orange), stellate (black), acinar (dark gray), and ductal cells (light gray). (PP) pancreatic polypeptide; (CPM) counts per million.

Density plots (Fig. 2A) revealed bimodal expression of each marker gene across the population of single cells. Therefore, we employed Gaussian mixture modeling (GMM) to classify the cells unambiguously (Fig. 2B). Approximate log_2_ counts per million (CPM) thresholds for each marker gene used to classify cell types are provided in Supplemental Table S3. This approach identified 617 single cells (∼63%) from T2D and ND islets expressing a single marker gene representative of each major endocrine and exocrine cell type, examples of which are shown in Figure 2C. This included 239 alpha, 264 beta, 25 delta, and 18 PP/gamma cells (Table 1); the proportions of each cell type are in the ranges previously reported (Brissova et al. 2005; Cabrera et al. 2006; Blodgett et al. 2015). Only one cell expressing high levels (log_2_CPM > 15) of *GHRL* was identified, which we presume to be an exceedingly rare epsilon cell. Additionally, we obtained 19 stellate, 24 acinar, and 27 ductal cells (Table 1), presumably from exocrine contamination of the islet cell preparations. Only 21 cells (∼2%) expressed none of the specified marker genes (Table 1). Approximately one-third (340/978) of cells expressed more than one marker gene; these were removed from subsequent analysis due to concerns that these represent two vertically stacked cells in a given capture site (for details, see Methods). Similar ratios of potential stacked cells have been reported in other studies using the Fluidigm C1 platform to capture mouse (Xin et al. 2016) and human islet cells (Wang et al. 2016). Collectively, these single-cell data capture transcriptome profiles representing each of the major and minor pancreatic endocrine and exocrine cell types. Genome Browser tracks representing aggregate single-cell expression for each islet cell type have been generated using HOMER (Heinz et al. 2010) and are made available (see Data Access) to facilitate their use and investigation by members of the islet biology and diabetes research communities.

**Table 1.**
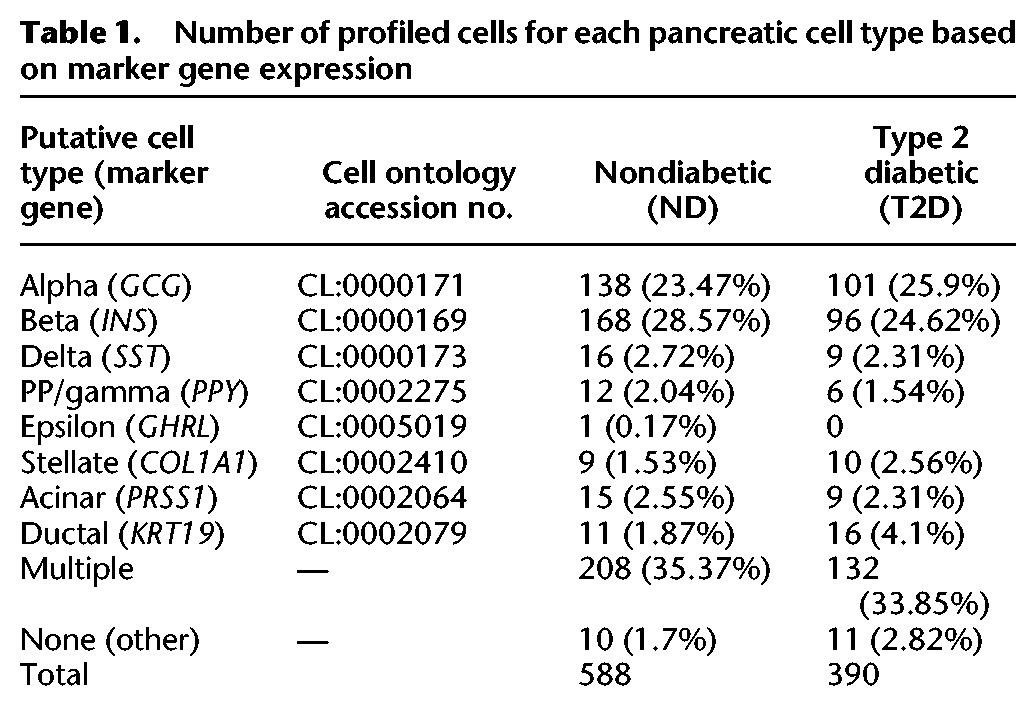
Number of profiled cells for each pancreatic cell type based on marker gene expression

### Unsupervised analyses of islet single-cell transcriptomes identify discrete clusters corresponding to cell type

**Figure 3.**
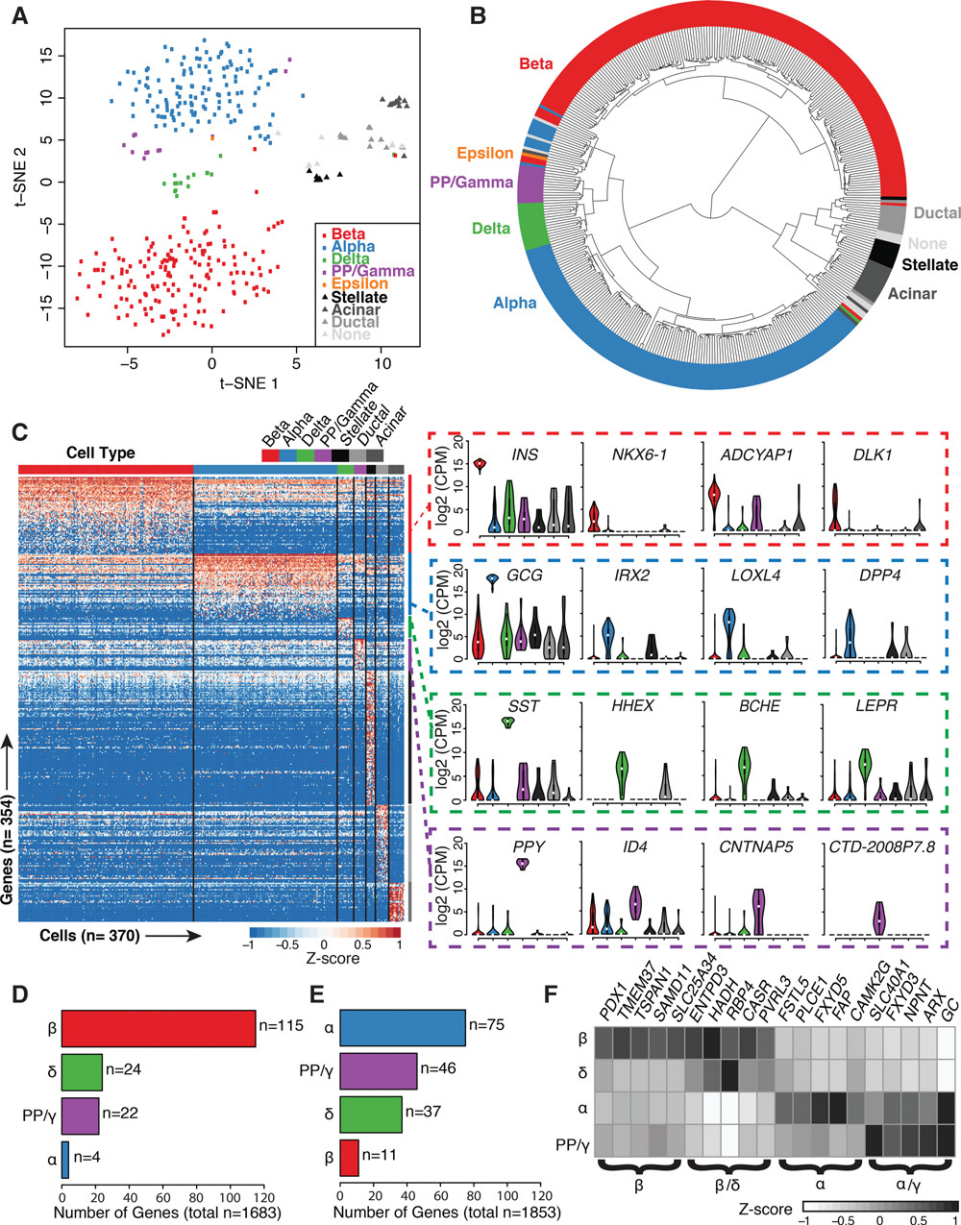
Statistical analysis of nondiabetic single-cell transcriptomes identifies cell-type–specific clusters and defines the signature genes of each islet cell type. (*A*) Unsupervised analysis of single-cell transcriptomes using t-distributed stochastic neighbor embedding (t-SNE) demonstrates grouping of single islet cell transcriptomes into the major constituent cell types. Respective cell labels and coloring were added after unsupervised analyses. (*B*) Unsupervised hierarchical clustering illustrates relationships of transcriptome profiles between respective endocrine and exocrine cells. (*C*) Supervised differential expression analysis of cell types determines cell-specific (signature) genes across all cells (see Methods). Values represent log_2_(CPM) expression after mean-centering and scaling between −1 and 1. Violin plots of selected signature gene expression are displayed to the *right* of the heatmap. (*D*,*E*) Bar plots depicting the numbers of previously reported beta-specific (*D*) and alpha-specific (*E*) genes (Dorrell et al. 2011b; Bramswig et al. 2013; Nica et al. 2013; Blodgett et al. 2015) confirmed to be expressed in each islet cell type after ANOVA and Tukey's honest significant difference (THSD) post-hoc analysis (Methods). (*F*) Several beta-specific genes demonstrate similar expression levels in delta cells, and alpha-specific genes demonstrate similar expression in PP/gamma cells. Values represent average log_2_(CPM) expression after mean-centering and scaling between −1 and 1. (β) Beta; (α) alpha; (δ) delta; (γ) PP/gamma cells.

To determine if and how islet cell transcriptomes cluster, we completed unsupervised dimensionality reduction via t-distributed stochastic neighbor embedding (t-SNE) on 380 ND single-cell samples (excluding “multiple” labeled samples). t-SNE assembled single-cell transcriptomes into discrete clusters based upon 1824 highly expressed genes (see Methods; Supplemental Table S4); GMM-based marker gene analysis revealed that each cluster corresponded to a distinct endocrine and exocrine cell type (Fig. 3A; Supplemental Fig. S6). Unsupervised hierarchical clustering also grouped single-cell transcriptomes into discrete cell types (Fig. 3B). Despite being obtained from different individuals, 161/168 beta, 128/138 alpha, 15/16 delta, and 12/12 PP/gamma cell transcriptomes clustered into the same dendrogram branches, strongly suggesting that cell type encodes the greatest variation in the data. Exocrine cells and those expressing none of the specified marker genes (“none”) clustered separately from the endocrine cell types. Importantly, this clustering was driven by neither sequencing depth (Supplemental Fig. S7B) nor expression of classic marker genes (*INS*, *GCG*, *SST*, *PPY, GHRL, COL1A1*, *PRSS1*, and *KRT19*), as cells continued to cluster into discrete cell types even when all marker genes were removed from the expression data sets (Supplemental Figs. S7C, S8). Recent studies have reported heterogeneity among beta cells. Specifically, Dorrell et al. characterized four subpopulations of human beta cells based on differing *ST8SIA1* and *CD9* expression (Dorrell et al. 2016). Similarly, Bader et al. 2016 distinguished two populations of proliferating (*Fltp*^+^) and mature (*Fltp*^−^) mouse beta cells. We did not find evidence of beta cell subpopulations (Supplemental Fig. S9), nor did we identify numerous proliferating cells (Supplemental Table S5). T2D single-cell transcriptomes (*n* = 258) also demonstrated clear clustering by cell type in unsupervised analyses (Supplemental Figs. S10–S14) based on 1908 highly expressed genes (Supplemental Table S4). Thus, each endocrine and exocrine pancreatic cell type exhibits a complex characteristic expression signature that uniquely identifies it.

### Differential expression analyses reveal islet cell-type**–**specific transcriptional signatures

To identify gene signatures distinguishing each islet cell type, we completed a series of pairwise differential expression analyses (Supplemental Table S6) between each cell type (see Methods). After intersecting the results from each pairwise comparison, we identified a conservative collection of 154 islet endocrine cell-type “signature” genes (61 beta, 51 alpha, 17 delta, 25 gamma), as well as 202 exocrine genes (109 stellate, 31 acinar, 62 ductal) at 5% false-discovery rate (FDR) (Fig. 3C; Supplemental Table S7). Two genes exhibited overlap between the endocrine and exocrine signature lists: *FAP* (alpha and stellate cell overlap) and *TNS1* (beta and stellate cell overlap). Gene set enrichment analysis (GSEA) identified enrichment (FDR-adjusted *P*-value <0.05) of insulin signaling, oxidative phosphorylation, maturity-onset diabetes of the young (MODY), and glycolysis/gluconeogenesis KEGG pathways in beta cells relative to the other endocrine cells (Supplemental Table S8).

Signature genes included previously reported beta-specific genes like *NKX6-1*, *DLK1*, and *ADCYAP1* (Fig. 3C, right) and alpha cell–specific genes like *IRX2*, *LOXL4*, and *DPP4*, a cell surface receptor and diabetes drug target (Dorrell et al. 2011a; Bramswig et al. 2013; Nica et al. 2013; Blodgett et al. 2015). Among delta cell signature genes, we detected exclusive expression of *HHEX,* a transcription factor reported to govern delta cell identity and function and linked to T2D GWAS (Zhang et al. 2014). Delta cells also specifically expressed *BCHE*, which encodes butyrylcholinesterase. BCHE catalyzes the breakdown of acetylcholine and ghrelin (Chen et al. 2015), thus providing a mechanism for delta cells to exert local inhibition of islet-influencing endocrine signals. PP/gamma cell–specific transcriptomes included *CTD-2008P7.8*, a lincRNA of unknown function; *CNTNAP5*, a member of the neurexin family of cell adhesion molecules; and *ID4*, which encodes an inhibitor of DNA-binding protein. In addition to *DPP4*, we detected 30 islet signature genes whose proteins SWISSPROT predicts to localize to the cell surface (Supplemental Table S9). DPP4 antibodies have recently been used to isolate purer alpha cell populations from islets (Arda et al. 2016). Thus, antibodies against the other candidate cell-type–specific surface markers we have identified may be useful to purify other islet cell types.

### Single-cell profiling identifies unexpected overlap in expression between minor and major islet cell types

Cell sorting and enrichment methods such as fluorescence-activated cell sorting (FACS) have been used to identify characteristic alpha and beta cell genes (Dorrell et al. 2011a,b; Bramswig et al. 2013; Nica et al. 2013; Blodgett et al. 2015). However, expression of *SST* or *PPY* in the reported alpha and beta cell gene sets suggests the presence of the less abundant delta and PP/gamma islet cell types in the enriched cell preparations. To distinguish genes exhibiting alpha- and beta-specific gene expression from those expressed also in delta and PP/gamma cells, we investigated the expression of previously reported alpha- and beta-specific genes (Supplemental Table S10; Supplemental Fig. S15) in our ND endocrine single-cell transcriptomes. Only 115/1683 previously reported beta-specific genes were expressed greater than fourfold higher in beta cells relative to the other endocrine cells (FDR < 0.05; one-way ANOVA followed by Tukey's honest significant difference [THSD]) (Fig. 3D). Similarly, 75/1853 reported alpha-specific genes were alpha cell enriched (Fig. 3E). Several genes previously reported to be enriched in the major islet cell types, such as *MAFA*, *SLC2A2*, *SIX3*, and *DLK1* in beta cells and *IRX2*, *DPP4*, and *ADORA2A* in alpha cells, were confirmed to be signature genes. Surprisingly, we found that 37 and 33 reported beta- and alpha-specific genes were also expressed in delta and PP/gamma cells, respectively (Fig. 3F; Supplemental Table S10). Notable examples included beta and delta cell expression of the congenital hyperinsulinemia (CHI) gene *HADH* and alpha and PP/gamma cell expression of the *ARX* transcription factor (Liu et al. 2011). *HADH* is typically associated with beta cell expression and, when mutated, leads to insulin hypersecretion and CHI (Kapoor et al. 2010; Pepin et al. 2010); these data implicate the delta cell in the molecular genetics of CHI. Misexpression of *ARX* has been shown to convey both alpha and PP/gamma cell features to cells (Collombat et al. 2007), suggesting that its expression in each cell type is important for identity and function.

### Genes underpinning metabolic function, regulation of energy homeostasis, and satiety are specific to distinct islet cell types

**Figure 4.**
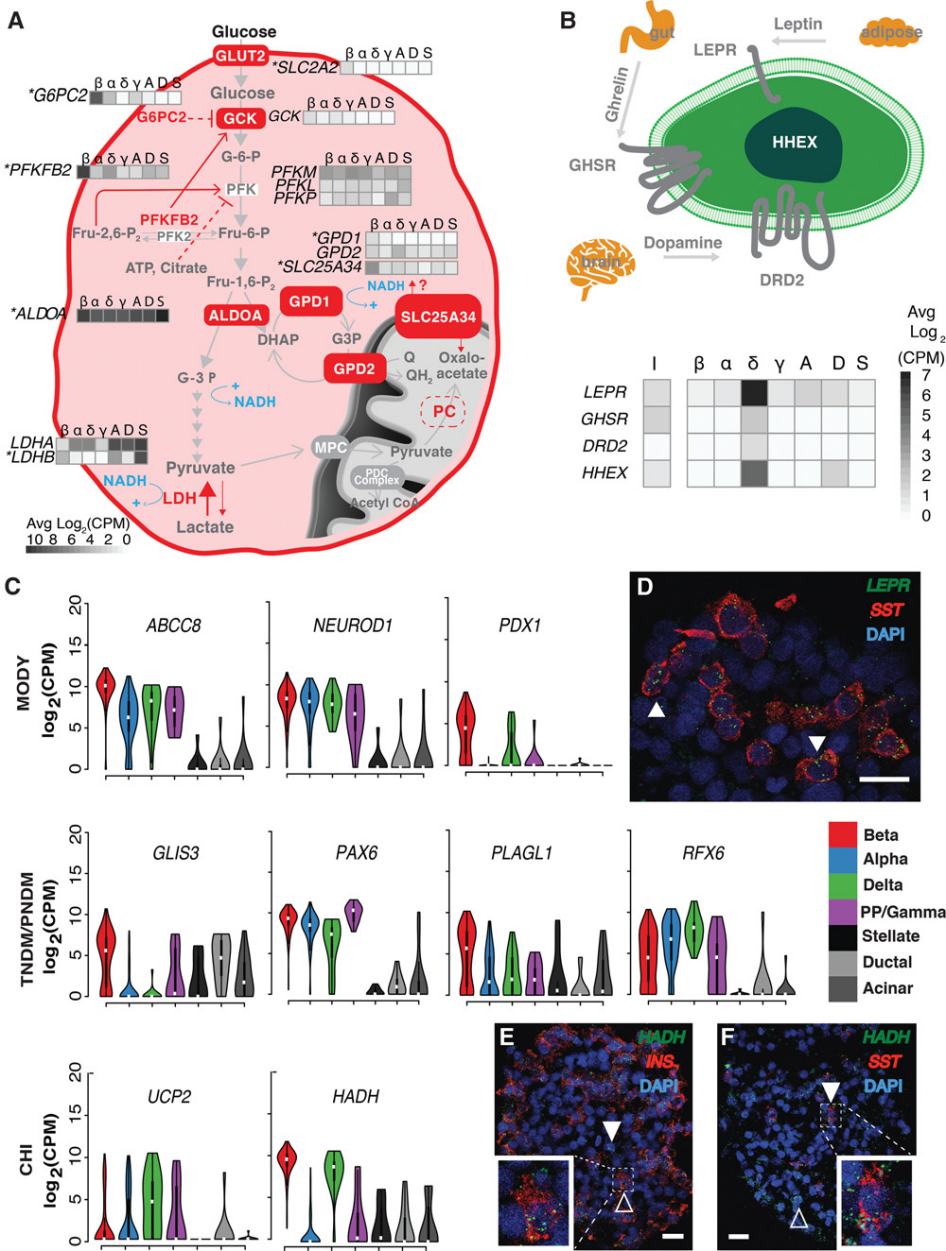
Cell-type**–**specific expression of metabolic, signaling, and diabetes trait genes. (*A*) Beta cell–specific expression of different isoforms of glycolytic and metabolic intermediate shuttles. Genes marked with an asterisk represent beta cell signature genes. (*B*) Delta cell–specific expression of neuroactive-ligand receptors and transcription factors. (I) Bulk intact islets; (β) beta; (α) alpha; (δ) delta; (γ) PP/gamma; (A) acinar; (D) ductal; (S) stellate cells. (*C*) Monogenic diabetes–associated genes and their cell-type**–**specific expression in islets. Violin plots show the log_2_(CPM) expression of each gene across cell types. (CHI) congenital hyperinsulinism; (MODY) maturity onset diabetes of the young; (TNDM) transient neonatal diabetes mellitus; (PNDM) permanent neonatal diabetes mellitus. (*D*) RNA in situ hybridization (ViewRNA, Affymetrix) of OCT-embedded islet sections from donor P3 labeling *SST* (red), *LEPR* (green), and nuclei (DAPI; blue). White arrowheads indicate *SST*^+^/*LEPR*^+^ cells. ViewRNA of OCT-embedded islet sections from donor P4 to detect the following: (*E*) *INS* (red), *HADH* (green), and nuclei (DAPI; blue) and (*F*) *SST* (red), *HADH* (green), and nuclei (DAPI; blue). White arrowheads highlight examples of *HADH*^+^/*INS*^−^ (*E*) and *HADH*^+^/*SST*^+^ (*F*) cells. Hollow arrowheads highlight *HADH*^+^/*INS*^+^ (*E*) and *HADH*^+^/*SST*^−^ (*F*) cells. In *D*–*F*, solid horizontal white lines indicate scale bars of 20 μm. In *E* and *F*, white dashed lines highlight a cell either co-expressing (*E*) *INS/HADH* or (*F*) *SST/HADH*. White squares in the *bottom left* of *E* and *bottom right* of *F* indicate magnified images of the cells highlighted in respective dashed white boxes.

Perturbations in genes involved in glucose sensing and proper maintenance of blood glucose levels contribute to T2D pathophysiology (Schuit et al. 2001; MacDonald et al. 2005). Beta cells regulate blood glucose through the secretion of insulin and are thus exquisitely sensitive to blood glucose levels. Glucose-stimulated insulin secretion (GSIS) is linked to universal basic pathways of cellular metabolism in beta cells. To gain insight into beta cell-type**–**specific transcriptomic features associated with GSIS, namely, glucose uptake and glycolysis, we examined the expression of relevant genes in our islet single-cell transcriptomes (Fig. 4A).

GSIS pathway genes associated with glucose sensing and uptake displayed highly beta cell–specific expression, including *SLC2A2*, which encodes the glucose transporter GLUT2; *G6PC2*, which encodes a subunit of glucose-6-phosphatase; and *PFKFB2*, which encodes an enzyme involved in regulation of glycolysis (Fig. 4A; Chen et al. 2008; Muller et al. 2015). While expressed in all cell types, the enzyme, ALDOA1, immediately downstream from PFK1 and associated with the glycerol phosphate (GP) shuttle, is enriched in beta cells, perhaps reflecting an additional point of GSIS control. Protein-coding genes for five subsequent glycolytic enzymatic steps from glyceraldehyde-3-phosphate to pyruvate were not significantly differentially expressed between cell types. Beta cells are known to be limited in their ability to produce lactate from pyruvate (Fridlyand and Philipson 2010); this is reflected by high *LDHB*/*LDHA* ratios that favor the lactate to pyruvate flux in beta cells.

The glycerol-3-phosphate shuttle allows NAD^+^ regeneration in the cytosol to sustain glycolytic flux essential for GSIS. Cytoplasmic NAD^+^ generation has been shown to be essential for GSIS (Eto et al. 1999). Both components of the glycerol-3-phosphate shuttle, cytoplasmic *GPD1* and mitochondrial *GPD2*, were expressed in beta cells, with the former representing a beta cell signature gene (Fig. 4A). Additionally, we identified the mitochondrial solute transporter *SLC25A34* as beta cell specific. While its transport specificities have yet to be determined, the closest yeast ortholog of *SLC25A34*, Oac1p/YKL120w (Palmieri et al. 1999; Marobbio et al. 2008), is thought to import oxaloacetate into the mitochondria. This is particularly intriguing considering our data and others (MacDonald et al. 2011) show the complete absence of pyruvate carboxylase (PC) expression in human beta cells, despite the essential role PC is known to play in rodent GSIS (Sugden and Holness 2011) through mitochondrial production of oxaloacetate. We hypothesize that *SLC25A34* may provide an alternate, cytoplasmic source for mitochondrial oxaloacetate in the human beta cell.

Single-cell profiling also allowed us to interrogate the transcriptional repertoire of less abundant delta and PP/gamma cell types, which have been elusive in both whole islet and sorted islet studies. While it is difficult to determine epsilon cell expression signatures with one ghrelin-positive cell, our ND data set includes 16 delta cells and 12 PP/gamma cells. Among the top 100 differentially expressed (FDR < 5%) genes in delta versus other islet endocrine cells are receptors for the appetite-regulating hormones leptin (*LEPR*) and ghrelin (*GHSR*), the growth factor neuregulin 4 (*ERBB4*), and the neurotransmitter dopamine (*DRD2*) (Fig. 4B). *GHSR* has recently been shown to be specifically expressed and functional in both human and mouse delta cells, reducing GSIS in human and mouse beta cells when induced (DiGruccio et al. 2016). *LEPR*, *DRD2*, and *ERBB4* expression is specific to human delta cells. In situ analyses (ViewRNA, Affymetrix) detected coexpression of *LEPR* in 79/102 (77%) of *SST*-expressing cells (Fig. 4D, arrowheads) in ND islets, confirming the delta cell–specific expression detected in Fluidigm C1 profiling. Thus, our data suggest intriguing roles for islet delta cells in the integration of metabolic signals via leptin, ghrelin, and dopamine signaling pathways.

PP/gamma, along with epsilon cells, are among the least studied islet cell types due to their scarcity in islets. Recent studies show that PP/gamma cells are crucial regulators of energy homeostasis (Yulyaningsih et al. 2014; Khandekar et al. 2015). In response to food intake, these cells secrete the anorexigenic hormone PPY to facilitate vagal stimulation of neuropeptide Y receptors in the hypothalamus and induce satiety (Khandekar et al. 2015). Our data suggest interesting parallels in expression between PP/gamma cells and serotonergic neurons, a group of neurons that influence various cognitive and physiological processes including anxiety, mood, sleep, and satiety. We report expression of *FEV*, a serotonergic transcription factor and necessary driver of neuronal maturation previously reported in mouse beta cells (Ohta et al. 2011), in PP/gamma cells (average log_2_CPM of 2.172). Interestingly, FEV has also been implicated in beta cell differentiation, and *Fev*^−/−^ mice exhibit insulin production, insulin secretion, and glucose clearance defects (Ohta et al. 2011). Other related signature genes in PP/gamma cells include *TPH1*, encoding a tryptophan hydroxylase essential for the initial catalysis of serotonin, and *SLC6A4*, a serotonin reuptake transporter. Serotonin colocalizes with insulin in beta cells and promotes GSIS (Paulmann et al. 2009). Mice lacking *TPH1* are diabetic and exhibit impaired insulin secretion due to a lack of pancreatic serotonin (Paulmann et al. 2009). Elevated *FEV*, *TPH1*, and *SLC6A4* expression suggests PP/gamma cells share a suite of characteristic genes with serotonergic neurons that, in the pancreas, integrate central and peripheral hunger and satiety cues. We also observed high PP/gamma expression of muscarinic acetylcholine receptor M3, *CHRM3*, which stimulates exocrine pancreatic amylase (Gautam et al. 2005), insulin secretion (Kong and Tobin 2011; Molina et al. 2014), and smooth muscle contraction and gastric emptying (Eglen et al. 1994). These data implicate the less abundant delta and PP/gamma cell types as critical for islet function via the integration of systemic cues and warrant further studies to elucidate the function and health of these cells in normal and diabetogenic conditions.

### Single-cell transcriptomes link rare and common diabetes genetic risk genes to islet cell types

We next sought to understand the cell type(s) involved in rare forms of diabetes, including transient/permanent neonatal diabetes (T/PNDM), CHI and MODY, as well as more common forms of islet dysfunction and diabetes (T1D/T2D). Monogenic diabetic disorders, including CHI, MODY, and neonatal diabetes, are characterized by mutations in a single gene, often resulting in beta cell dysfunction and death (Schwitzgebel 2014). Five monogenic diabetes risk genes (Supplemental Table S11; Hoffmann and Spengler 2012; Senniappan et al. 2013; Schwitzgebel 2014), were enriched in beta cells (i.e., greater than fourfold change in expression in specific islet cell type relative to other endocrine cells), including glucose transporter *SLC2A2* (data not shown), beta cell maturation transcription factor *PDX1*, and the sulfonylurea drug target *ABCC8* (Fig. 4C). *PDX1* expression has been reported in human (Li et al. 2016) and mouse (DiGruccio et al. 2016) beta and delta cells. Despite the modest number of delta cells sampled, our data also suggest moderate *PDX1* expression in delta cells (four of 16 delta cells with expression ≥16 CPM). Robust expression of *HADH* in both beta and delta cells (Fig. 4C) was confirmed by in situ (View RNA) analyses (Fig. 4E,F). Approximately 386/457 cells (84%) in *HADH* and *INS* labeled sections coexpressed both markers (shown in Fig. 4E). Adjacent *SST/HADH* colabeling yielded an approximately equal proportion (*n* = 255/306; 83%) of *SST*-negative/*HADH*-positive cells. Finally, 43/457 (9%) cells were *INS* negative/*HADH* positive, and 41/306 (13%) cells coexpressed *SST* and *HADH* (shown in Fig. 4F) in the respective serial sections. Another CHI-associated gene, *UCP2* (González-Barroso et al. 2008; Senniappan et al. 2013), which was reported to be highly expressed in human beta cells (Liu et al. 2013) and to suppress insulin secretion (Krauss et al. 2003), was enriched in delta cells (Fig. 4C). Delta cell expression of monogenic diabetes genes thus implicate this cell type in the molecular genetics of rare islet dysfunction and diabetes disorders, particularly CHI.

We also investigated cell type expression patterns of 536 islet expression quantitative trait loci (eQTL) target genes (Lyssenko et al. 2009; Dupuis et al. 2010; Dayeh et al. 2013; Fadista et al. 2014; Kulzer et al. 2014; van de Bunt et al. 2015). The majority of these genes (*n* = 309; Supplemental Table S11) were lowly expressed in both the endocrine islet single-cell transcriptomes and in the paired bulk islet transcriptomes (Supplemental Fig. S16A). One hundred fifty-nine additional genes did not exhibit a greater than or equal to fourfold expression change in any endocrine islet cell type. Of the remaining 68 eQTL genes, 54, 46, 51, and 43 were expressed in beta, alpha, delta, and PP/gamma cells, respectively. Surprisingly, beta and delta cells possessed the highest numbers of cell-type**–**specific eQTL genes (Supplemental Table S11).

Genome-wide association studies (GWAS) have identified more than 100 loci associated with T2D and related quantitative traits (Mohlke and Boehnke 2015). Because GWAS identify genetic variants associated with a disease, but not the specific gene(s) affected (Pearson and Manolio 2008; Manolio 2010), we took two approaches to assess cell-type expression of patterns of putative GWAS genes. First, we compiled and examined a list of 197 reported putative T1D and T2D GWAS genes (Bakay et al. 2013; Nica et al. 2013; Fadista et al. 2014; Marroqui et al. 2015; Mohlke and Boehnke 2015). Of these genes, 37 were expressed in beta, 24 in alpha, 28 in delta, and 22 in PP/gamma cells (Supplemental Table S11). Similarly, genes that were cell-type specific were expressed at higher levels in ND bulk intact islets compared with those genes without cell-type specificity (Supplemental Fig. S16B). Ten genes were uniquely expressed in beta cells, including *MEG3*, a type 1 diabetes (T1D)–associated lincRNA with reported expression in mouse beta cells and potential tumor suppressor activity (Modali et al. 2015), and *IAPP*, whose protein product, when aggregated, possesses cytotoxic properties that may contribute to beta cell death and dysfunction in T2D (Westermark et al. 2011). We also identified five putative T2D GWAS genes (including *HHEX*) to be uniquely expressed in delta cells. To conduct a more liberal analysis of putative GWAS genes, we identified all single-nucleotide polymorphisms (SNPs) associated with polygenic diabetes and related traits from the GWAS catalog (https://www.ebi.ac.uk/gwas/). For each reported SNP associated with T2D, T1D, fasting insulin, fasting glucose, and proinsulin, we examined the expression of all genes overlapping within one megabase of the chromosomal locus and identified 263 genes with cell-type**–**specific expression (Supplemental Table S12). Together, our observations of cell-type**–**specific expression of eQTL and monogenic and common (T2D GWAS) diabetes genes both confirm beta cell–specific expression of multiple diabetes-associated genes (*MEG3*, *DLK1*, *SLC2A2*, etc.) and implicate other cell types in the molecular genetic pathogenesis of diabetes. In light of recent studies (Zhang et al. 2014; DiGruccio et al. 2016) and our data, which suggest that delta cells may be critical regulators of glucose homeostasis and islet function, this provides a new avenue for investigation of T2D pathogenesis, as well as potentially new therapeutic targets and treatment options.

### Comparison of T2D and ND single-cell transcriptomes uncovers cell-type**–**specific differences not detected in whole islets

**Figure 5.**
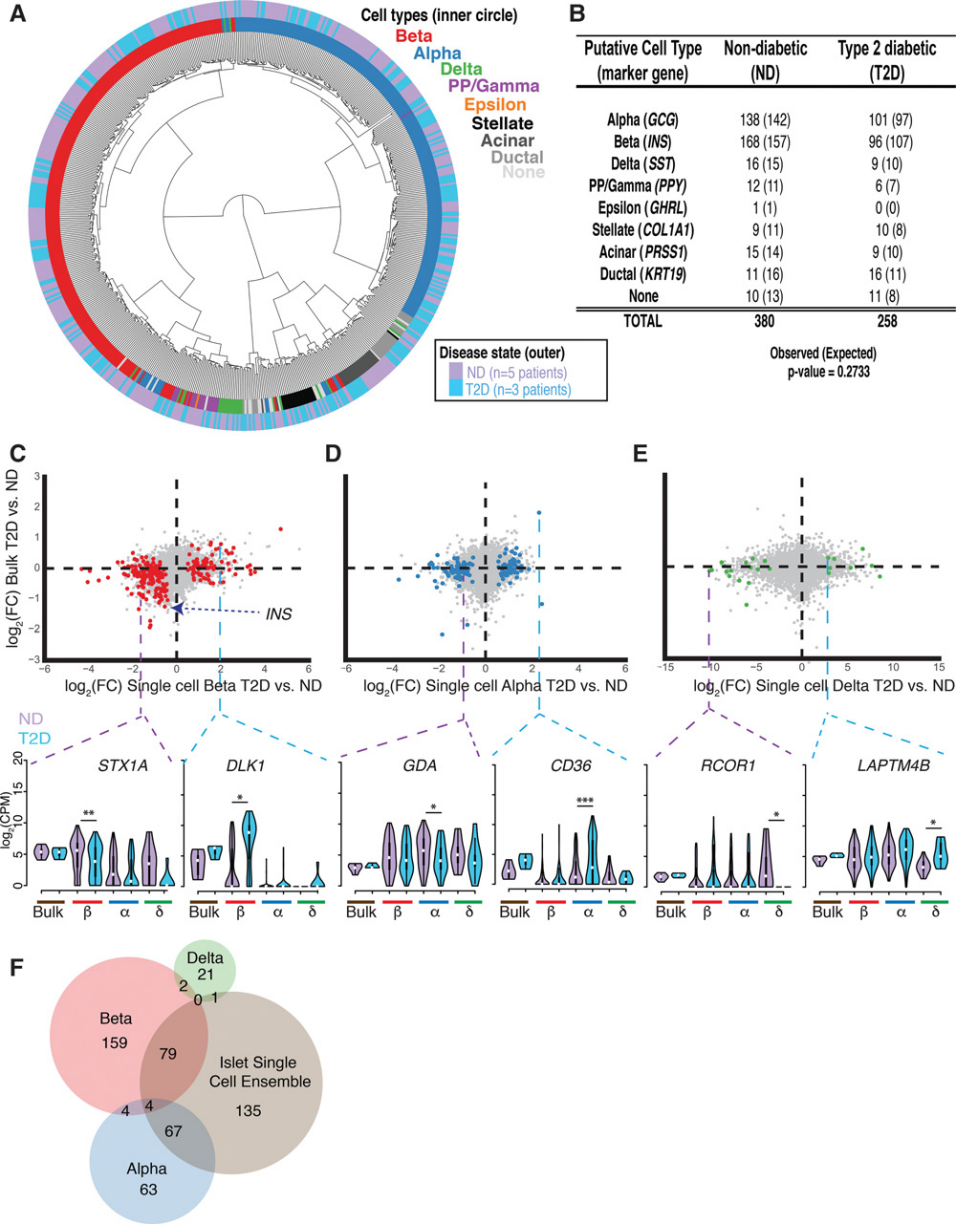
Single-cell transcriptome analyses identify cell-type–specific expression changes in T2D islets. (*A*) T2D and ND single-cell transcriptomes cluster together by cell type after unsupervised hierarchical clustering. (*B*) Number of each ND and T2D cell type classified by marker gene expression as shown in Figure 2. The numbers of cells expected in each condition based on a χ^2^ test are indicated in parentheses. (*C*–*E*, *top*) Scatter plots of log_2_ fold-change (FC) expression detected between T2D and ND samples from bulk intact RNA-seq (*y*-axis) and from Fluidigm C1 single-cell RNA-seq (*x*-axis) from beta cells (*left* plot; red), alpha cells (*middle* plot; blue), and delta cells (*right* plot; green). (*Bottom*) Violin plots highlight examples of differentially expressed genes in one single-cell type. Dashed purple lines represent repressed genes in the respective T2D cell type, while dashed blue lines represent induced genes. (*) FDR < 0.05, (**) FDR < 0.01, (***) FDR < 0.001. (*F*) Venn diagram showing the intersections of differentially expressed genes identified between T2D and ND transcriptomes at single-cell-type and islet single-cell ensemble resolution. The islet single-cell ensemble represents the pooled collection of beta, alpha, delta, and PP/gamma single cells.

Finally, we compared single-cell transcriptome profiles from T2D and ND donors to identify differentially regulated genes and obtain greater insight into the molecular genetic pathogenesis of diabetes. After unsupervised hierarchical clustering (Fig. 5A) and t-SNE analysis (Supplemental Figs. S17, S18) using 2754 of the most highly expressed genes (Supplemental Table S4), we observed that transcriptomes clustered by cell type regardless of disease state. As previously observed, clustering was not driven by marker gene expression (Supplemental Figs. S19, S20). For regions of the dendrogram (Fig. 5A) where samples appeared to cluster by disease state, we found that islet donor identity was an underlying factor that reflected sample subclustering (Supplemental Fig. S21). We obtained fewer beta cells among the T2D islet cells sampled compared with ND samples (Fig. 5B). However, observed differences in T2D and ND single-cell proportions did not differ significantly from expected cell-type proportions (Fig. 5B, χ^2^ *P*-value = 0.2733), and none of the islets from these newly diagnosed T2D individuals exhibited as significant a decrease as previously reported (Butler et al. 2003; Cnop et al. 2005; Donath et al. 2005; Prentki and Nolan 2006).

Recent studies have reported features of beta cell de-differentiation under diabetogenic and stress conditions (Talchai et al. 2012; Wang et al. 2014; Cinti et al. 2016). However, we did not identify significant shifts in islet cell populations, increases in number of hormone-negative “none” cells, or appearances of new or more abundant populations of cells in T2D islets that clustered distinctly from the known islet cell types in this study. Moreover, expression of reported de-differentiation genes including *FOXO1*, *NANOG*, and *POU5F1* (Talchai et al. 2012) did not differ significantly between T2D and ND islet cell types nor the paired bulk intact islet preparations (Supplemental Fig. S22). Finally, other de-differentiation markers such as *NEUROG3* and *MYCL* were not detected in our single-cell or bulk intact islet data. Thus, our analysis did not identify transcriptional evidence of de-differentiated cells in T2D islets.

Comparison of islet cell-type transcriptomes (e.g., T2D beta vs. ND beta) did, however, identify 410 genes that were differentially expressed (FDR < 5%) between T2D and ND donors (Supplemental Table S6) beta, (Fig. 5C, *n* = 248), alpha (Fig. 5D, *n* = 138), and delta cells (Fig. 5E, *n* = 24). We also identified differentially expressed genes in acinar (*n* = 74), ductal (*n* = 35), and stellate (*n* = 28) exocrine cell types (Supplemental Fig. S23; Supplemental Table S6). T2D beta cells exhibited a 1.4-fold decreased *INS* expression compared with ND beta cells (Fig. 5C). *STX1A* was significantly reduced (log_2_FC −1.5178) in T2D beta cells, consistent with reported decreases in STX1A protein levels in T2D beta cells (Andersson et al. 2012). STX1A combines with SNAP-25 and VAMP2 to form a tertiary SNARE protein complex important for insulin secretion in beta cells (Andersson et al. 2012), and *STX1A* inhibition drastically reduces GSIS and exocytosis (Vikman et al. 2006). Additionally, we detected elevated *DLK1* expression in T2D beta cells (log_2_FC 2.010), which has been implicated in T1D/T2D GWAS (Wallace et al. 2010) and is part of a dysregulated locus in T2D islets (Kameswaran et al. 2014). *Dlk1*^−/−^ mice exhibit increased glucose sensitivity and insulin secretion (Abdallah et al. 2015), and high levels of serum DLK1 have been associated with insulin resistance in both rodents and humans (Chacón et al. 2008). Immunofluorescence indicates that DLK1 is beta cell specific in human but not mouse islets (Li et al. 2016), and FACS-enriched mouse beta cells show low expression of *Dlk1* in comparison to other sorted islet alpha and delta cells (DiGruccio et al. 2016), potentially implicating a unique role of this gene in human T2D progression. These findings suggest that perturbations in *STX1A* and *DLK1* expression may contribute to the beta cell dysfunction and impaired insulin secretion that is commonly observed in T2D pathogenesis.

Decreased beta cell function and mass are hallmarks of T2D pathophysiology (Cerf 2013; Halban et al. 2014). Our analyses suggest that transcriptional changes in nonbeta cells may also contribute to T2D pathogenesis. Specifically, we highlight increased expression of fatty acid translocase gene *CD36* (log_2_FC 2.296), as well as decreased expression of the guanine deaminase gene, *GDA* (log_2_FC −1.062), in T2D alpha cells. Soluble CD36 is a biomarker of T2D (Alkhatatbeh et al. 2013) and diabetic nephropathy (Shiju et al. 2015) and coordinates activation of the NLRP3 inflammasome, leading to proinflammatory cytokine release and reduced insulin secretion (Sheedy et al. 2013). Within T2D delta cell transcriptomes, we note increased *LAPTM4B* expression (log_2_FC 2.871) and drastically reduced *RCOR1* expression (log_2_FC −10.128). The underlying biological significance of these differentially regulated genes remains unclear and thus requires further investigation of their roles in nonbeta cell types and T2D pathology. We also compared the transcriptional differences between T2D and ND endocrine cells without first segregating them into islet cell types (334 ND and 212 T2D single-cell profiles). Approximately 66% of beta cell–specific (*n* = 165/248), 50% of alpha cell–specific (*n* = 67/138), and >90% of delta–specific (*n* = 23/24) changes in gene expression were missed when cell types were not defined and specifically compared (Fig. 5F). The decreased heterogeneity in the transcriptional profiles of cell-type**–**specific comparisons provides increased power to detect the transcriptomic differences and argues the importance of single-cell analysis in understanding the molecular basis of T2D.

Recent islet single-cell studies emerged while this study was under review. We therefore sought to validate our observed cell-type–specific differences in T2D islets using these independent data sets (Wang et al. 2016; Segerstolpe et al. 2016). We found that 54/77 genes and 32/171 were also significantly up- and down-regulated, respectively, in T2D beta cells in these studies (*P* < 0.05, two-sided Wilcoxon rank-sum test) (Supplemental Fig. S24A,B; Supplemental Table S13). Notably, *DLK1* consistently exhibited approximately fourfold induction in T2D beta cells in each study (Supplemental Fig. S24C,D) Similarly, 39/60 and 14/78 genes were significantly induced or repressed, respectively, in T2D alpha cells (Supplemental Fig. S24E,F). This included approximately twofold *CD36* induction in each study (Supplemental Fig. S24G,H). Validation rates for delta cells was notably lower, likely due to the relatively few cells profiled for comparison. However, we did note a significant increase (log_2_FC 1.203) in *LAPTM4B* in T2D delta cells from Segerstolpe et al. (2016), consistent with our data.

## Discussion

In this study, we completed transcriptome profiling and analysis of 638 single islet cells from ND and T2D individuals. Single-cell RNA-seq protocols are often limited by their capture efficiency due to the fact that a limited proportion of each cell's total transcripts is represented in the final sequencing library (Liu and Trapnell 2016). Additionally, these approaches have difficulty detecting expression and changes in expression of low abundance transcripts. Despite these limitations, we observed a strong correlation between the transcriptomes of paired bulk islets and single cells, indicating these are high-quality and representative data sets. Based on single-cell transcriptome profiles, we have identified cells of each endocrine (alpha, beta, delta, PP/gamma, epsilon) and exocrine (stellate, ductal, acinar) type in the pancreas in an agnostic and data-driven manner.

This approach has defined expression signatures of each cell type with single-cell precision. Cell-type–specific expression patterns in our data such as *MAFA* in beta cells and *IRX2* in alpha cells are concordant with and extend those generated on a smaller set of cells and an independent platform (Li et al. 2016). Notably, our approach also uncovered important instances of shared expression between these cell types and the less common delta and PP/gamma islet populations, including genes mutated in CHI (*HADH*) and transcription factors regulating cell fate/identity (*ARX*). *HADH* encodes hydroxyacyl-CoA dehydrogenase, an important enzyme and negative regulator of glutamate dehydrogenase (GDH) and insulin secretion. Expression of *HADH* in islets has been shown to be beta cell specific (Kapoor et al. 2010; Pepin et al. 2010), and indeed, knockdown of *HADH* in rat 832/13 beta cells increases insulin secretion (Pepin et al. 2010). Surprisingly, our combined transcriptomic analyses and in situ (ViewRNA) validation of *HADH* revealed shared expression in beta and delta cells. These findings suggest delta cell dysfunction, in addition to beta cell dysfunction, as potential contributing factors to the development of monogenic diabetic disorders.

Most importantly, analysis of the delta and PP/gamma islet cell transcriptomes revealed cell-type**–**specific expression of multiple genes that suggest important roles for these cells in islet physiology and the molecular genetics of islet dysfunction in rare (e.g., PNDM, TNDM, and MODY) and common (e.g., T2D) forms of diabetes. The novel transcriptome signatures uncovered for human delta and PP/gamma cells includes genes that strongly suggest important roles for each cell type in sensing and integrating specific systemic cues to govern islet (dys)function. This clearly warrants additional work to better understand the regulation and function of these cells in normal and diabetic states. New cell surface markers identified for each of these cell types could be used to specifically enrich and purify these populations for detailed functional analysis.

Finally, by comparing single-cell transcriptomes from T2D and ND islets, we were able to study quantitative changes in cell populations and cell-type–specific expression in T2D pathogenesis. Although not reaching statistical significance, we did observe a trend of decreased beta cells in T2D islets versus ND islets. This difference was not as pronounced as in previous reports, possibly due to the relatively modest number of cells sampled per individual. Alternatively, as most of the T2D islet single-cell transcriptomes came from newly diagnosed individuals, this difference may also reflect the shorter duration or decreased severity of T2D in these samples compared with other studies. Previous studies suggested that beta cell de-differentiation may underlie beta cell loss in T2D (Talchai et al. 2012; Wang et al. 2014; Cinti et al. 2016). However, a subsequent study comparing human islets from 14 T2D and 13 ND individuals did not identify clear evidence of this phenomenon (Butler et al. 2016). Similarly, our data do not provide transcriptome-based evidence of *trans*-differentiation or de-differentiation phenomena in T2D islets. We observed neither the appearance of new or distinct subpopulations among the T2D islet single cells nor significant changes of reported de-differentiation genes between T2D and ND cell types (e.g., T2D beta cells vs. ND beta cells), although it is possible that de-differentiated cells were simply not captured in our study. Overall, we identify 248, 138, and 24 genes exhibiting differential expression in T2D versus ND beta, alpha, and delta cells, respectively. Consistent with Simpson's paradox, approximately half of these genes in each major islet cell type (64% beta, 45% alpha) and ∼90% of these in the less abundant delta cells were not detected in whole islet or single-cell islet transcriptomes when they were not stratified by cell type (Simpson 1951; Trapnell 2015). Each of these differentially regulated genes may represent important new candidate genes in T2D pathogenesis and therapeutic targeting.

## Methods

### Islet acquisition, processing, and dissociation

Islets were procured from ProdoLabs or the Integrated Islet Distribution Program (IIDP) and shipped in PIM(T) media (ProdoLabs) overnight on cold packs. Upon arrival, islets were washed and transferred into PIM(S) media with PIM(G) and PIM(ABS) supplements according to the manufacturer's instructions (ProdoLabs) and incubated at 37°C in a 5% CO_2_ tissue culture incubator. Twenty-four hours after transfer, approximately 500 islet equivalents (IEq) were aliquoted and centrifuged at 180*g* for 3 min at room temperature (RT). One aliquot (100 IEq) was immediately flash frozen (Fig. 1A, baseline), one (200 IEq) was resuspended in 1 mL Prodo-media (Fig. 1A, intact), and one (200 IEq) was resuspended in 1 mL Accutase (Innovative Cell Technologies) (Fig. 1A, dissociated and single cell) and incubated for 10 min in a 37°C water bath, with pipetting every 2 min. Accutase-dissociated cells were filtered through a prewet cell strainer (BD) to collect single cells, rinsed with 9 mL prewarmed CMRL + 10%FBS media to stop the reaction, and centrifuged at 180*g* for 3 min at RT. Dissociated cells were resuspended in 300 µL CMRL + 10%FBS media. Cell size, number, and viability were assessed using Countess II FL (Thermo Fisher Scientific), and the cell suspension was diluted to a final concentration of 300 cells/µL. Total processing and handling time for each islet was ≤60 min.

### Single-cell processing on the C1 single-cell Autoprep system

After counting, cells were diluted to a final concentration range of 250–400 cells/μL and 5 µL loaded onto each C1 integrated fluidic circuit (IFC; 10- to 17-μm chip) for cell capture on the C1 single-cell Autoprep system. For each islet preparation, up to two microfluidic chips were used. After capture, cells were imaged within each capture nest with an EVOS FL auto microscope (Life Technologies). IFCs were subsequently loaded with additional reagents for subsequent cell lysis; SMARTer v1- based (Clontech), olio-(dT)-primed reverse transcription; template switching for second-strand priming; and amplification of cDNA on the C1 System. Qualitative and quantitative analysis of all single-cell cDNA products was performed on a 96 capillary fragment analyzer (Advanced Analytical). Only cell singlets, as determined by imaging, with adequate cDNA yield and quality were processed for subsequent sequencing. Fragmentation and tagmentation of cDNA was done with Nextera XT reagent (Illumina) using dual indices to prepare single-cell multiplexed libraries.

### Bulk-cell RNA-seq

Bulk cells were pelleted and RNA purified using the PicoPure RNA isolation kit (Life Technologies). All RNA-seq libraries from bulk-sample RNA were generated with the same SMARTer v1 chemistry (Clontech) as for the C1 single-cell data largely following the manufacturer's instructions. Unlike the C1 workflow, after first-strand DNA synthesis, cDNA was purified using Agencourt AMPure beads (Beckman Coulter). cDNA was subsequently amplified through 12 PCR cycles. The cDNA yield and fragment size were measured on a 2100 Bioanalyzer (Agilent). For sequencing library preparation, amplified cDNA was sheared using a Covaris LE220 system to obtain fragments of ∼200 bp. The fragmented cDNA was prepared for sequencing using the NEBNext DNA library prep kit for Illumina sequencing (New England BioLabs).

### Sequencing, read mapping, and quality control

All sequencing was performed on a NextSeq500 (Illumina) using the 75-cycle high-output chip. RNA-seq reads were subjected to quality control using custom scripts developed at the computational sciences group at The Jackson Laboratory. Briefly, reads with >30% of bases with quality scores less than 30 were removed from the analysis, and samples with >50% of the low-quality reads were removed from the cohort. Trimmed reads were mapped to human transcriptome (GRCh37, Ensembl v70) using Bowtie 2 (Langmead and Salzberg 2012), and expression levels of all genes were estimated using RSEM (Li and Dewey 2011). Transcript per million (TPM) values as defined by RSEM were added a value of one prior to log__2__ transformation to avoid zeros. GRCh37 was selected for mapping to facilitate integration and comparative analyses with existing islet data sets (e.g., Parker et al. 2013; Fadista et al. 2014; van de Bunt et al. 2015) and ENCODE and NIH Roadmap data by members of the islet biology, diabetes, and functional genomics communities. The observation of expected “positive control” genes for each cell type strongly suggested that mapping to GRCh37 instead of GRCh38 did not mask or alter any important conclusions that could be drawn from the data.

### Single-cell sample processing and quality filtering

We used 26,616 protein-coding genes and lincRNAs from the GRCh37, Ensembl v70 build in our study. Genes with expression five or more TPMs in a sample were defined as expressed. Seventy-two single-cell samples that expressed fewer than 3500 genes according to these criteria were removed from downstream analysis.

### Islet cell type classification

GMM of islet marker genes was performed on a per gene basis using the R-package *mclust_5.2* (Scrucca et al. 2016). Each single-cell sample was classified as a specific pancreatic cell type if and only if a single gene from the selected marker gene list—*INS* (beta), *GCG* (alpha), *SST* (delta), *PPY* (PP/gamma), *KRT19* (ductal), *PRSS1* (acinar), and *COL1A1* (stellate)—was expressed in the sample and none of the other marker genes were expressed. Cells expressing no marker genes were labeled as “none,” and those expressing >1 marker gene were labeled as “multiple.” Fluidigm released a white paper report detailing the potential for single cells to “*z*-stack” in up to 30% of capture nests on the medium (10–17 µm) Fluidigm C1 chip (http://info.fluidigm.com/rs/673-MRG-416/images/C1-Med-96-IFC-Redesign_wp_101-3328B1_FINAL.pdf). DAPI staining identified *z*-stacked islet cell doublets in 10% and 30% of capture nests from two additional C1 single-cell captures. Because the proportion of “multiple” labeled cells approximately equaled that of *z*-stacked doublets, we discarded these cells (*n* = 340) from subsequent analyses.

### Unsupervised dimensionality reduction and hierarchical clustering

Barnes-Hut variant of t-SNE (van der Maaten 2014) was implemented using the R-package *Rtsne_0.10* (https://github.com/jkrijthe/Rtsne). ND single-cell transcriptomes were reduced to two dimensions in an unsupervised manner using genes with log_2_ CPM values greater than 10.5 in at least one sample. Similar analyses were repeated using only the T2D single-cell data and the combined single-cell data. Hierarchical clustering of cell transcriptomes was performed using Euclidean distance, Ward.D2 linkage, and the same gene selection criteria. Resultant “fan” dendrogram images were produced using the R-packages *dendextend_1.1.8* (Galili 2015) and *ape_3.5* (Paradis et al. 2004). Bulk islet transcriptomes were clustered using the same criteria.

### Supervised differential gene expression analysis

Differential expression analyses were performed using the Bioconductor package *edgeR_3.14.1* (Robinson et al. 2010). Gender was used as a blocking factor to account for variability between male and female patient islets. In each comparison, protein-coding genes and lincRNAs with 20 or fewer counts in at least 20% of either cell type population being compared or at least a minimum of three cells were used. Differentially expressed genes with FDR < 5% were regarded as significant results. Endocrine cell signature genes were identified by first performing the above differential expression analysis procedure between each endocrine cell type (e.g., beta vs. alpha, beta vs. delta, and beta vs. PP/gamma). Afterward, the intersection of these results was performed to identify genes exclusively enriched in the cell type. Exocrine cell signature genes were identified after pairwise comparisons between each respective exocrine cell type (e.g., acinar vs. stellate, acinar vs. ductal). Comparisons between T2D and ND single-cell transcriptomes were performed for the same cell types (e.g., T2D beta cells vs. ND aeta cells) to identify cell-type–specific differences in gene expression between T2D and ND states.

### ANOVA and post-hoc analyses

For each collection of diabetes-associated and eQTL genes examined, one-way analysis of variance (ANOVA) was used to identify statistically significant differences (FDR > 5%) in islet cell-type gene expression. Following this, we performed a post hoc analysis using a THSD test to determine genes with cell-type–specific expression patterns (fold change > 4). Genes were classified as “pan-islet” if they had an average log_2_(CPM) expression greater than four in all islet cell types. Genes that were not enriched in a cell type or pan-islet were classified as “lowly expressed” (average log_2_(CPM) < 2 in all cell types), and the remaining genes were classified as “less than fourfold change.” This same methodology was used to characterize expression of the previously reported alpha- and beta-specific genes from Dorrell et al. (2011b), Bramswig et al. (2013), Nica et al. (2013), and Blodgett et al. (2015). Similar methods were used to characterize expression patterns of genes nearby diabetes-related GWAS SNPs (downloaded from the GWAS Catalog, https://www.ebi.ac.uk/gwas/, and available in Supplemental Table S12). Protein-coding RNAs and lincRNAs that overlapped within one megabase upstream of and downstream from the diabetes-associated SNPs were analyzed.

### Gene set enrichment analysis

The Bioconductor package *gage_2.22.0*, (Luo et al. 2009) was used with default parameters to identify enrichment (FDR < 5%) of human Kyoto Encyclopedia of Genes and Genomes (KEGG) pathways in each of the ND islet cell transcriptomes. Enriched pathways were determined by comparing each cell-type transcriptome against the other aggregate islet cell-type transcriptomes (e.g., beta vs. alpha, delta, and PP/gamma).

### RNA in situ hybridization

RNA transcripts were visualized in OCT-embedded pancreatic islet sections from two ND donors (P3 and P4) using QuantiGene ViewRNA ISH cell assay kit (catalog no. QVC0001, Affymetrix). Human *INS* ViewRNA type 6 probe (Catalog no. VA6-13248-06), *SST* ViewRNA type 6 probe (catalog no. VA6-17244-06), *LEPR* ViewRNA type 1 probe (catalog no. VA1-15221-06), and *HADH* ViewRNA type 1 probe (catalog no. VA1-12106-06) were purchased from Affymetrix (Santa Clara). The assay was performed according to the cell-based ViewRNA assay protocol with a 15-min formaldehyde fixation and a 10-min protease treatment (dilution factor 1:4000). ViewRNA probes were detected at 550 nm (Cy3) and 650 nm (Cy5) using a Leica TSC SP8 confocal microscope at 63× magnification.

### Islet cell subpopulation analyses

Dorrell et al. 2016 previously defined four beta cell subpopulations with differing expression of 59 genes. With this gene set, we attempted to validate the presence of these four subpopulations via unsupervised t-SNE and hierarchical clustering of all ND beta cell transcriptomes (*n* = 168). Similarly, Bader et al. (2016) characterized proliferative (*Fltp*^+^/FVR^+^) and mature (*Fltp*^−^/FVR^−^) mouse beta cells that showed differential expression of 996 transcripts. By using the Mouse Genome Informatics (MGI; http://www.informatics.jax.org) database, these 996 transcripts corresponded to 691 human orthologs that were detected in our data set. Beta cell transcriptomes were clustered using these orthologs to attempt to identify mature and proliferating subpopulations. Finally, we used the functions available in *scran_1.04* (http://bioconductor.org/packages/release/bioc/html/scran.html) to computationally assign single-cell samples into cell cycle phases (G1, G2/M, or S phase) and investigate whether our data set contained proliferating islet cells.

## Data access

Raw sequence data from this study have been submitted to the databases of NCBI Sequence Read Archive (SRA; http://www.ncbi.nlm.nih.gov/sra) under accession number SRP075970 and BioProject (http://www.ncbi.nlm.nih.gov/bioproject/) under accession number PRJNA323853. Processed data sets from this study have been submitted to Gene Expression Omnibus (GEO; http://www.ncbi.nlm.nih.gov/geo/) under accession number GSE86473. UCSC Genome Browser tracks of aggregate ND islet single-cell-type transcriptomes are available at http://genome.ucsc.edu/ and may be accessed with the user name “lawlorn” and session name “Islet_Single_Cell_Type_Transcriptomes.” The source code used to produce the figures and tables in this paper is available in the Supplemental_Methods_Source_Code folder as suggested by Hoffman (2016).

## Supplementary Material

Supplemental Material
