# Supplementary material for: Single-cell transcriptomes identify human islet cell signatures and reveal cell-type–specific expression changes in type 2 diabetes
Source: Genome Res. 2017 Feb;27(2):208–22. doi: 10.1101/gr.212720.116 (PMC5287227; doi:10.1101/gr.212720.116)
Supplement: Supplemental Material [file supp_gr.212720.116_Supplemental_Methods_Source_Code.zip › Supplemental_Methods_Source_Code/Figure1_Source_Code/Figure_1B_Source_Code.pdf]

Unsupervised Hierarchical Clustering of Patient Islet Samples

Introduction

This report describes the steps used to perform unsupervised hierarchical clustering of the patient bulk islet samples. For each patient islet, we defined three different types of "bulk" islet samples. First, the "baseline" bulk islet represents the initial flash frozen whole islet sample that remained unaltered prior to RNA sequencing. Second, the "dissociated" islet represents a portion of the baseline islet treated with the enzyme Accutase to dissociate into a collection of single cells. Third, the "intact" islet represents another portion of whole islet sample that was incubated and processed in parallel to the dissociated islets. For each of the 8 patients, there was a specific "baseline", "intact", and "dissociated" bulk islet sample. These samples were clustered in an unsupervised manner to illustrate that there was no variation in gene expression within each patient bulk sample. In other words, our experimental process and preparation of dissociated single cells from bulk intact islets did not dramatically alter the transcriptomes of the samples.

Preprocessing Steps

All 24 bulk islet samples were clustered using genes with a log2(CPM) value of 10 in at least one sample. A total of 181 genes were used to cluster the samples. The resulting dendrogram is shown below.

```
# Load libraries
suppressPackageStartupMessages(library(Biobase))
suppressPackageStartupMessages(library(ape))
suppressPackageStartupMessages(library(RColorBrewer))
suppressPackageStartupMessages(library(edgeR))
suppressPackageStartupMessages(library(readxl))
library(Biobase)
library(edgeR)
library(ape)
library(RColorBrewer)
library(readxl)

rm(list=ls())
setwd("/Users/lawlon/Documents/RNA-seq/RNA-seq Data/Bulk Islet Data/")
# Load the bulk sample expression data
load("islet_bulk_uniq_data.rdata")
# Obtain the bulk sample annotation information
setwd("/Users/lawlon/Documents/Final_RNA_Seq_3/Supplemental_Tables/")
sample.anns.sel <- read_excel("Supplemental_Table_S1_Patient_Islet_Metadata.xlsx",
                              col_names = TRUE, skip = 1)
# Obtain the counts for each bulk sample
bulk.counts <- exprs(bulk.cnts)

# Calculate cpm of data
cpms <- cpm(x = bulk.counts)
log.cpm <- log2(cpms+1)

#Data frame of samp id, type (baseline, intact, dissociate), sex, and race
annotation_col = data.frame(Sex = sample.anns.sel$Sex,
            Race = sample.anns.sel$Race,
```

```r
            Phenotype = sample.anns.sel$Phenotype)

# Add sample labels to annotation data frame
rownames(annotation_col) = colnames(bulk.counts)

# Obtain genes that have a maximum log2 (CPM) of 10 or more in one sample
r.max <- apply(log.cpm,1,max)
mat <- log.cpm[r.max > 10,]

# Change Type to abbreviations
Type <- as.character(sample.anns.sel$Type)

# Add labels to new matrix
colnames(mat) <- paste(sample.anns.sel$`Patient Number`,Type,sep=".")
rownames(annotation_col) = colnames(mat)

# Assign a specific color to each patient
colorcodes <- c(P1 = "#1b9e77", P2 = "#d95f02", P3 = "#7570b3", P4 = "#e7298a",
                P5 = "#66a61e", P6 = "#e6ab02", P7 = "#a6761d", P8 = "#666666")

# Create hclust object
d <- dist(t(mat))
hc <- hclust(d, method = "ward.D2")
phy <- as.phylo(hc)

# Create a vector of color labels
cols <- NULL
# Match up colors with patient labels
for (i in 1:length(phy$tip.label)) {
  if (grepl(x = phy$tip.label[i], pattern = "P1") == TRUE) {
    cols <- c(cols,as.character(colorcodes["P1"]))
  } else if (grepl(x = phy$tip.label[i], pattern = "P2") == TRUE) {
    cols <- c(cols,as.character(colorcodes["P2"]))
  } else if (grepl(x = phy$tip.label[i], pattern = "P3") == TRUE) {
    cols <- c(cols,as.character(colorcodes["P3"]))
  } else if (grepl(x = phy$tip.label[i], pattern = "P4") == TRUE) {
    cols <- c(cols,as.character(colorcodes["P4"]))
  } else if (grepl(x = phy$tip.label[i], pattern = "P5") == TRUE) {
    cols <- c(cols,as.character(colorcodes["P5"]))
  } else if (grepl(x = phy$tip.label[i], pattern = "P6") == TRUE) {
    cols <- c(cols,as.character(colorcodes["P6"]))
  } else if (grepl(x = phy$tip.label[i], pattern = "P7") == TRUE) {
    cols <- c(cols,as.character(colorcodes["P7"]))
  } else if (grepl(x = phy$tip.label[i], pattern = "P8") == TRUE) {
    cols <- c(cols,as.character(colorcodes["P8"]))
  }
}

# Plot the dendrogram
plot(phy, tip.color = cols, cex = 1.5)
```

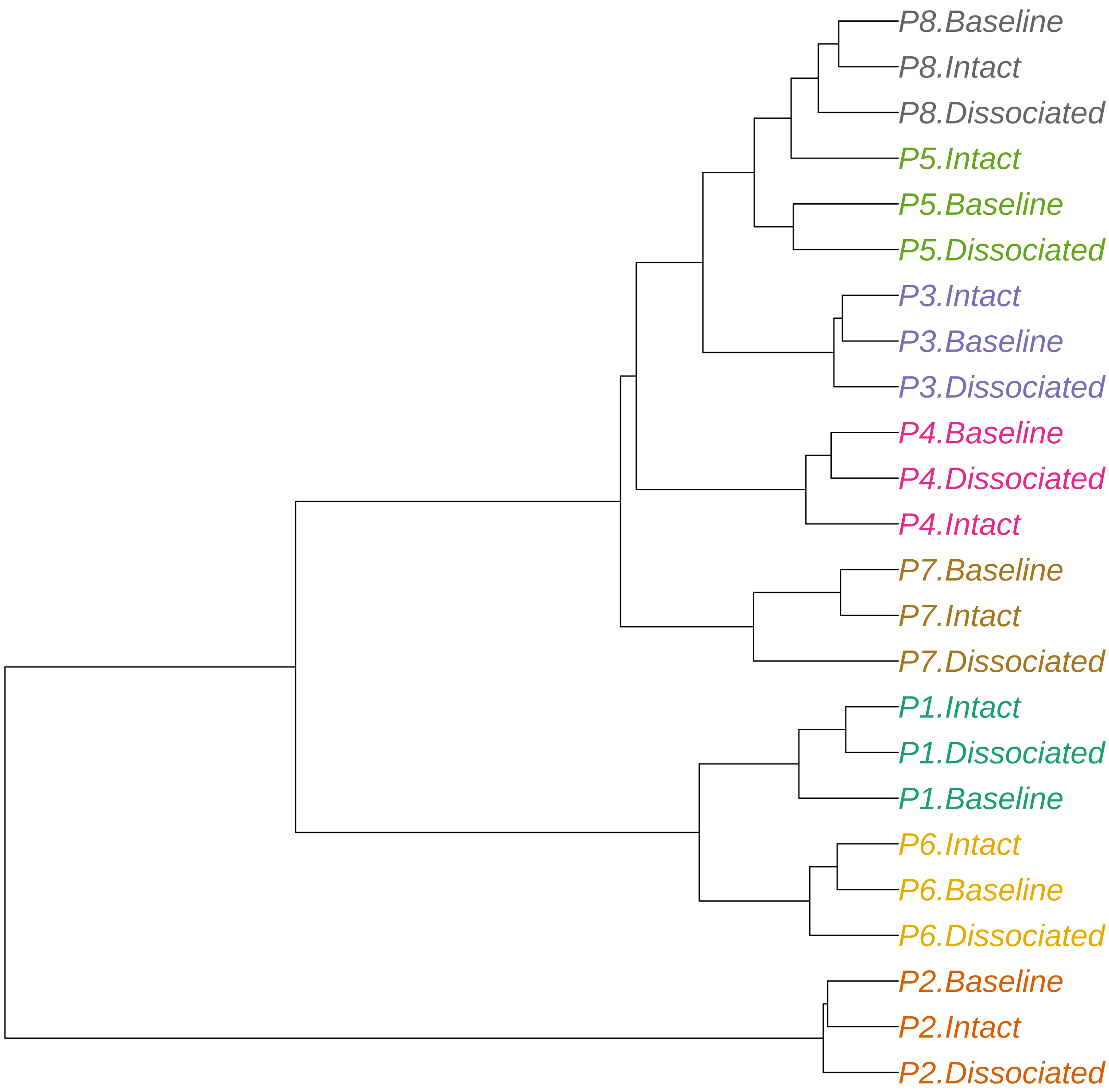

Figure 1: Unsupervised hierarchical clustering of patient islets using genes with log2(CPM) values greater than 10 in at least one sample.

Session Information

```
suppressPackageStartupMessages(library(Biobase))
suppressPackageStartupMessages(library(ape))
suppressPackageStartupMessages(library(RColorBrewer))
suppressPackageStartupMessages(library(edgeR))
suppressPackageStartupMessages(library(readxl))
library(Biobase)
library(edgeR)
library(ape)
library(RColorBrewer)
library(readxl)
sessionInfo()
```

```
## R version 3.3.0 (2016-05-03)
## Platform: x86_64-apple-darwin13.4.0 (64-bit)
## Running under: OS X 10.11.3 (El Capitan)
##
## locale:
## [1] en_US.UTF-8/en_US.UTF-8/en_US.UTF-8/C/en_US.UTF-8/en_US.UTF-8
##
## attached base packages:
## [1] parallel  stats     graphics  grDevices utils     datasets  methods
## [8] base
##
## other attached packages:
## [1] readxl_0.1.1        edgeR_3.14.0        limma_3.28.7
## [4] RColorBrewer_1.1-2  ape_3.5             Biobase_2.32.0
## [7] BiocGenerics_0.18.0
##
## loaded via a namespace (and not attached):
##  [1] Rcpp_0.12.5     lattice_0.20-33 digest_0.6.9    grid_3.3.0
##  [5] nlme_3.1-128    formatR_1.4     magrittr_1.5    evaluate_0.9
##  [9] stringi_1.1.1   rmarkdown_0.9.6 tools_3.3.0     stringr_1.0.0
## [13] yaml_2.1.13     htmltools_0.3.5 knitr_1.13
```
