# Supplementary material for: Single-cell transcriptomes identify human islet cell signatures and reveal cell-type–specific expression changes in type 2 diabetes
Source: Genome Res. 2017 Feb;27(2):208–22. doi: 10.1101/gr.212720.116 (PMC5287227; doi:10.1101/gr.212720.116)
Supplement: Supplemental Material [file supp_gr.212720.116_Supplemental_Methods_Source_Code.zip › Supplemental_Methods_Source_Code/Figure1_Source_Code/Figure_1C_Source_Code.pdf]

C1 Single Cell Sample Preprocessing

Introduction

26,616 protein coding genes and long non-coding RNAs (lincRNAs) from the ENSEMBL build 70 were used in our study. Genes with expression levels greater than or equal to 5 counts in a sample were considered to be expressed. 72 single cell samples which expressed fewer than 3500 genes according to these criteria, were removed from downstream analysis leaving 978 samples.

```
rm(list = ls())
setwd("/Users/lawlon/Documents/Final_RNA_Seq_2/Raw_Data/")
suppressPackageStartupMessages(library(Biobase))
suppressPackageStartupMessages(library(ggplot2))
suppressPackageStartupMessages(library(RColorBrewer))
library(RColorBrewer)
library(Biobase)
library(ggplot2)
# Load raw data for each single cell sequencing run
load("Human_islet_1st_run_normalized_expression_data.rdata")
load("Human_islet_2nd_run_normalized_expression_data.rdata")
load("Human_islet_4th_run_normalized_expression_data.rdata")
load("Human_islet_5th_run_normalized_expression_data.rdata")
load("Human_islet_6th_run_normalized_expression_data.rdata")
load("Human_islet_8_9th_run_normalized_expression_data.rdata")
load("Human_islet_c9L_run_normalized_expression_data.rdata")
load("Human_islet_c9R_run_normalized_expression_data.rdata")

# extract expression data
first.cnts <- exprs(islet_1st_eset)
second.cnts <- exprs(islet_2nd_eset)
fourth.cnts <- exprs(islet_4th_eset)
fifth.cnts <- exprs(islet_5th_eset)
sixth.cnts <- exprs(islet_6th_eset)
eight.9.cnts <- exprs(islet_8_9th_eset)
c9L.cnts <- exprs(islet_c9L_eset)
c9R.cnts <- exprs(islet_c9R_eset)

# gene annotation data
probe.anns <- as(featureData(islet_1st_eset),"data.frame")
# combine all expression data
all.cnts.1 <- cbind(cbind(cbind(cbind(cbind(first.cnts,second.cnts),fourth.cnts),
                                fifth.cnts),sixth.cnts),eight.9.cnts)

# Find which are not repeats, the C9R and C9L contain repeat samples which are ND samples only
  # Any ND samples in C9R and C9L are repeats
c9L.r <- which(grepl(colnames(c9L.cnts), pattern = "9th") == TRUE)
c9R.r <- which(grepl(colnames(c9R.cnts), pattern = "9th") == TRUE)

# Remove repeat samples
c9L.new <- c9L.cnts[, -c9L.r]
c9R.new <- c9R.cnts[, -c9R.r]
```

```r
# dataset without repeats
all.new <- cbind(cbind(all.cnts.1, c9L.new), c9R.new)

# Only use protein coding and lincRNAs
probes.sel <- probe.anns[probe.anns$Gene.Biotype %in% c("lincRNA", "protein_coding"),]
p.anns <- probes.sel
all.new <- all.new[rownames(probes.sel),]

# Code to binarize expression data using expression data
all.bin <- all.new
all.bin[all.bin < 5] <- 0
all.bin[all.bin >= 5] <- 1
num.exp <- apply(all.bin,2,sum)

# keep samples with greater than 3500 expressed genes
all.bin.sel <- all.bin[,num.exp > 3500]
numGenes <- apply(all.bin.sel,2,sum)
num.samples.exp <- apply(all.bin.sel,1,sum)
exp <- all.new[,num.exp > 3500]

# identify which samples had less than 3500 expressed genes
samps <- which(num.exp < 3500)

# set color panel
grey <- brewer.pal(n=9, name="Greys")
```

```r
# Overlay the histograms
hist(num.exp, breaks = 40, col = "blue", main="", ylab = "Cells (n)",
     xlab = "Number of genes expressed")
hist(num.exp[samps], breaks = 10, col = grey[7], main="", ylab="Cells (n)",
     xlab= "Number of genes expressed", add = TRUE)
abline(v=3500, col = "red", lty = 2)
text(1500,40, paste("n = ", length(samps), sep = ""), col = grey[7], cex = 2)
text(9600,60, paste("n = ", dim(all.bin.sel)[2], sep = ""), col = "blue", cex = 2)
```

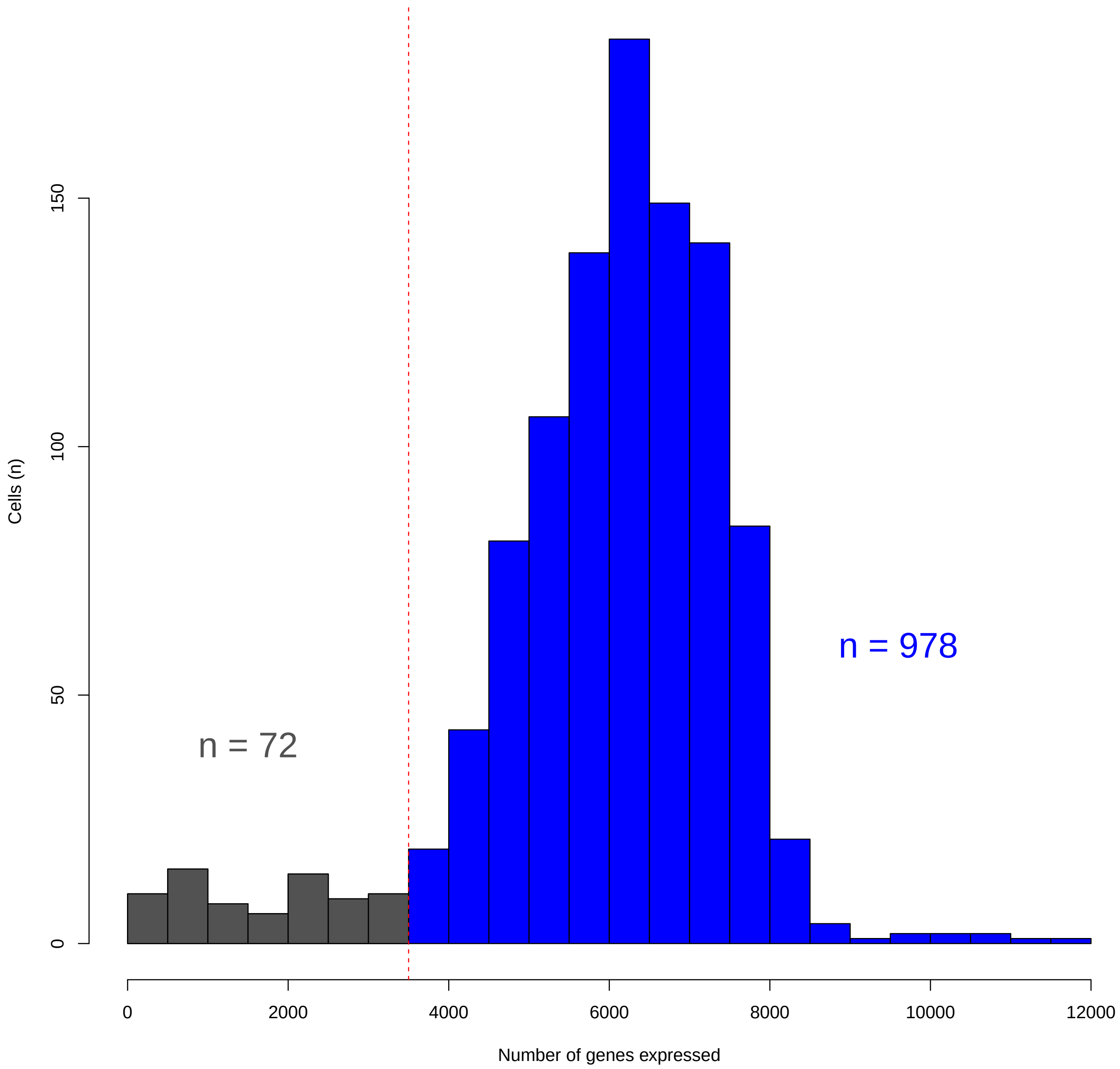

Figure 1: Histogram demonstrating the number of genes detected in each single cell. Cells expressing less than 3500 genes (n = 72) were removed from downstream analysis.

Session Information

```r
sessionInfo()
```

```
## R version 3.3.0 (2016-05-03)
## Platform: x86_64-apple-darwin13.4.0 (64-bit)
## Running under: OS X 10.11.3 (El Capitan)
##
## locale:
## [1] en_US.UTF-8/en_US.UTF-8/en_US.UTF-8/C/en_US.UTF-8/en_US.UTF-8
##
## attached base packages:
## [1] parallel  stats     graphics  grDevices utils     datasets  methods
## [8] base
##
## other attached packages:
## [1] RColorBrewer_1.1-2  ggplot2_2.1.0       Biobase_2.32.0
## [4] BiocGenerics_0.18.0
##
## loaded via a namespace (and not attached):
##  [1] Rcpp_0.12.5      digest_0.6.9     plyr_1.8.4       grid_3.3.0
##  [5] gtable_0.2.0     formatR_1.4      magrittr_1.5     scales_0.4.0
##  [9] evaluate_0.9     stringi_1.1.1    rmarkdown_0.9.6  tools_3.3.0
## [13] stringr_1.0.0    munsell_0.4.3    yaml_2.1.13      colorspace_1.2-6
## [17] htmltools_0.3.5  knitr_1.13
```
