# Supplementary material for: Single-cell transcriptomes identify human islet cell signatures and reveal cell-type–specific expression changes in type 2 diabetes
Source: Genome Res. 2017 Feb;27(2):208–22. doi: 10.1101/gr.212720.116 (PMC5287227; doi:10.1101/gr.212720.116)
Supplement: Supplemental Material [file supp_gr.212720.116_Supplemental_Methods_Source_Code.zip › Supplemental_Methods_Source_Code/Figure1_Source_Code/Figure_1D_Source_Code.pdf]

Bulk Intact vs Ensemble Single Cell Transcriptome Correlation

Scatter plot of Bulk Intact and Ensemble Single Cell Transcriptomes

This report will mkae a scatter plot comparing log2 transcripts per million (TPM) of intact islet bulk RNA-seq (n = 5) and ensemble single cell (n = 978) RNA-seq data. Overall, the correlation between the two groups demonstrates high (rsq=0.87, p<0.001) Pearson's correlation.

```
rm(list = ls())
suppressPackageStartupMessages(library(Biobase))
suppressPackageStartupMessages(library(ggplot2))
suppressPackageStartupMessages(library(edgeR))
library(Biobase)
library(ggplot2)
library(edgeR)
# Load in NonT2D single cell data
setwd("/Users/lawlon/Documents/Final_RNA_Seq_3/Data/")
load("nonT2D.rdata")
sc <- exprs(cnts.eset)
sc.anns <- pData(cnts.eset)
sc.nonT2D.sel <- sc
# Load in T2D data
load("T2D.rdata")
sc <- exprs(cnts.eset)
sc.anns <- pData(cnts.eset)
sc.T2D.sel <- sc
# Combine the single cell data
sc <- cbind(sc.T2D.sel,sc.nonT2D.sel)
# Take averages across genes
sc.r.mu <- apply(sc,1,mean)
# Log transform the data
sc.r.mu.log2 <- log2(sc.r.mu+1)

# Load in bulk islet expression data
load("/Users/lawlon/Documents/Final_RNA_Seq/islet_bulk_uniq_data.rdata")
bulk.anns <- pData(bulk.cnts)
# Extract expression data for bulk intact samples
type <- which(bulk.anns$Type == "Intact")
bulk <- exprs(bulk.cnts)
bulk <- bulk[,type]
# Take averages across genes
bulk.r.mu <- apply(bulk,1,mean)
# Log2 transform the data
bulk.r.mu.log2 <- log2(bulk.r.mu +1)

res <- data.frame(bulk = bulk.r.mu.log2, sc = sc.r.mu.log2)

# R-squared to be displayed on plot
```

```r
Rsq <- cor(sc.r.mu.log2, bulk.r.mu.log2)**2
msg <- paste("Rsq = ",format(Rsq,digits=2),sep="")

# Make scatter plot
ggplot(res, aes(x=bulk, y=sc)) + geom_point(shape=19,alpha=1/4,col="dodgerblue4",cex=.8) +
                annotate("text", x = 20, y = 5, label = msg)
```

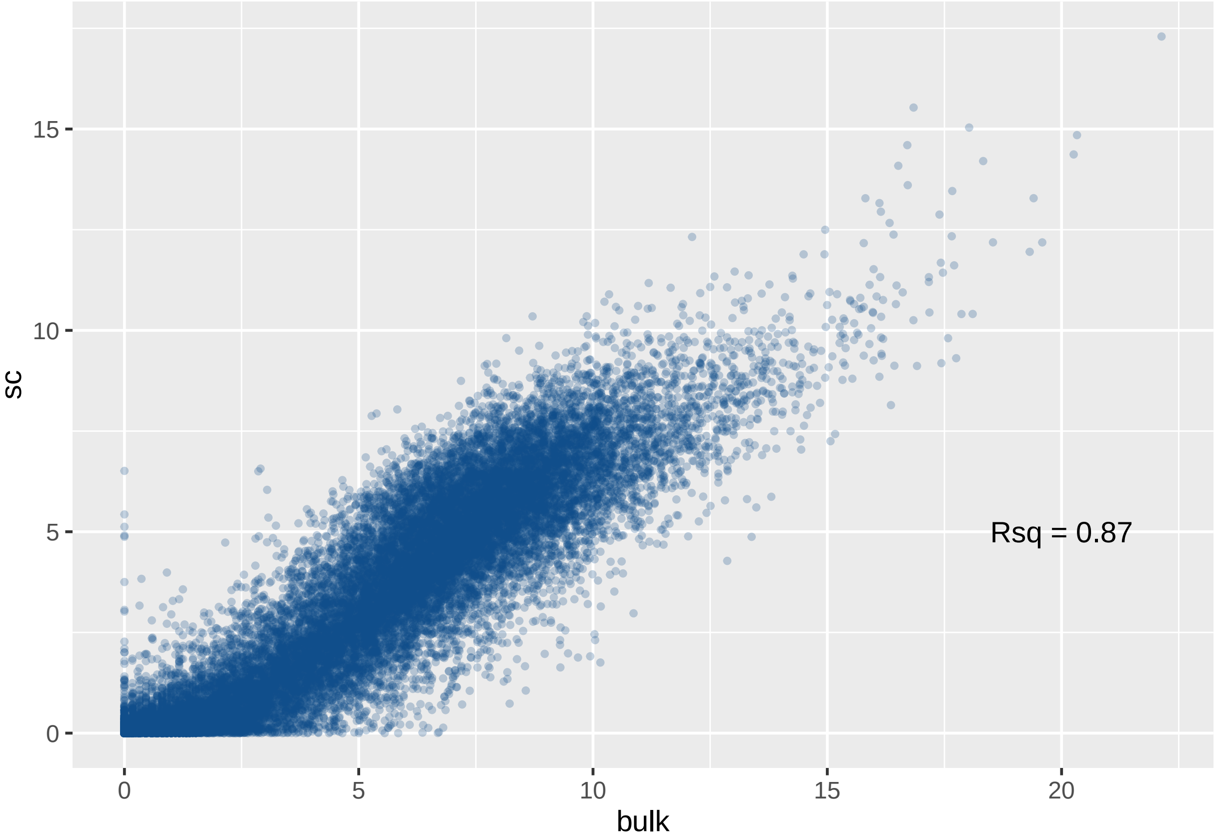

Session Information

```r
suppressPackageStartupMessages(library(Biobase))
suppressPackageStartupMessages(library(ggplot2))
suppressPackageStartupMessages(library(edgeR))
library(Biobase)
library(ggplot2)
library(edgeR)
sessionInfo()
```

```
## R version 3.3.0 (2016-05-03)
## Platform: x86_64-apple-darwin13.4.0 (64-bit)
## Running under: OS X 10.11.3 (El Capitan)
##
## locale:
## [1] en_US.UTF-8/en_US.UTF-8/en_US.UTF-8/C/en_US.UTF-8/en_US.UTF-8
##
## attached base packages:
```

```
## [1] parallel  stats     graphics  grDevices utils     datasets  methods
## [8] base
##
## other attached packages:
## [1] edgeR_3.14.0        limma_3.28.7        ggplot2_2.1.0
## [4] Biobase_2.32.0      BiocGenerics_0.18.0
##
## loaded via a namespace (and not attached):
##  [1] Rcpp_0.12.5      digest_0.6.9     plyr_1.8.4       grid_3.3.0
##  [5] gtable_0.2.0     formatR_1.4      magrittr_1.5     scales_0.4.0
##  [9] evaluate_0.9     stringi_1.1.1    rmarkdown_0.9.6  labeling_0.3
## [13] tools_3.3.0      stringr_1.0.0    munsell_0.4.3    yaml_2.1.13
## [17] colorspace_1.2-6 htmltools_0.3.5  knitr_1.13
```
