# Supplementary material for: Single-cell transcriptomes identify human islet cell signatures and reveal cell-type–specific expression changes in type 2 diabetes
Source: Genome Res. 2017 Feb;27(2):208–22. doi: 10.1101/gr.212720.116 (PMC5287227; doi:10.1101/gr.212720.116)
Supplement: Supplemental Material [file supp_gr.212720.116_Supplemental_Methods_Source_Code.zip › Supplemental_Methods_Source_Code/Figure2_Source_Code/Figure_2A_Source_Code.pdf]

Figure2_gaussian

June 21, 2016

In [1]:
```
%matplotlib inline
import pandas as pd
import numpy as np
import matplotlib
import matplotlib.pyplot as plt
from matplotlib.backends.backend_pdf import PdfPages
import scipy.stats as stats
import scipy.cluster.hierarchy as sch
from operator import *
pd.core.config.option_context('mode.use_inf_as_null',True)
import seaborn as sns
rcdefsns = plt.rcParams.copy()
import brewer2mpl
import os
import sys

matplotlib.rcParams['axes.linewidth'] = 3
matplotlib.rcParams['axes.edgecolor'] = 'k'
matplotlib.rcParams['axes.spines.top']='False'
matplotlib.rcParams['axes.spines.right']='False'
matplotlib.rcParams['axes.facecolor']='white'
```

In [11]:
```
islet_bulk_uniq=pd.read_csv('islet_bulk_uniq_data_cnts.csv',index_col=0)
islet_bulk_uniq_cpm=(islet_bulk_uniq/islet_bulk_uniq.sum())*1e6

t2d=pd.read_csv('../New.T2D-Counts.csv',index_col=0)
t2d_cpm=(t2d/t2d.sum())*1e6

nd=pd.read_csv('../New.NonT2D-Counts.csv',index_col=0)
nd_cpm=(nd/nd.sum())*1e6

islet_bulk_phenoData=pd.read_csv('islet_bulk_uniq_phenodata.csv',index_col=0)
islet_bulk_fetureData=pd.read_csv('islet_bulk_uniq_featuredata.csv',index_col=0)

t2d_phenoData=pd.read_csv('../New.T2D-CellClassification.csv',index_col=0)
nd_phenoData=pd.read_csv('../NewNonT2D-CellClassification.csv',index_col=0)
```

In [12]:
```
idx_T2D_intact=islet_bulk_phenoData[(islet_bulk_phenoData['Phenotype']=='T2D') & (islet_bulk_pl
idx_ND_intact=islet_bulk_phenoData[(islet_bulk_phenoData['Phenotype']=='ND') & (islet_bulk_phen
```

In [13]:
```
idx_beta_cells_t2d=t2d_phenoData[t2d_phenoData['cell.type']=='INS'].index
idx_alpha_cells_t2d=t2d_phenoData[t2d_phenoData['cell.type']=='GCG'].index
idx_gamma_cells_t2d=t2d_phenoData[t2d_phenoData['cell.type']=='PPY'].index
idx_delta_cells_t2d=t2d_phenoData[t2d_phenoData['cell.type']=='SST'].index
```

```
idx_beta_cells_nd=nd_phenoData[nd_phenoData['cell.type']=='INS'].index
idx_alpha_cells_nd=nd_phenoData[nd_phenoData['cell.type']=='GCG'].index
idx_gamma_cells_nd=nd_phenoData[nd_phenoData['cell.type']=='PPY'].index
idx_delta_cells_nd=nd_phenoData[nd_phenoData['cell.type']=='SST'].index
idx_acinar_cells_nd=nd_phenoData[nd_phenoData['cell.type']=='PRSS1'].index
idx_stellate_cells_nd=nd_phenoData[nd_phenoData['cell.type']=='COL1A1'].index
idx_dutcal_cells_nd=nd_phenoData[nd_phenoData['cell.type']=='KRT19'].index
```

```
In [15]: t2d_means_by_celltype=pd.DataFrame(index=t2d.index,columns=['INS','GCG','PPY','SST'])
         t2d_means_by_celltype['INS']=(t2d_cpm[idx_beta_cells_t2d]+1.).apply(np.log2).apply(np.mean,1)
         t2d_means_by_celltype['GCG']=(t2d_cpm[idx_alpha_cells_t2d]+1.).apply(np.log2).apply(np.mean,1)
         t2d_means_by_celltype['PPY']=(t2d_cpm[idx_gamma_cells_t2d]+1.).apply(np.log2).apply(np.mean,1)
         t2d_means_by_celltype['SST']=(t2d_cpm[idx_delta_cells_t2d]+1.).apply(np.log2).apply(np.mean,1)
```

Colors and Genes

```
Red (#e41a1c) = Beta
Blue (#377eb8) = Alpha
Green (#4daf4a) = Delta
Purple = (#984ea3) Gamma
Orange = (#ff7f00) Epsilon/Ghrelin
Black = (#000000) Stellate - COL1A1
Grey = (#525252) Acinar - PRSS1
Grey = (#969696) Ductal - KRT19
Grey = (#D9D9D9) Nones
```

```
In [16]: ins_ensg=islet_bulk_fetureData[islet_bulk_fetureData['Associated.Gene.Name']=='INS'].index[0]
         gcg_ensg=islet_bulk_fetureData[islet_bulk_fetureData['Associated.Gene.Name']=='GCG'].index[0]
         ppy_ensg=islet_bulk_fetureData[islet_bulk_fetureData['Associated.Gene.Name']=='PPY'].index[0]
         sst_ensg=islet_bulk_fetureData[islet_bulk_fetureData['Associated.Gene.Name']=='SST'].index[0]
         col1a1_ensg=islet_bulk_fetureData[islet_bulk_fetureData['Associated.Gene.Name']=='COL1A1'].inde
         krt19_ensg=islet_bulk_fetureData[islet_bulk_fetureData['Associated.Gene.Name']=='KRT19'].index
         prss1_ensg=islet_bulk_fetureData[islet_bulk_fetureData['Associated.Gene.Name']=='PRSS1'].index
```

```
In [19]: matplotlib.rcParams['figure.figsize'] = (10.0, 5.0)
         matplotlib.rcParams['axes.linewidth'] = 3
         matplotlib.rcParams['axes.edgecolor'] = 'k'
         matplotlib.rcParams['axes.spines.top']='False'
         matplotlib.rcParams['axes.spines.right']='False'
         matplotlib.rcParams['axes.facecolor']='white'

         fig,ax=plt.subplots(1,2)

         sns.kdeplot((nd_cpm.ix[ins_ensg]+1.).apply(np.log2),ax=ax[0],shade=True, color="#e41a1c",lw=3)
         ax[0].set_xlim(-4,25)
         ax[0].legend().set_visible(False)
         ax[0].set_title('INS',fontsize=14)
         ax[0].tick_params(axis='both', which='major', labelsize=14)
         ax[0].set_xlabel(r'$\log_2(CPM)$',fontsize=14)

         sns.kdeplot((nd_cpm.ix[gcg_ensg]+1.).apply(np.log2),ax=ax[1],shade=True, color="#377eb8",lw=3)
         ax[1].set_xlim(-4,25)
         ax[1].legend().set_visible(False)
         ax[1].set_title('GCG',fontsize=14)
```

```
ax[1].tick_params(axis='both', which='major', labelsize=14)
ax[1].set_xlabel(r'$\log_2(CPM)$',fontsize=14)

#nd_cpm.ix[gcg_ensg].plot(kind='kde',lw=3,color='#e41a1c',ax=ax[1])
```

Out[19]: <matplotlib.text.Text at 0x13882eed0>

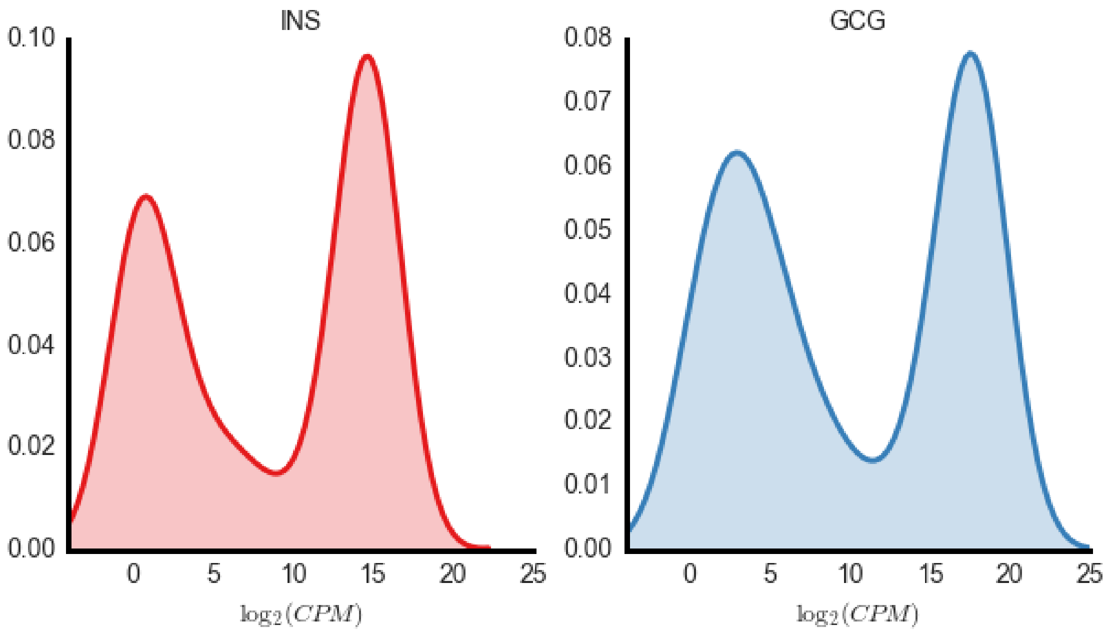

In [20]:
```
%%bash
mkdir Figures_06142016
```

In [21]:
```
fig.savefig('Figures_06142016/Figure2_gaussian_panelA.pdf',format='pdf',dpi=600)
```

In [47]:
```
fig.savefig('Figure2D.pdf',format='pdf',dpi=600)
```

In [22]:
```
matplotlib.rcParams['figure.figsize'] = (10.0, 5.0)
matplotlib.rcParams['axes.linewidth'] = 3
matplotlib.rcParams['axes.edgecolor'] = 'k'
matplotlib.rcParams['axes.spines.top']='False'
matplotlib.rcParams['axes.spines.right']='False'
matplotlib.rcParams['axes.facecolor']='white'

fig,ax=plt.subplots(1,2)

sns.kdeplot((nd_cpm.ix[sst_ensg]+1.).apply(np.log2),ax=ax[0],shade=True, color="#4daf4a",lw=3,l
ax[0].set_xlim(-4,25)
ax[0].legend().set_visible(False)
ax[0].set_title('SST',fontsize=14)
ax[0].tick_params(axis='both', which='major', labelsize=14)
ax[0].set_xlabel(r'$\log_2(CPM)$',fontsize=14)

sns.kdeplot((nd_cpm.ix[ppy_ensg]+1.).apply(np.log2),ax=ax[1],shade=True, color="#984ea3",lw=3,l
```

```
ax[1].set_xlim(-4,25)
ax[1].legend().set_visible(False)
ax[1].set_title('PPY',fontsize=14)
ax[1].tick_params(axis='both', which='major', labelsize=14)
ax[1].set_xlabel(r'$\log_2(CPM)$',fontsize=14)
```

Out[22]: <matplotlib.text.Text at 0x116e8ea90>

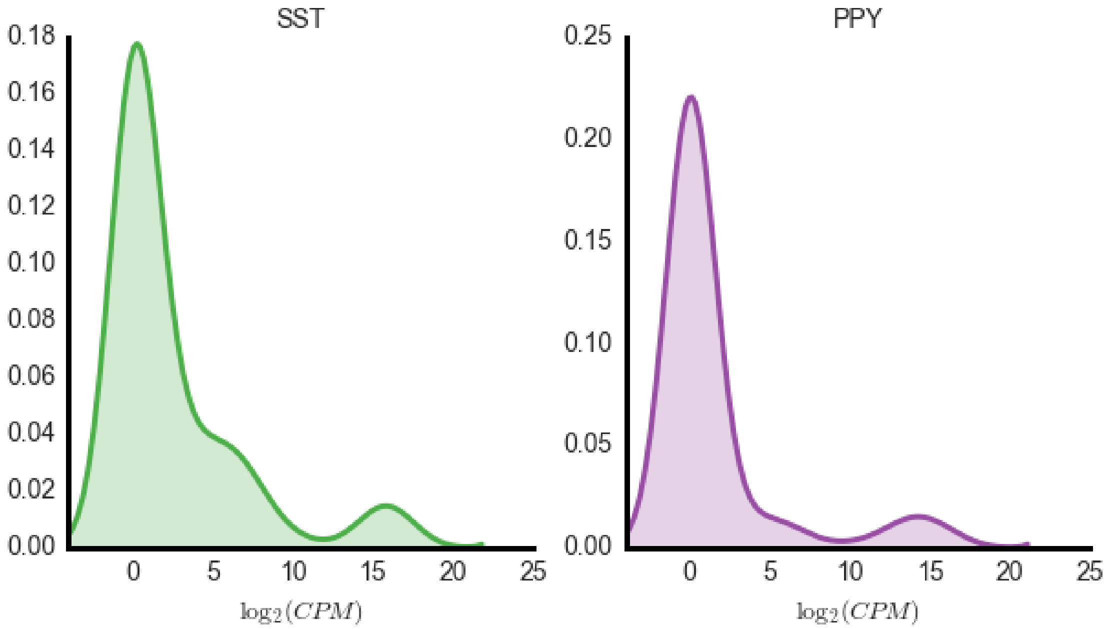

In [24]:
```
matplotlib.rcParams['figure.figsize'] = (10.0, 5.0)
matplotlib.rcParams['axes.linewidth'] = 3
matplotlib.rcParams['axes.edgecolor'] = 'k'
matplotlib.rcParams['axes.spines.top']='False'
matplotlib.rcParams['axes.spines.right']='False'
matplotlib.rcParams['axes.facecolor']='white'

fig,ax=plt.subplots(1,2)

sns.kdeplot((nd_cpm.ix[col1a1_ensg]+1.).apply(np.log2),ax=ax[0],shade=True, color="#000000",lw=
ax[0].set_xlim(-4,25)
ax[0].legend().set_visible(False)
ax[0].set_title('COL1A1',fontsize=14)
ax[0].tick_params(axis='both', which='major', labelsize=14)
ax[0].set_xlabel(r'$\log_2(CPM)$',fontsize=14)

sns.kdeplot((nd_cpm.ix[krt19_ensg]+1.).apply(np.log2),ax=ax[1],shade=True, color="#969696",lw=
ax[1].set_xlim(-4,25)
ax[1].legend().set_visible(False)
ax[1].set_title('KRT19',fontsize=14)
ax[1].tick_params(axis='both', which='major', labelsize=14)
ax[1].set_xlabel(r'$\log_2(CPM)$',fontsize=14)
```

```
Out[24]: <matplotlib.text.Text at 0x11a278c90>
```

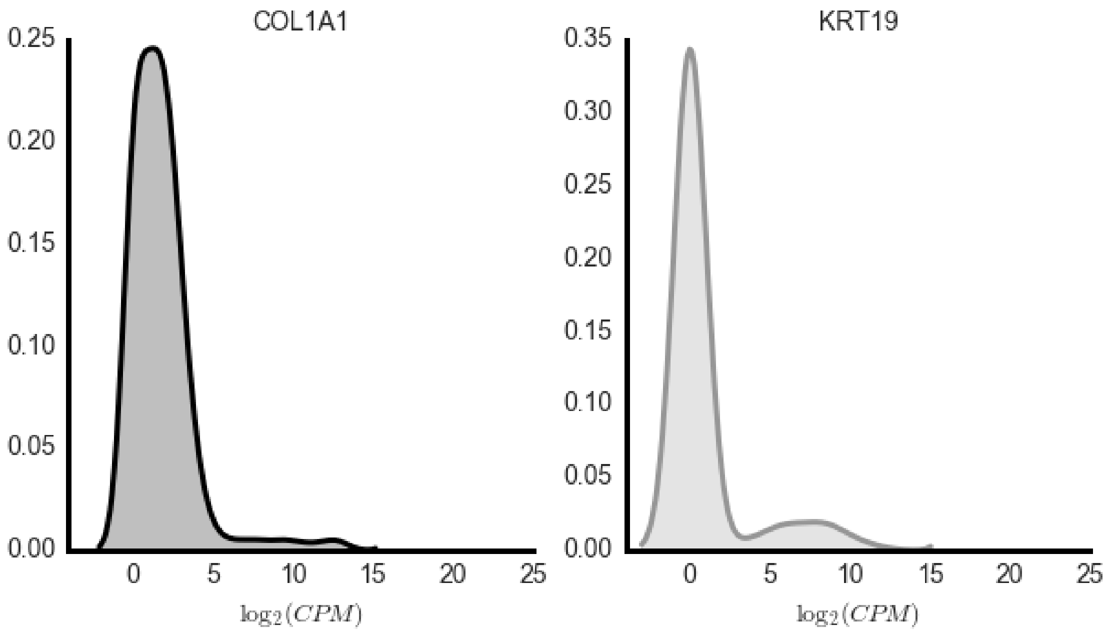


```
In [25]: fig.savefig('Figures_06142016/Figure2D_col1a1-krt19.pdf',format='pdf',dpi=600)
```

```
In [26]: matplotlib.rcParams['figure.figsize'] = (5.0, 5.0)
         matplotlib.rcParams['axes.linewidth'] = 3
         matplotlib.rcParams['axes.edgecolor'] = 'k'
         matplotlib.rcParams['axes.spines.top']='False'
         matplotlib.rcParams['axes.spines.right']='False'
         matplotlib.rcParams['axes.facecolor']='white'

         fig,ax=plt.subplots(1)

         sns.kdeplot((nd_cpm.ix[prss1_ensg]+1.).apply(np.log2),ax=ax,shade=True, color="#525252",lw=3,bw
         ax.set_xlim(-4,25)
         ax.legend().set_visible(False)
         ax.set_title('PRSS1',fontsize=14)
         ax.tick_params(axis='both', which='major', labelsize=14)
         ax.set_xlabel(r'$\log_2(CPM)$',fontsize=14)
```

```
Out[26]: <matplotlib.text.Text at 0x115258c90>
```

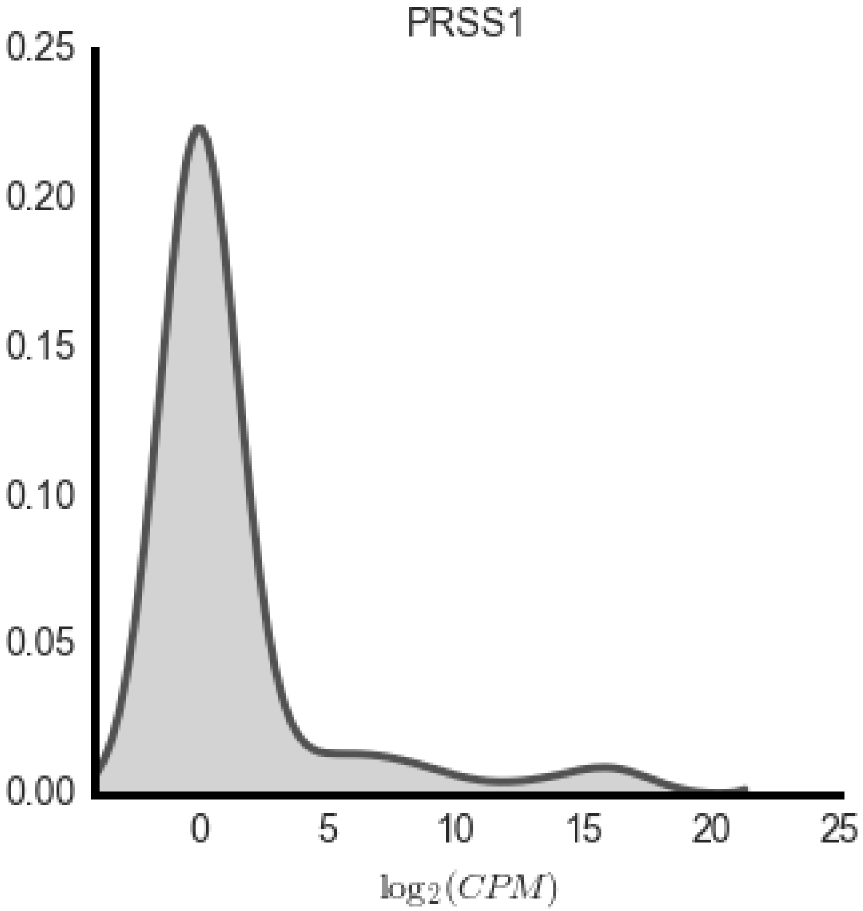


```
In [27]: fig.savefig('Figures_06142016/Figure2D_prss1.pdf',format='pdf',dpi=600)

In [ ]:
```
