# Supplementary material for: Single-cell transcriptomes identify human islet cell signatures and reveal cell-type–specific expression changes in type 2 diabetes
Source: Genome Res. 2017 Feb;27(2):208–22. doi: 10.1101/gr.212720.116 (PMC5287227; doi:10.1101/gr.212720.116)
Supplement: Supplemental Material [file supp_gr.212720.116_Supplemental_Methods_Source_Code.zip › Supplemental_Methods_Source_Code/Figure3_Source_Code/Figure_3A_Source_Code.pdf]

t-distributed Stochastic Neighbor Embedding (t-SNE) of Non-Diabetic Data

Introduction

This report describes the t-SNE (t-Distributed Stochastic Neighbor Embedding) method implemented to reduce the dimensionality of the single cell data. Similar to Principal Components Analysis (PCA), t-SNE is a dimensionality reduction method capable of embedding high dimensional data into two or three dimensions. This technique models each object such that those of high similarity are modeled by nearby points and those of low similarity are modeled by distant points. In particular, the R-package "Rtsne" was used to perform a Barnes-Hut variant of t-SNE. Barnes-Hut is an approximation algorithm that is used for grouping nearby objects using a quad-tree method, in which objects are divided into based on their distance from each other. Within each quadrant of this quad-tree, objects are further subdivided into quadrants until 0 or 1 objects remain in a single quadrant. The Barnes-Hut variant of t-SNE demonstrates faster computation time in comparison to standard t-SNE.

Reference: Maaten, Laurens Van Der. "Accelerating T-SNE Using Tree-Based Algorithms." Journal of Machine Learning Research (2014): 1-21.

Preprocessing Steps

All non-diabetic single cell samples were used except for the cells labeled as "multiples" yielding a total of 380 samples. Only highly expressed genes with a log2 counts per million (CPM) expression level greater than 10.5 in at least one sample were used to conduct the t-SNE analysis. Ultimately, 1824 genes were used in the t-SNE analysis. A two dimensional plot of the analysis is shown in the figure below. t-SNE analyses were also performed using only the Type 2 diabetic single cell data.

```r
# Load libraries
suppressPackageStartupMessages(library(Biobase))
suppressPackageStartupMessages(library(RColorBrewer))
suppressPackageStartupMessages(library(Rtsne))
suppressPackageStartupMessages(library(edgeR))
library(Biobase)
library(RColorBrewer)
library(Rtsne)
library(edgeR)
set.seed(125342)

# Load in Single Cell RNA-seq data
setwd("/Users/lawlon/Documents/Final_RNA_Seq_3/Data/")
load("nonT2D.rdata")

# Probe annotation data
p.anns <- as(featureData(cnts.eset), "data.frame")
# Sample annotation data
s.anns <- pData(cnts.eset)
# Remove multiples and keep all other groups
s.anns.sel <- s.anns[s.anns$cell.type %in% c("INS", "PPY", "GCG", "SST",
                              "COL1A1", "KRT19", "PRSS1", "none"),]

# Expression data
```

```
counts <- exprs(cnts.eset)

# Calculate cpm of data
cpm <- cpm(x = counts)
cpm.vals <- log2(cpm+1)
cpm.vals <- cpm.vals[,rownames(s.anns.sel)]

# Change name of one KRT19 cell to ghrelin cell
g <- which(p.anns$Associated.Gene.Name == "GHRL")
ghrl <- cpm.vals[g,]
samp <- which(ghrl > 15)
g.idx <- which(rownames(s.anns.sel) == names(samp))

# Change sample anns of cell to GHRL
s.anns.sel$cell.type[g.idx] <- "GHRL"

# Use genes with max value > 10.5 in at least one sample
r.max <- apply(cpm.vals,1,max)
cpm.sel <- cpm.vals[r.max > 10.5,]
cpm.sel <- cpm.sel[, rownames(s.anns.sel)]

# Transpose the matrix
cpm1 <- t(cpm.sel)

# Remove groups that are all zeros
df <- cpm1[,apply(cpm1, 2, var, na.rm=TRUE) != 0]

#Run tsne with defaults
rtsne_out <- Rtsne(as.matrix(df), dims = 2)

# Set rownames of matrix to tsne matrix
rownames(rtsne_out$Y) <- rownames(cpm1)

# Write tSNE matrix to file
#write.csv(rtsne_out$Y, file = paste(name, "tsne.matrix.data.2D.csv", sep="."))

# Color Schema
grey <- brewer.pal(n=9, name="Greys")

colorCodes <- c(INS="#e41a1c", GCG = "#377eb8", SST = "#4daf4a",
                PPY = "#984ea3", GHRL = "#ff7f00",
                COL1A1 = grey[9], PRSS1 = grey[7], KRT19 = grey[5],
                none = grey[3])

# Cell type name list
namelist <- c("Beta", "Alpha", "Delta", "Gamma", "Epsilon",
              "Stellate", "Acinar", "Ductal", "none")

# Shapes for 2D plot
type1 <- NULL
for (i in 1:length(s.anns.sel$cell.type)){
  # Endocrine cells labeled by circle
  if ((s.anns.sel$cell.type[i] %in% c("INS","GCG","SST","PPY", "GHRL")) == TRUE) {
```

```r
    idx = 20
    type1 = c(type1, idx)
  } else {
    # Exocrine cells labeled by triangle
    idx = 17
    type1 = c(type1, idx)
  }
}

# Match up cell type name with hormone type
cellnames = NULL
for (i in 1:length(s.anns.sel$cell.type)) {
  if (s.anns.sel$cell.type[i] %in% names(namelist) == TRUE) {
    cellnames = c(cellnames, as.character(namelist[s.anns.sel$cell.type[i]]))
  }
}

# Match up colors and hormone labels
cols = NULL
for (i in 1:length(s.anns.sel$cell.type)) {
  if ((s.anns.sel$cell.type[i] %in% names(colorCodes)) == TRUE) {
    cols <- c(cols, colorCodes[s.anns.sel$cell.type[i]])
  }
}

# Match up cell name with hormone name
# Have cell type name and color
for (i in 1:length(cols)) {
  if (names(cols)[i] %in% names(namelist) == TRUE) {
    names(cols)[i] <- namelist[names(cols)[i]]
  }
}
```

```r
# Plot the t-sne in 2-D
plot(rtsne_out$Y[,1], rtsne_out$Y[,2], col = cols, pch = type1,
     xlab = "t-SNE 1", ylab = "t-SNE 2")
legend("bottomright", legend = as.character(namelist),
  text.col = colorCodes, pch = c(20,20,20,20,20,17,17,17,17), col = colorCodes,
  cex = 1)
```

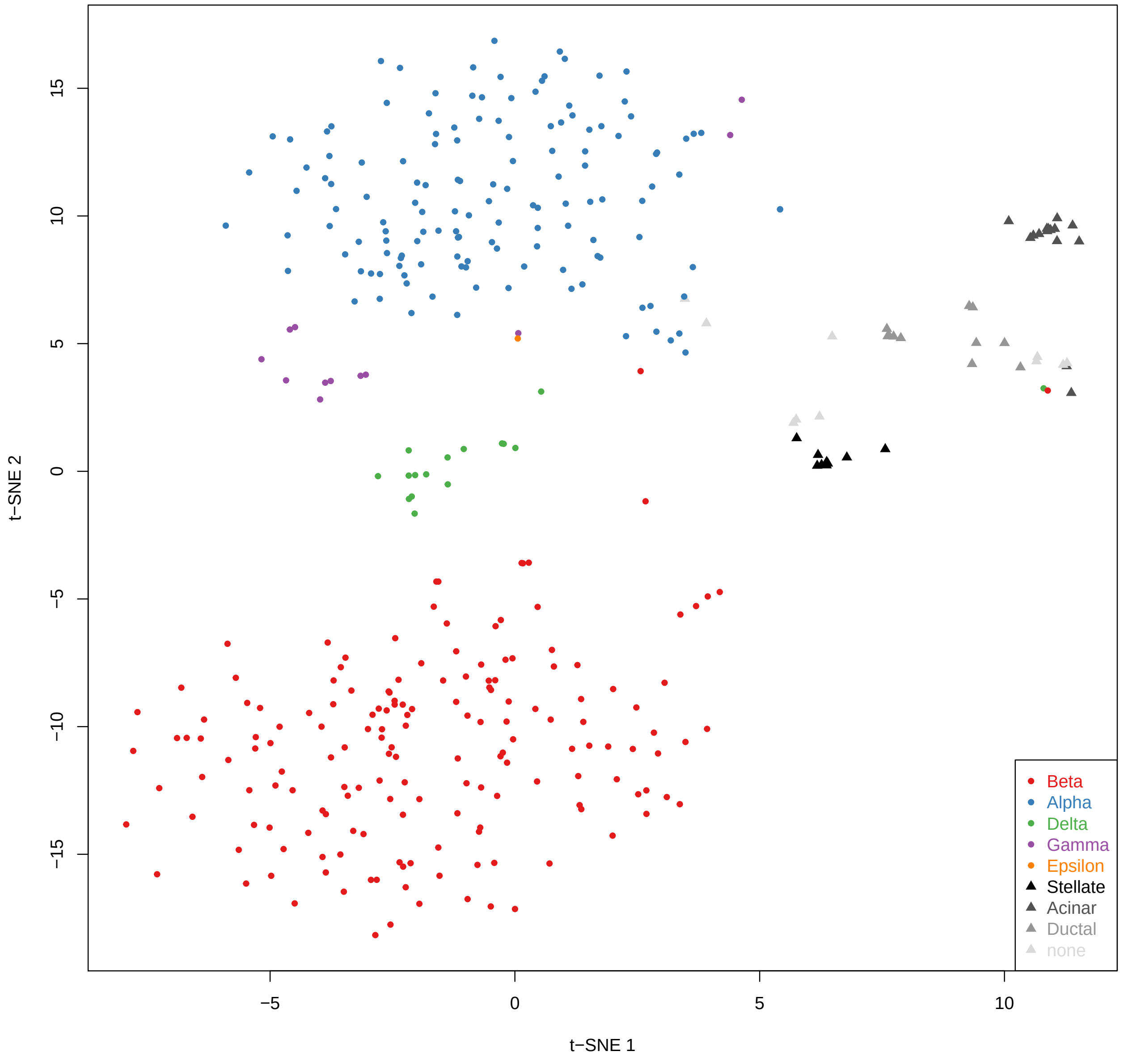

Figure 1: t-SNE analysis of non-diabetic and diabetic single cell data using genes with log2(CPM) values greater than 10.5 in at least one sample.

Session Information

```
suppressPackageStartupMessages(library(Biobase))
suppressPackageStartupMessages(library(RColorBrewer))
suppressPackageStartupMessages(library(Rtsne))
suppressPackageStartupMessages(library(edgeR))
library(Biobase)
library(RColorBrewer)
library(Rtsne)
library(edgeR)
sessionInfo()
```

```
## R version 3.3.0 (2016-05-03)
## Platform: x86_64-apple-darwin13.4.0 (64-bit)
## Running under: OS X 10.11.3 (El Capitan)
##
## locale:
## [1] en_US.UTF-8/en_US.UTF-8/en_US.UTF-8/C/en_US.UTF-8/en_US.UTF-8
##
## attached base packages:
## [1] parallel  stats     graphics  grDevices utils     datasets  methods
## [8] base
##
## other attached packages:
## [1] edgeR_3.14.0        limma_3.28.7        Rtsne_0.10
## [4] RColorBrewer_1.1-2  Biobase_2.32.0      BiocGenerics_0.18.0
##
## loaded via a namespace (and not attached):
##  [1] Rcpp_0.12.5     digest_0.6.9    formatR_1.4     magrittr_1.5
##  [5] evaluate_0.9    stringi_1.1.1   rmarkdown_0.9.6 tools_3.3.0
##  [9] stringr_1.0.0   yaml_2.1.13     htmltools_0.3.5 knitr_1.13
```
