# Supplementary material for: Single-cell transcriptomes identify human islet cell signatures and reveal cell-type–specific expression changes in type 2 diabetes
Source: Genome Res. 2017 Feb;27(2):208–22. doi: 10.1101/gr.212720.116 (PMC5287227; doi:10.1101/gr.212720.116)
Supplement: Supplemental Material [file supp_gr.212720.116_Supplemental_Methods_Source_Code.zip › Supplemental_Methods_Source_Code/Figure3_Source_Code/Figure_3B_Source_Code.pdf]

Unsupervised Hierarchical Clustering of Non-Diabetic Single Cell Data

Introduction

This report describes the methods used to perform unsupervised hierarchical clustering of the non-diabetic single cell data. The samples were clustered using highly expressed genes with log2(CPM) values greater than 10.5 in at least one sample. The clustering was performed using the “hclust” function using Euclidean distance and Ward.D2 linkage. The resultant “fan” dendrogram image was produced using the “ape” and “dendextend” R-packages.

Reference: Paradis E., Claude J. & Strimmer K. 2004. APE: analyses of phylogenetics and evolution in R language. Bioinformatics 20: 289-290.

Preprocessing Steps

All single cell samples from our non-diabetic cohort were used except for the cells labeled as “multiples” yielding a total of 380 samples. Only highly expressed genes with a log2 CPM expression level greater than 10.5 in at least one sample were used to perform the clustering. Ultimately, 1824 genes were used in the analysis. A two dimensional plot of the resultant dendrogram is shown in the figure below. Similar unsupervised hierarchical clustering analyses were performed on the non-diabetic and Type 2 diabetic single cell data combined and Type 2 diabetic data alone.

```
# Load libraries
suppressPackageStartupMessages(library(Biobase))
suppressPackageStartupMessages(library(gplots))
suppressPackageStartupMessages(library(dendextend))
suppressPackageStartupMessages(library(ape))
suppressPackageStartupMessages(library(knitr))
suppressPackageStartupMessages(library(RColorBrewer))
suppressPackageStartupMessages(library(edgeR))
library(Biobase)
library(gplots)
library(dendextend)
library(ape)
library(knitr)
library(RColorBrewer)
library(edgeR)
set.seed(53079239)

# Load NonT2D single cell data
setwd("/Users/lawlon/Documents/Final_RNA_Seq_3/Data/")
load("nonT2D.rdata")
p.anns <- featureData(cnts.eset)
probe.anns <- as(p.anns,"data.frame")
```

```r
ND.anns <- pData(cnts.eset)
# Remove multiples and keep all other groups
ND.sel <- ND.anns[ND.anns$cell.type %in% c("INS", "PPY", "GCG", "SST", "COL1A1", "KRT
19", "PRSS1", "none"),]

# Obtain counts
ND.counts <- exprs(cnts.eset)
# Calculate log2 cpm
cpms <- cpm(x = ND.counts)
data <- log2(cpms+1)
data <- data[, rownames(ND.sel)]

# Filter gene by those with max CPM value greater than 10 (high expressed genes)
r.max <- apply(data,1,max)
ND.data.sel <- data[r.max > 10.5,]

# Change column name labels to cell type
colnames(ND.data.sel)[1:dim(ND.sel)[1]] <- ND.sel$cell.type

# Change name of one KRT19 cell (which is ghrelin positive) to ghrelin cell label
g <- which(probe.anns$Associated.Gene.Name == "GHRL")
ghrl <- data[g,]
samp <- which(ghrl > 15)
g.idx <- which(rownames(ND.sel) == names(samp))
colnames(ND.data.sel)[g.idx] <- "GHRL"

# Combine probe anns with selected fpkm values
p.res <- probe.anns[rownames(ND.data.sel),]
ND.data.sel.exp <- cbind(p.res,ND.data.sel)

# Write selected gene matrix to file
#write.csv(ND.data.sel.exp, paste(fname, "genes_selected_for_cing.csv", sep = "."))

# Create an hclust object
d <- dist(t(ND.data.sel))
hc.final <- hclust(d,method="ward.D2")

# Change hclust object to dendrogram
dend1 <- as.dendrogram(hc.final)
groupCodes <- ND.sel$cell.type

# Color Schema
grey <- brewer.pal(n=9, name="Greys")

# Specify a color for each cell type
colorCodes <- c(INS="#e41a1c", GCG = "#377eb8", SST = "#4daf4a", PPY = "#984ea3", GHR
L = "#ff7f00",
                COL1A1 = grey[9], PRSS1 = grey[7], KRT19 = grey[5],
                none = grey[3])
```

```
# Names of the cell types corresponding to hormone marker genes
namelist <- c("Beta", "Alpha", "Delta", "Gamma", "Epsilon", "Stellate", "Acinar", "Du
ctal", "none")

# Assign color labels to the dendrogram
labels_colors(dend1) <- colorCodes[groupCodes][order.dendrogram(dend1)]

# Change dendrogram object to phylo object to make fan dendrogram later
dend2 <- as.phylo(dend1)

# Match up colors and labels
cols = NULL
for (i in 1:length(labels(dend2))) {
  if ((dend2$tip.label[i] %in% names(colorCodes)) == TRUE) {
    cols <- c(cols, colorCodes[dend2$tip.label[i]])
  }
}

# Change labels of dendrogram to hyphen symbol for visualization
labels(dend2) <- rep(x = "—", length(labels(dend2)))

# Set margins
par(mar = c(0,0,0,0))

plot(dend2, type = "fan", tip.color = cols, cex = 8.0, label.offset = 0)
legend("bottomleft", title = "Cell Types", title.col = "black",
       legend = c(expression(bold("Beta (INS)")), expression(bold("Alpha (GCG)")),
       expression(bold("Delta (SST)")), expression(bold("Gamma (PPY)")),  expression(
bold("Epsilon (GHRL)")),
       expression(bold("Stellate (COL1A1)")), expression(bold("Acinar (PRSS1)")),
       expression(bold("Ductal (KRT19)")), expression(bold("None"))), text.col = colo
rCodes,
       cex = 0.75, xjust=0, yjust=0)

# Note that increasing the cex value when plotting can make the color bars appear mor
e blended
# In addition, producing a high resolution tiff image as output is recommended:
  # tiff(file=paste(fname, "dendrogram.tiff", sep = "."),
  #  width = 9000, height = 9000, units = "px", res = 800)
```

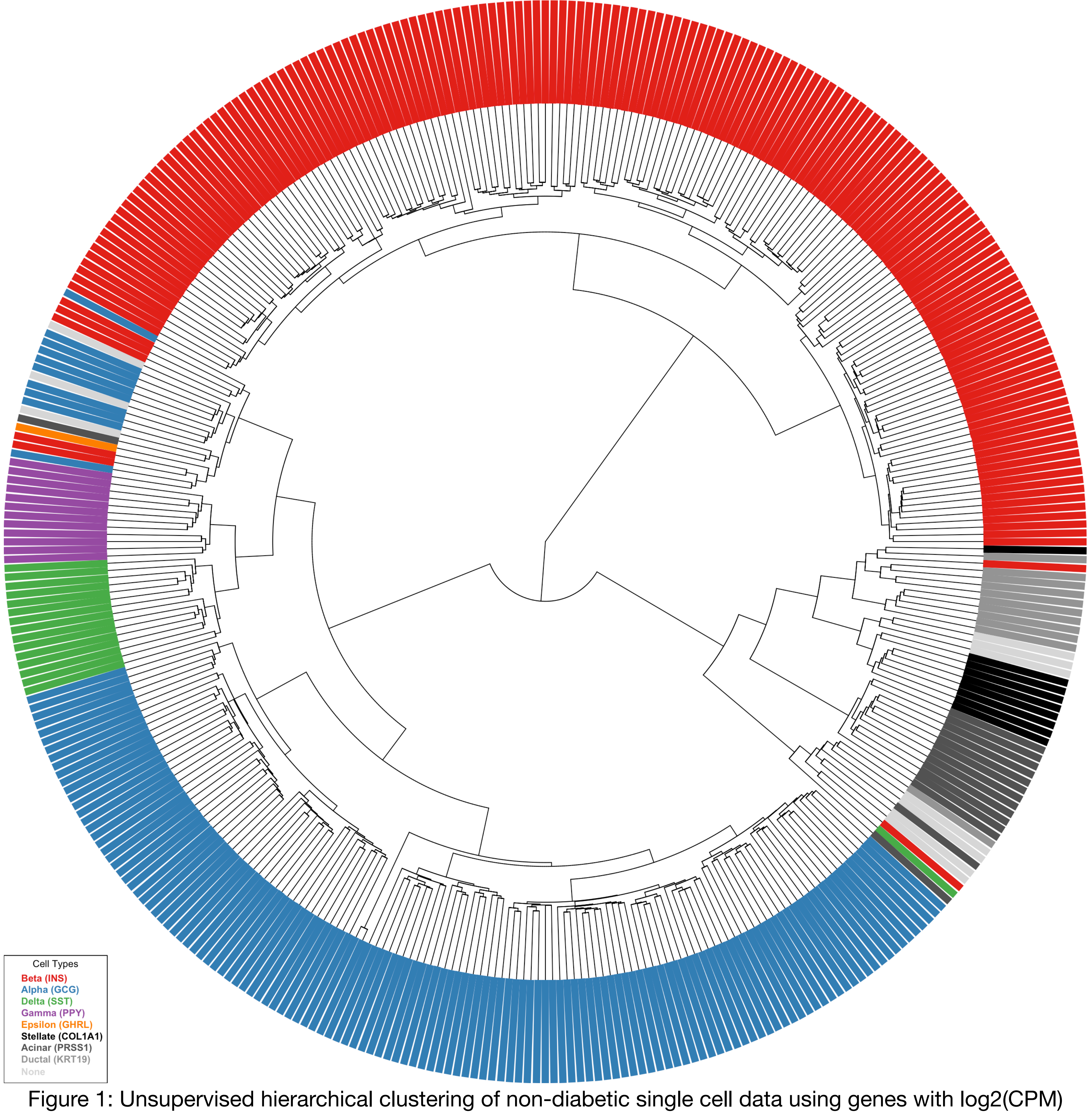

Figure 1: Unsupervised hierarchical clustering of non-diabetic single cell data using genes with log2(CPM) values greater than 10.5 in at least one sample.

Session Information

```
suppressPackageStartupMessages(library(Biobase))
suppressPackageStartupMessages(library(gplots))
suppressPackageStartupMessages(library(dendextend))
suppressPackageStartupMessages(library(ape))
suppressPackageStartupMessages(library(knitr))
suppressPackageStartupMessages(library(RColorBrewer))
suppressPackageStartupMessages(library(edgeR))
library(Biobase)
library(gplots)
library(dendextend)
library(ape)
library(knitr)
library(RColorBrewer)
library(edgeR)
sessionInfo()
```

```
## R version 3.3.0 (2016-05-03)
## Platform: x86_64-apple-darwin13.4.0 (64-bit)
## Running under: OS X 10.11.3 (El Capitan)
##
## locale:
## [1] en_US.UTF-8/en_US.UTF-8/en_US.UTF-8/C/en_US.UTF-8/en_US.UTF-8
##
## attached base packages:
## [1] parallel  stats     graphics  grDevices utils     datasets  methods
## [8] base
##
## other attached packages:
## [1] edgeR_3.14.0        limma_3.28.7        RColorBrewer_1.1-2
## [4] knitr_1.13          ape_3.5             dendextend_1.1.8
## [7] gplots_3.0.1        Biobase_2.32.0      BiocGenerics_0.18.0
##
## loaded via a namespace (and not attached):
##  [1] Rcpp_0.12.5        whisker_0.3-2      magrittr_1.5
##  [4] lattice_0.20-33    stringr_1.0.0      caTools_1.17.1
##  [7] tools_3.3.0        grid_3.3.0         nlme_3.1-128
## [10] KernSmooth_2.23-15 htmltools_0.3.5    gtools_3.5.0
## [13] yaml_2.1.13        digest_0.6.9       formatR_1.4
## [16] bitops_1.0-6       evaluate_0.9       rmarkdown_0.9.6
## [19] gdata_2.17.0       stringi_1.1.1
```
