# Supplementary material for: Single-cell transcriptomes identify human islet cell signatures and reveal cell-type–specific expression changes in type 2 diabetes
Source: Genome Res. 2017 Feb;27(2):208–22. doi: 10.1101/gr.212720.116 (PMC5287227; doi:10.1101/gr.212720.116)
Supplement: Supplemental Material [file supp_gr.212720.116_Supplemental_Methods_Source_Code.zip › Supplemental_Methods_Source_Code/Figure3_Source_Code/Figure_3C_Source_Code.pdf]

# Heat map visualization of signature genes in non-diabetic single cells

## Introduction

This report describes the steps used to produce a heat map of log2(CPM) expression of all identified signature genes across all non-diabetic single cell samples. All non-diabetic endocrine and exocrine single cell samples were included in the heat map excluding "none" and "multiple" classified samples. The resulting heat map shows the log2(CPM) expression of genes after mean centering and scalining the values between -1 and 1.

```
# Load libraries
suppressPackageStartupMessages(library(Biobase))
suppressPackageStartupMessages(library(pheatmap))
suppressPackageStartupMessages(library(RColorBrewer))
suppressPackageStartupMessages(library(edgeR))
library(Biobase)
library(edgeR)
library(pheatmap)
library(RColorBrewer)

setwd("/Users/lawlon/Documents/Final_RNA_Seq_3/Data/")
# Load in nonT2D single cell data
load("nonT2D.rdata")
# Load sample annotations and probe annotations
s.anns <- pData(cnts.eset)
p.anns <- as(featureData(cnts.eset), "data.frame")
# Obtain count data for nonT2D cells
counts <- exprs(cnts.eset)
# Calculate the cpm of the data
cpms <- cpm(x = counts)
# Log2 transform the data
data <- log2(cpms+1)

# Separate cell types into groups
s.1 <- s.anns[s.anns$cell.type %in% c("INS"),]
s.2 <- s.anns[s.anns$cell.type %in% c("GCG"),]
s.3 <- s.anns[s.anns$cell.type %in% c("SST"),]
s.4 <- s.anns[s.anns$cell.type %in% c("PPY"),]
s.5 <- s.anns[s.anns$cell.type %in% c("PRSS1"),]
s.6 <- s.anns[s.anns$cell.type %in% c("KRT19"),]
s.7 <- s.anns[s.anns$cell.type %in% c("COL1A1"),]

# Get Expression matrices for each cell type
f.1 <- data[, rownames(s.1)]
f.2 <- data[, rownames(s.2)]
f.3 <- data[, rownames(s.3)]
f.4 <- data[, rownames(s.4)]
f.5 <- data[, rownames(s.5)]
f.6 <- data[, rownames(s.6)]
f.7 <- data[, rownames(s.7)]
```

```r
# Create a matrix containing all log2 CPM expression of each gene across cell types
  # with cell types ordered in groups
mat.orig <- cbind(f.1, f.2, f.3, f.4, f.5, f.6, f.7)

# Change rownames of matrix to gene ensembl ids
rownames(mat.orig) <- rownames(p.anns)

# Read in list of signature genes and the cell type they correspond to
setwd("/Users/lawlon/Documents/Final_RNA_Seq_3/Signature_Genes/")
genes <- read.csv(file = "NonT2D.Endo.and.Exo.Signature.genes.csv", header = FALSE,
                  check.names = FALSE, row.names = NULL, stringsAsFactors = FALSE)

# Remove any duplicate genes
dups <- duplicated(genes[,1])
genes <- genes[!dups,]

# Extract genes of interest from original expression matrix
mat.sel <- mat.orig[genes[,1],]

# Extract genes of interest for each individual cell group
f.1.sel <- f.1[genes[,1],]
f.2.sel <- f.2[genes[,1],]
f.3.sel <- f.3[genes[,1],]
f.4.sel <- f.4[genes[,1],]
f.5.sel <- f.5[genes[,1],]
f.6.sel <- f.6[genes[,1],]
f.7.sel <- f.7[genes[,1],]

# Sort genes in each Beta, Alpha, etc cell type by log2 CPM
beta.id <- which(genes[,2] == "Beta")
# If there are beta signature genes continue with code
if (length(beta.id) >1 ) {
  # Calculate mean expression of beta signature genes in beta cells
  beta.rm <- rowMeans(f.1.sel[beta.id,])
  # Sort the genes from high to low average log2 CPM
  beta.sort <- sort(beta.rm, decreasing = TRUE)
  # Obtain names of sorted genes
  beta.nam <- names(beta.sort)
} else {
  beta.nam <- rownames(f.1.sel)[beta.id]
}
# Make vector of beta names
beta.vec <- rep("Beta", length(beta.nam))

# Repeat the process for all other cell types
alp.id <- which(genes[,2] == "Alpha")
if (length(alp.id) > 1) {
  alp.rm <- rowMeans(f.2.sel[alp.id,])
  alp.sort <- sort(alp.rm, decreasing = TRUE)
  alp.nam <- names(alp.sort)
} else {
  alp.nam <- rownames(f.2.sel)[alp.id]
}
```

```r
# Make vector of alpha names
alp.vec <- rep("Alpha", length(alp.nam))

del.id <- which(genes[,2] == "Delta")
if (length(del.id) > 1) {
  del.rm <- rowMeans(f.3.sel[del.id,])
  del.sort <- sort(del.rm, decreasing = TRUE)
  del.nam <- names(del.sort)
} else {
  del.nam <- rownames(f.3.sel)[del.id]
}

# Make vector of delta names
del.vec <- rep("Delta", length(del.nam))

gam.id <- which(genes[,2] == "Gamma")
if (length(gam.id) > 1) {
  gam.rm <- rowMeans(f.4.sel[gam.id,])
  gam.sort <- sort(gam.rm, decreasing = TRUE)
  gam.nam <- names(gam.sort)
} else {
  gam.nam <- rownames(f.4.sel)[gam.id]
}

# Make vector of gamma names
gam.vec <- rep("Gamma", length(gam.nam))

ac.id <- which(genes[,2] == "Acinar")
if (length(ac.id) > 1) {
   ac.rm <- rowMeans(f.5.sel[ac.id,])
   ac.sort <- sort(ac.rm, decreasing = TRUE)
   ac.nam <- names(ac.sort)
   } else {
   ac.nam <- rownames(f.5.sel)[ac.id]
}

# Make vector of acinar names
ac.vec <- rep("Acinar", length(ac.nam))

duc.id <- which(genes[,2] == "Ductal")
if (length(duc.id) >1) {
   duc.rm <- rowMeans(f.6.sel[duc.id,])
   duc.sort <- sort(duc.rm, decreasing = TRUE)
   duc.nam <- names(duc.sort)
   } else {
   duc.nam <- rownames(f.6.sel)[duc.id]
}

# Make vector of ductal names
duc.vec <- rep("Ductal", length(duc.nam))

stel.id <- which(genes[,2] == "Stellate")
if (length(stel.id) >1) {
```

```
   stel.rm <- rowMeans(f.7.sel[stel.id,])
   stel.sort <- sort(stel.rm, decreasing = TRUE)
   stel.nam <- names(stel.sort)
   } else {
   stel.nam <- rownames(f.7.sel)[stel.id]
}

# Make a vector of stellate names
stel.vec <- rep("Stellate", length(stel.nam))

# Obtain order of log2 CPM ranked genes
sort.genes <- c(beta.nam, alp.nam, del.nam, gam.nam, ac.nam, duc.nam, stel.nam)
# Obtain full list of cell types in matrix
sort.names <- c(beta.vec, alp.vec, del.vec, gam.vec, ac.vec, duc.vec, stel.vec)
# Re-order the expression matrix by genes ranked by log2 CPM
mat.orig.sort <- mat.orig[sort.genes,]

# Mean center the data by row
center_apply <- function(x) {
  apply(x, 1, function(y) y - mean(y))
}

mat.center <- center_apply(mat.orig.sort)
mat.center <- t(mat.center)

# Scale the data between -1 and 1
nor.min.max <- function(x) {
  if (is.numeric(x) == FALSE) {
    stop("Please input numeric for x")
  }
  x.min <- min(x)
  x.max <- max(x)
  x <- 2*((x - x.min) / (x.max - x.min)) - 1
  return (x)
}
mat.scale <- t(apply(mat.center, 1, nor.min.max))

# Change row names of expression matrix to gene symbol
symbols <- NULL
for (i in 1:dim(mat.scale)[1]) {
  idx <- which(rownames(p.anns) == rownames(mat.scale)[i])
  symbols <- c(symbols, idx)
}

# Set gene symbols as the names of the annotation matrix
gen.sym <- p.anns$Associated.Gene.Name[symbols]
rownames(mat.scale) <- gen.sym
rownames(mat.orig.sort) <- gen.sym

# Annotation matrix for cell types
annotation_col = data.frame(Cell_Type = c(rep("Beta", dim(f.1)[2]), rep("Alpha",
        dim(f.2)[2]), rep("Delta", dim(f.3)[2]), rep("Gamma", dim(f.4)[2]),
        rep("Acinar", dim(f.5)[2]), rep("Ductal", dim(f.6)[2]),
```

```
        rep("Stellate", dim(f.7)[2])))
rownames(annotation_col) <- colnames(mat.orig)

# Annotation rows by signature gene type
annotation_row = data.frame(Gene_Type = sort.names)
rownames(annotation_row) <- gen.sym

# Specify cell type colors
grey <- brewer.pal(n=9, name="Greys")
ann_colors <- list(
  Cell_Type = c(Beta="#e41a1c", Alpha = "#377eb8", Delta = "#4daf4a", Gamma = "#984ea3",
                Acinar = grey[7], Ductal = grey[5],Stellate = grey[9]),
  Gene_Type = c(Beta="#e41a1c", Alpha = "#377eb8", Delta = "#4daf4a", Gamma = "#984ea3",
                Acinar = grey[7], Ductal = grey[5], Stellate = grey[9]))
```

```
# Make heatmap
pheatmap(mat = mat.scale, annotation_col = annotation_col, annotation_row = annotation_row,
         cluster_rows = FALSE, cluster_cols = FALSE,
         color =  colorRampPalette(rev(brewer.pal(n = 7, name ="RdBu")))(20),
         annotation_colors = ann_colors, clustering_distance_rows = NULL,
         clustering_method=NULL,show_rownames=FALSE,
         show_colnames = FALSE, annotation_names_row = TRUE,
         annotation_names_col = TRUE, trace = "none", fontsize_row = 6)
```

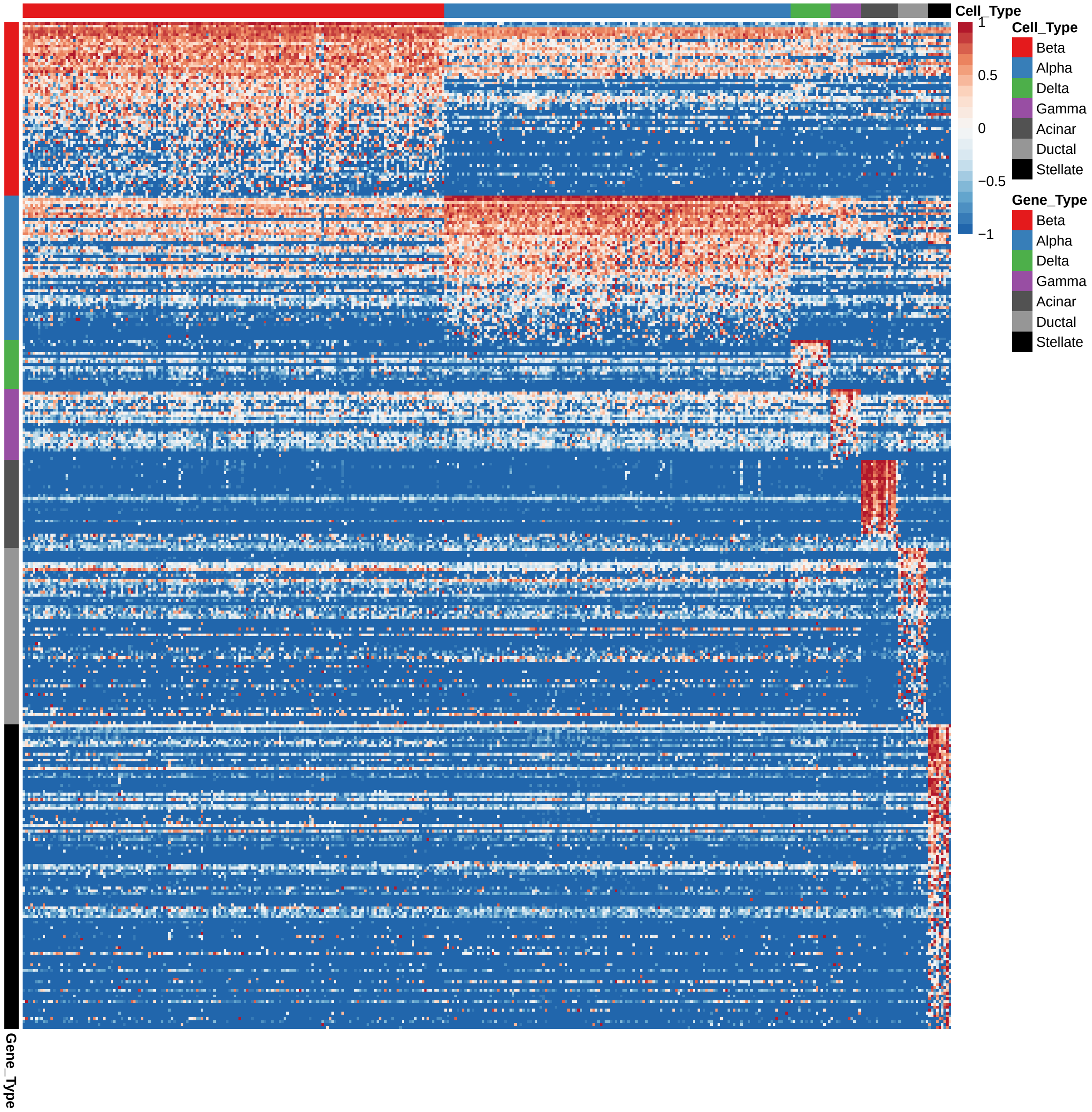


Figure 1: Heat map of log2(CPM) expression of all identified signature genes across non-diabetic single cell samples.

## Session Information

```
# Load libraries
suppressPackageStartupMessages(library(Biobase))
suppressPackageStartupMessages(library(pheatmap))
suppressPackageStartupMessages(library(RColorBrewer))
suppressPackageStartupMessages(library(edgeR))
library(Biobase)
library(edgeR)
library(pheatmap)
library(RColorBrewer)
sessionInfo()
```

```
## R version 3.3.0 (2016-05-03)
## Platform: x86_64-apple-darwin13.4.0 (64-bit)
## Running under: OS X 10.11.3 (El Capitan)
##
## locale:
## [1] en_US.UTF-8/en_US.UTF-8/en_US.UTF-8/C/en_US.UTF-8/en_US.UTF-8
##
## attached base packages:
## [1] parallel  stats     graphics  grDevices utils     datasets  methods
## [8] base
##
## other attached packages:
## [1] edgeR_3.14.0        limma_3.28.7        RColorBrewer_1.1-2
## [4] pheatmap_1.0.8      Biobase_2.32.0      BiocGenerics_0.18.0
##
## loaded via a namespace (and not attached):
##  [1] Rcpp_0.12.5      digest_0.6.9     plyr_1.8.4       grid_3.3.0
##  [5] gtable_0.2.0     formatR_1.4      magrittr_1.5     scales_0.4.0
##  [9] evaluate_0.9     stringi_1.1.1    rmarkdown_0.9.6  tools_3.3.0
## [13] stringr_1.0.0    munsell_0.4.3    yaml_2.1.13      colorspace_1.2-6
## [17] htmltools_0.3.5  knitr_1.13
```
