# Supplementary material for: Single-cell transcriptomes identify human islet cell signatures and reveal cell-type–specific expression changes in type 2 diabetes
Source: Genome Res. 2017 Feb;27(2):208–22. doi: 10.1101/gr.212720.116 (PMC5287227; doi:10.1101/gr.212720.116)
Supplement: Supplemental Material [file supp_gr.212720.116_Supplemental_Methods_Source_Code.zip › Supplemental_Methods_Source_Code/Figure3_Source_Code/Figure_3C_Violin_Plots_Source_Code.pdf]

Violin Plots of Signature Genes in Non-diabetic Single Cells

Introduction

This report describes the steps used to produce violin plots of log2(CPM) expression of all identified signature genes across all non-diabetic single cell samples.

```
rm(list=ls())
# Load  in libraries
suppressPackageStartupMessages(library(Biobase))
suppressPackageStartupMessages(library(RColorBrewer))
suppressPackageStartupMessages(library(edgeR))
suppressPackageStartupMessages(library(sm))
library(edgeR)
library(Biobase)
library(RColorBrewer)
library(sm)
setwd("/Users/lawlon/Documents/Final_RNA_Seq_3/Data/")
# Load in counts, sample anns, probe anns
load("nonT2D.rdata")
s.anns <- pData(cnts.eset)
p.anns <- as(featureData(cnts.eset), "data.frame")
cnts <- exprs(cnts.eset)

# Calculate the cpm of the data
cpms <- cpm(x = cnts)
data <- log2(cpms+1)

## LOAD SELECTED GENE LIST ##
setwd("/Users/lawlon/Documents/Final_RNA_Seq_3/Signature_Genes/")
genes <- read.csv(file="NonT2D.Endo.and.Exo.Signature.genes.csv", header=F,
                  check.names = F, row.names = NULL)
genes <- genes[,1]

name = "NonT2D.All.Signature.Genes"

# Hormone marker genes for each cell type
grp1 <- "INS"
grp2 <- "GCG"
grp3 <- "SST"
grp4 <- "PPY"
grp5 <- "COL1A1"
grp6 <- "KRT19"
grp7 <- "PRSS1"

# Cell type name corresponding to the hormone marker gene
name1 <- "Beta"
name2 <- "Alpha"
name3 <- "Delta"
name4 <- "Gamma"
```

```
name5 <- "Stellate"
name6 <- "Ductal"
name7 <- "Acinar"

# Find which samples are labeled as which
  # INS labeled
ins <- which(s.anns$cell.type == grp1)
cpm.ins <- data[, ins]
# Check for zeros
y1 <- t(cpm.ins)
for (i in 1:dim(y1)[2]){
  if (max(y1[,i]) == 0) {
    y1[1,i] <- 0.00001
  }
}

  # GCG labeled
gcg <- which(s.anns$cell.type == grp2)
cpm.gcg <- data[, gcg]
# Check for zeros
y2 <- t(cpm.gcg)
for (i in 1:dim(y2)[2]){
  if (max(y2[,i]) == 0) {
    y2[1,i] <- 0.00001
  }
}

  # SST labeled
sst <- which(s.anns$cell.type == grp3)
cpm.sst <- data[, sst]
# Check for zeros
y3 <- t(cpm.sst)
for (i in 1:dim(y3)[2]){
  if (max(y3[,i]) == 0) {
    y3[1,i] <- 0.00001
  }
}

  # PPY labeled
ppy <- which(s.anns$cell.type == grp4)
cpm.ppy <- data[, ppy]
# Check for zeros
y4 <- t(cpm.ppy)
for (i in 1:dim(y4)[2]){
  if (max(y4[,i]) == 0) {
    y4[1,i] <- 0.00001
  }
}

  # COL1A1 labeled
col1a1 <- which(s.anns$cell.type == grp5)
cpm.col1a1 <- data[, col1a1]
# Check for zeros
```

```r
y5 <- t(cpm.col1a1)
for (i in 1:dim(y5)[2]){
  if (max(y5[,i]) == 0) {
    y5[1,i] <- 0.00001
  }
}

# KRT19 labeled
krt19 <- which(s.anns$cell.type == grp6)
cpm.krt19 <- data[, krt19]
# Check for zeros
y6 <- t(cpm.krt19)
for (i in 1:dim(y6)[2]){
  if (max(y6[,i]) == 0) {
    y6[1,i] <- 0.00001
  }
}

# PRSS1 labeled
prss1 <- which(s.anns$cell.type == grp7)
cpm.prss1 <- data[, prss1]
# Check for zeros
y7 <- t(cpm.prss1)
for (i in 1:dim(y7)[2]){
  if (max(y7[,i]) == 0) {
    y7[1,i] <- 0.00001
  }
}

# Set color panel
grey <- brewer.pal(n=9, name="Greys")

## ALTERED VIOPLOT SOURCE CODE
vioplot <- function(x,...,range=1.5,h=NULL,ylim=NULL,names=NULL, horizontal=FALSE,
                    col="magenta", border="black", lty=1, lwd=1, rectCol="black",
                    colMed="white", pchMed=19, at, add=FALSE, wex=1,
                    drawRect=TRUE)
{
  # process multiple datas
  datas <- list(x,...)
  n <- length(datas)

  if(missing(at)) at <- 1:n

  # pass 1
  #
  # - calculate base range
  # - estimate density
  #

  # setup parameters for density estimation
  upper  <- vector(mode="numeric",length=n)
  lower  <- vector(mode="numeric",length=n)
```

```r
q1     <- vector(mode="numeric",length=n)
q3     <- vector(mode="numeric",length=n)
med    <- vector(mode="numeric",length=n)
base   <- vector(mode="list",length=n)
height <- vector(mode="list",length=n)
baserange <- c(Inf,-Inf)

# global args for sm.density function-call
args <- list(display="none")

if (!(is.null(h)))
  args <- c(args, h=h)

for(i in 1:n) {
  data<-datas[[i]]

  # calculate plot parameters
  #   1- and 3-quantile, median, IQR, upper- and lower-adjacent
  data.min <- min(data)
  data.max <- max(data)
  q1[i]<-quantile(data,0.25)
  q3[i]<-quantile(data,0.75)
  med[i]<-median(data)
  iqd <- q3[i]-q1[i]
  upper[i] <- min( q3[i] + range*iqd, data.max )
  lower[i] <- max( q1[i] - range*iqd, data.min )

  #   strategy:
  #       xmin = min(lower, data.min))
  #       ymax = max(upper, data.max))
  #

  est.xlim <- c( min(lower[i], data.min), max(upper[i], data.max) )

  # estimate density curve
  smout <- do.call("sm.density", c( list(data, xlim=est.xlim), args ) )

  # calculate stretch factor
  #
  #  the plots density heights is defined in range 0.0 ... 0.5
  #  we scale maximum estimated point to 0.4 per data
  #
  hscale <- 0.4/max(smout$estimate) * wex

  # add density curve x,y pair to lists
  base[[i]]   <- smout$eval.points
  height[[i]] <- smout$estimate * hscale

  # calculate min,max base ranges
  t <- range(base[[i]])
  baserange[1] <- min(baserange[1],t[1])
  baserange[2] <- max(baserange[2],t[2])
```

```
}

# pass 2
#
# - plot graphics

# setup parameters for plot
if(!add){
  xlim <- if(n==1)
    at + c(-.5, .5)
  else
    range(at) + min(diff(at))/2 * c(-1,1)

  if (is.null(ylim)) {
    ylim <- baserange
  }
}
if (is.null(names)) {
  label <- 1:n
} else {
  label <- names
}

boxwidth <- 0.05 * wex

# setup plot
if(!add)
  plot.new()
if(!horizontal) {
  if(!add){
    plot.window(xlim = xlim, ylim = ylim)
    axis(2)
    axis(1,at = at, label=label )
  }

  box()
  for(i in 1:n) {
    # plot left/right density curve
    polygon( c(at[i]-height[[i]], rev(at[i]+height[[i]])),
             c(base[[i]], rev(base[[i]])),
             col = col[i %% length(col) + 1], border=border, lty=lty, lwd=lwd)

    if(drawRect){
      # plot IQR
      lines( at[c( i, i)], c(lower[i], upper[i]) ,lwd=lwd, lty=lty)

      # plot 50% KI box
      rect( at[i]-boxwidth/2, q1[i], at[i]+boxwidth/2, q3[i], col=rectCol)

      # plot median point
      points( at[i], med[i], pch=pchMed, col=colMed )
    }
  }
```

```
  }
  else {
    if(!add){
      plot.window(xlim = ylim, ylim = xlim)
      axis(1)
      axis(2,at = at, label=label )
    }

    box()
    for(i in 1:n) {
      # plot left/right density curve
      polygon( c(base[[i]], rev(base[[i]])),
               c(at[i]-height[[i]], rev(at[i]+height[[i]])),
               col = col[i %% length(col) + 1], border=border, lty=lty, lwd=lwd)

      if(drawRect){
        # plot IQR
        lines( c(lower[i], upper[i]), at[c(i,i)] ,lwd=lwd, lty=lty)

        # plot 50% KI box
        rect( q1[i], at[i]-boxwidth/2, q3[i], at[i]+boxwidth/2,  col=rectCol)

        # plot median point
        points( med[i], at[i], pch=pchMed, col=colMed )
      }
    }
  }
  invisible (list( upper=upper, lower=lower, median=med, q1=q1, q3=q3))
}

# Loop through the gene list to make a violin plot for each gene
fname = paste(name, "log2cpm.violin.plots.pdf", sep=".")

pdf(file=fname, onefile = TRUE)

for (i in 1:length(genes))
{
  idx <- which(rownames(p.anns)==genes[i])
  sym <- p.anns$Associated.Gene.Name[idx]

  if (length(idx) > 0) {
  idx <- idx[1]
  vioplot(y1[,idx], y2[,idx], y3[,idx], y4[,idx], y5[,idx], y6[,idx], y7[,idx],
    names = c(name1,name2,name3,name4,name5,name6,name7),
    col = c(grey[7],"#e41a1c", "#377eb8", "#4daf4a", "#984ea3", grey[8], grey[5]),
    ylim = c(0,20))
  title(main = sym, ylab = "log2(CPM)", xlab = "Cell Type")
  } else {}
}

dev.off()
```

Session Information

```r
suppressPackageStartupMessages(library(Biobase))
suppressPackageStartupMessages(library(RColorBrewer))
suppressPackageStartupMessages(library(edgeR))
suppressPackageStartupMessages(library(sm))
library(edgeR)
library(Biobase)
library(RColorBrewer)
library(sm)
sessionInfo()
```

```
## R version 3.3.0 (2016-05-03)
## Platform: x86_64-apple-darwin13.4.0 (64-bit)
## Running under: OS X 10.11.3 (El Capitan)
##
## locale:
## [1] en_US.UTF-8/en_US.UTF-8/en_US.UTF-8/C/en_US.UTF-8/en_US.UTF-8
##
## attached base packages:
## [1] parallel  stats     graphics  grDevices utils     datasets  methods
## [8] base
##
## other attached packages:
## [1] sm_2.2-5.4          edgeR_3.14.0        limma_3.28.7
## [4] RColorBrewer_1.1-2  Biobase_2.32.0      BiocGenerics_0.18.0
##
## loaded via a namespace (and not attached):
##  [1] Rcpp_0.12.5     digest_0.6.9    formatR_1.4     magrittr_1.5
##  [5] evaluate_0.9    stringi_1.1.1   rmarkdown_0.9.6 tools_3.3.0
##  [9] stringr_1.0.0   yaml_2.1.13     htmltools_0.3.5 knitr_1.13
```
