# Supplementary material for: Single-cell transcriptomes identify human islet cell signatures and reveal cell-type–specific expression changes in type 2 diabetes
Source: Genome Res. 2017 Feb;27(2):208–22. doi: 10.1101/gr.212720.116 (PMC5287227; doi:10.1101/gr.212720.116)
Supplement: Supplemental Material [file supp_gr.212720.116_Supplemental_Methods_Source_Code.zip › Supplemental_Methods_Source_Code/Figure3_Source_Code/Figure_3D_3E_Source_Code.pdf]

Alpha and Beta Cell Type Specific Barplots

Introduction

This report will explain the steps used to make barplots depicting the numbers of previously reported beta and alpha specific genes from Dorrell et al., 2011, Nica et al., 2013, Bramswig et al., 2013, and Blodgett et al., 2015 confirmed to be enriched in each cell type.

Barplot of Alpha Specific Genes

```
rm(list=ls())
# Load in ANOVA results for alpha reported genes
setwd("/Users/lawlon/Documents/Final_RNA_Seq_3/ANOVA_3/Cell_Type_Reported/")
res <- read.csv(file = "NonT2D.Blodgett.Bramswig.Dorrell.Nica.Alpha.Reported.Genes.ANOVA.stats.csv",
                header = TRUE, check.names = FALSE, row.names = 1)

# Only use values that don't have zeros
res.sel <- res[res[,2] != 0,]

# Define colors for each group
  # Beta alpha, delta, gamma
cols <- c("#e41a1c","#4daf4a","#984ea3","#377eb8")

# Symbol names for each cell type
new.names <- c("Beta", "Delta", "Gamma","Alpha")
```

```
bplot <- barplot(height = res.sel[c(1,3,4,2),2], horiz = TRUE,
                 xlab = "Number of Genes out of total 1853", ylab = "",
        axisnames = TRUE, names.arg = new.names, las=1, col = cols ,
        main = "", cex.names = 0.75, xlim = c(0,120))
# Add number labels to bars
text(x = res.sel[c(1,3,4,2),2]+4, y = bplot,
     labels = as.character(res.sel[c(1,3,4,2),2]), xpd = TRUE)
```

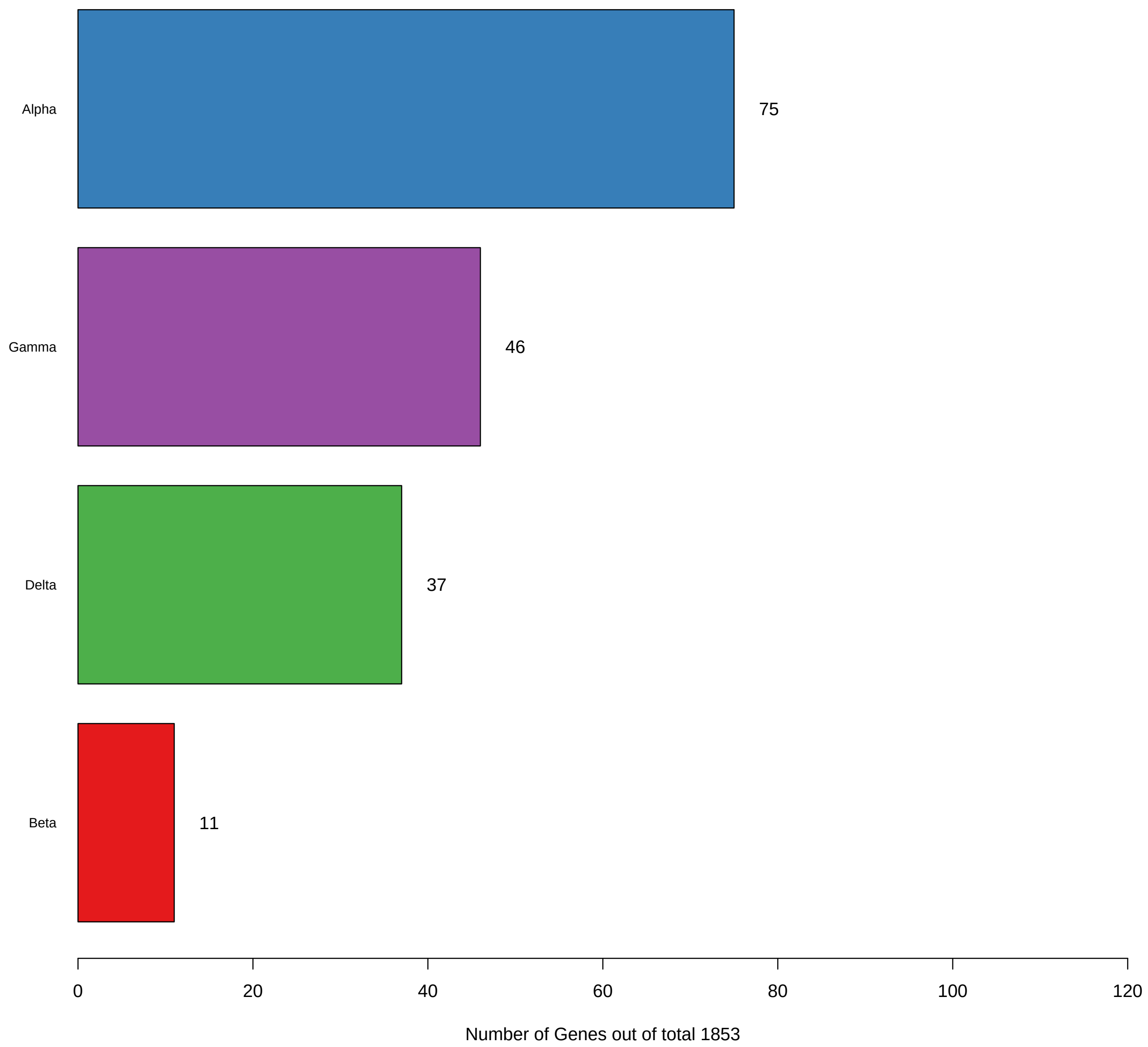

Figure 1: Barplot showing the number of alpha reported genes expressed in each cell type.

Barplot of Beta Specific Genes

```
rm(list=ls())
setwd("/Users/lawlon/Documents/Final_RNA_Seq_3/ANOVA_3/Cell_Type_Reported/")
res <- read.csv(file = "NonT2D.Blodgett.Bramswig.Dorrell.Nica.Beta.Reported.Genes.ANOVA.stats.csv",
                header = TRUE, check.names = FALSE, row.names = 1)

# Only use values that don't have zeros
res.sel <- res[res[,2] != 0,]

# Define colors for each group
  # Beta alpha, delta, gamma
cols <- c("#377eb8", "#984ea3", "#4daf4a","#e41a1c")

# Symbol names for each cell type
new.names <- c("Alpha", "Gamma", "Delta", "Beta")
```

```
# Make barplot
bplot <- barplot(height = res.sel[c(2,4,3,1),2], horiz = TRUE,
                 xlab = "Number of Genes out of total 1683", ylab = "",
        axisnames = TRUE, names.arg = new.names, las=1,
        col = cols , main = "", cex.names = 0.75, xlim = c(0,120))
# Add numbers to barplot
text(x = res.sel[c(2,4,3,1),2]+4, y = bplot,
     labels = as.character(res.sel[c(2,4,3,1),2]), xpd = TRUE)
```

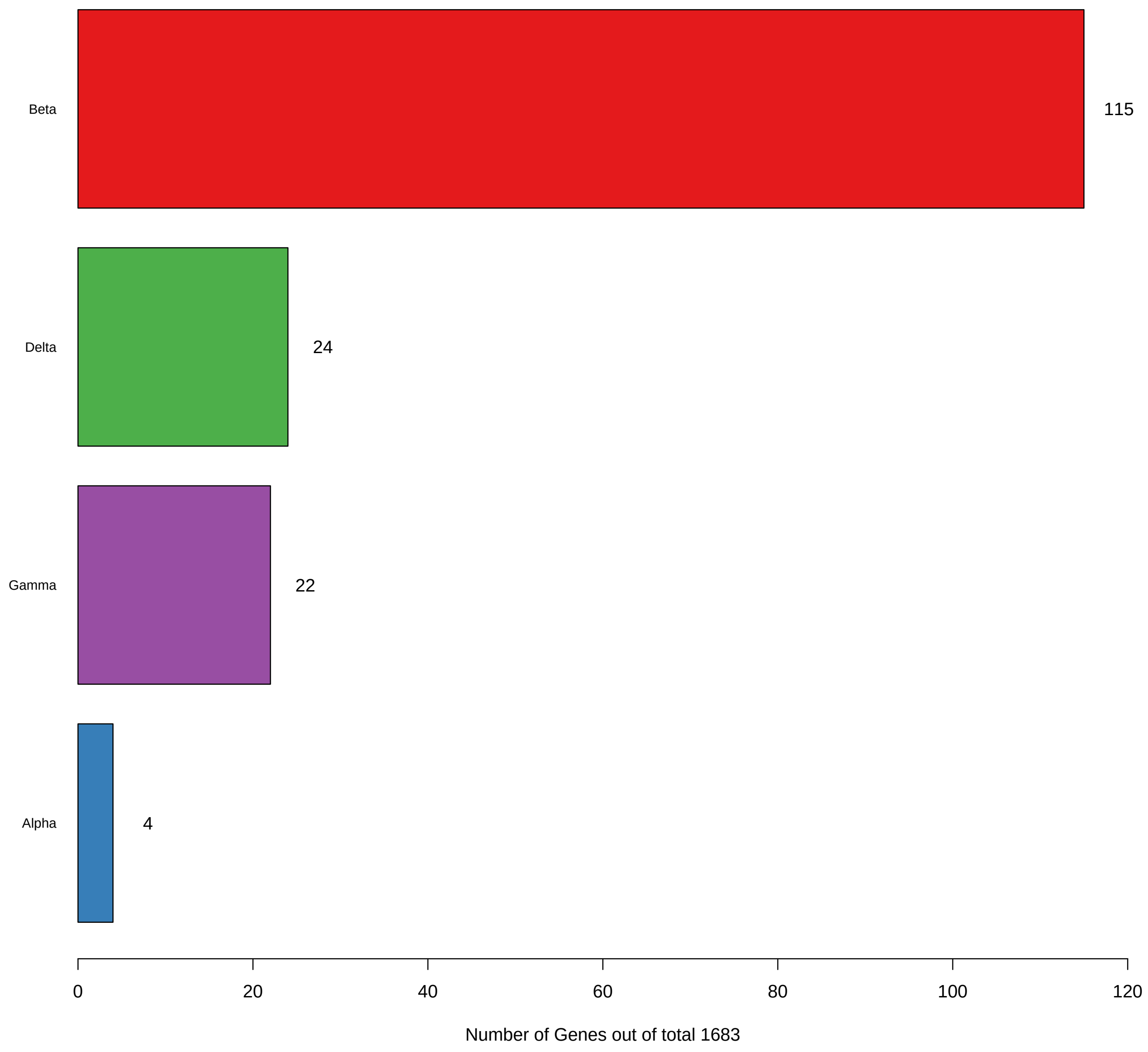

Figure 2: Barplot showing the number of beta reported genes expressed in each cell type.

Session Information

```r
sessionInfo()
```

```
## R version 3.3.0 (2016-05-03)
## Platform: x86_64-apple-darwin13.4.0 (64-bit)
## Running under: OS X 10.11.3 (El Capitan)
##
## locale:
## [1] en_US.UTF-8/en_US.UTF-8/en_US.UTF-8/C/en_US.UTF-8/en_US.UTF-8
##
## attached base packages:
## [1] stats     graphics  grDevices utils     datasets  methods   base
##
## loaded via a namespace (and not attached):
##  [1] magrittr_1.5    formatR_1.4     tools_3.3.0     htmltools_0.3.5
##  [5] yaml_2.1.13     Rcpp_0.12.5     stringi_1.1.1   rmarkdown_0.9.6
##  [9] knitr_1.13      stringr_1.0.0   digest_0.6.9    evaluate_0.9
```
