# Supplementary material for: Single-cell transcriptomes identify human islet cell signatures and reveal cell-type–specific expression changes in type 2 diabetes
Source: Genome Res. 2017 Feb;27(2):208–22. doi: 10.1101/gr.212720.116 (PMC5287227; doi:10.1101/gr.212720.116)
Supplement: Supplemental Material [file supp_gr.212720.116_Supplemental_Methods_Source_Code.zip › Supplemental_Methods_Source_Code/Figure3_Source_Code/Figure_3F_Source_Code.pdf]

Alpha and Beta Cell Type Specific Heatmap

Introduction

This report will explain the steps used to make a heatmap produce of log2(CPM) expression of all identified signature genes across all non-diabetic single cell samples. All non-diabetic endocrine samples were included in the heat map excluding "none" and "multiple" classified samples. The resulting heat map shows the log2(CPM) expression of genes after mean centering and scalining the values between -1 and 1.

```
suppressPackageStartupMessages(library(Biobase))
suppressPackageStartupMessages(library(pheatmap))
suppressPackageStartupMessages(library(RColorBrewer))
suppressPackageStartupMessages(library(edgeR))
suppressPackageStartupMessages(library(gplots))
suppressPackageStartupMessages(library(ggplot2))
library(Biobase)
library(pheatmap)
library(gplots)
library(ggplot2)
library(edgeR)
library(RColorBrewer)
rm(list=ls())
# Load in data
setwd("/Users/lawlon/Documents/Final_RNA_Seq_3/Data/")
load("nonT2D.rdata")
s.anns <- pData(cnts.eset)
p.anns <- as(featureData(cnts.eset), "data.frame")
counts <- exprs(cnts.eset)
# Calculate the cpm of the data
cpms <- cpm(x = counts)
data <- log2(cpms+1)

# Get endocrine cell types in order
s.1 <- s.anns[s.anns$cell.type %in% c("INS"),]
s.2 <- s.anns[s.anns$cell.type %in% c("GCG"),]
s.3 <- s.anns[s.anns$cell.type %in% c("SST"),]
s.4 <- s.anns[s.anns$cell.type %in% c("PPY"),]

# Get Expression matrices and average mean expression
f.1 <- data[, rownames(s.1)]
avg1 <- rowMeans(f.1)
f.2 <- data[, rownames(s.2)]
avg2 <- rowMeans(f.2)
f.3 <- data[, rownames(s.3)]
avg3 <- rowMeans(f.3)
f.4 <- data[, rownames(s.4)]
avg4 <- rowMeans(f.4)

# Match up cell type with hormone marker
namelist <- c(INS="Beta", GCG="Alpha", SST="Delta", PPY="Gamma")

# Combine all cell expression data into one matrix
```

```r
mat.orig <- cbind(f.1, f.2, f.3, f.4)
mat.avg <- cbind(avg1, avg2, avg3, avg4)
colnames(mat.avg) <- c("Beta", "Alpha", "Delta", "Gamma")
# Transpose avg matrix
trans.mat <- t(mat.avg)

# Change colnames of mat to gene symbol
colnames(trans.mat) <- p.anns$Associated.Gene.Name

# Specify genes of interest
genes.sel <- c("PDX1", "TMEM37", "TSPAN1", "SAMD11", "SLC25A34",
               "ENTPD3", "HADH", "RBP4", "CASR", "PVRL3",
                "FSTL5", "PLCE1", "FXYD5", "FAP", "CAMK2G",
               "SLC40A1", "FXYD3", "NPNT", "ARX", "GC")
# Extract ids for selected genes
genes.ids <- NULL
for (i in 1:length(genes.sel)) {
  idx <- which(colnames(trans.mat) == genes.sel[i])
  genes.ids <- c(genes.ids, idx)
}

# Extract genes of interest
mat.sel <- trans.mat[, genes.ids]
# Mean center by column (gene)
center_apply <- function(x) {
  apply(x, 2, function(y) y - mean(y))
}
mat.center <- center_apply(mat.sel)

# Scale the data between -1 and 1
nor.min.max <- function(x) {
  if (is.numeric(x) == FALSE) {
    stop("Please input numeric for x")
  }
  x.min <- min(x)
  x.max <- max(x)
  x <- 2*((x - x.min) / (x.max - x.min)) - 1
  return (x)
}
mat.scale <- t(apply(mat.center, 1, nor.min.max))

# Annotation matrix
annotation_col = data.frame(Cell_Type = colnames(mat.scale))
rownames(annotation_col) <- colnames(mat.scale)

# Specify cell type colors
grey <- brewer.pal(n=9, name="Greys")

ann_colors <- list(
  Cell_Type = c(Beta="#e41a1c", Alpha = "#377eb8", Delta = "#4daf4a", Gamma = "#984ea3"))

# Reorder the matrix
mat.scale <- mat.scale[c(1,3,2,4),]
```

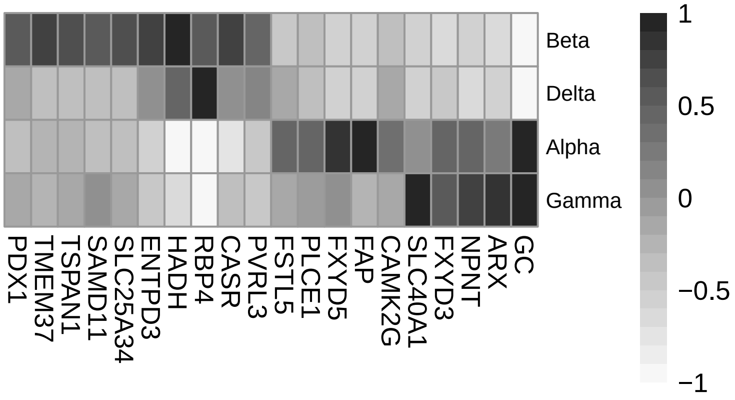

Figure 1: Heat map of scaled, average log2(CPM) expression of beta and alpha specific genes across non-diabetic endocrine single cell samples.

```r
# Make heatmap
pheatmap(mat = mat.scale, cluster_rows = FALSE, cluster_cols = FALSE,
         color =  colorRampPalette(brewer.pal(n = 7, name ="Greys"))(20),
         annotation_colors = ann_colors, clustering_distance_rows = NULL,
         clustering_method=NULL,show_rownames=TRUE,
         show_colnames = TRUE, annotation_names_row = TRUE,
         annotation_names_col = FALSE, trace = "none", fontsize_row = 8,
         cellwidth = 10, cellheight = 20, annotation_legend = FALSE)
```

Session Information

```r
# Load libraries
suppressPackageStartupMessages(library(Biobase))
suppressPackageStartupMessages(library(pheatmap))
suppressPackageStartupMessages(library(RColorBrewer))
suppressPackageStartupMessages(library(edgeR))
suppressPackageStartupMessages(library(gplots))
suppressPackageStartupMessages(library(ggplot2))
library(Biobase)
library(pheatmap)
library(gplots)
library(ggplot2)
library(edgeR)
library(RColorBrewer)
sessionInfo()
```

```
## R version 3.3.0 (2016-05-03)
## Platform: x86_64-apple-darwin13.4.0 (64-bit)
## Running under: OS X 10.11.3 (El Capitan)
##
## locale:
## [1] en_US.UTF-8/en_US.UTF-8/en_US.UTF-8/C/en_US.UTF-8/en_US.UTF-8
##
## attached base packages:
## [1] parallel  stats     graphics  grDevices utils     datasets  methods
## [8] base
##
## other attached packages:
## [1] ggplot2_2.1.0       gplots_3.0.1        edgeR_3.14.0
## [4] limma_3.28.7        RColorBrewer_1.1-2  pheatmap_1.0.8
## [7] Biobase_2.32.0      BiocGenerics_0.18.0
##
## loaded via a namespace (and not attached):
##  [1] Rcpp_0.12.5        knitr_1.13         magrittr_1.5
##  [4] munsell_0.4.3      colorspace_1.2-6   stringr_1.0.0
##  [7] plyr_1.8.4         caTools_1.17.1     tools_3.3.0
## [10] grid_3.3.0         gtable_0.2.0       KernSmooth_2.23-15
## [13] htmltools_0.3.5    gtools_3.5.0       yaml_2.1.13
## [16] digest_0.6.9       formatR_1.4        bitops_1.0-6
## [19] evaluate_0.9       rmarkdown_0.9.6    gdata_2.17.0
## [22] stringi_1.1.1      scales_0.4.0
```
