# Supplementary material for: Single-cell transcriptomes identify human islet cell signatures and reveal cell-type–specific expression changes in type 2 diabetes
Source: Genome Res. 2017 Feb;27(2):208–22. doi: 10.1101/gr.212720.116 (PMC5287227; doi:10.1101/gr.212720.116)
Supplement: Supplemental Material [file supp_gr.212720.116_Supplemental_Methods_Source_Code.zip › Supplemental_Methods_Source_Code/Figure4_Source_Code/Figure_4A_Source_Code.pdf]

Glycolysis and Gluconeogenesis Pathway Heatmap

Introduction

This report will explain the steps used to make a heatmap produce of average log2(CPM) expression of selected glycolysis and gluconeogenesis genes across all non-diabetic single cell samples. All non-diabetic samples were included in the heat map excluding “none” and “multiple” classified samples.

```
# Load in libraries
suppressPackageStartupMessages(library(Biobase))
suppressPackageStartupMessages(library(pheatmap))
suppressPackageStartupMessages(library(RColorBrewer))
suppressPackageStartupMessages(library(edgeR))
suppressPackageStartupMessages(library(gplots))
suppressPackageStartupMessages(library(ggplot2))
library(Biobase)
library(pheatmap)
library(gplots)
library(ggplot2)
library(edgeR)
library(RColorBrewer)
rm(list=ls())
# Load in data
setwd("/Users/lawlon/Documents/Final_RNA_Seq_3/Data/")
load("nonT2D.rdata")
s.anns <- pData(cnts.eset)
p.anns <- as(featureData(cnts.eset), "data.frame")
counts <- exprs(cnts.eset)
# Calculate the cpm of the data
cpms <- cpm(x = counts)
data <- log2(cpms+1)

# Get cell types in order
s.1 <- s.anns[s.anns$cell.type %in% c("INS"),]
s.2 <- s.anns[s.anns$cell.type %in% c("GCG"),]
s.3 <- s.anns[s.anns$cell.type %in% c("SST"),]
s.4 <- s.anns[s.anns$cell.type %in% c("PPY"),]
s.5 <- s.anns[s.anns$cell.type %in% c("PRSS1"),]
s.6 <- s.anns[s.anns$cell.type %in% c("KRT19"),]
s.7 <- s.anns[s.anns$cell.type %in% c("COL1A1"),]

# Get Expression matrices and average mean expression
f.1 <- data[, rownames(s.1)]
avg1 <- rowMeans(f.1)
f.2 <- data[, rownames(s.2)]
avg2 <- rowMeans(f.2)
f.3 <- data[, rownames(s.3)]
avg3 <- rowMeans(f.3)
f.4 <- data[, rownames(s.4)]
avg4 <- rowMeans(f.4)
f.5 <- data[, rownames(s.5)]
avg5 <- rowMeans(f.5)
```

```
f.6 <- data[, rownames(s.6)]
avg6 <- rowMeans(f.6)
f.7 <- data[, rownames(s.7)]
avg7 <- rowMeans(f.7)

# Match up cell type with hormone marker
namelist <- c(INS="Beta", GCG="Alpha", SST="Delta", PPY="Gamma",
              GHRL="Epsilon", COL1A1="Stellate", PRSS1="Acinar",
              KRT19="Ductal", none="None")
# Combine all cell expression data into one matrix
mat.orig <- cbind(f.1, f.2, f.3, f.4, f.5, f.6, f.7)
mat.avg <- cbind(avg1, avg2, avg3, avg4, avg5, avg6, avg7)
colnames(mat.avg) <- c("Beta", "Alpha", "Delta", "Gamma",
                       "Acinar", "Ductal", "Stellate")
# Transpose avg matrix
trans.mat <- t(mat.avg)
# Change colnames of mat to gene symbol
colnames(trans.mat) <- p.anns$Associated.Gene.Name

# Glycolysis pathway genes
genes.sel <- c("SLC2A1", "SLC2A2", "SLC2A3", "GCK", "HK1", "HK2",
               "GPI", "PFKL", "PFKM", "PFKP", "ALDOA", "ALDOB", "ALDOC",
               "GAPDH", "PGK1", "PGAM1", "PGAM5", "ENO1", "ENO2", "ENO3",
               "ENO4", "PKM", "LDHA", "LDHB", "LDHC",
               "G6PC2", "PFKFB2", "GPD1", "GPD2")

# Find ids of genes
genes.ids <- NULL
for (i in 1:length(genes.sel)) {
  idx <- which(colnames(trans.mat) == genes.sel[i])
  genes.ids <- c(genes.ids, idx)
}

# Extract genes of interest
mat.sel <- trans.mat[, genes.ids]
# transpose again
mat.sel <- t(mat.sel)

# Mean center the data
# Mean center by column (gene)
center_apply <- function(x) {
  apply(x, 1, function(y) y - mean(y))
}

mat.center <- center_apply(mat.sel)

# Scale the data between -1 and 1
nor.min.max <- function(x) {
  if (is.numeric(x) == FALSE) {
    stop("Please input numeric for x")
  }
  x.min <- min(x)
  x.max <- max(x)
```

```r
  x <- 2*((x - x.min) / (x.max - x.min)) - 1
  return (x)
}

# Only use if want to scale data
mat.scale <- t(apply(mat.center, 2, nor.min.max))

# Annotation matrix
annotation_col = data.frame(Cell_Type = colnames(mat.scale))
rownames(annotation_col) <- colnames(mat.scale)

# Specify cell type colors
grey <- brewer.pal(n=9, name="Greys")

ann_colors <- list(
  Cell_Type = c(Beta="#e41a1c", Alpha = "#377eb8",
                Delta = "#4daf4a", Gamma = "#984ea3",
                Acinar = "#525252", Ductal = "#969696", Stellate = "#000000"))
```

```r
# Make heatmap
pheatmap(mat = mat.sel, cluster_rows = FALSE, cluster_cols = FALSE,
         color =  colorRampPalette(brewer.pal(n = 7, name ="Greys"))(20),
         annotation_colors = ann_colors, clustering_distance_rows = NULL,
         clustering_method=NULL,show_rownames=TRUE,
         show_colnames = TRUE, annotation_names_row = TRUE,
         annotation_names_col = FALSE, trace = "none", fontsize_row = 8,
         cellwidth = 16, cellheight = 16, annotation_legend = FALSE)
```

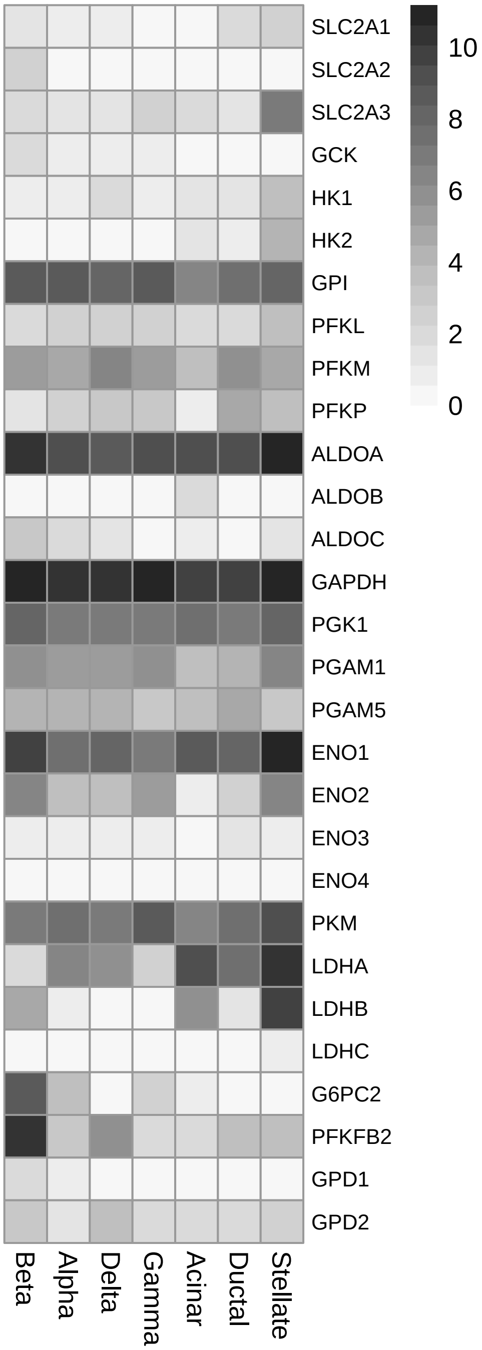

Figure 1: Heat map of average log2(CPM) expression of glycolysis and gluconeogenesis genes across non-diabetic endocrine single cell samples.

Session Information

```r
# Load libraries
suppressPackageStartupMessages(library(Biobase))
suppressPackageStartupMessages(library(pheatmap))
suppressPackageStartupMessages(library(RColorBrewer))
suppressPackageStartupMessages(library(edgeR))
suppressPackageStartupMessages(library(gplots))
suppressPackageStartupMessages(library(ggplot2))
library(Biobase)
library(pheatmap)
library(gplots)
library(ggplot2)
library(edgeR)
library(RColorBrewer)
sessionInfo()
```

```
## R version 3.3.0 (2016-05-03)
## Platform: x86_64-apple-darwin13.4.0 (64-bit)
## Running under: OS X 10.11.3 (El Capitan)
##
## locale:
## [1] en_US.UTF-8/en_US.UTF-8/en_US.UTF-8/C/en_US.UTF-8/en_US.UTF-8
##
## attached base packages:
## [1] parallel  stats     graphics  grDevices utils     datasets  methods
## [8] base
##
## other attached packages:
## [1] ggplot2_2.1.0       gplots_3.0.1        edgeR_3.14.0
## [4] limma_3.28.7        RColorBrewer_1.1-2  pheatmap_1.0.8
## [7] Biobase_2.32.0      BiocGenerics_0.18.0
##
## loaded via a namespace (and not attached):
##  [1] Rcpp_0.12.5        knitr_1.13         magrittr_1.5
##  [4] munsell_0.4.3      colorspace_1.2-6   stringr_1.0.0
##  [7] plyr_1.8.4         caTools_1.17.1     tools_3.3.0
## [10] grid_3.3.0         gtable_0.2.0       KernSmooth_2.23-15
## [13] htmltools_0.3.5    gtools_3.5.0       yaml_2.1.13
## [16] digest_0.6.9       formatR_1.4        bitops_1.0-6
## [19] evaluate_0.9       rmarkdown_0.9.6    gdata_2.17.0
## [22] stringi_1.1.1      scales_0.4.0
```
