# Supplementary material for: Single-cell transcriptomes identify human islet cell signatures and reveal cell-type–specific expression changes in type 2 diabetes
Source: Genome Res. 2017 Feb;27(2):208–22. doi: 10.1101/gr.212720.116 (PMC5287227; doi:10.1101/gr.212720.116)
Supplement: Supplemental Material [file supp_gr.212720.116_Supplemental_Methods_Source_Code.zip › Supplemental_Methods_Source_Code/Figure5_Source_Code/Figure_5A_Source_Code.pdf]

Unsupervised Hierarchical Clustering of Non-diabetic and Type 2 Diabetic Single Cell Data

Introduction

This report describes the methods used to perform unsupervised hierarchical clustering of the combined Type 2 diabetic and non-diabetic single cell data. The samples were clustered using highly expressed genes with log2(CPM) values greater than 10.5 in at least one sample. The clustering was performed using the “hclust” function using Euclidean distance and Ward.D2 linkage. The resultant “fan” dendrogram image was produced using the “ape” and “dendextend” R-packages.

Reference: Paradis E., Claude J. & Strimmer K. 2004. APE: analyses of phylogenetics and evolution in R language. Bioinformatics 20: 289-290.

Preprocessing Steps

All single cell samples from our non-diabetic and diabetic cohorts were used except for the cells labeled as “multiples” yielding a total of 638 samples. Only highly expressed genes with a log2 CPM expression level greater than 10.5 in at least one sample were used to perform the clustering. Ultimately, 2754 genes were used in the analysis. A two dimensional plot of the resultant dendrogram is shown in the figure below. Similar unsupervised hierarchical clustering analyses were performed on the non-diabetic and Type 2 diabetic single cell data alone.

```
# Load libraries
suppressPackageStartupMessages(library(Biobase))
suppressPackageStartupMessages(library(gplots))
suppressPackageStartupMessages(library(dendextend))
suppressPackageStartupMessages(library(ape))
suppressPackageStartupMessages(library(knitr))
suppressPackageStartupMessages(library(RColorBrewer))
suppressPackageStartupMessages(library(edgeR))
library(Biobase)
library(gplots)
library(dendextend)
library(ape)
library(knitr)
library(RColorBrewer)
library(edgeR)
set.seed(2135435)

# Load non-diabetic single cell data
setwd("/Users/lawlon/Documents/Final_RNA_Seq_3/Data/")
```

```
load("nonT2D.rdata")
# Obtain gene annotation information
p.anns <- featureData(cnts.eset)
probe.anns <- as(p.anns,"data.frame")
# Extract sample annotation information
ND.anns <- pData(cnts.eset)
# Remove multiples and keep all other groups
ND.sel <- ND.anns[ND.anns$cell.type %in% c("INS", "PPY", "GCG", "SST",
                    "COL1A1", "KRT19", "PRSS1", "none"),]

# Calculate cpm
ND.counts <- exprs(cnts.eset)
ND.cpms <- cpm(x = ND.counts)
ND.cpm <- log2(ND.cpms+1)
# Obtain log2 cpm values for selected samples
ND.cpm.sel <- ND.cpm[, rownames(ND.sel)]

# Load type 2 diabetic single cell data
load("T2D.rdata")
# Obtain sample annotations
T2D.anns <- pData(cnts.eset)
# Remove multiples and keep all other groups
T2D.sel <- T2D.anns[T2D.anns$cell.type %in% c("INS", "PPY", "GCG", "SST",
                            "COL1A1", "KRT19", "PRSS1", "none"),]

# Calculate cpm
T2D.counts <- exprs(cnts.eset)
T2D.cpms <- cpm(x = T2D.counts)
T2D.cpm <- log2(T2D.cpms+1)
# Obtain log2 cpm values for selected samples
T2D.cpm.sel <- T2D.cpm[, rownames(T2D.sel)]

# Combine sample anns and expression data
cpm.vals <- cbind(ND.cpm.sel, T2D.cpm.sel)
s.anns.sel <- rbind(ND.sel, T2D.sel)

# Filter gene by those with max cpm value greater than 10.5 (highly expressed genes)
r.max <- apply(cpm.vals,1,max)
cpm.res <- cpm.vals[r.max > 10.5,]

# Change column name labels to cell type and disease state
colnames(cpm.res)[1:dim(ND.sel)[1]] <- paste(ND.sel$cell.type, "NonT2D", sep="-")
colnames(cpm.res)[(dim(ND.sel)[1]+1):dim(cpm.res)[2]] <- paste(T2D.sel$cell.type, "T2
D", sep="-")

# Change name of one KRT19 cell to ghrelin cell
g <- which(probe.anns$Associated.Gene.Name == "GHRL")
ghrl <- cpm.vals[g,]
samp <- which(ghrl > 15)
g.idx <- which(rownames(s.anns.sel) == names(samp))
```

```r
colnames(cpm.res)[g.idx] <- "GHRL-NonT2D"

p.res <- probe.anns[rownames(cpm.res),]
# Combine probe anns with selected cpm values
cpm.res.exp <- cbind(p.res,cpm.res)
# Write selected gene matrix to file
#write.csv(cpm.res.exp, paste(fname, "genes_selected_for_cing.csv", sep = "."))

# Create an hclust object
d <- dist(t(cpm.res))
hc.final <- hclust(d,method="ward.D2")

# Create two dendrogram objects
dend1 <- as.dendrogram(hc.final)
dendcol <- as.dendrogram(hc.final)
# Create vectors of labels for cell types and disease
groupCodes1 <- s.anns.sel$cell.type
groupCodes <- c(rep("NonT2D", dim(ND.sel)[1]), rep("T2D", dim(T2D.sel)[1]))

# Color Schema for cell types
grey <- brewer.pal(n=9, name="Greys")
colorCodes1 <- c(INS="#e41a1c", GCG = "#377eb8", SST = "#4daf4a",
                 PPY = "#984ea3", GHRL = "#ff7f00",
                 COL1A1 = grey[9], PRSS1 = grey[7], KRT19 = grey[5],
                 none = grey[3])

# Color schema for disease state
colorCodes <- c(NonT2D="#bda2e5", T2D = "#10d2f0")
# Cell names that correspond to the hormone marker genes
namelist <- c("Beta", "Alpha", "Delta", "Gamma", "Epsilon",
              "Stellate", "Acinar", "Ductal", "none")
# Assign label colors to each dendrogram object
labels_colors(dend1) <- colorCodes[groupCodes][order.dendrogram(dend1)]
labels_colors(dendcol) <- colorCodes1[groupCodes1][order.dendrogram(dendcol)]

# Change dendrogram objects to phylo objects
dend2 <- as.phylo(dend1)
dend3 <- as.phylo(dendcol)

# Match up colors and labels for disease state
cols = NULL
for (i in 1:length(labels(dend2))) {
  if (grepl(x = dend2$tip.label[i], pattern = "NonT2D") == TRUE) {
    cols <- c(cols, colorCodes["NonT2D"])
  } else if (grepl(x = dend2$tip.label[i], pattern = "T2D") == TRUE) {
    cols <- c(cols, colorCodes["T2D"])
  }
}

# Match up cell type and color for dend3
```

```
col3 = NULL
for (i in 1:length(labels(dend3))) {
  if (grepl(x = dend3$tip.label[i], pattern = "INS") == TRUE) {
    col3 <- c(col3, colorCodes1["INS"])
  } else if (grepl(x = dend3$tip.label[i], pattern = "GCG") == TRUE) {
    col3 <- c(col3, colorCodes1["GCG"])
  } else if (grepl(x = dend3$tip.label[i], pattern = "SST") == TRUE) {
    col3 <- c(col3, colorCodes1["SST"])
  } else if (grepl(x = dend3$tip.label[i], pattern = "PPY") == TRUE) {
    col3 <- c(col3, colorCodes1["PPY"])
  } else if (grepl(x = dend3$tip.label[i], pattern = "COL1A1") == TRUE) {
    col3 <- c(col3, colorCodes1["COL1A1"])
  } else if (grepl(x = dend3$tip.label[i], pattern = "PRSS1") == TRUE) {
    col3 <- c(col3, colorCodes1["PRSS1"])
  } else if (grepl(x = dend3$tip.label[i], pattern = "KRT19") == TRUE) {
    col3 <- c(col3, colorCodes1["KRT19"])
  } else if (grepl(x = dend3$tip.label[i], pattern = "GHRL") == TRUE) {
    col3 <- c(col3, colorCodes1["GHRL"])
  } else if (grepl(x = dend3$tip.label[i], pattern = "none") == TRUE) {
    col3 <- c(col3, colorCodes1["none"])
  }
}

# Change cell type and disease state labels to hyphens for visualization
labels(dend2) <- rep(x = "—", length(labels(dend2)))
labels(dend3) <- rep(x = "—", length(labels(dend3)))

# Set margins
par(mar = c(0,0,0,0))

plot(dend2, type = "fan", tip.color = col3, cex = 6.0, label.offset = 0)
legend("bottomleft", title = "Cell Types", title.col = "black",
       legend = c(expression(bold("Beta (INS)")), expression(bold("Alpha (GCG)")),
       expression(bold("Delta (SST)")), expression(bold("Gamma (PPY)")),
       expression(bold("Epsilon (GHRL)")),
       expression(bold("Stellate (COL1A1)")), expression(bold("Acinar (PRSS1)")),
       expression(bold("Ductal (KRT19)")), expression(bold("None"))), text.col = colo
rCodes1,
       cex = 0.75, xjust=0, yjust=0)
legend("bottomright", title = "Disease State", title.col = "black",
       legend = c(expression(bold("NonT2D")), expression(bold("T2D"))),
       text.col = colorCodes,
       cex = 1.0, xjust=0, yjust=0)
par(new = TRUE)
plot(dend3, type = "fan", tip.color = cols, cex = 6.0, label.offset = 40)

# Note that increasing the cex value when plotting can make the color bars appear mor
e blended
# In addition, producing a high resolution tiff image as output is recommended:
  # tiff(file=paste(fname, "dendrogram.tiff", sep = "."),
```

```
#  width = 9000, height = 9000, units = "px", res = 800)
```

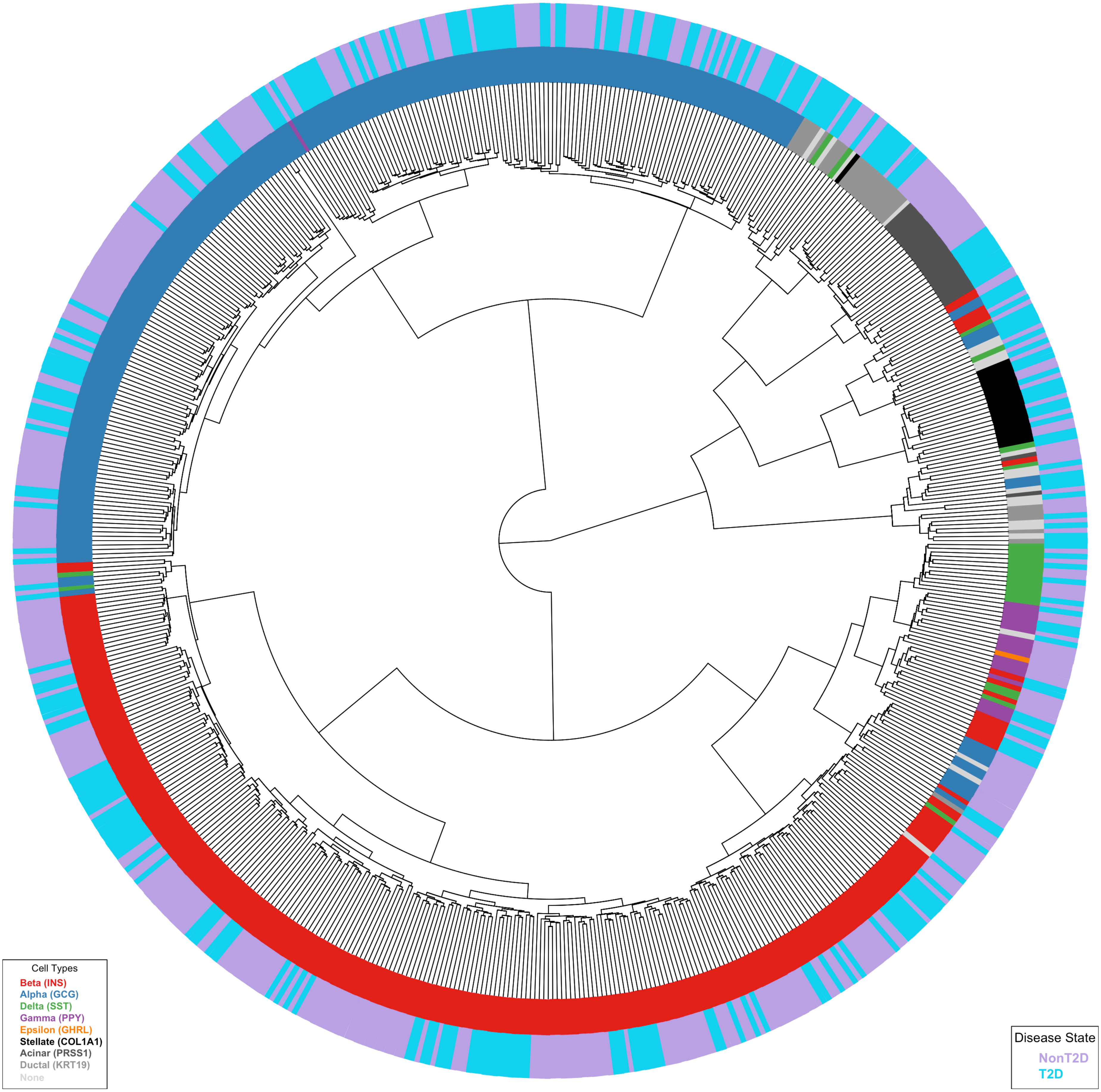

Figure 1: Unsupervised hierarchical clustering of non-diabetic and diabetic single cell data using genes with log2(CPM) values greater than 10.5 in at least one sample.

Session Information

```r
# Load libraries
suppressPackageStartupMessages(library(Biobase))
suppressPackageStartupMessages(library(gplots))
suppressPackageStartupMessages(library(dendextend))
suppressPackageStartupMessages(library(ape))
suppressPackageStartupMessages(library(knitr))
suppressPackageStartupMessages(library(RColorBrewer))
suppressPackageStartupMessages(library(edgeR))
library(Biobase)
library(gplots)
library(dendextend)
library(ape)
library(knitr)
library(RColorBrewer)
library(edgeR)
sessionInfo()
```

```
## R version 3.3.0 (2016-05-03)
## Platform: x86_64-apple-darwin13.4.0 (64-bit)
## Running under: OS X 10.11.3 (El Capitan)
##
## locale:
## [1] en_US.UTF-8/en_US.UTF-8/en_US.UTF-8/C/en_US.UTF-8/en_US.UTF-8
##
## attached base packages:
## [1] parallel  stats     graphics  grDevices utils     datasets  methods
## [8] base
##
## other attached packages:
## [1] edgeR_3.14.0        limma_3.28.7        RColorBrewer_1.1-2
## [4] knitr_1.13          ape_3.5             dendextend_1.1.8
## [7] gplots_3.0.1        Biobase_2.32.0      BiocGenerics_0.18.0
##
## loaded via a namespace (and not attached):
##  [1] Rcpp_0.12.5        whisker_0.3-2      magrittr_1.5
##  [4] lattice_0.20-33    stringr_1.0.0      caTools_1.17.1
##  [7] tools_3.3.0        grid_3.3.0         nlme_3.1-128
## [10] KernSmooth_2.23-15 htmltools_0.3.5    gtools_3.5.0
## [13] yaml_2.1.13        digest_0.6.9       formatR_1.4
## [16] bitops_1.0-6       evaluate_0.9       rmarkdown_0.9.6
## [19] gdata_2.17.0       stringi_1.1.1
```
