# Supplementary material for: Single-cell transcriptomes identify human islet cell signatures and reveal cell-type–specific expression changes in type 2 diabetes
Source: Genome Res. 2017 Feb;27(2):208–22. doi: 10.1101/gr.212720.116 (PMC5287227; doi:10.1101/gr.212720.116)
Supplement: Supplemental Material [file supp_gr.212720.116_Supplemental_Methods_Source_Code.zip › Supplemental_Methods_Source_Code/Figure5_Source_Code/Figure_5C_5D_5E_Source_Code.pdf]

Figure5

June 21, 2016

```
In [1]: %matplotlib inline
        import pandas as pd
        import numpy as np
        import matplotlib
        import matplotlib.pyplot as plt
        from matplotlib.backends.backend_pdf import PdfPages
        import scipy.stats as stats
        import scipy.cluster.hierarchy as sch
        from operator import *
        pd.core.config.option_context('mode.use_inf_as_null',True)
        import seaborn as sns
        rcdefsns = plt.rcParams.copy()
        import brewer2mpl
        import os
        import sys
        import bokeh
        from bokeh.plotting import ColumnDataSource, figure, show, gridplot,output_file,hplot,vplot
        from bokeh.models import HoverTool
        bokeh.io.output_notebook()

        matplotlib.rcParams['figure.figsize'] = (5.0, 5.0)
        matplotlib.rcParams['axes.linewidth'] = 3
        matplotlib.rcParams['axes.edgecolor'] = 'k'
        matplotlib.rcParams['axes.spines.top']='False'
        matplotlib.rcParams['axes.spines.right']='False'
        matplotlib.rcParams['axes.spines.right']='False'

        TOOLS="pan,wheel_zoom,box_zoom,reset,hover,lasso_select,save"

In [2]: bulk_dge=pd.read_csv('T2D.vs.NonT2D.Bulk.Intact.csv',index_col=0)
        sc_dge=pd.read_csv(
            'T2D_vs_NonT2D_Differential_Gene_Lists/EdgeR.Robust.T2D.vs.NonT2D.Gender.Covariate.Beta.csv
            index_col=0)
        sc_alpha=pd.read_csv(
            'T2D_vs_NonT2D_Differential_Gene_Lists/EdgeR.Robust.T2D.vs.NonT2D.Gender.Covariate.Alpha.cs
            index_col=0)
        sc_gamma=pd.read_csv(
            'T2D_vs_NonT2D_Differential_Gene_Lists/EdgeR.Robust.T2D.vs.NonT2D.Gender.Covariate.Gamma.cs
            index_col=0)
        sc_delta=pd.read_csv(
            'T2D_vs_NonT2D_Differential_Gene_Lists/EdgeR.Robust.T2D.vs.NonT2D.Gender.Covariate.Delta.cs
            index_col=0)

In [4]: matplotlib.rcParams['figure.figsize'] = (25.0, 5.0)
        fig,ax=plt.subplots(1,4,sharey=True)
```

```
ax[0].axvline(0,color='k',linestyle='--')
ax[0].axhline(0,color='k',linestyle='--')
ax[0].grid('off')
ax[0].set_axis_bgcolor('white')

merged=bulk_dge.join(sc_dge,rsuffix='SC',how='outer')
merged['color']=merged['FDRSC'].apply(lambda x: '#e41a1c' if x < 0.05 else '#bdbdbd')
merged.loc[merged[merged['Associated.Gene.Name']=='INS'].index,'color']='blue'
ax[0].axvline(0,color='k',linestyle='--')
hold=merged[merged['color']=='#bdbdbd']
ax[0].scatter(hold['logFCSC'],hold['logFC'],c='#bdbdbd',s=20,lw=0.,label='FDR > 0.05 SC')
hold=merged[merged['color']!='#bdbdbd']
ax[0].scatter(hold['logFCSC'],hold['logFC'],c='#e41a1c',s=40,lw=0., label='FDR < 0.05 SC')
ax[0].tick_params(axis='both', which='major', labelsize=16)
ax[0].set_ylabel('log(FC) - Bulk T2D vs ND', fontsize=16)
ax[0].set_xlabel(r'log(FC) - Single Cell $\beta$ T2D vs ND',fontsize=16)
ax[0].set_ylim(-3,3)
ax[0].set_xlim(-6,6)


ax[1].axvline(0,color='k',linestyle='--')
ax[1].axhline(0,color='k',linestyle='--')
ax[1].grid('off')
ax[1].set_axis_bgcolor('white')
merged=bulk_dge.join(sc_alpha,rsuffix='SC',how='outer')
merged['color']=merged['FDRSC'].apply(lambda x: '#377EB8' if x < 0.05 else '#bdbdbd')
hold=merged[merged['color']=='#bdbdbd']
ax[1].scatter(hold['logFCSC'],hold['logFC'],c='#bdbdbd',s=20,lw=0.,label='FDR > 0.05 SC')
hold=merged[merged['color']!='#bdbdbd']
ax[1].scatter(hold['logFCSC'],hold['logFC'],c=hold['color'],s=40,lw=0., label='FDR < 0.05 SC')
ax[1].tick_params(axis='both', which='major', labelsize=16)
ax[1].set_xlabel(r'log(FC) - Single Cell $\alpha$ T2D vs ND',fontsize=16)
ax[1].set_ylim(-3,3)
ax[1].set_xlim(-6,6)

ax[2].axvline(0,color='k',linestyle='--')
ax[2].axhline(0,color='k',linestyle='--')
ax[2].grid('off')
ax[2].set_axis_bgcolor('white')
merged=bulk_dge.join(sc_gamma,rsuffix='SC',how='outer')
merged['color']=merged['FDRSC'].apply(lambda x: 'green' if x < 0.05 else '#bdbdbd')
hold=merged[merged['color']=='#bdbdbd']
ax[2].scatter(hold['logFCSC'],hold['logFC'],c='#bdbdbd',s=20,lw=0.,label='FDR > 0.05 SC')
hold=merged[merged['color']=='green']
ax[2].scatter(hold['logFCSC'],hold['logFC'],c='green',s=40,lw=0., label='FDR < 0.05 SC')
ax[2].tick_params(axis='both', which='major', labelsize=16)
ax[2].set_xlabel(r'log(FC) - Single Cell $\gamma$ T2D vs ND',fontsize=16)
ax[2].set_ylim(-3,3)
ax[2].set_xlim(-8,8)

ax[3].axvline(0,color='k',linestyle='--')
ax[3].axhline(0,color='k',linestyle='--')
ax[3].grid('off')
ax[3].set_axis_bgcolor('white')
```

```
merged=bulk_dge.join(sc_delta,rsuffix='SC',how='outer')
merged['color']=merged['FDRSC'].apply(lambda x: '#4DAF4A' if x < 0.05 else '#bdbdbd')
hold=merged[merged['color']=='#bdbdbd']
ax[3].scatter(hold['logFCSC'],hold['logFC'],c='#bdbdbd',s=20,lw=0.,label='FDR > 0.05 SC')
hold=merged[merged['color']!='#bdbdbd']
ax[3].scatter(hold['logFCSC'],hold['logFC'],c='#4DAF4A',s=40,lw=0., label='FDR < 0.05 SC')
ax[3].tick_params(axis='both', which='major', labelsize=16)
ax[3].set_xlabel(r'log(FC) - Single Cell $\delta$ T2D vs ND',fontsize=16)
ax[3].set_ylim(-3,3)
ax[3].set_xlim(-15,15)

fig.tight_layout()
fig.savefig('Figures_06142016/Figure5_DGE.pdf',dpi=300,format='pdf')
```

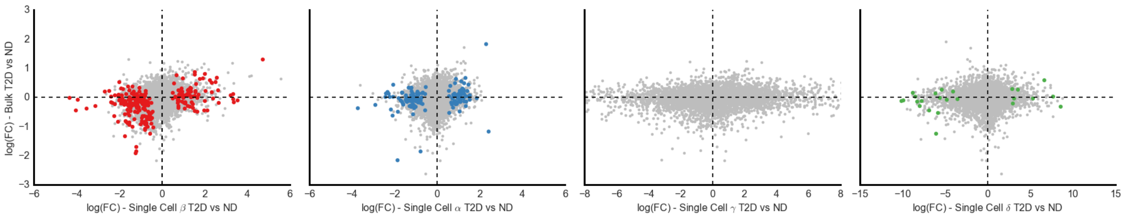

In [5]:
```
output_file("Figures_06142016/Figure5_DGE-T2DvND_Bulk-SC-BetavsBeta.html")
p = figure(title="DGE: T2D vs ND - Bulk and Single Cell", tools=TOOLS, width=650, plot_height=65

merged=bulk_dge.join(sc_dge,rsuffix='SC',how='outer')
merged['color']=merged['FDRSC'].apply(lambda x: '#e41a1c' if x < 0.05 else '#bdbdbd')
hold=merged[merged['color']=='#bdbdbd'].replace('nan',np.nan).dropna()
color='#bdbdbd'
genename=hold['Associated.Gene.Name'].values
fdr_bulk=hold['FDR'].values
fdr_sc=hold['FDRSC'].values
fc_bulk=hold['logFC'].values
fc_sc=hold['logFCSC'].values

source = ColumnDataSource(
data=dict(
    x=fc_bulk,
    y=fc_sc,
    a=genename,
    b=fdr_bulk,
    c=fdr_sc,
    d=fc_bulk,
    e=fc_sc
    ))

p.circle(fc_sc,fc_bulk,radius=.05,fill_color=color,line_color=color,source=source,legend='FDR >

hold=merged[merged['color']=='#e41a1c'].replace('nan',np.nan).dropna()
color='#e41a1c'
genename=hold['Associated.Gene.Name'].values
fdr_bulk=hold['FDR'].values
```

```
fdr_sc=hold['FDRSC'].values
fc_bulk=hold['logFC'].values
fc_sc=hold['logFCSC'].values

source = ColumnDataSource(
data=dict(
    x=fc_bulk,
    y=fc_sc,
    a=genename,
    b=fdr_bulk,
    c=fdr_sc,
    d=fc_bulk,
    e=fc_sc
    ))

p.circle(fc_sc,fc_bulk,radius=.05,fill_color=color,line_color=color,source=source,legend='FDR <

hover = p.select(dict(type=HoverTool))
hover.tooltips = [
("index", "$index"),
("log(FC)-Bulk,log(FC)-SC", "($x, $y)"),
("Gene", "@a"),
("FDR-Bulk", "@b"),
("FDR-SC", "@c"),
]
p.yaxis.axis_label='log(FC) - Bulk T2D vs ND'
p.xaxis.axis_label='log(FC) - Single Cell beta T2D vs ND'
#p.xaxis.bounds=(-6,6)
show(p)
```

Out[5]: <bokeh.io._CommsHandle at 0x116ccd810>

In [6]:
```
output_file("Figures_06142016/Figure5_DGE-T2DvND_Bulk-SC-Alpha.html")
p = figure(title="DGE: T2D vs ND - Bulk and Single Cell - Alpha", tools=TOOLS, width=650, plot_

merged=bulk_dge.join(sc_alpha,rsuffix='SC',how='outer')
merged['color']=merged['FDRSC'].apply(lambda x: 'green' if x < 0.05 else '#bdbdbd')
hold=merged[merged['color']=='#bdbdbd'].replace('nan',np.nan).dropna()
color='#bdbdbd'
genename=hold['Associated.Gene.Name'].values
fdr_bulk=hold['FDR'].values
fdr_sc=hold['FDRSC'].values
fc_bulk=hold['logFC'].values
fc_sc=hold['logFCSC'].values

source = ColumnDataSource(
data=dict(
    x=fc_bulk,
    y=fc_sc,
    a=genename,
    b=fdr_bulk,
    c=fdr_sc,
    d=fc_bulk,
    e=fc_sc
    ))
```

```
p.circle(fc_sc,fc_bulk,radius=.05,fill_color=color,line_color=color,source=source,legend='FDR >

hold=merged[merged['color']=='green'].replace('nan',np.nan).dropna()
color='#377EB8'
genename=hold['Associated.Gene.Name'].values
fdr_bulk=hold['FDR'].values
fdr_sc=hold['FDRSC'].values
fc_bulk=hold['logFC'].values
fc_sc=hold['logFCSC'].values

source = ColumnDataSource(
data=dict(
    x=fc_bulk,
    y=fc_sc,
    a=genename,
    b=fdr_bulk,
    c=fdr_sc,
    d=fc_bulk,
    e=fc_sc
    ))

p.circle(fc_sc,fc_bulk,radius=.05,fill_color=color,line_color=color,source=source,legend='FDR <

hover = p.select(dict(type=HoverTool))
hover.tooltips = [
("index", "$index"),
("log(FC)-Bulk,log(FC)-SC", "($x, $y)"),
("Gene", "@a"),
("FDR-Bulk", "@b"),
("FDR-SC", "@c"),
]
p.yaxis.axis_label='log(FC) - Bulk T2D vs ND'
p.xaxis.axis_label='log(FC) - Single Cell alpha T2D vs ND'
#p.xaxis.bounds=(-6,6)
show(p)
```

Out[6]: <bokeh.io._CommsHandle at 0x11ad57910>

In [8]:
```
output_file("Figures_06142016/Figure5_DGE-T2DvND_Bulk-SC-delta.html")
p = figure(title="DGE: T2D vs ND - Bulk and Single Cell - delta", tools=TOOLS, width=650, plot_

merged=bulk_dge.join(sc_delta,rsuffix='SC',how='outer')
merged['color']=merged['FDRSC'].apply(lambda x: 'green' if x < 0.05 else '#bdbdbd')
hold=merged[merged['color']=='#bdbdbd'].replace('nan',np.nan).dropna()
color='#bdbdbd'
genename=hold['Associated.Gene.Name'].values
fdr_bulk=hold['FDR'].values
fdr_sc=hold['FDRSC'].values
fc_bulk=hold['logFC'].values
fc_sc=hold['logFCSC'].values

source = ColumnDataSource(
data=dict(
    x=fc_bulk,
```

```
            y=fc_sc,
            a=genename,
            b=fdr_bulk,
            c=fdr_sc,
            d=fc_bulk,
            e=fc_sc
            ))

        p.circle(fc_sc,fc_bulk,radius=.05,fill_color=color,line_color=color,source=source,legend='FDR >


        hold=merged[merged['color']=='green'].replace('nan',np.nan).dropna()
        color='#4DAF4A'
        genename=hold['Associated.Gene.Name'].values
        fdr_bulk=hold['FDR'].values
        fdr_sc=hold['FDRSC'].values
        fc_bulk=hold['logFC'].values
        fc_sc=hold['logFCSC'].values

        source = ColumnDataSource(
        data=dict(
            x=fc_bulk,
            y=fc_sc,
            a=genename,
            b=fdr_bulk,
            c=fdr_sc,
            d=fc_bulk,
            e=fc_sc
            ))

        p.circle(fc_sc,fc_bulk,radius=.05,fill_color=color,line_color=color,source=source,legend='FDR <

        hover = p.select(dict(type=HoverTool))
        hover.tooltips = [
        ("index", "$index"),
        ("log(FC)-Bulk,log(FC)-SC", "($x, $y)"),
        ("Gene", "@a"),
        ("FDR-Bulk", "@b"),
        ("FDR-SC", "@c"),
        ]
        p.yaxis.axis_label='log(FC) - Bulk T2D vs ND'
        p.xaxis.axis_label='log(FC) - Single Cell delta T2D vs ND'
        #p.xaxis.bounds=(-6,6)
        show(p)
```

Out[8]: <bokeh.io._CommsHandle at 0x11a5ce550>

In [ ]:
