# Supplementary material for: Single-cell transcriptomes identify human islet cell signatures and reveal cell-type–specific expression changes in type 2 diabetes
Source: Genome Res. 2017 Feb;27(2):208–22. doi: 10.1101/gr.212720.116 (PMC5287227; doi:10.1101/gr.212720.116)
Supplement: Supplemental Material [file supp_gr.212720.116_Supplemental_Methods_Source_Code.zip › Supplemental_Methods_Source_Code/Figure5_Source_Code/Figure_5C_5D_5E_Violin_Plot_Source_Code.pdf]

Violin Plots of Diabetic vs Non-Diabetic Differentially Expressed Genes

Introduction

This report describes the steps used to produce violin plots of log2(CPM) expression of differentially genes across bulk intact islets, non-diabetic single cells, and type 2 diabetic single cells. These plots were produced to highlight cell type specific changes in gene expression that could not be detected in bulk islet differential expression analyses.

```
rm(list=ls())
# Load  in libraries
suppressPackageStartupMessages(library(Biobase))
suppressPackageStartupMessages(library(RColorBrewer))
suppressPackageStartupMessages(library(edgeR))
suppressPackageStartupMessages(library(sm))
library(edgeR)
library(Biobase)
library(RColorBrewer)
library(sm)
rm(list=ls())
setwd("/Users/lawlon/Documents/Final_RNA_Seq/")
# Load the data, get probe anns, sample anns
# Get only intact bulk samples
type <- "Intact"
load("islet_bulk_uniq_data.rdata")
p.anns <- as(featureData(bulk.cnts),"data.frame")
# Get count data
bulk.counts <- exprs(bulk.cnts)
# Get sample annotations
bulk.anns <- pData(bulk.cnts)
bulk.anns.sel <- bulk.anns[bulk.anns$Type == type,]
# Isolate ND samples
nonT2D.anns <- bulk.anns.sel[bulk.anns.sel$Phenotype == "ND",]
# Separate counts
ND.counts <- bulk.counts[, rownames(nonT2D.anns)]
# Calculate cpm
ND.cpm <- cpm(x = ND.counts)
ND.data <- log2(ND.cpm+1)

#Isolate T2D data for bulk islets
T2D.anns <- bulk.anns.sel[bulk.anns.sel$Phenotype == "T2D",]
# Separate counts
T2D.counts <- bulk.counts[, rownames(T2D.anns)]
# Calculate cpm
T2D.cpm <- cpm(x = T2D.counts)
T2D.data <- log2(T2D.cpm+1)

setwd("/Users/lawlon/Documents/Final_RNA_Seq_3/Data/")
# Load in single cell data
load("nonT2D.rdata")
```

```r
ND.sc.c <- exprs(cnts.eset)
# Calculate cpm
ND.sc.cpm <- cpm(x = ND.sc.c)
ND.sc.data <- log2(ND.sc.cpm+1)

# Single cell sample anns
ND.sc.anns <- pData(cnts.eset)
ND.betas <- ND.sc.anns[ND.sc.anns$cell.type == "INS",]
ND.alp <- ND.sc.anns[ND.sc.anns$cell.type == "GCG",]
ND.del <- ND.sc.anns[ND.sc.anns$cell.type == "SST",]

# Load in single cell data
load("T2D.rdata")
T2D.sc.c <- exprs(cnts.eset)
# Calculate cpm
T2D.sc.cpm <- cpm(x = T2D.sc.c)
T2D.sc.data <- log2(T2D.sc.cpm+1)

# Single cell sample anns
T2D.sc.anns <- pData(cnts.eset)
T2D.betas <- T2D.sc.anns[T2D.sc.anns$cell.type == "INS",]
T2D.alp <- T2D.sc.anns[T2D.sc.anns$cell.type == "GCG",]
T2D.del <- T2D.sc.anns[T2D.sc.anns$cell.type == "SST",]

## LOAD SELECTED GENE LIST ##
setwd("/Users/lawlon/Documents/Final_RNA_Seq_3/Differential_Expression_3/Single_Cell/T2D_vs_NonT2D_3/")
genes <- read.csv(file="EdgeR.Robust.T2D.vs.NonT2D.Gender.Covariate.Delta.FDR.0.05.csv", header=T,
                   check.names = F, row.names = 1)

# Names to display on violin plot
name1 <- "ND Bulk"
name2 <- "T2D"
name3 <- "ND Beta"
name4 <- "T2D"
name5 <- "ND Alpha"
name6 <- "T2D"
name7 <- "ND Delta"
name8 <- "T2D"

# Find which samples are labeled as which
# Bulk NonT2D islets
ND.bulk <- ND.data
# Check for zeros, prevent error for 0 values
y1 <- t(ND.bulk)
for (i in 1:dim(y1)[2]){
  if (max(y1[,i]) == 0) {
    y1[1,i] <- 0.00001
  }
}

# Bulk T2D intact islets
T2D.bulk <- T2D.data
# Check for zeros
```

```r
y2 <- t(T2D.bulk)
for (i in 1:dim(y2)[2]){
  if (max(y2[,i]) == 0) {
    y2[1,i] <- 0.00001
  }
}

# Beta ND single cell
beta.data <- ND.sc.data[, rownames(ND.betas)]
# Check for zeros
y3 <- t(beta.data)
for (i in 1:dim(y3)[2]){
  if (max(y3[,i]) == 0) {
    y3[1,i] <- 0.00001
  }
}

# Beta T2D single cell
beta.t2d.data <- T2D.sc.data[, rownames(T2D.betas)]
# Check for zeros
y4 <- t(beta.t2d.data)
for (i in 1:dim(y4)[2]){
  if (max(y4[,i]) == 0) {
    y4[1,i] <- 0.00001
  }
}

# Alpha ND
alp.data <- ND.sc.data[, rownames(ND.alp)]
# Check for zeros
y5 <- t(alp.data)
for (i in 1:dim(y5)[2]){
  if (max(y5[,i]) == 0) {
    y5[1,i] <- 0.00001
  }
}

# Alpha T2D
alp.t2d.data <- T2D.sc.data[, rownames(T2D.alp)]
# Check for zeros
y6 <- t(alp.t2d.data)
for (i in 1:dim(y6)[2]){
  if (max(y6[,i]) == 0) {
    y6[1,i] <- 0.00001
  }
}

# Delta ND
del.data <- ND.sc.data[, rownames(ND.del)]
# Check for zeros
y7 <- t(del.data)
for (i in 1:dim(y7)[2]){
  if (max(y7[,i]) == 0) {
```

```
    y7[1,i] <- 0.00001
  }
}

# Delta T2D
T2d.del.data <- T2D.sc.data[, rownames(T2D.del)]
# Check for zeros
y8 <- t(T2d.del.data)
for (i in 1:dim(y8)[2]){
  if (max(y8[,i]) == 0) {
    y8[1,i] <- 0.00001
  }
}

# Set color panel
grey <- brewer.pal(n=9, name="Greys")

## ALTERED VIOPLOT SOURCE CODE
vioplot <- function(x,...,range=1.5,h=NULL,ylim=NULL,names=NULL, horizontal=FALSE,
                    col="magenta", border="black", lty=1, lwd=1, rectCol="black",
                    colMed="white", pchMed=19, at, add=FALSE, wex=1,
                    drawRect=TRUE)
{
  # process multiple datas
  datas <- list(x,...)
  n <- length(datas)

  if(missing(at)) at <- 1:n

  # pass 1
  #
  # - calculate base range
  # - estimate density
  #

  # setup parameters for density estimation
  upper  <- vector(mode="numeric",length=n)
  lower  <- vector(mode="numeric",length=n)
  q1     <- vector(mode="numeric",length=n)
  q3     <- vector(mode="numeric",length=n)
  med    <- vector(mode="numeric",length=n)
  base   <- vector(mode="list",length=n)
  height <- vector(mode="list",length=n)
  baserange <- c(Inf,-Inf)

  # global args for sm.density function-call
  args <- list(display="none")

  if (!(is.null(h)))
    args <- c(args, h=h)

  for(i in 1:n) {
    data<-datas[[i]]
```

```
    # calculate plot parameters
    #   1- and 3-quantile, median, IQR, upper- and lower-adjacent
    data.min <- min(data)
    data.max <- max(data)
    q1[i]<-quantile(data,0.25)
    q3[i]<-quantile(data,0.75)
    med[i]<-median(data)
    iqd <- q3[i]-q1[i]
    upper[i] <- min( q3[i] + range*iqd, data.max )
    lower[i] <- max( q1[i] - range*iqd, data.min )

    #   strategy:
    #       xmin = min(lower, data.min))
    #       ymax = max(upper, data.max))
    #

    est.xlim <- c( min(lower[i], data.min), max(upper[i], data.max) )

    # estimate density curve
    smout <- do.call("sm.density", c( list(data, xlim=est.xlim), args ) )

    # calculate stretch factor
    #
    #  the plots density heights is defined in range 0.0 ... 0.5
    #  we scale maximum estimated point to 0.4 per data
    #
    hscale <- 0.4/max(smout$estimate) * wex

    # add density curve x,y pair to lists
    base[[i]]   <- smout$eval.points
    height[[i]] <- smout$estimate * hscale

    # calculate min,max base ranges
    t <- range(base[[i]])
    baserange[1] <- min(baserange[1],t[1])
    baserange[2] <- max(baserange[2],t[2])

  }

  # pass 2
  #
  # - plot graphics

  # setup parameters for plot
  if(!add){
    xlim <- if(n==1)
      at + c(-.5, .5)
    else
      range(at) + min(diff(at))/2 * c(-1,1)

    if (is.null(ylim)) {
      ylim <- baserange
    }
```

```r
}
if (is.null(names)) {
  label <- 1:n
} else {
  label <- names
}

boxwidth <- 0.05 * wex

# setup plot
if(!add)
  plot.new()
if(!horizontal) {
  if(!add){
    plot.window(xlim = xlim, ylim = ylim)
    axis(2)
    axis(1,at = at, label=label )
  }

  box()
  for(i in 1:n) {
    # plot left/right density curve
    polygon( c(at[i]-height[[i]], rev(at[i]+height[[i]])),
             c(base[[i]], rev(base[[i]])),
             col = col[i %% length(col) + 1], border=border, lty=lty, lwd=lwd)

    if(drawRect){
      # plot IQR
      lines( at[c( i, i)], c(lower[i], upper[i]) ,lwd=lwd, lty=lty)

      # plot 50% KI box
      rect( at[i]-boxwidth/2, q1[i], at[i]+boxwidth/2, q3[i], col=rectCol)

      # plot median point
      points( at[i], med[i], pch=pchMed, col=colMed )
    }
  }

}
else {
  if(!add){
    plot.window(xlim = ylim, ylim = xlim)
    axis(1)
    axis(2,at = at, label=label )
  }

  box()
  for(i in 1:n) {
    # plot left/right density curve
    polygon( c(base[[i]], rev(base[[i]])),
             c(at[i]-height[[i]], rev(at[i]+height[[i]])),
             col = col[i %% length(col) + 1], border=border, lty=lty, lwd=lwd)
```

```
      if(drawRect){
        # plot IQR
        lines( c(lower[i], upper[i]), at[c(i,i)] ,lwd=lwd, lty=lty)

        # plot 50% KI box
        rect( q1[i], at[i]-boxwidth/2, q3[i], at[i]+boxwidth/2,  col=rectCol)

        # plot median point
        points( med[i], at[i], pch=pchMed, col=colMed )
      }
    }
  }
  invisible (list( upper=upper, lower=lower, median=med, q1=q1, q3=q3))
}

## END VIOPLOT SOURCE CODE


# Loop through the gene list to make a violin plot for each gene
for(i in 1:dim(genes)[1])
{
  # Which gene id
  gen <- rownames(genes)[i]
  gen.id <- which(rownames(p.anns) == gen)

  sym <- p.anns$Associated.Gene.Name[gen.id]
    vioplot(y1[,gen.id], y2[,gen.id], y3[,gen.id], y4[,gen.id], y5[,gen.id],
            y6[,gen.id], y7[,gen.id], y8[,gen.id],
            names = c(name1, name2, name3, name4, name5, name6, name7, name8),
            col = c("#10d2f0", "#bda2e5"),
            ylim = c(0,20))
    title(main = sym, ylab = "log2(CPM)", xlab = "")
    legend("topright", legend = c("NonT2D", "T2D"), text.col = c("#bda2e5", "#10d2f0"))
}

dev.off()
```

Session Information

```
suppressPackageStartupMessages(library(Biobase))
suppressPackageStartupMessages(library(RColorBrewer))
suppressPackageStartupMessages(library(edgeR))
suppressPackageStartupMessages(library(sm))
library(edgeR)
library(Biobase)
library(RColorBrewer)
library(sm)
sessionInfo()
```

```
## R version 3.3.0 (2016-05-03)
## Platform: x86_64-apple-darwin13.4.0 (64-bit)
## Running under: OS X 10.11.3 (El Capitan)
```

```
##
## locale:
## [1] en_US.UTF-8/en_US.UTF-8/en_US.UTF-8/C/en_US.UTF-8/en_US.UTF-8
##
## attached base packages:
## [1] parallel  stats     graphics  grDevices utils     datasets  methods
## [8] base
##
## other attached packages:
## [1] sm_2.2-5.4          edgeR_3.14.0        limma_3.28.7
## [4] RColorBrewer_1.1-2  Biobase_2.32.0      BiocGenerics_0.18.0
##
## loaded via a namespace (and not attached):
##  [1] Rcpp_0.12.5     digest_0.6.9    formatR_1.4     magrittr_1.5
##  [5] evaluate_0.9    stringi_1.1.1   rmarkdown_0.9.6 tools_3.3.0
##  [9] stringr_1.0.0   yaml_2.1.13     htmltools_0.3.5 knitr_1.13
```
