# Supplementary material for: Single-cell transcriptomes identify human islet cell signatures and reveal cell-type–specific expression changes in type 2 diabetes
Source: Genome Res. 2017 Feb;27(2):208–22. doi: 10.1101/gr.212720.116 (PMC5287227; doi:10.1101/gr.212720.116)
Supplement: Supplemental Material [file supp_gr.212720.116_Supplemental_Methods_Source_Code.zip › Supplemental_Methods_Source_Code/Figure5_Source_Code/Figure_5F_Source_Code.pdf]

Cell Type Specific Gene Expression Differences Are Masked by Islet Cellular Heterogeneity

Introduction

We compared the transcriptional differences between T2D and ND endocrine cells without first segregating them into islet cell types (334 ND and 212 T2D single cell profiles). Approximately 2/3 of beta (n=165/248), one half of alpha (n=67/138), and over 90% of delta (n=23/24) cell-specific changes in gene expression were missed when cell types were not defined and specifically compared.The decreased heterogeneity in the transcriptional profiles of cell type-specific comparisons provides increased power in detecting the transcriptomic differences and validates the importance of single-cell analysis in understanding the molecular basis of T2D.

```
rm(list=ls())
# Load  in libraries
suppressPackageStartupMessages(library(d3vennR))
suppressPackageStartupMessages(library(venneuler))
library(d3vennR)
library(venneuler)
rm(list=ls())

# Load in differential lists
setwd("/Users/lawlon/Documents/Final_RNA_Seq_3/Differential_Expression_3/Single_Cell/T2D_vs_NonT2D_3/Si

beta <- read.csv("EdgeR.Robust.T2D.vs.NonT2D.Gender.Covariate.Beta.FDR.0.05.csv",
                 header = T, check.names = F, row.names = 1)
alpha <- read.csv("EdgeR.Robust.T2D.vs.NonT2D.Gender.Covariate.Alpha.FDR.0.05.csv",
                  header = T, check.names = F, row.names = 1)
delta <- read.csv("EdgeR.Robust.T2D.vs.NonT2D.Gender.Covariate.Delta.FDR.0.05.csv",
                  header = T, check.names = F, row.names = 1)
recon_endo <- read.csv("/Users/lawlon/Documents/Final_RNA_Seq_3/Differential_Expression_3/Single_Cell/T
                       header = T, check.names = F, row.names = 1)

# Perform intersections of DE genes with reconst pancreas and islets
int.b.i <- intersect(beta$Associated.Gene.Name, recon_endo$Associated.Gene.Name)
int.a.i <- intersect(alpha$Associated.Gene.Name, recon_endo$Associated.Gene.Name)
int.d.i <- intersect(delta$Associated.Gene.Name, recon_endo$Associated.Gene.Name)

# Intersection between alpha and beta
int.b.a <- intersect(beta$Associated.Gene.Name, alpha$Associated.Gene.Name)
int.b.d <- intersect(beta$Associated.Gene.Name, delta$Associated.Gene.Name)
int.a.d <- intersect(alpha$Associated.Gene.Name, delta$Associated.Gene.Name)

# intersection between three groups
int.b.a.i. <- intersect(int.b.i, alpha$Associated.Gene.Name)
int.b.d.i <- intersect(int.b.i, delta$Associated.Gene.Name)

# create venn diagram
venn <- venneuler(c(Beta = dim(beta)[1], Alpha = dim(alpha)[1], Delta = 50,
                    Reconstituted_Islet = dim(recon_endo)[1],
                    "Beta&Reconstituted_Islet" = length(int.b.i),
```

```r
                    "Alpha&Reconstituted_Islet" = length(int.a.i),
                    "Delta&Reconstituted_Islet" = length(int.d.i),
                    "Beta&Alpha"=length(int.b.a), "Beta&Delta"=length(int.b.d)))
# venn diagram labels
venn$labels <- c(
  paste("Beta\n", dim(beta)[1]),
  paste("Alpha\n", dim(alpha)[1]),
  paste("Delta\n", dim(delta)[1]),
  paste("Islet Single Cell Ensemble \n", dim(recon_endo)[1])
)

# Plot venn diagram
plot(venn, col = c("#e41a1c", "#377eb8", "#4daf4a", "#654321"), cex = 1)
```

Session Information

```r
suppressPackageStartupMessages(library(d3vennR))
suppressPackageStartupMessages(library(venneuler))
library(d3vennR)
library(venneuler)
sessionInfo()
```

```
## R version 3.3.0 (2016-05-03)
## Platform: x86_64-apple-darwin13.4.0 (64-bit)
## Running under: OS X 10.11.6 (El Capitan)
##
## locale:
## [1] en_US.UTF-8/en_US.UTF-8/en_US.UTF-8/C/en_US.UTF-8/en_US.UTF-8
##
## attached base packages:
## [1] stats     graphics  grDevices utils     datasets  methods   base
##
## other attached packages:
## [1] venneuler_1.1-0 rJava_0.9-8     d3vennR_0.1
##
## loaded via a namespace (and not attached):
##  [1] Rcpp_0.12.7     digest_0.6.10   assertthat_0.1  formatR_1.4
##  [5] magrittr_1.5    evaluate_0.10   stringi_1.1.2   rmarkdown_1.1
##  [9] tools_3.3.0     stringr_1.1.0   htmlwidgets_0.7 yaml_2.1.13
## [13] htmltools_0.3.5 knitr_1.14      tibble_1.2
```
