# Supplementary material for: Single-cell transcriptomes identify human islet cell signatures and reveal cell-type–specific expression changes in type 2 diabetes
Source: Genome Res. 2017 Feb;27(2):208–22. doi: 10.1101/gr.212720.116 (PMC5287227; doi:10.1101/gr.212720.116)
Supplement: Supplemental Material [file supp_gr.212720.116_Supplemental_Methods_Source_Code.zip › Supplemental_Methods_Source_Code/Supplemental_Figure_Source_Code/Supplemental_Fig_S10_Source_Code.pdf]

Unsupervised t-SNE of Type 2 diabetic Single Cell Ensemble Transcriptomes

Introduction

This file will detail the steps used to perform unsupervised t-SNE analysis on the Type 2 diabetic single cell transcriptomes.

t-SNE Analysis

```
suppressPackageStartupMessages(library(Biobase))
suppressPackageStartupMessages(library(edgeR))
suppressPackageStartupMessages(library(Rtsne))
suppressPackageStartupMessages(library(RColorBrewer))
library(Biobase)
library(edgeR)
library(Rtsne)
library(RColorBrewer)
rm(list = ls())
set.seed(125342)
# Load in Single Cell RNA-seq data
setwd("/Users/lawlon/Documents/Final_RNA_Seq_3/Data/")
load("T2D.rdata")
# File name
name = "T2D.log2.cpm"
# Probe annotation data
p.anns <- as(featureData(cnts.eset), "data.frame")
# Sample annotation data
s.anns <- pData(cnts.eset)
# Remove multiples and keep all other groups
s.anns.sel <- s.anns[s.anns$cell.type %in% c("INS", "PPY", "GCG", "SST",
                        "COL1A1", "KRT19", "PRSS1", "none"),]
# Expression data
counts <- exprs(cnts.eset)
# Calculate cpm of data
cpm <- cpm(x = counts)
cpm.vals <- log2(cpm+1)
cpm.vals <- cpm.vals[,rownames(s.anns.sel)]
r.max <- apply(cpm.vals,1,max)
# Get about 2500 genes
cpm.sel <- cpm.vals[r.max > 10.5,]
# Transpose the matrix
cpm1 <- t(cpm.sel)
# Remove groups that are all zeros
df <- cpm1[,apply(cpm1, 2, var, na.rm=TRUE) != 0]
#Run tsne with defaults
rtsne_out <- Rtsne(as.matrix(df), dims = 2)
# Set rownames of matrix to tsne matrix
rownames(rtsne_out$Y) <- rownames(cpm1)
# Write tSNE matrix to file
```

```r
write.csv(rtsne_out$Y, file = paste(name, "tsne.matrix.data.2D.csv", sep="."))
# Color Schema
grey <- brewer.pal(n=9, name="Greys")
colorCodes <- c(INS="#e41a1c", GCG = "#377eb8", SST = "#4daf4a", PPY = "#984ea3",
                COL1A1 = grey[9], PRSS1 = grey[7], KRT19 = grey[5],
                none = grey[3])

namelist <- c("Beta", "Alpha", "Delta", "Gamma",
              "Stellate", "Acinar", "Ductal", "none")

# Shapes for 2D plot
type1 <- NULL
for (i in 1:length(s.anns.sel$cell.type)){
  if ((s.anns.sel$cell.type[i] %in% c("INS","GCG","SST","PPY")) == TRUE) {
    idx = 20
    type1 = c(type1, idx)
  } else {
    idx = 17
    type1 = c(type1, idx)
  }
}

# Match up cell type name with hormone type
cellnames = NULL
for (i in 1:length(s.anns.sel$cell.type)) {
  if (s.anns.sel$cell.type[i] %in% names(namelist) == TRUE) {
    cellnames = c(cellnames, as.character(namelist[s.anns.sel$cell.type[i]]))
  }
}

# Match up colors and hormone labels
cols = NULL
for (i in 1:length(s.anns.sel$cell.type)) {
  if ((s.anns.sel$cell.type[i] %in% names(colorCodes)) == TRUE) {
    cols <- c(cols, colorCodes[s.anns.sel$cell.type[i]])
  }
}

# Match up cell name with hormone name
# Have cell type name and color
for (i in 1:length(cols)) {
  if (names(cols)[i] %in% names(namelist) == TRUE) {
    names(cols)[i] <- namelist[names(cols)[i]]
  }
}

# Plot the t-sne in 2-D
pdf(file = paste(name, "tSNE.2D.plot.pdf", sep = "."))
plot(rtsne_out$Y[,1], rtsne_out$Y[,2], col = cols, pch = type1,
     xlab = "t-SNE 1", ylab = "t-SNE 2")
legend("topleft", legend = as.character(namelist),
  text.col = colorCodes, pch = c(20,20,20,20,17,17,17,17), col = colorCodes,
  cex = 1)
```

```r
dev.off()
```

Session Information

```r
suppressPackageStartupMessages(library(Biobase))
suppressPackageStartupMessages(library(edgeR))
```

```
## Warning: package 'limma' was built under R version 3.3.1
```

```r
suppressPackageStartupMessages(library(Rtsne))
suppressPackageStartupMessages(library(RColorBrewer))
library(Biobase)
library(edgeR)
library(Rtsne)
library(RColorBrewer)
sessionInfo()
```

```
## R version 3.3.0 (2016-05-03)
## Platform: x86_64-apple-darwin13.4.0 (64-bit)
## Running under: OS X 10.11.6 (El Capitan)
##
## locale:
## [1] en_US.UTF-8/en_US.UTF-8/en_US.UTF-8/C/en_US.UTF-8/en_US.UTF-8
##
## attached base packages:
## [1] parallel  stats     graphics  grDevices utils     datasets  methods
## [8] base
##
## other attached packages:
## [1] RColorBrewer_1.1-2  Rtsne_0.11          edgeR_3.14.0
## [4] limma_3.28.21       Biobase_2.32.0      BiocGenerics_0.18.0
##
## loaded via a namespace (and not attached):
##  [1] Rcpp_0.12.7     digest_0.6.10   assertthat_0.1  formatR_1.4
##  [5] magrittr_1.5    evaluate_0.10   stringi_1.1.2   rmarkdown_1.1
##  [9] tools_3.3.0     stringr_1.1.0   yaml_2.1.13     htmltools_0.3.5
## [13] knitr_1.14      tibble_1.2
```
