# Supplementary material for: Single-cell transcriptomes identify human islet cell signatures and reveal cell-type–specific expression changes in type 2 diabetes
Source: Genome Res. 2017 Feb;27(2):208–22. doi: 10.1101/gr.212720.116 (PMC5287227; doi:10.1101/gr.212720.116)
Supplement: Supplemental Material [file supp_gr.212720.116_Supplemental_Methods_Source_Code.zip › Supplemental_Methods_Source_Code/Supplemental_Figure_Source_Code/Supplemental_Fig_S12_Source_Code.pdf]

Unsupervised Hierarchical Clustering of Type 2 diabetic Single Cell Ensemble Transcriptomes

Introduction

This file will detail the steps used to perform unsupervised hierarchical clustering analysis of the Type 2 diabetic single cell transcriptomes.

Hierarchical Clustering

```
suppressPackageStartupMessages(library(Biobase))
suppressPackageStartupMessages(library(edgeR))
suppressPackageStartupMessages(library(ape))
suppressPackageStartupMessages(library(gplots))
suppressPackageStartupMessages(library(dendextend))
suppressPackageStartupMessages(library(RColorBrewer))
library(edgeR)
library(Biobase)
library(gplots)
library(dendextend)
library(ape)
library(RColorBrewer)
rm(list=ls())
set.seed(53079239)
# File name
fname = "T2D.log2cpm"
setwd("/Users/lawlon/Documents/Final_RNA_Seq_3/Data/")
load("T2D.rdata")
p.anns <- featureData(cnts.eset)
probe.anns <- as(p.anns,"data.frame")
T2D.anns <- pData(cnts.eset)
# Remove multiples and keep all other groups
T2D.sel <- T2D.anns[T2D.anns$cell.type %in% c("INS", "PPY", "GCG", "SST",
                              "COL1A1", "KRT19", "PRSS1", "none"),]
# Expression data
T2D.counts <- exprs(cnts.eset)
cpms <- cpm(x = T2D.counts)
data <- log2(cpms+1)
data <- data[,rownames(T2D.sel)]
# Combine sample anns and expression data
s.anns.sel <- T2D.sel
r.max <- apply(data,1,max)
# Use highly expressed genes
data.sel <- data[r.max > 10.5,]
T2D.data.sel<- data.sel[, rownames(T2D.sel)]

# Save a copy of the data
exp.sel <- T2D.data.sel
# Change column name labels to cell type
colnames(T2D.data.sel)[1:dim(T2D.sel)[1]] <- T2D.sel$cell.type
```

```r
p.res <- probe.anns[rownames(T2D.data.sel),]
# Combine probe anns with selected cpm values
T2D.data.sel.exp <- cbind(p.res,T2D.data.sel)
# Write genes used for clustering to file
write.csv(T2D.data.sel.exp, paste(fname, "genes_selected_for_cing.csv", sep = "."))

# Dendrogram of samples
d <- dist(t(T2D.data.sel))
hc.final <- hclust(d,method="ward.D2")

# Change hclust to dendrogram
dend1 <- as.dendrogram(hc.final)
groupCodes <- s.anns.sel$cell.type

# Color Schema
grey <- brewer.pal(n=9, name="Greys")
colorCodes <- c(INS="#e41a1c", GCG = "#377eb8", SST = "#4daf4a", PPY = "#984ea3",
                COL1A1 = grey[9], PRSS1 = grey[7], KRT19 = grey[5],
                none = grey[3])

namelist <- c("Beta", "Alpha", "Delta", "Gamma",
              "Stellate", "Acinar", "Ductal", "none")

labels_colors(dend1) <- colorCodes[groupCodes][order.dendrogram(dend1)]

# Change dend to phylo object
dend2 <- as.phylo(dend1)

# Match up colors and labels
cols = NULL
for (i in 1:length(labels(dend2))) {
  if ((dend2$tip.label[i] %in% names(colorCodes)) == TRUE) {
    cols <- c(cols, colorCodes[dend2$tip.label[i]])
  }
}

#Use the long hyphen or the minus sign instead of regular hyphen symbol
labels(dend2) <- rep(x = "-", length(labels(dend2)))

# Create high resolution tiff of dendrogram
tiff(file=paste(fname, "dendrogram.no.legend.tiff", sep = "."),
    width = 9000, height = 9000, units = "px", res = 800)

plot(dend2, type = "fan", tip.color = cols, cex = 10.5, label.offset = 0)
legend("bottomleft", title = "Cell Types", title.col = "black",
       legend = c(expression(bold("Beta (INS)")), expression(bold("Alpha (GCG)")),
                  expression(bold("Delta (SST)")), expression(bold("Gamma (PPY)")),
                  expression(bold("Stellate (COL1A1)")), expression(bold("Acinar (PRSS1)")),
                  expression(bold("Ductal (KRT19)")), expression(bold("None"))), text.col = colorCodes,
       cex = 0.75, xjust=0, yjust=0)
dev.off()
```

Session Information

```r
suppressPackageStartupMessages(library(Biobase))
suppressPackageStartupMessages(library(edgeR))
```

```
## Warning: package 'limma' was built under R version 3.3.1
```

```r
suppressPackageStartupMessages(library(ape))
suppressPackageStartupMessages(library(gplots))
suppressPackageStartupMessages(library(dendextend))
suppressPackageStartupMessages(library(RColorBrewer))
library(edgeR)
library(Biobase)
library(gplots)
library(dendextend)
library(ape)
library(RColorBrewer)
sessionInfo()
```

```
## R version 3.3.0 (2016-05-03)
## Platform: x86_64-apple-darwin13.4.0 (64-bit)
## Running under: OS X 10.11.6 (El Capitan)
##
## locale:
## [1] en_US.UTF-8/en_US.UTF-8/en_US.UTF-8/C/en_US.UTF-8/en_US.UTF-8
##
## attached base packages:
## [1] parallel  stats     graphics  grDevices utils     datasets  methods
## [8] base
##
## other attached packages:
## [1] RColorBrewer_1.1-2  dendextend_1.3.0    gplots_3.0.1
## [4] ape_3.5             edgeR_3.14.0        limma_3.28.21
## [7] Biobase_2.32.0      BiocGenerics_0.18.0
##
## loaded via a namespace (and not attached):
##  [1] Rcpp_0.12.7        DEoptimR_1.0-6     formatR_1.4
##  [4] plyr_1.8.4         class_7.3-14       bitops_1.0-6
##  [7] tools_3.3.0        prabclus_2.2-6     digest_0.6.10
## [10] mclust_5.2         evaluate_0.10      tibble_1.2
## [13] nlme_3.1-128       gtable_0.2.0       lattice_0.20-34
## [16] yaml_2.1.13        mvtnorm_1.0-5      trimcluster_0.1-2
## [19] stringr_1.1.0      knitr_1.14         cluster_2.0.5
## [22] gtools_3.5.0       caTools_1.17.1     fpc_2.1-10
## [25] diptest_0.75-7     stats4_3.3.0       grid_3.3.0
## [28] nnet_7.3-12        robustbase_0.92-6  flexmix_2.3-13
## [31] rmarkdown_1.1      gdata_2.17.0       kernlab_0.9-25
## [34] ggplot2_2.1.0      magrittr_1.5       whisker_0.3-2
## [37] scales_0.4.0       htmltools_0.3.5    modeltools_0.2-21
## [40] MASS_7.3-45        assertthat_0.1     colorspace_1.2-7
## [43] KernSmooth_2.23-15 stringi_1.1.2      munsell_0.4.3
```
