# Supplementary material for: Single-cell transcriptomes identify human islet cell signatures and reveal cell-type–specific expression changes in type 2 diabetes
Source: Genome Res. 2017 Feb;27(2):208–22. doi: 10.1101/gr.212720.116 (PMC5287227; doi:10.1101/gr.212720.116)
Supplement: Supplemental Material [file supp_gr.212720.116_Supplemental_Methods_Source_Code.zip › Supplemental_Methods_Source_Code/Supplemental_Figure_Source_Code/Supplemental_Fig_S15_Source_Code.pdf]

Intersection of Reported Alpha and Beta Specific Gene Lists

Introduction

This file will detail the steps used to determine the intersection of several previously reported beta and alpha specific genes from Dorrell et al., 2011, Nica et al., 2013, Bramswig et al., 2013, and Blodgett et al., 2015.

Intersection of Reported Beta Specific Gene Lists

```
suppressPackageStartupMessages(library(Biobase))
suppressPackageStartupMessages(library(VennDiagram))
library(Biobase)
library(VennDiagram)
# Load in gene annotations
setwd("/Users/lawlon/Documents/Final_RNA_Seq/")
load("nonT2D.rdata")
p.anns <- as(featureData(cnts.eset),"data.frame")
# read in the gene lists
setwd("/Users/lawlon/Documents/Final_RNA_Seq_2/Published_Gene_Lists_2/")
genelist <- read.csv("Dorrell.Bramswig.Nica.Blodgett.All.Beta.genes.csv",
                     header = TRUE, check.names = FALSE, row.names = NULL)
# File name
name <- "Dorrell.Bramswig.Nica.Blodgett.Reported.Beta.Gene.Overlaps"
# isolate by gene list
dorrell <- genelist[,1]
# find number of genes we have data for
dorr.sel <- p.anns[p.anns$Associated.Gene.Name %in% dorrell,5]
# nica list
nica <- genelist[,2]
nica.sel <- p.anns[p.anns$Associated.Gene.Name %in% nica,5]
#bramswig list
bramswig <- genelist[,3]
bram.sel <- p.anns[p.anns$Associated.Gene.Name %in% bramswig,5]
# Blodgett list
blodgett <- genelist[,4]
blod.sel <- p.anns[p.anns$Associated.Gene.Name %in% blodgett,5]
# intersections
n12 <- length(intersect(bram.sel, blod.sel))
n13 <- length(intersect(bram.sel, nica.sel))
n14 <- length(intersect(bram.sel, dorr.sel))
n23 <- length(intersect(blod.sel, nica.sel))
n24 <- length(intersect(blod.sel, dorr.sel))
n34 <- length(intersect(nica.sel, dorr.sel))
int12 <- intersect(bram.sel, blod.sel)
int123 <- intersect(int12, nica.sel)
int124 <- intersect(int12, dorr.sel)
int13 <- intersect(bram.sel, nica.sel)
int134 <- intersect(int13, dorr.sel)
int23 <- intersect(blod.sel, nica.sel)
```

```
int234 <- intersect(int23, dorr.sel)
int1234 <- intersect(bram.sel, int234)
# Venn diagram
grid.newpage()
pdf(file = paste(name, "venn.diagram.pdf", sep = "."))
draw.quad.venn(area1 = length(bram.sel), area2 = length(blod.sel),
               area3 = length(nica.sel), area4 = length(dorr.sel),
               n12 = n12, n13 = n13, n14 = n14, n23 = n23, n24 = n24,
               n34 = n34, n123 = length(int123), n124 = length(int124),
               n134 = length(int134), n234 = length(int234), n1234 = length(int1234),
               category = c("Bramswig", "Blodgett", "Nica", "Dorrell"),
               fill = c("blue", "red", "grey", "yellow"))
dev.off()
```

Intersection of Reported Alpha Specific Gene Lists

```
suppressPackageStartupMessages(library(Biobase))
suppressPackageStartupMessages(library(VennDiagram))
library(Biobase)
library(VennDiagram)
# Load in gene annotations
setwd("/Users/lawlon/Documents/Final_RNA_Seq/")
load("nonT2D.rdata")
p.anns <- as(featureData(cnts.eset),"data.frame")
# read in the gene lists
setwd("/Users/lawlon/Documents/Final_RNA_Seq_2/Published_Gene_Lists_2/")
genelist <- read.csv("Dorrell.Bramswig.Nica.Blodgett.All.Alpha.genes.csv",
                     header = TRUE, check.names = FALSE, row.names = NULL)
# File name
name <- "Dorrell.Bramswig.Nica.Blodgett.Reported.Alpha.Gene.Overlaps"
# isolate by gene list
dorrell <- genelist[,1]
# find number of genes we have data for
dorr.sel <- p.anns[p.anns$Associated.Gene.Name %in% dorrell,5]
# nica list
nica <- genelist[,2]
nica.sel <- p.anns[p.anns$Associated.Gene.Name %in% nica,5]
#bramswig list
bramswig <- genelist[,3]
bram.sel <- p.anns[p.anns$Associated.Gene.Name %in% bramswig,5]
# Blodgett list
blodgett <- genelist[,4]
blod.sel <- p.anns[p.anns$Associated.Gene.Name %in% blodgett,5]
# intersections
n12 <- length(intersect(bram.sel, blod.sel))
n13 <- length(intersect(bram.sel, nica.sel))
n14 <- length(intersect(bram.sel, dorr.sel))
n23 <- length(intersect(blod.sel, nica.sel))
n24 <- length(intersect(blod.sel, dorr.sel))
n34 <- length(intersect(nica.sel, dorr.sel))
int12 <- intersect(bram.sel, blod.sel)
int123 <- intersect(int12, nica.sel)
```

```r
int124 <- intersect(int12, dorr.sel)
int13 <- intersect(bram.sel, nica.sel)
int134 <- intersect(int13, dorr.sel)
int23 <- intersect(blod.sel, nica.sel)
int234 <- intersect(int23, dorr.sel)
int1234 <- intersect(bram.sel, int234)
# Venn diagram
grid.newpage()
pdf(file = paste(name, "venn.diagram.pdf", sep = "."))
draw.quad.venn(area1 = length(bram.sel), area2 = length(blod.sel),
               area3 = length(nica.sel), area4 = length(dorr.sel),
               n12 = n12, n13 = n13, n14 = n14, n23 = n23, n24 = n24,
               n34 = n34, n123 = length(int123), n124 = length(int124),
               n134 = length(int134), n234 = length(int234), n1234 = length(int1234),
               category = c("Bramswig", "Blodgett", "Nica", "Dorrell"),
               fill = c("blue", "red", "grey", "yellow"))
dev.off()
```

Session Information

```r
suppressPackageStartupMessages(library(Biobase))
suppressPackageStartupMessages(library(readxl))
suppressPackageStartupMessages(library(VennDiagram))
library(Biobase)
library(readxl)
library(VennDiagram)
sessionInfo()
```

```
## R version 3.3.0 (2016-05-03)
## Platform: x86_64-apple-darwin13.4.0 (64-bit)
## Running under: OS X 10.11.6 (El Capitan)
##
## locale:
## [1] en_US.UTF-8/en_US.UTF-8/en_US.UTF-8/C/en_US.UTF-8/en_US.UTF-8
##
## attached base packages:
## [1] grid      parallel  stats     graphics  grDevices utils     datasets
## [8] methods   base
##
## other attached packages:
## [1] VennDiagram_1.6.17  futile.logger_1.4.3 readxl_0.1.1.9000
## [4] Biobase_2.32.0      BiocGenerics_0.18.0
##
## loaded via a namespace (and not attached):
##  [1] Rcpp_0.12.7          digest_0.6.10        assertthat_0.1
##  [4] futile.options_1.0.0 formatR_1.4          magrittr_1.5
##  [7] evaluate_0.10        stringi_1.1.2        rmarkdown_1.1
## [10] lambda.r_1.1.9       tools_3.3.0          stringr_1.1.0
## [13] yaml_2.1.13          htmltools_0.3.5      knitr_1.14
## [16] tibble_1.2
```
