# Supplementary material for: Single-cell transcriptomes identify human islet cell signatures and reveal cell-type–specific expression changes in type 2 diabetes
Source: Genome Res. 2017 Feb;27(2):208–22. doi: 10.1101/gr.212720.116 (PMC5287227; doi:10.1101/gr.212720.116)
Supplement: Supplemental Material [file supp_gr.212720.116_Supplemental_Methods_Source_Code.zip › Supplemental_Methods_Source_Code/Supplemental_Figure_Source_Code/Supplemental_Fig_S16_Source_Code.pdf]

Bulk Islet Expression of Islet Cell Type Specific Diabetes Associated GWAS and eQTL Genes

Introduction

This file will detail the steps used to show the expression of islet cell type specific diabetes associated GWAS and eQTL genes in non diabetic bulk intact islets. Ultimately, the average expression of cell type specific GWAS and eQTL genes were higher than that of the genes without cell type-specificity.

Boxplot of Bulk Islet Expression of Cell Type Specific Diabetes GWAS Genes

```
rm(list = ls())
# Examine avg log2 cpm expression of ANOVA and eQTL genes in non-diabetic bulk islets
library(edgeR)
library(Biobase)
setwd("/Users/lawlon/Documents/RNA-seq/RNA-seq Data/Bulk Islet Data/")
load("islet_bulk_uniq_data.rdata")
# Load in counts
bulk.counts <- exprs(bulk.cnts)
# Calculate cpm of data
cpms <- cpm(x = bulk.counts)
log.cpm <- log2(cpms+1)
# Gene and sample anns
p.anns <- as(featureData(bulk.cnts), "data.frame")
s.anns <- pData(bulk.cnts)
# Obtain only non-diabetic intact islets
s.sel <- s.anns[s.anns$Phenotype == "ND" & s.anns$Type == "Intact",]
# Obtain selected log2cpm for these islets
cpm.sel <- log.cpm[, rownames(s.sel)]
# Calculate averages for all genes
cpm.avg <- rowMeans(cpm.sel)
# Load in list of called genes and list of genes
setwd("/Users/lawlon/Documents/Final_RNA_Seq_3/ANOVA_3/Reported_GWAS_Genes/")
# ANOVA genes
anova.genes <- read.csv("NonT2D.3.All.Disease.GWAS.logFC.filtered.adj.pval.expressed.genes.csv",
                        header = T, check.names = F, row.names = 1)
# Get ANOVA gene ensembl ids
anova.ensl <- NULL
for (i in 1:dim(anova.genes)[1]) {
  id1 <- which(p.anns$Associated.Gene.Name == rownames(anova.genes)[i])
  anova.ids <- rownames(p.anns)[id1]
  anova.ensl <- c(anova.ensl, anova.ids)
}

# Get avg exp values for anova genes
anova.avg <- cpm.avg[anova.ensl]

#  lowly expressed genes
undet <- read.csv("NonT2D.3.All.T2D.and.T1D.GWAS.sub.expression.threshold.genes.csv",
                  header = T, check.names = F, row.names = 1)
```

```r
undet.genes <- undet[,1]
undet.genes <- as.character(undet.genes)
# Use unique genes
undet.genes <- unique(undet.genes)

# Get  ensembl ids
undet.ensl <- NULL
for (i in 1:length(undet.genes)) {
  id2 <- which(p.anns$Associated.Gene.Name == undet.genes[i])
  if (length(id2) > 1) {
    id2 <- id2[1]
    undet.ids <- rownames(p.anns)[id2]
    undet.ensl <- c(undet.ensl, undet.ids)
  } else {
  undet.ids <- rownames(p.anns)[id2]
  undet.ensl <- c(undet.ensl, undet.ids)
  }
}

# Get avg exp values for undet genes
undet.avg <- cpm.avg[undet.ensl]

#  <4 fold change genes
thresh <- read.csv("NonT2D.3.All.T2D.and.T1D.GWAS.sub.fold.change.threshold.genes.csv",
                      header = T, check.names = F, row.names = 1)
thresh.genes <- thresh[,1]
thresh.genes <- as.character(thresh.genes)
# Use unique genes
thresh.genes <- unique(thresh.genes)

# Get  ensembl ids
thresh.ensl <- NULL
for (i in 1:length(thresh.genes)) {
  id2 <- which(p.anns$Associated.Gene.Name == thresh.genes[i])
  if (length(id2) > 1) {
    id2 <- id2[1]
    thresh.ids <- rownames(p.anns)[id2]
    thresh.ensl <- c(thresh.ensl, thresh.ids)
  } else {
    thresh.ids <- rownames(p.anns)[id2]
    thresh.ensl <- c(thresh.ensl, thresh.ids)
  }
}

# Get avg exp values for thresh genes
thresh.avg <- cpm.avg[thresh.ensl]

# Get unique pan genes
pan <- read.csv("NonT2D.3.All.T2D.and.T1D.GWAS.Pan.islet.genes.csv",
                header = T, check.names = F, row.names = 1)
pan.genes <- pan[,1]
pan.genes <- as.character(pan.genes)
# Use unique genes
```

```r
pan.genes <- unique(pan.genes)

# Get pan gene ensembl ids
pan.ensl <- NULL
for (i in 1:length(pan.genes)) {
  id5 <- which(p.anns$Associated.Gene.Name == pan.genes[i])
  if (length(id5) > 1) {
    id5 <- id5[1]
    pan.ids <- rownames(p.anns)[id5]
    pan.ensl <- c(pan.ensl, pan.ids)
  } else {
    pan.ids <- rownames(p.anns)[id5]
    pan.ensl <- c(pan.ensl, pan.ids)
  }
}

# Get avg exp values for pan genes
pan.avg <- cpm.avg[pan.ensl]

# Combine cell specific and pan genes
cell.and.pan <- c(anova.avg, pan.avg)

# Make three box plots (cell specific, pan islet, low exprs)
setwd("/Users/lawlon/Documents/Final_RNA_Seq_3/ANOVA_3/Bulk_Expression_GWAS_and_EQTL/")
pdf(file = "T2D.and.T1D.GWAS.in.ND.Bulk.Intact.Islets.boxplots.pdf")
boxplot(undet.avg, thresh.avg, cell.and.pan,
        names = c(paste("\nLowly Expressed,\n n = ", length(undet.genes), sep =""),
                  paste("\n< 4-Fold Change,\n n = ", length(thresh.genes), sep =""),
                  paste("\nCell Specific and\n Pan-Islet, n = ", length(cell.and.pan), sep = "")),
        ylim = c(0,20))
title(main = "T1D/T2D Associated Gene Expression in NonT2D Bulk Intact Islets",
      ylab = "Average log2(CPM) Bulk Intact", xlab = "")
dev.off()
```

Boxplot of Bulk Islet Expression of Cell Type Specific Diabetes eQTL Genes

```r
rm(list = ls())
# Examine avg log2 cpm expression of ANOVA and  eQTL genes in non-diabetic bulk islets
library(edgeR)
library(Biobase)
setwd("/Users/lawlon/Documents/RNA-seq/RNA-seq Data/Bulk Islet Data/")
load("islet_bulk_uniq_data.rdata")
# Load in counts
bulk.counts <- exprs(bulk.cnts)
# Calculate cpm of data
cpms <- cpm(x = bulk.counts)
log.cpm <- log2(cpms+1)
# Gene and sample anns
p.anns <- as(featureData(bulk.cnts), "data.frame")
s.anns <- pData(bulk.cnts)
# Obtain only non-diabetic intact islets
s.sel <- s.anns[s.anns$Phenotype == "ND" & s.anns$Type == "Intact",]
```

```r
# Obtain selected log2cpm for these islets
cpm.sel <- log.cpm[, rownames(s.sel)]
# Calculate averages for all genes
cpm.avg <- rowMeans(cpm.sel)
# Load in list of called genes and list of  genes
setwd("/Users/lawlon/Documents/Final_RNA_Seq_3/ANOVA_3/eQTL/")
# ANOVA genes
anova.genes <- read.csv("NonT2D.3.eQTL.logFC.filtered.adj.pval.expressed.genes.csv",
                        header = T, check.names = F, row.names = 1)
# Get ANOVA gene ensembl ids
anova.ensl <- NULL
for (i in 1:dim(anova.genes)[1]) {
  id1 <- which(p.anns$Associated.Gene.Name == rownames(anova.genes)[i])
  anova.ids <- rownames(p.anns)[id1]
  anova.ensl <- c(anova.ensl, anova.ids)
}

# Get avg exp values for anova genes
anova.avg <- cpm.avg[anova.ensl]

#  genes (lowly expressed genes)
undet <- read.csv("NonT2D.eQTL.sub.expression.threshold.genes.csv",
                  header = T, check.names = F, row.names = 1)
undet.genes <- undet[,1]
undet.genes <- as.character(undet.genes)

# Get  lowly expressed gene ensembl ids
undet.ensl <- NULL
for (i in 1:length(undet.genes)) {
  id2 <- which(p.anns$Associated.Gene.Name == undet.genes[i])
  if (length(id2) > 1) {
    id2 <- id2[1]
    undet.ids <- rownames(p.anns)[id2]
    undet.ensl <- c(undet.ensl, undet.ids)
  } else {
  undet.ids <- rownames(p.anns)[id2]
  undet.ensl <- c(undet.ensl, undet.ids)
  }
}

# Get avg exp values for undet genes
undet.avg <- cpm.avg[undet.ensl]

#  genes below fold change enrichment threshold
thresh <- read.csv("NonT2D.eQTL.sub.fold.change.threshold.genes.csv",
                   header = T, check.names = F, row.names = 1)
thresh.genes <- thresh[,1]
thresh.genes <- as.character(thresh.genes)

# Get  sub fold change gene ensembl ids
thresh.ensl <- NULL
for (i in 1:length(thresh.genes)) {
  idx <- which(p.anns$Associated.Gene.Name == thresh.genes[i])
```

```r
  if (length(idx) > 1) {
    idx <- idx[1]
    thresh.ids <- rownames(p.anns)[idx]
    thresh.ensl <- c(thresh.ensl, thresh.ids)
  } else {
    thresh.ids <- rownames(p.anns)[idx]
    thresh.ensl <- c(thresh.ensl, thresh.ids)
  }
}

# Get avg exp values for thresh genes
thresh.avg <- cpm.avg[thresh.ensl]

# Read in pan-islet genes
pan <- read.csv("NonT2D.3.eQTL.Pan.islet.genes.csv",
                header = T, check.names = F, row.names = 1)
pan.genes <- pan[,1]
pan.genes <- as.character(pan.genes)

## Get  ensembl ids
pan.ensl <- NULL
for (i in 1:length(pan.genes)) {
  id3 <- which(p.anns$Associated.Gene.Name == pan.genes[i])
  if (length(id3) > 1) {
    id3 <- id2[1]
    pan.ids <- rownames(p.anns)[id3]
    pan.ensl <- c(pan.ensl, pan.ids)
  } else {
    pan.ids <- rownames(p.anns)[id3]
    pan.ensl <- c(pan.ensl, pan.ids)
  }
}

# Get avg exp values for undet genes
pan.avg <- cpm.avg[pan.ensl]

# Combine cell specific and pan genes
cell.and.pan <- c(anova.avg, pan.avg)

setwd("/Users/lawlon/Documents/Final_RNA_Seq_3/ANOVA_3/Bulk_Expression_GWAS_and_EQTL/")
# Make three box plots (cell specific, pan islet, low exprs)
pdf(file = "eQTL.Genes.in.ND.Bulk.Intact.Islets.boxplots.pdf")
boxplot(undet.avg, thresh.avg, cell.and.pan,
        names = c(paste("\nLowly Expressed,\n n = ", length(undet.genes), sep =""),
                paste("\n< 4-Fold Change,\n n = ", length(thresh.genes), sep =""),
                paste("\nCell Specific and\n Pan-Islet, n = ", length(cell.and.pan), sep = "")),
        ylim = c(0,20))
title(main = "eQTL Target Gene Expression in NonT2D Bulk Intact Islets",
      ylab = "Average log2(CPM) Bulk Intact", xlab = "")
dev.off()
```

Session Information

```r
suppressPackageStartupMessages(library(Biobase))
suppressPackageStartupMessages(library(edgeR))
library(Biobase)
library(edgeR)
sessionInfo()
```

```
## R version 3.3.0 (2016-05-03)
## Platform: x86_64-apple-darwin13.4.0 (64-bit)
## Running under: OS X 10.11.3 (El Capitan)
##
## locale:
## [1] en_US.UTF-8/en_US.UTF-8/en_US.UTF-8/C/en_US.UTF-8/en_US.UTF-8
##
## attached base packages:
## [1] parallel  stats     graphics  grDevices utils     datasets  methods
## [8] base
##
## other attached packages:
## [1] edgeR_3.14.0        limma_3.28.11       Biobase_2.32.0
## [4] BiocGenerics_0.18.0
##
## loaded via a namespace (and not attached):
##  [1] magrittr_1.5    formatR_1.4     tools_3.3.0     htmltools_0.3.5
##  [5] yaml_2.1.13     Rcpp_0.12.5     stringi_1.1.1   rmarkdown_0.9.6
##  [9] knitr_1.13      stringr_1.0.0   digest_0.6.9    evaluate_0.9
```
