# Supplementary material for: Single-cell transcriptomes identify human islet cell signatures and reveal cell-type–specific expression changes in type 2 diabetes
Source: Genome Res. 2017 Feb;27(2):208–22. doi: 10.1101/gr.212720.116 (PMC5287227; doi:10.1101/gr.212720.116)
Supplement: Supplemental Material [file supp_gr.212720.116_Supplemental_Methods_Source_Code.zip › Supplemental_Methods_Source_Code/Supplemental_Figure_Source_Code/Supplemental_Fig_S18_Source_Code.pdf]

3-Dimensional t-SNE Analysis of Non-diabetic and Type 2 Diabetic Single Cell Ensemble Transcriptomes

Introduction

This file will detail the steps used to perform unsupervised t-SNE analysis on the non-diabetic and type 2 diabetic single cell transcriptomes. The data was reduced to three dimensions in order to produce a 3D gif animation.

t-SNE Analysis

```
suppressPackageStartupMessages(library(Biobase))
suppressPackageStartupMessages(library(edgeR))
suppressPackageStartupMessages(library(Rtsne))
suppressPackageStartupMessages(library(scatterplot3d))
suppressPackageStartupMessages(library(rgl))
suppressPackageStartupMessages(library(pca3d))
suppressPackageStartupMessages(library(RColorBrewer))
library(Biobase)
library(edgeR)
library(Rtsne)
library(scatterplot3d)
library(rgl)
library(pca3d)
library(RColorBrewer)
rm(list = ls())
set.seed(123345)
# Set working directory
setwd("/Users/lawlon/Documents/Final_RNA_Seq_3/Data/")
# File name
name = "NonT2D.and.T2D.CPM.by.disease"
# Load in NonT2D single cell data
load("nonT2D.rdata")
p.anns <- featureData(cnts.eset)
probe.anns <- as(p.anns,"data.frame")
ND.anns <- pData(cnts.eset)
# Remove multiples and keep all other groups
ND.sel <- ND.anns[ND.anns$cell.type %in% c("INS", "PPY", "GCG", "SST",
                "COL1A1", "KRT19", "PRSS1", "none"),]
# Calculate cpm
ND.counts <- exprs(cnts.eset)
ND.cpms <- cpm(x = ND.counts)
ND.cpm <- log2(ND.cpms+1)
ND.cpm.sel <- ND.cpm[, rownames(ND.sel)]
# Load in T2D single cell data
load("T2D.rdata")
T2D.anns <- pData(cnts.eset)
# Remove multiples and keep all other groups
T2D.sel <- T2D.anns[T2D.anns$cell.type %in% c("INS", "PPY", "GCG", "SST",
                 "COL1A1", "KRT19", "PRSS1", "none"),]
```

```r
# Calculate cpm
T2D.counts <- exprs(cnts.eset)
T2D.cpms <- cpm(x = T2D.counts)
T2D.cpm <- log2(T2D.cpms+1)
T2D.cpm.sel <- T2D.cpm[, rownames(T2D.sel)]
# Combine sample anns and expression data
cpm.vals <- cbind(ND.cpm.sel, T2D.cpm.sel)
s.anns.sel <- rbind(ND.sel, T2D.sel)
# Identify the epsilon cell (high expression of GHRL)
g <- which(probe.anns$Associated.Gene.Name == "GHRL")
ghrl <- cpm.vals[g,]
samp <- which(ghrl > 15)
g.idx <- which(rownames(s.anns.sel) == names(samp))
# Change sample anns of cell to GHRL
s.anns.sel$cell.type[g.idx] <- "GHRL"
cpm.vals <- cpm.vals[, rownames(s.anns.sel)]
r.max <- apply(cpm.vals,1,max)
# Use highly expressed genes
cpm.sel <- cpm.vals[r.max > 10.5,]
# transpose the matrix
cpm1 <- t(cpm.sel)
# Remove groups that are all zeros
df <- cpm1[,apply(cpm1, 2, var, na.rm=TRUE) != 0]
# t-SNE ANALYSIS
rtsne_out <- Rtsne(as.matrix(df), dims = 3)
# Set rownames of matrix to tsne matrix
rownames(rtsne_out$Y) <- rownames(cpm1)
# write the tsne matrix to file
write.csv(rtsne_out$Y, file = paste(name, "tsne.matrix.data.3D.csv", sep="."))
# Specify the phenotype of each single cell sample for plot
group <- c(rep("NonT2D", dim(ND.sel)[1]), rep("T2D", dim(T2D.sel)[1]))
# Color Schema
grey <- brewer.pal(n=9, name="Greys")
# Color codes for each specific cell type
colorCodes <- c(INS="#e41a1c", GCG = "#377eb8", SST = "#4daf4a",
                PPY = "#984ea3", GHRL = "#ff7f00",
                COL1A1 = grey[9], PRSS1 = grey[7], KRT19 = grey[5],
                none = grey[3], NonT2D = "black", T2D = "black")

namelist <- c("Beta", "Alpha", "Delta", "Gamma",
              "Epsilon", "Stellate", "Acinar", "Ductal", "none")
# Make variable of T2D and NonT2D for 3D plot
type = NULL
for (i in 1:length(group)) {
  if (group[i] %in% c("NonT2D") == TRUE) {
    idx = "sphere"
    type = c(type, idx)
  } else {
    idx = "tetrahedron"
    type = c(type, idx)
  }
}
# Specify the shape for T2D and NonT2D
```

```
type1 <- NULL
for (i in 1:length(group)){
  if ((group[i] %in% c("NonT2D")) == TRUE) {
    idx = 20
    type1 = c(type1, idx)
  } else {
    idx = 17
    type1 = c(type1, idx)
  }
}
# Match up colors and hormone labels
cols = NULL
for (i in 1:length(s.anns.sel$cell.type)) {
  if ((s.anns.sel$cell.type[i] %in% names(colorCodes)) == TRUE) {
    cols <- c(cols, colorCodes[s.anns.sel$cell.type[i]])
  }
}
# Match up cell name with hormone name
# Have cell type name and color
for (i in 1:length(cols)) {
  if (names(cols)[i] %in% names(namelist) == TRUE) {
    names(cols)[i] <- namelist[names(cols)[i]]
  }
}
# Make movie
pca3d(rtsne_out$Y, components = c(1:3), col = cols, title=name,
      axe.titles = c("t-SNE 1", "t-SNE 2", "t-SNE 3"),
      legend = TRUE, new = TRUE, shape = type)
legend3d("topright", c("Beta (INS)", "Alpha (GCG)",
                       "Delta (SST)", "Gamma (PPY)", "Epsilon (GHRL)",
                       "Stellate (COL1A1)", "Acinar (PRSS1)",
                       "Ductal (KRT19)", "None", "NonT2D", "T2D"),
         text.col = colorCodes, col = colorCodes,
         pch = c(NA,NA,NA,NA,NA,NA,NA,NA,NA,20,17))

#Take picture of the 3d ploot
rgl.snapshot(filename = paste(name,"3D.PCA.plot.png", sep = "."), fmt = "png")

#Take pictures of multiple angles of the plot to create a gif
for (i in 1:360) {
  rgl.viewpoint(i, 20)
  filename <- paste(name, formatC(i, digits = 1, width = 3,
                                  flag = "0"), ".png", sep = "")
  rgl.snapshot(filename)
}

# Make a gif animation using ImageMagick
system(command = "convert -delay 10 *.png -loop 0 pic.gif")
```

Session Information

```
suppressPackageStartupMessages(library(Biobase))
suppressPackageStartupMessages(library(edgeR))
```

```
## Warning: package 'limma' was built under R version 3.3.1
```

```
suppressPackageStartupMessages(library(Rtsne))
suppressPackageStartupMessages(library(scatterplot3d))
suppressPackageStartupMessages(library(rgl))
suppressPackageStartupMessages(library(pca3d))
suppressPackageStartupMessages(library(RColorBrewer))
library(Biobase)
library(edgeR)
library(Rtsne)
library(scatterplot3d)
library(rgl)
library(pca3d)
library(RColorBrewer)
sessionInfo()
```

```
## R version 3.3.0 (2016-05-03)
## Platform: x86_64-apple-darwin13.4.0 (64-bit)
## Running under: OS X 10.11.6 (El Capitan)
##
## locale:
## [1] en_US.UTF-8/en_US.UTF-8/en_US.UTF-8/C/en_US.UTF-8/en_US.UTF-8
##
## attached base packages:
## [1] parallel  stats     graphics  grDevices utils     datasets  methods
## [8] base
##
## other attached packages:
## [1] RColorBrewer_1.1-2   pca3d_0.8            rgl_0.96.0
## [4] scatterplot3d_0.3-37 Rtsne_0.11           edgeR_3.14.0
## [7] limma_3.28.21        Biobase_2.32.0       BiocGenerics_0.18.0
##
## loaded via a namespace (and not attached):
##  [1] Rcpp_0.12.7     knitr_1.14      magrittr_1.5    ellipse_0.3-8
##  [5] xtable_1.8-2    R6_2.2.0        stringr_1.1.0   tools_3.3.0
##  [9] htmltools_0.3.5 yaml_2.1.13     assertthat_0.1  digest_0.6.10
## [13] tibble_1.2      shiny_0.14.1    formatR_1.4     htmlwidgets_0.7
## [17] evaluate_0.10   mime_0.5        rmarkdown_1.1   stringi_1.1.2
## [21] jsonlite_1.1    httpuv_1.3.3
```
