# Supplementary material for: Single-cell transcriptomes identify human islet cell signatures and reveal cell-type–specific expression changes in type 2 diabetes
Source: Genome Res. 2017 Feb;27(2):208–22. doi: 10.1101/gr.212720.116 (PMC5287227; doi:10.1101/gr.212720.116)
Supplement: Supplemental Material [file supp_gr.212720.116_Supplemental_Methods_Source_Code.zip › Supplemental_Methods_Source_Code/Supplemental_Figure_Source_Code/Supplemental_Fig_S1_Source_Code.pdf]

Correlations of Baseline, Intact, and Dissociated Bulk Islet Transcriptomes

Introduction

Islets with high purity and viability were obtained from 8 human cadaveric organ donors and cultured (Methods) overnight to recover from shipment (Table_S1; 5 non-diabetic (ND) and 3 type 2 diabetic (T2D)). For each islet sample, cDNA was prepared from single cells on the Fluidigm C1 chip and from paired whole islet (Baseline, Intact) and single cell suspension "bulk" samples (Dissociated) as shown in Fig. 1A using the SMARTer2 protocol. Total processing and handling time for each islet was less than or equal to 60 minutes. Ultimately, Baseline, Intact, and Dissociated transcriptomes from each patient were highly correlated.

Correlation of Bulk Transcriptomes within Each Patient

```
suppressPackageStartupMessages(library(Biobase))
suppressPackageStartupMessages(library(gplots))
suppressPackageStartupMessages(library(ggplot2))
library(Biobase)
library(gplots)
library(ggplot2)
rm(list=ls())
setwd("/Users/lawlon/Documents/RNA-seq/RNA-seq Data/Bulk Islet Data/")
load("islet_bulk_uniq_data.rdata")

s.anns <- read.csv(file = "Unique Bulk Data/sample_anns_unique_bulk_samples.csv",
                            header = TRUE, check.names = FALSE, row.names = 1)
bulk.counts <- exprs(bulk.cnts)
log.tpm <- log2(bulk.counts+1)

# Indicate patient name
name <- c("Patient.1", "Patient.2", "Patient.3", "Patient.4",
          "Patient.5", "Patient.6", "Patient.7", "Patient.8")
p.id <- c("P1", "P2", "P3", "P4", "P5", "P6", "P7", "P8")

# Start of loop
setwd("/Users/lawlon/Documents/Final_RNA_Seq_3/Correlations_3/Bulk_within_Patient_Correlations/")
pdf(file = "Patient.bulk.correlation.scatterplots.pdf", onefile = TRUE)
par(mfrow = c(5,5), pty = "s", cex.lab = 0.75, cex.axis=0.75, mgp = c(1.25,0.5,0), mar = c(2.5,2,0.5,0.[illegible]

for (i in 1:length(p.id)) {

# Specify patient number
pat1 <- which(s.anns$`Patient Number` == p.id[i])
p1.anns <- s.anns[pat1,]

# Plot log2 tpm of patient intact vs baseline
p1.in <- which(p1.anns$Type == "Intact")
p1.bl <- which(p1.anns$Type == "Baseline")
p1.d <- which(p1.anns$Type == "Dissociated")
```

```r
# Make a panel of plots, first intact vs baseline
plot(log.tpm[,rownames(p1.anns)[p1.in]], log.tpm[, rownames(p1.anns)[p1.bl]],
     xlab = paste(p.id[i], "IN log2(TPM)", sep = " "), ylab = paste(p.id[i], "BL log2(TPM)", sep = " ")
     xlim = c(0,20), ylim = c(0,20))

Rsq <- cor(log.tpm[,rownames(p1.anns)[p1.in]], log.tpm[, rownames(p1.anns)[p1.bl]])**2
msg <- paste("Rsq = ",format(Rsq,digits=2),sep="")
text(14,1, msg, cex = 0.75)

# Plot log2 tpm of pat1 intact vs diss
plot(log.tpm[,rownames(p1.anns)[p1.in]], log.tpm[, rownames(p1.anns)[p1.d]],
     xlab = paste(p.id[i], "IN log2(TPM)", sep = " "), ylab = paste(p.id[i], "DI log2(TPM)", sep = " ")
     xlim = c(0,20), ylim = c(0,20))

Rsq <- cor(log.tpm[,rownames(p1.anns)[p1.in]], log.tpm[, rownames(p1.anns)[p1.d]])**2
msg <- paste("Rsq = ",format(Rsq,digits=2),sep="")
text(14,1, msg, cex = 0.75)

# Plot log2 tpm of baseline vs diss
plot(log.tpm[,rownames(p1.anns)[p1.bl]], log.tpm[, rownames(p1.anns)[p1.d]],
     xlab = paste(p.id[i], "BL log2(TPM)", sep = " "), ylab = paste(p.id[i], "DI log2(TPM)", sep = " ")
     xlim = c(0,20), ylim = c(0,20))

Rsq <- cor(log.tpm[,rownames(p1.anns)[p1.bl]], log.tpm[, rownames(p1.anns)[p1.d]])**2
msg <- paste("Rsq = ",format(Rsq,digits=2),sep="")
text(14,1, msg, cex = 0.75)

}

dev.off()
```

Session Information

```r
suppressPackageStartupMessages(library(Biobase))
suppressPackageStartupMessages(library(gplots))
suppressPackageStartupMessages(library(ggplot2))
rm(list = ls())
library(Biobase)
library(gplots)
library(ggplot2)
sessionInfo()
```

```
## R version 3.3.0 (2016-05-03)
## Platform: x86_64-apple-darwin13.4.0 (64-bit)
## Running under: OS X 10.11.3 (El Capitan)
##
## locale:
## [1] en_US.UTF-8/en_US.UTF-8/en_US.UTF-8/C/en_US.UTF-8/en_US.UTF-8
##
## attached base packages:
## [1] parallel  stats     graphics  grDevices utils     datasets  methods
## [8] base
```

```
##
## other attached packages:
## [1] ggplot2_2.1.0       gplots_3.0.1        Biobase_2.32.0
## [4] BiocGenerics_0.18.0
##
## loaded via a namespace (and not attached):
##  [1] Rcpp_0.12.5        knitr_1.13         magrittr_1.5
##  [4] munsell_0.4.3      colorspace_1.2-6   stringr_1.0.0
##  [7] plyr_1.8.4         caTools_1.17.1     tools_3.3.0
## [10] grid_3.3.0         gtable_0.2.0       KernSmooth_2.23-15
## [13] htmltools_0.3.5    gtools_3.5.0       yaml_2.1.13
## [16] digest_0.6.9       formatR_1.4        bitops_1.0-6
## [19] evaluate_0.9       rmarkdown_0.9.6    gdata_2.17.0
## [22] stringi_1.1.1      scales_0.4.0
```
