# Supplementary material for: Single-cell transcriptomes identify human islet cell signatures and reveal cell-type–specific expression changes in type 2 diabetes
Source: Genome Res. 2017 Feb;27(2):208–22. doi: 10.1101/gr.212720.116 (PMC5287227; doi:10.1101/gr.212720.116)
Supplement: Supplemental Material [file supp_gr.212720.116_Supplemental_Methods_Source_Code.zip › Supplemental_Methods_Source_Code/Supplemental_Figure_Source_Code/Supplemental_Fig_S21_Source_Code.pdf]

Unsupervised Hierarchical Clustering of Non-diabetic and Type 2 Diabetic Single Cell Ensemble Transcriptomes With Patient Identifier Information

Introduction

This file will detail the steps used to perform unsupervised hierarchical clustering analysis on the non-diabetic and type 2 diabetic single cell transcriptomes while including the cell type, disease status, and patienti identifier labels for each sample.

Hierarchical Clustering

```
suppressPackageStartupMessages(library(Biobase))
suppressPackageStartupMessages(library(edgeR))
suppressPackageStartupMessages(library(ape))
suppressPackageStartupMessages(library(gplots))
suppressPackageStartupMessages(library(dendextend))
suppressPackageStartupMessages(library(RColorBrewer))
library(edgeR)
library(Biobase)
library(gplots)
library(dendextend)
library(ape)
library(RColorBrewer)
rm(list=ls())
set.seed(2135435)
# file name
fname = "NonT2D.and.T2D.log2cpm.by.disease.and.patient"
setwd("/Users/lawlon/Documents/Final_RNA_Seq_3/Data/")
# Load in NonT2D single cell data
load("nonT2D.rdata")
p.anns <- featureData(cnts.eset)
probe.anns <- as(p.anns,"data.frame")
ND.anns <- pData(cnts.eset)
# Remove multiples and keep all other groups
ND.sel <- ND.anns[ND.anns$cell.type %in% c("INS", "PPY", "GCG", "SST",
                  "COL1A1", "KRT19", "PRSS1", "none"),]
# Calculate cpm
ND.counts <- exprs(cnts.eset)
ND.cpms <- cpm(x = ND.counts)
ND.cpm <- log2(ND.cpms+1)
ND.cpm.sel <- ND.cpm[, rownames(ND.sel)]
# Load in T2D single cell data
load("T2D.rdata")
T2D.anns <- pData(cnts.eset)
# Remove multiples and keep all other groups
T2D.sel <- T2D.anns[T2D.anns$cell.type %in% c("INS", "PPY", "GCG", "SST",
                  "COL1A1", "KRT19", "PRSS1", "none"),]
```

```r
# Calculate cpm
T2D.counts <- exprs(cnts.eset)
T2D.cpms <- cpm(x = T2D.counts)
T2D.cpm <- log2(T2D.cpms+1)
T2D.cpm.sel <- T2D.cpm[, rownames(T2D.sel)]
# Combine sample anns and expression data
cpm.vals <- cbind(ND.cpm.sel, T2D.cpm.sel)
s.anns.sel <- rbind(ND.sel, T2D.sel)
r.max <- apply(cpm.vals,1,max)
# Use highly expressed genes
cpm.sel <- cpm.vals[r.max > 10.5,]
# Save a copy of data
cpm.res <- cpm.sel
# Change column name labels to cell type and disease state
colnames(cpm.res)[1:dim(ND.sel)[1]] <- paste(ND.sel$cell.type, "NonT2D", sep="-")
colnames(cpm.res)[(dim(ND.sel)[1]+1):dim(cpm.res)[2]] <- paste(T2D.sel$cell.type, "T2D", sep="-")
# Identify the ghrelin positive cell
g <- which(probe.anns$Associated.Gene.Name == "GHRL")
ghrl <- cpm.vals[g,]
samp <- which(ghrl > 15)
g.idx <- which(rownames(s.anns.sel) == names(samp))
colnames(cpm.res)[g.idx] <- "GHRL-NonT2D"
# Attach labels of patient number to data
  # Make a vector of patient number labels
i=1
pats <- NULL
for (i in 1:length(s.anns.sel$run)) {
  if (s.anns.sel$run[i] %in% c("1st", "2nd", "3rd")==TRUE) {
    colnames(cpm.res)[i] <- paste(colnames(cpm.res)[i], "P1", sep = "-")
    pats <- c(pats, "P1")
  } else if (s.anns.sel$run[i] %in% c("5th")==TRUE) {
    colnames(cpm.res)[i] <- paste(colnames(cpm.res)[i], "P2", sep = "-")
    pats <- c(pats, "P2")
  } else if (s.anns.sel$run[i] %in% c("6th", "7th")==TRUE) {
    colnames(cpm.res)[i] <- paste(colnames(cpm.res)[i], "P3", sep = "-")
    pats <- c(pats, "P3")
  } else if (s.anns.sel$run[i] %in% c("8th")==TRUE) {
    colnames(cpm.res)[i] <- paste(colnames(cpm.res)[i], "P4", sep = "-")
    pats <- c(pats, "P4")
  } else if (s.anns.sel$run[i] %in% c("9th")==TRUE) {
    colnames(cpm.res)[i] <- paste(colnames(cpm.res)[i], "P5", sep = "-")
    pats <- c(pats, "P5")
  } else if (s.anns.sel$run[i] %in% c("4th")==TRUE) {
    colnames(cpm.res)[i] <- paste(colnames(cpm.res)[i], "P6", sep = "-")
    pats <- c(pats, "P6")
  } else if (s.anns.sel$run[i] %in% c("10t", "11t")==TRUE) {
    colnames(cpm.res)[i] <- paste(colnames(cpm.res)[i], "P7", sep = "-")
    pats <- c(pats, "P7")
  } else if (s.anns.sel$run[i] %in% c("12t", "13t")==TRUE) {
    colnames(cpm.res)[i] <- paste(colnames(cpm.res)[i], "P8", sep = "-")
    pats <- c(pats, "P8")
  }
}
```

```
p.res <- probe.anns[rownames(cpm.res),]
# Combine probe anns with selected fpkm values
cpm.res.exp <- cbind(p.res,cpm.res)
# Output genes used for clustering to file
write.csv(cpm.res.exp,paste(fname, "genes_selected_for_cing.csv", sep = "."))
# Hclust object of samples
exp.sel <- cpm.res
d <- dist(t(exp.sel))
hc.final <- hclust(d,method="ward.D2")
# Change hclust to dendrogram
dend1 <- as.dendrogram(hc.final)
dendcol <- as.dendrogram(hc.final)
# Make another dend for patient color
dendpat <- as.dendrogram(hc.final)
# Color codes for cell type and disease state
groupCodes1 <- s.anns.sel$cell.type
groupCodes <- c(rep("NonT2D", dim(ND.sel)[1]), rep("T2D", dim(T2D.sel)[1]))
# Make a vector of patient numbers
grouppat <- pats

# Color Schema for cell type
grey <- brewer.pal(n=9, name="Greys")
colorCodes1 <- c(INS="#e41a1c", GCG = "#377eb8", SST = "#4daf4a",
                 PPY = "#984ea3", GHRL = "#ff7f00",
                 COL1A1 = grey[9], PRSS1 = grey[7], KRT19 = grey[5],
                 none = grey[3])
# Colors for disease phenotype
colorCodes <- c(NonT2D="#bda2e5", T2D = "#10d2f0")
# Color schema for patient number
colorpats <- c(P1 = "#7fc97f", P2 = "#beaed4", P3 = "#fdc086",
               P4 = "#ffff99", P5 = "#386cb0",
               P6 = "#f0027f", P7 = "#bf5b17", P8 = "#666666")

namelist <- c("Beta", "Alpha", "Delta", "Gamma",
              "Epsilon", "Stellate", "Acinar", "Ductal", "none")

labels_colors(dend1) <- colorCodes[groupCodes][order.dendrogram(dend1)]
labels_colors(dendcol) <- colorCodes1[groupCodes1][order.dendrogram(dendcol)]
labels_colors(dendpat) <- colorpats[grouppat][order.dendrogram(dendpat)]

# Change dend to phylo object
dend2 <- as.phylo(dend1)
dend3 <- as.phylo(dendcol)
dend4 <- as.phylo(dendpat)

# Match up colors and labels
cols = NULL
for (i in 1:length(labels(dend2))) {
  if (grepl(x = dend2$tip.label[i], pattern = "NonT2D") == TRUE) {
    cols <- c(cols, colorCodes["NonT2D"])
  } else if (grepl(x = dend2$tip.label[i], pattern = "T2D") == TRUE) {
    cols <- c(cols, colorCodes["T2D"])
  }
```

```r
}

# Match up cell type and color for dend3
col3 = NULL
for (i in 1:length(labels(dend3))) {
  if (grepl(x = dend3$tip.label[i], pattern = "INS") == TRUE) {
    col3 <- c(col3, colorCodes1["INS"])
  } else if (grepl(x = dend3$tip.label[i], pattern = "GCG") == TRUE) {
    col3 <- c(col3, colorCodes1["GCG"])
  } else if (grepl(x = dend3$tip.label[i], pattern = "SST") == TRUE) {
    col3 <- c(col3, colorCodes1["SST"])
  } else if (grepl(x = dend3$tip.label[i], pattern = "PPY") == TRUE) {
    col3 <- c(col3, colorCodes1["PPY"])
  } else if (grepl(x = dend3$tip.label[i], pattern = "COL1A1") == TRUE) {
    col3 <- c(col3, colorCodes1["COL1A1"])
  } else if (grepl(x = dend3$tip.label[i], pattern = "PRSS1") == TRUE) {
    col3 <- c(col3, colorCodes1["PRSS1"])
  } else if (grepl(x = dend3$tip.label[i], pattern = "KRT19") == TRUE) {
    col3 <- c(col3, colorCodes1["KRT19"])
  } else if (grepl(x = dend3$tip.label[i], pattern = "GHRL") == TRUE) {
    col3 <- c(col3, colorCodes1["GHRL"])
  } else if (grepl(x = dend3$tip.label[i], pattern = "none") == TRUE) {
    col3 <- c(col3, colorCodes1["none"])
  }
}

# Match up colors and labels for patient number dendrogram
col4 <- NULL
for (i in 1:length(labels(dend4))) {
  if (grepl(x = dend4$tip.label[i], pattern = "P1") == TRUE) {
    col4 <- c(col4, colorpats["P1"])
  } else if (grepl(x = dend4$tip.label[i], pattern = "P2") == TRUE) {
    col4 <- c(col4, colorpats["P2"])
  } else if (grepl(x = dend4$tip.label[i], pattern = "P3") == TRUE) {
    col4 <- c(col4, colorpats["P3"])
  } else if (grepl(x = dend4$tip.label[i], pattern = "P4") == TRUE) {
    col4 <- c(col4, colorpats["P4"])
  } else if (grepl(x = dend4$tip.label[i], pattern = "P5") == TRUE) {
    col4 <- c(col4, colorpats["P5"])
  } else if (grepl(x = dend4$tip.label[i], pattern = "P6") == TRUE) {
    col4 <- c(col4, colorpats["P6"])
  } else if (grepl(x = dend4$tip.label[i], pattern = "P7") == TRUE) {
    col4 <- c(col4, colorpats["P7"])
  } else if (grepl(x = dend4$tip.label[i], pattern = "P8") == TRUE) {
    col4 <- c(col4, colorpats["P8"])
  }
}
#Use the long hyphen or the minus sign instead of regular hyphen symbol
labels(dend2) <- rep(x = "−", length(labels(dend2)))
labels(dend3) <- rep(x = "−", length(labels(dend3)))
labels(dend4) <- rep(x = "−", length(labels(dend4)))

# Make a high resolution tiff image, file size is very large
```

```r
tiff(file= paste(fname, "dendrogram.tiff", sep = "."),
      width = 18500, height = 18500, units = "px", res = 800)
plot(dend2, type = "fan", tip.color = col3, cex = 9.0, label.offset = 0)
legend("bottomleft", title = "Cell Types", title.col = "black",
       legend = c(expression(bold("Beta (INS)")), expression(bold("Alpha (GCG)")),
       expression(bold("Delta (SST)")), expression(bold("Gamma (PPY)")),  expression(bold("Epsilon (GHR
       expression(bold("Stellate (COL1A1)")), expression(bold("Acinar (PRSS1)")),
       expression(bold("Ductal (KRT19)")), expression(bold("None"))), text.col = colorCodes1,
       cex = 1.5, xjust=0, yjust=0)
legend("bottomright", title = "Disease State", title.col = "black",
       legend = c(expression(bold("NonT2D")), expression(bold("T2D"))),
       text.col = colorCodes,
       cex = 1.5, xjust=0, yjust=0)
par(new = TRUE)
plot(dend3, type = "fan", tip.color = cols, cex = 9.0, label.offset = 30)
par(new = TRUE)
plot(dend4, type = "fan", tip.color = col4, cex = 9.0, label.offset = 60)
legend("topright", title = "Patient", title.col = "black",
       legend = c(expression(bold("Patient 1")), expression(bold("Patient 2")),
                  expression(bold("Patient 3")), expression(bold("Patient 4")),
                  expression(bold("Patient 5")), expression(bold("Patient 6")),
                  expression(bold("Patient 7")), expression(bold("Patient 8"))),
       text.col = colorpats, cex = 1.5, xjust = 0, yjust = 0)
dev.off()
```

Session Information

```r
suppressPackageStartupMessages(library(Biobase))
suppressPackageStartupMessages(library(edgeR))
```

```
## Warning: package 'limma' was built under R version 3.3.1
```

```r
suppressPackageStartupMessages(library(ape))
suppressPackageStartupMessages(library(gplots))
suppressPackageStartupMessages(library(dendextend))
suppressPackageStartupMessages(library(RColorBrewer))
library(edgeR)
library(Biobase)
library(gplots)
library(dendextend)
library(ape)
library(RColorBrewer)
sessionInfo()
```

```
## R version 3.3.0 (2016-05-03)
## Platform: x86_64-apple-darwin13.4.0 (64-bit)
## Running under: OS X 10.11.6 (El Capitan)
##
## locale:
## [1] en_US.UTF-8/en_US.UTF-8/en_US.UTF-8/C/en_US.UTF-8/en_US.UTF-8
##
## attached base packages:
## [1] parallel  stats     graphics  grDevices utils     datasets  methods
```

```
## [8] base
##
## other attached packages:
## [1] RColorBrewer_1.1-2  dendextend_1.3.0    gplots_3.0.1
## [4] ape_3.5             edgeR_3.14.0        limma_3.28.21
## [7] Biobase_2.32.0      BiocGenerics_0.18.0
##
## loaded via a namespace (and not attached):
##  [1] Rcpp_0.12.7        DEoptimR_1.0-6     formatR_1.4
##  [4] plyr_1.8.4         class_7.3-14       bitops_1.0-6
##  [7] tools_3.3.0        prabclus_2.2-6     digest_0.6.10
## [10] mclust_5.2         evaluate_0.10      tibble_1.2
## [13] nlme_3.1-128       gtable_0.2.0       lattice_0.20-34
## [16] yaml_2.1.13        mvtnorm_1.0-5      trimcluster_0.1-2
## [19] stringr_1.1.0      knitr_1.14         cluster_2.0.5
## [22] gtools_3.5.0       caTools_1.17.1     fpc_2.1-10
## [25] diptest_0.75-7     stats4_3.3.0       grid_3.3.0
## [28] nnet_7.3-12        robustbase_0.92-6  flexmix_2.3-13
## [31] rmarkdown_1.1      gdata_2.17.0       kernlab_0.9-25
## [34] ggplot2_2.1.0      magrittr_1.5       whisker_0.3-2
## [37] scales_0.4.0       htmltools_0.3.5    modeltools_0.2-21
## [40] MASS_7.3-45        assertthat_0.1     colorspace_1.2-7
## [43] KernSmooth_2.23-15 stringi_1.1.2      munsell_0.4.3
```
