# Supplementary material for: Single-cell transcriptomes identify human islet cell signatures and reveal cell-type–specific expression changes in type 2 diabetes
Source: Genome Res. 2017 Feb;27(2):208–22. doi: 10.1101/gr.212720.116 (PMC5287227; doi:10.1101/gr.212720.116)
Supplement: Supplemental Material [file supp_gr.212720.116_Supplemental_Methods_Source_Code.zip › Supplemental_Methods_Source_Code/Supplemental_Figure_Source_Code/Supplemental_Fig_S22_Source_Code.pdf]

Islet de-differentiation genes were not differentially expressed in single cell nor paired bulk intact islet transcriptomes

Introduction

The de-differentiation of islet cells has been proposed as a mechanism for type 2 diabetes progression. Several studies including Talchai et al. 2012 have defined genes (such as FOXO1, SOX9, NEUROG3, MYCL, POU5F1, NANOG) that are expressed by islets that have reverted to an endocrine progenitor-like state. After one-way ANOVA, we did not identify any of these islet de-differentiation genes to be significantly differential (p-value < 0.05) in any islet cell type or paired bulk intact islet.

Box plots showing the log2(CPM) expression of defined islet de-differentiation genes in single cell transcriptomes

```r
suppressPackageStartupMessages(library(Biobase))
suppressPackageStartupMessages(library(edgeR))
library(Biobase)
library(edgeR)
setwd("/Users/lawlon/Documents/Final_RNA_Seq_3/Data/")
# Load in the ND data
load("nonT2D.rdata")
ND.anns <- pData(cnts.eset)
ND.cnts <- exprs(cnts.eset)
p.anns <- as(featureData(cnts.eset), "data.frame")
# Calculate the cpm of the data
ND.cpms <- cpm(x = ND.cnts)
ND.data <- log2(ND.cpms+1)
# Load in T2D data
load("T2D.rdata")
T2D.anns <- pData(cnts.eset)
T2D.cnts <- exprs(cnts.eset)
# Calculate cpm
T2D.cpm <- cpm(x = T2D.cnts)
T2D.data <- log2(T2D.cpm+1)
# Load in de-diff genes
genes.sel <- c("FOXO1", "SOX9", "NEUROG3", "MYCL", "POU5F1", "NANOG")
# Cell groups
grp1 <- "INS"
grp2 <- "GCG"
grp3 <- "SST"
grp4 <- "PPY"
# Separate out the groups by disease state
ND.bet <- ND.anns[ND.anns$cell.type %in% c(grp1),]
ND.alp <- ND.anns[ND.anns$cell.type %in% c(grp2),]
ND.del <- ND.anns[ND.anns$cell.type %in% c(grp3),]
ND.gam <- ND.anns[ND.anns$cell.type %in% c(grp4),]
T2D.bet <- T2D.anns[T2D.anns$cell.type %in% c(grp1),]
```

```
T2D.alp <- T2D.anns[T2D.anns$cell.type %in% c(grp2),]
T2D.del <- T2D.anns[T2D.anns$cell.type %in% c(grp3),]
T2D.gam <- T2D.anns[T2D.anns$cell.type %in% c(grp4),]
# Expression data
ND.bet.exp <- ND.data[, rownames(ND.bet)]
ND.alp.exp <- ND.data[, rownames(ND.alp)]
ND.del.exp <- ND.data[, rownames(ND.del)]
ND.gam.exp <- ND.data[, rownames(ND.gam)]
T2D.bet.exp <- T2D.data[, rownames(T2D.bet)]
T2D.alp.exp <- T2D.data[, rownames(T2D.alp)]
T2D.del.exp <- T2D.data[, rownames(T2D.del)]
T2D.gam.exp <- T2D.data[, rownames(T2D.gam)]

# Boxplots of the ND data separated by gender and cell type
par(mfrow=c(2,3))
for (i in 1:length(genes.sel)) {
  idx <- which(p.anns$Associated.Gene.Name == genes.sel[i])
  if ( length(idx) > 0) {
    boxplot(ND.bet.exp[idx,], T2D.bet.exp[idx,], ND.alp.exp[idx,], T2D.alp.exp[idx,],
            ND.del.exp[idx,], T2D.del.exp[idx,], ND.gam.exp[idx,], T2D.gam.exp[idx,],
            names = c("ND", "T2D", "ND", "T2D",
                      "ND", "T2D", "ND", "T2D"),
            col = c("#e41a1c", "#e41a1c", "#377eb8", "#377eb8",
                    "#4daf4a", "#4daf4a", "#984ea3", "#984ea3"),
            main = genes.sel[i], ylab = "log2(CPM)", ylim = c(0,20), cex.axis = 1.5, cex.lab = 1.5, cex
    legend("topright", legend = c("Beta", "Alpha", "Delta", "Gamma"),
           text.col = c("#e41a1c", "#377eb8","#4daf4a", "#984ea3"), cex = 1.5)
  }
}
```

Box plots showing the log2(CPM) expression of defined islet de-differentiation genes in bulk intact transcriptomes

```
setwd("/Users/lawlon/Documents/Final_RNA_Seq/")
type <- "Intact"
load("islet_bulk_uniq_data.rdata")
p.anns <- as(featureData(bulk.cnts),"data.frame")
# Get count data
bulk.counts <- exprs(bulk.cnts)
# Get sample annotations
bulk.anns <- pData(bulk.cnts)
bulk.anns.sel <- bulk.anns[bulk.anns$Type == type,]
# Isolate ND samples
nonT2D.anns <- bulk.anns.sel[bulk.anns.sel$Phenotype == "ND",]
# Separate counts
ND.counts <- bulk.counts[, rownames(nonT2D.anns)]
# Calculate cpm
ND.cpm <- cpm(x = ND.counts)
ND.data <- log2(ND.cpm+1)
#Isolate T2D data
T2D.anns <- bulk.anns.sel[bulk.anns.sel$Phenotype == "T2D",]
# Separate counts
```

```r
T2D.counts <- bulk.counts[, rownames(T2D.anns)]
# Calculate cpm
T2D.cpm <- cpm(x = T2D.counts)
T2D.data <- log2(T2D.cpm+1)
# Load in de-diff genes
genes.sel <- c("FOXO1", "SOX9", "NEUROG3", "MYCL", "POU5F1", "NANOG")
# Boxplots of the ND data separated by gender and cell type
par(mfrow=c(2,3))
for (i in 1:length(genes.sel)) {
  idx <- which(p.anns$Associated.Gene.Name == genes.sel[i])
  if ( length(idx) > 0) {
    boxplot(ND.data[idx,], T2D.data[idx,],
            names = c("ND Bulk", "T2D Bulk"),
            col = c("#bda2e5","#10d2f0"),
            main = genes.sel[i], ylab = "log2(CPM)", ylim = c(0,20), cex.axis = 2, cex.lab = 1.5, cex.ma
  }
}
```

Session Information

```r
suppressPackageStartupMessages(library(Biobase))
suppressPackageStartupMessages(library(edgeR))
```

```
## Warning: package 'limma' was built under R version 3.3.1
```

```r
library(Biobase)
library(edgeR)
sessionInfo()
```

```
## R version 3.3.0 (2016-05-03)
## Platform: x86_64-apple-darwin13.4.0 (64-bit)
## Running under: OS X 10.11.6 (El Capitan)
##
## locale:
## [1] en_US.UTF-8/en_US.UTF-8/en_US.UTF-8/C/en_US.UTF-8/en_US.UTF-8
##
## attached base packages:
## [1] parallel  stats     graphics  grDevices utils     datasets  methods
## [8] base
##
## other attached packages:
## [1] edgeR_3.14.0        limma_3.28.21       Biobase_2.32.0
## [4] BiocGenerics_0.18.0
##
## loaded via a namespace (and not attached):
##  [1] Rcpp_0.12.7     digest_0.6.10   assertthat_0.1  formatR_1.4
##  [5] magrittr_1.5    evaluate_0.10   stringi_1.1.2   rmarkdown_1.1
##  [9] tools_3.3.0     stringr_1.1.0   yaml_2.1.13     htmltools_0.3.5
## [13] knitr_1.14      tibble_1.2
```
