# Supplementary material for: Single-cell transcriptomes identify human islet cell signatures and reveal cell-type–specific expression changes in type 2 diabetes
Source: Genome Res. 2017 Feb;27(2):208–22. doi: 10.1101/gr.212720.116 (PMC5287227; doi:10.1101/gr.212720.116)
Supplement: Supplemental Material [file supp_gr.212720.116_Supplemental_Methods_Source_Code.zip › Supplemental_Methods_Source_Code/Supplemental_Figure_Source_Code/Supplemental_Fig_S23_Source_Code.pdf]

Figure5-exocrine

June 21, 2016

In [2]:
```
%matplotlib inline
import pandas as pd
import numpy as np
import matplotlib
import matplotlib.pyplot as plt
from matplotlib.backends.backend_pdf import PdfPages
import scipy.stats as stats
import scipy.cluster.hierarchy as sch
from operator import *
pd.core.config.option_context('mode.use_inf_as_null',True)
import seaborn as sns
rcdefsns = plt.rcParams.copy()
import brewer2mpl
import os
import sys
import bokeh
from bokeh.plotting import ColumnDataSource, figure, show, gridplot,output_file,hplot,vplot
from bokeh.models import HoverTool
bokeh.io.output_notebook()

matplotlib.rcParams['figure.figsize'] = (5.0, 5.0)
matplotlib.rcParams['axes.linewidth'] = 3
matplotlib.rcParams['axes.edgecolor'] = 'k'
matplotlib.rcParams['axes.spines.top']='False'
matplotlib.rcParams['axes.spines.right']='False'
matplotlib.rcParams['axes.spines.right']='False'

TOOLS="pan,wheel_zoom,box_zoom,reset,hover,lasso_select,save"
```

In [3]:
```
bulk_dge=pd.read_csv('T2D.vs.NonT2D.Bulk.Intact.csv',index_col=0)
sc_acinar=pd.read_csv(
    'T2D_vs_NonT2D_Differential_Gene_Lists/EdgeR.Robust.T2D.vs.NonT2D.Gender.Covariate.Acinar.c
    index_col=0)
sc_ductal=pd.read_csv(
    'T2D_vs_NonT2D_Differential_Gene_Lists/EdgeR.Robust.T2D.vs.NonT2D.Gender.Covariate.Ductal.c
    index_col=0)
sc_stellate=pd.read_csv(
    'T2D_vs_NonT2D_Differential_Gene_Lists/EdgeR.Robust.T2D.vs.NonT2D.Gender.Covariate.Stellate
    index_col=0)
```

In [4]:
```
matplotlib.rcParams['figure.figsize'] = (25.0, 5.0)
fig,ax=plt.subplots(1,4,sharey=True)
ax[0].axvline(0,color='k',linestyle='--')
ax[0].axhline(0,color='k',linestyle='--')
```

```python
ax[0].grid('off')
ax[0].set_axis_bgcolor('white')

merged=bulk_dge.join(sc_acinar,rsuffix='SC',how='outer')
merged['color']=merged['FDRSC'].apply(lambda x: '#000000' if x < 0.05 else '#bdbdbd')
merged.loc[merged[merged['Associated.Gene.Name']=='INS'].index,'color']='blue'
ax[0].axvline(0,color='k',linestyle='--')
hold=merged[merged['color']=='#bdbdbd']
ax[0].scatter(hold['logFCSC'],hold['logFC'],c='#bdbdbd',s=20,lw=0.,label='FDR > 0.05 SC')
hold=merged[merged['color']!='#bdbdbd']
ax[0].scatter(hold['logFCSC'],hold['logFC'],c='#000000',s=40,lw=0., label='FDR < 0.05 SC')
ax[0].tick_params(axis='both', which='major', labelsize=16)
ax[0].set_ylabel('log(FC) - Bulk T2D vs ND', fontsize=16)
ax[0].set_xlabel(r'log(FC) - Single Cell Acinar T2D vs ND',fontsize=16)
ax[0].set_ylim(-8,8)
ax[0].set_xlim(-10,10)


ax[1].axvline(0,color='k',linestyle='--')
ax[1].axhline(0,color='k',linestyle='--')
ax[1].grid('off')
ax[1].set_axis_bgcolor('white')
merged=bulk_dge.join(sc_ductal,rsuffix='SC',how='outer')
merged['color']=merged['FDRSC'].apply(lambda x: '#000000' if x < 0.05 else '#bdbdbd')
hold=merged[merged['color']=='#bdbdbd']
ax[1].scatter(hold['logFCSC'],hold['logFC'],c='#bdbdbd',s=20,lw=0.,label='FDR > 0.05 SC')
hold=merged[merged['color']!='#bdbdbd']
ax[1].scatter(hold['logFCSC'],hold['logFC'],c=hold['color'],s=40,lw=0., label='FDR < 0.05 SC')
ax[1].tick_params(axis='both', which='major', labelsize=16)
ax[1].set_xlabel(r'log(FC) - Single Cell Ductal T2D vs ND',fontsize=16)
ax[1].set_ylim(-8,8)
ax[1].set_xlim(-10,10)

ax[2].axvline(0,color='k',linestyle='--')
ax[2].axhline(0,color='k',linestyle='--')
ax[2].grid('off')
ax[2].set_axis_bgcolor('white')
merged=bulk_dge.join(sc_stellate,rsuffix='SC',how='outer')
merged['color']=merged['FDRSC'].apply(lambda x: '#000000' if x < 0.05 else '#bdbdbd')
hold=merged[merged['color']=='#bdbdbd']
ax[2].scatter(hold['logFCSC'],hold['logFC'],c='#bdbdbd',s=20,lw=0.,label='FDR > 0.05 SC')
hold=merged[merged['color']=='#000000']
ax[2].scatter(hold['logFCSC'],hold['logFC'],c='#000000',s=40,lw=0., label='FDR < 0.05 SC')
ax[2].tick_params(axis='both', which='major', labelsize=16)
ax[2].set_xlabel(r'log(FC) - Single Cell Stellate T2D vs ND',fontsize=16)
ax[2].set_ylim(-8,8)
ax[2].set_xlim(-15,15)

# ax[3].axvline(0,color='k',linestyle='--')
# ax[3].axhline(0,color='k',linestyle='--')
# ax[3].grid('off')
# ax[3].set_axis_bgcolor('white')
# merged=bulk_dge.join(sc_delta,rsuffix='SC',how='outer')
# merged['color']=merged['FDRSC'].apply(lambda x: '#4DAF4A' if x < 0.05 else '#bdbdbd')
```

```
# hold=merged[merged['color']=='#bdbdbd']
# ax[3].scatter(hold['logFCSC'],hold['logFC'],c='#bdbdbd',s=20,lw=0.,label='FDR > 0.05 SC')
# hold=merged[merged['color']!='#bdbdbd']
# ax[3].scatter(hold['logFCSC'],hold['logFC'],c='#4DAF4A',s=40,lw=0., label='FDR < 0.05 SC')
# ax[3].tick_params(axis='both', which='major', labelsize=16)
# ax[3].set_xlabel(r'log(FC) - Single Cell £\delta£ T2D vs ND',fontsize=16)
# ax[3].set_ylim(-3,3)
# ax[3].set_xlim(-15,15)

ax[3].axis('off')

fig.tight_layout()
fig.savefig('Figures_06142016/Figure5_Exocrine.pdf',dpi=300,format='pdf')
```

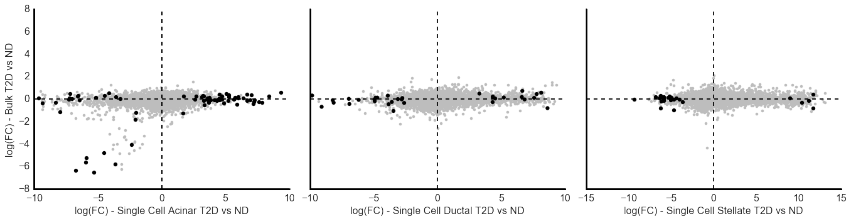

In [5]:
```
output_file("Figures_06142016/Figure5_DGE-T2DvND_Bulk-SC-DuctalvsDuctal.html")
p = figure(title="DGE: T2D vs ND - Bulk and Single Cell Ductal", tools=TOOLS, width=650, plot_h

merged=bulk_dge.join(sc_ductal,rsuffix='SC',how='outer')
merged['color']=merged['FDRSC'].apply(lambda x: '#000000' if x < 0.05 else '#bdbdbd')
hold=merged[merged['color']=='#bdbdbd'].replace('nan',np.nan).dropna()
color='#bdbdbd'
genename=hold['Associated.Gene.Name'].values
fdr_bulk=hold['FDR'].values
fdr_sc=hold['FDRSC'].values
fc_bulk=hold['logFC'].values
fc_sc=hold['logFCSC'].values

source = ColumnDataSource(
data=dict(
    x=fc_bulk,
    y=fc_sc,
    a=genename,
    b=fdr_bulk,
    c=fdr_sc,
    d=fc_bulk,
    e=fc_sc
    ))

p.circle(fc_sc,fc_bulk,radius=.05,fill_color=color,line_color=color,source=source,legend='FDR >

hold=merged[merged['color']=='#000000'].replace('nan',np.nan).dropna()
color='#000000'
genename=hold['Associated.Gene.Name'].values
fdr_bulk=hold['FDR'].values
```

```
fdr_sc=hold['FDRSC'].values
fc_bulk=hold['logFC'].values
fc_sc=hold['logFCSC'].values

source = ColumnDataSource(
data=dict(
    x=fc_bulk,
    y=fc_sc,
    a=genename,
    b=fdr_bulk,
    c=fdr_sc,
    d=fc_bulk,
    e=fc_sc
    ))

p.circle(fc_sc,fc_bulk,radius=.05,fill_color=color,line_color=color,source=source,legend='FDR <

hover = p.select(dict(type=HoverTool))
hover.tooltips = [
("index", "$index"),
("log(FC)-Bulk,log(FC)-SC", "($x, $y)"),
("Gene", "@a"),
("FDR-Bulk", "@b"),
("FDR-SC", "@c"),
]
p.yaxis.axis_label='log(FC) - Bulk T2D vs ND'
p.xaxis.axis_label='log(FC) - Single Cell beta T2D vs ND'
#p.xaxis.bounds=(-6,6)
show(p)
```

Out[5]: <bokeh.io._CommsHandle at 0x11bc8d510>

In [6]:
```
output_file("Figures_06142016/Figure5_DGE-T2DvND_Bulk-SC-Acinar.html")
p = figure(title="DGE: T2D vs ND - Bulk and Single Cell - Acinar", tools=TOOLS, width=650, plot_

merged=bulk_dge.join(sc_acinar,rsuffix='SC',how='outer')
merged['color']=merged['FDRSC'].apply(lambda x: '#000000' if x < 0.05 else '#bdbdbd')
hold=merged[merged['color']=='#bdbdbd'].replace('nan',np.nan).dropna()
color='#bdbdbd'
genename=hold['Associated.Gene.Name'].values
fdr_bulk=hold['FDR'].values
fdr_sc=hold['FDRSC'].values
fc_bulk=hold['logFC'].values
fc_sc=hold['logFCSC'].values

source = ColumnDataSource(
data=dict(
    x=fc_bulk,
    y=fc_sc,
    a=genename,
    b=fdr_bulk,
    c=fdr_sc,
    d=fc_bulk,
    e=fc_sc
    ))
```

```
p.circle(fc_sc,fc_bulk,radius=.05,fill_color=color,line_color=color,source=source,legend='FDR >

hold=merged[merged['color']=='#000000'].replace('nan',np.nan).dropna()
color='#000000'
genename=hold['Associated.Gene.Name'].values
fdr_bulk=hold['FDR'].values
fdr_sc=hold['FDRSC'].values
fc_bulk=hold['logFC'].values
fc_sc=hold['logFCSC'].values

source = ColumnDataSource(
data=dict(
    x=fc_bulk,
    y=fc_sc,
    a=genename,
    b=fdr_bulk,
    c=fdr_sc,
    d=fc_bulk,
    e=fc_sc
    ))

p.circle(fc_sc,fc_bulk,radius=.05,fill_color=color,line_color=color,source=source,legend='FDR <

hover = p.select(dict(type=HoverTool))
hover.tooltips = [
("index", "$index"),
("log(FC)-Bulk,log(FC)-SC", "($x, $y)"),
("Gene", "@a"),
("FDR-Bulk", "@b"),
("FDR-SC", "@c"),
]
p.yaxis.axis_label='log(FC) - Bulk T2D vs ND'
p.xaxis.axis_label='log(FC) - Single Cell alpha T2D vs ND'
#p.xaxis.bounds=(-6,6)
show(p)
```

Out[6]: <bokeh.io._CommsHandle at 0x11bd384d0>

In [7]:
```
output_file("Figures_06142016/Figure5_DGE-T2DvND_Bulk-SC-Stellate.html")
p = figure(title="DGE: T2D vs ND - Bulk and Single Cell - Stellate", tools=TOOLS, width=650, pl

merged=bulk_dge.join(sc_stellate,rsuffix='SC',how='outer')
merged['color']=merged['FDRSC'].apply(lambda x: '#000000' if x < 0.05 else '#bdbdbd')
hold=merged[merged['color']=='#bdbdbd'].replace('nan',np.nan).dropna()
color='#bdbdbd'
genename=hold['Associated.Gene.Name'].values
fdr_bulk=hold['FDR'].values
fdr_sc=hold['FDRSC'].values
fc_bulk=hold['logFC'].values
fc_sc=hold['logFCSC'].values

source = ColumnDataSource(
data=dict(
    x=fc_bulk,
```

```
        y=fc_sc,
        a=genename,
        b=fdr_bulk,
        c=fdr_sc,
        d=fc_bulk,
        e=fc_sc
        ))

    p.circle(fc_sc,fc_bulk,radius=.05,fill_color=color,line_color=color,source=source,legend='FDR >


    hold=merged[merged['color']=='#000000'].replace('nan',np.nan).dropna()
    color='#000000'
    genename=hold['Associated.Gene.Name'].values
    fdr_bulk=hold['FDR'].values
    fdr_sc=hold['FDRSC'].values
    fc_bulk=hold['logFC'].values
    fc_sc=hold['logFCSC'].values

    source = ColumnDataSource(
    data=dict(
        x=fc_bulk,
        y=fc_sc,
        a=genename,
        b=fdr_bulk,
        c=fdr_sc,
        d=fc_bulk,
        e=fc_sc
        ))

    p.circle(fc_sc,fc_bulk,radius=.05,fill_color=color,line_color=color,source=source,legend='FDR <

    hover = p.select(dict(type=HoverTool))
    hover.tooltips = [
    ("index", "$index"),
    ("log(FC)-Bulk,log(FC)-SC", "($x, $y)"),
    ("Gene", "@a"),
    ("FDR-Bulk", "@b"),
    ("FDR-SC", "@c"),
    ]
    p.yaxis.axis_label='log(FC) - Bulk T2D vs ND'
    p.xaxis.axis_label='log(FC) - Single Cell delta T2D vs ND'
    #p.xaxis.bounds=(-6,6)
    show(p)
```

Out[7]: <bokeh.io._CommsHandle at 0x11bd387d0>

In [ ]:
