# Supplementary material for: Single-cell transcriptomes identify human islet cell signatures and reveal cell-type–specific expression changes in type 2 diabetes
Source: Genome Res. 2017 Feb;27(2):208–22. doi: 10.1101/gr.212720.116 (PMC5287227; doi:10.1101/gr.212720.116)
Supplement: Supplemental Material [file supp_gr.212720.116_Supplemental_Methods_Source_Code.zip › Supplemental_Methods_Source_Code/Supplemental_Figure_Source_Code/Supplemental_Fig_S24_Source_Code.pdf]

Comparison of T2D and Non-diabetic Cell Type Specific Differentially Expressed Genes between Lawlor et al. 2016, Segerstolpe et al. 2016, and Wang et al. 2016

Introduction

Using a two-sided Wilcoxon rank sum test, we sought to validate the observed cell type-specific differences in T2D islets from recent, independent islet single cell RNA-seq studies (Wang et al. 2016; Segerstolpe et al., 2016). Resuls are summarized in venn diagram format.

Venn Diagram showing overlap of differentially expressed genes across studies

```
suppressPackageStartupMessages(library(venneuler))
suppressPackageStartupMessages(library(gridExtra))
suppressPackageStartupMessages(library(readxl))
library(venneuler)
library(gridExtra)
library(readxl)
# make venn diagram of overlapping genes
# separate diagrams by direction of fold change in expression
beta.pos <- read.csv("Sandberg.T2D.vs.NonT2D.beta cell.53.pos.FC.intersected.genes.csv",
                     header = T, check.names = F, row.names = 1, stringsAsFactors = F)
beta.neg <- read.csv("Sandberg.T2D.vs.NonT2D.beta cell.21.neg.FC.intersected.genes.csv",
                     header = T, check.names = F, row.names = 1, stringsAsFactors = F)
alpha.pos <- read.csv("Sandberg.T2D.vs.NonT2D.alpha cell.32.pos.FC.intersected.genes.csv",
                      header = T, check.names = F, row.names = 1, stringsAsFactors = F)
alpha.neg <- read.csv("Sandberg.T2D.vs.NonT2D.alpha cell.9.neg.FC.intersected.genes.csv",
                      header = T, check.names = F, row.names = 1, stringsAsFactors = F)
delta.pos <- read.csv("Sandberg.T2D.vs.NonT2D.delta cell.1.pos.FC.intersected.genes.csv",
                      header = T, check.names = F, row.names = 1, stringsAsFactors = F)
delta.neg <- read.csv("Sandberg.T2D.vs.NonT2D.delta cell.1.neg.FC.intersected.genes.csv",
                      header = T, check.names = F, row.names = 1, stringsAsFactors = F)
sand.beta <- read.csv("Sandberg.T2D.vs.NonT2D.beta cell.233.out.of.248.genes.used.csv",
                      header = T, check.names = F, row.names = 1, stringsAsFactors = F)
sand.alpha <- read.csv("Sandberg.T2D.vs.NonT2D.alpha cell.134.out.of.138.genes.used.csv",
                       header = T, check.names = F, row.names = 1, stringsAsFactors = F)
sand.delta <- read.csv("Sandberg.T2D.vs.NonT2D.delta cell.23.out.of.24.genes.used.csv",
                       header = T, check.names = F, row.names = 1, stringsAsFactors = F)
# Load in kaestner results
b.pos <- read.csv("Kaestner.T2D.vs.NonT2D.beta.11.pos.FC.intersected.genes.csv",
                  header = T, check.names = F, row.names = 1, stringsAsFactors = F)
b.neg <- read.csv("Kaestner.T2D.vs.NonT2D.beta.12.neg.FC.intersected.genes.csv",
                  header = T, check.names = F, row.names = 1, stringsAsFactors = F)
a.pos <- read.csv("Kaestner.T2D.vs.NonT2D.alpha.14.pos.FC.intersected.genes.csv",
                  header = T, check.names = F, row.names = 1, stringsAsFactors = F)
a.neg <- read.csv("Kaestner.T2D.vs.NonT2D.alpha.5.neg.FC.intersected.genes.csv",
                  header = T, check.names = F, row.names = 1, stringsAsFactors = F)
d.pos <- read.csv("Kaestner.T2D.vs.NonT2D.delta.0.pos.FC.intersected.genes.csv",
```

```r
                  header = T, check.names = F, row.names = 1, stringsAsFactors = F)
d.neg <- read.csv("Kaestner.T2D.vs.NonT2D.delta.0.neg.FC.intersected.genes.csv",
                  header = T, check.names = F, row.names = 1, stringsAsFactors = F)
k.beta <- read.csv("Kaestner.T2D.vs.NonT2D.beta.228.out.of.248.genes.used.csv",
                   header = T, check.names = F, row.names = 1, stringsAsFactors = F)
k.alpha <- read.csv("Kaestner.T2D.vs.NonT2D.alpha.126.out.of.138.genes.used.csv",
                    header = T, check.names = F, row.names = 1, stringsAsFactors = F)
k.delta <- read.csv("Kaestner.T2D.vs.NonT2D.delta.19.out.of.24.genes.used.csv",
                    header = T, check.names = F, row.names = 1, stringsAsFactors = F)

# Only use genes in common between lists (ones that had some sort of FC in the ANOVA)
diff.b <- setdiff(sand.beta[,1], k.beta[,1])
diff.a <- setdiff(sand.alpha[,1], k.alpha[,1])
diff.d <- setdiff(sand.delta[,1], k.delta[,1])

# find intersection between pos and neg fc results
int.b.pos <- Reduce(intersect, list(beta.pos[,1], sand.beta[,1], b.pos[,1], k.beta[,1]))
int.b.neg <- Reduce(intersect, list(beta.neg[,1], sand.beta[,1], b.neg[,1], k.beta[,1]))
int.a.pos <- Reduce(intersect, list(alpha.pos[,1], sand.alpha[,1], a.pos[,1], k.alpha[,1]))
int.a.neg <- Reduce(intersect, list(alpha.neg[,1], sand.alpha[,1], a.neg[,1], k.alpha[,1]))
int.d.pos <- Reduce(intersect, list(delta.pos[,1], sand.delta[,1], d.pos[,1], k.delta[,1]))
int.d.neg <- Reduce(intersect, list(delta.neg[,1], sand.delta[,1], d.neg[,1], k.delta[,1]))

# Make venn diagrams of the results (split by Positive FC and negative FC)
# color panel
col = c("#e41a1c", "#377eb8", "#4daf4a", "#654321")

# beta up genes
ve1 <- venneuler(c(A = length(beta.pos[,1]), B = length(b.pos[,1]), "A&B" = length(int.b.pos)))
ve1$labels <- c(
  paste("Segerstolpe et al. 2016\n", length(beta.pos[,1])),
  paste("Wang et al. 2016\n", length(b.pos[,1])),
  paste("\n\n\n\n\n\nInt\n", length(int.b.pos))
)
plot(ve1, col = col[1], main = "T2D Genes up in Beta")

# beta down genes
ve2 <- venneuler(c(A = length(beta.neg[,1]), B = length(b.neg[,1]), "A&B" = length(int.b.neg)))
ve2$labels <- c(
  paste("Segerstolpe et al. 2016\n", length(beta.neg[,1])),
  paste("Wang et al. 2016\n", length(b.neg[,1])),
  paste("\n\n\n\n\n\nInt\n", length(int.b.neg))
)
plot(ve2, col = col[1], main = "T2D Genes down in Beta")

# alpha up genes
ve3 <- venneuler(c(A = length(alpha.pos[,1]), B = length(a.pos[,1]), "A&B" = length(int.a.pos)))
ve3$labels <- c(
  paste("Segerstolpe et al. 2016\n", length(alpha.pos[,1])),
  paste("Wang et al. 2016\n", length(a.pos[,1])),
  paste("\n\n\n\n\n\nInt\n", length(int.a.pos))
)
plot(ve3, col = col[2], main = "T2D Genes up in Alpha")
```

```
ve4 <- venneuler(c(A = length(alpha.neg[,1]), B = length(a.neg[,1]), "A&B" = length(int.a.neg)))
ve4$labels <- c(
  paste("Segerstolpe et al. 2016\n", length(alpha.neg[,1])),
  paste("Wang et al. 2016\n", length(a.neg[,1])),
  paste("\n\n\n\n\n\nInt\n", length(int.a.neg))
)
plot(ve4, col = col[2], main = "T2D Genes down in Alpha")
```

Violin Plots depicting expression of DLK1 and CD36 in other datasets

```
suppressPackageStartupMessages(library(Biobase))
suppressPackageStartupMessages(library(vioplot))
suppressPackageStartupMessages(library(sm))
library(Biobase)
library(vioplot)
library(sm)
# load in Sandberg data
setwd("/Users/lawlon/Documents/Sandberg_Islet/")
load("Sandberg.processed.FPKM.rdata")
# convert to numeric matrix
sand.dat <- exprs(eset)
sand.num <- apply(sand.dat,1,as.numeric)
sand.exp <- t(sand.num)
colnames(sand.exp) <- colnames(sand.dat)
sand.anns <- read.csv("/Users/lawlon/Documents/Sandberg_Islet/Sandberg.islet.sample.annotations.ordered
                      check.names = F, row.names = NULL, stringsAsFactors = F)
# set column names
col.names <- as.character(sand.anns[1,])
colnames(sand.anns) <- col.names
sand.anns <- sand.anns[-1,]
# set rownames as sample names
rownames(sand.anns) <- sand.anns[,1]
sand.anns[,1] <- NULL
# specify cell type
cell_type = "beta cell"
name1 <- "Beta"

# Use samples with phenotype info
nont2d.ids <- which(sand.anns$`Characteristics[cell type]` == cell_type & sand.anns$`Factor Value[diseas
sand.nont2d <- sand.exp[, nont2d.ids]
t2d.ids <- which(sand.anns$`Characteristics[cell type]` == cell_type & sand.anns$`Factor Value[disease]
sand.t2d <- sand.exp[, t2d.ids]

# Load genes to compare
genes <- "DLK1"
idx <- which(rownames(sand.exp) == genes)
# log transform data
nd.log <- log2(sand.nont2d+1)
t2d.log <- log2(sand.t2d+1)

# vioplot function
```

```
vioplot <- function(x,...,range=1.5,h=NULL,ylim=NULL,names=NULL, horizontal=FALSE,
                    col="magenta", border="black", lty=1, lwd=1, rectCol="black", colMed="white", pchMe[illegible]
                    drawRect=TRUE)
{
  # process multiple datas
  datas <- list(x,...)
  n <- length(datas)

  if(missing(at)) at <- 1:n

  # pass 1
  #
  # - calculate base range
  # - estimate density
  #

  # setup parameters for density estimation
  upper  <- vector(mode="numeric",length=n)
  lower  <- vector(mode="numeric",length=n)
  q1     <- vector(mode="numeric",length=n)
  q3     <- vector(mode="numeric",length=n)
  med    <- vector(mode="numeric",length=n)
  base   <- vector(mode="list",length=n)
  height <- vector(mode="list",length=n)
  baserange <- c(Inf,-Inf)

  # global args for sm.density function-call
  args <- list(display="none")

  if (!(is.null(h)))
    args <- c(args, h=h)

  for(i in 1:n) {
    data<-datas[[i]]

    # calculate plot parameters
    #   1- and 3-quantile, median, IQR, upper- and lower-adjacent
    data.min <- min(data)
    data.max <- max(data)
    q1[i]<-quantile(data,0.25)
    q3[i]<-quantile(data,0.75)
    med[i]<-median(data)
    iqd <- q3[i]-q1[i]
    upper[i] <- min( q3[i] + range*iqd, data.max )
    lower[i] <- max( q1[i] - range*iqd, data.min )

    #   strategy:
    #       xmin = min(lower, data.min))
    #       ymax = max(upper, data.max))
    #

    est.xlim <- c( min(lower[i], data.min), max(upper[i], data.max) )
```

```
    # estimate density curve
    smout <- do.call("sm.density", c( list(data, xlim=est.xlim), args ) )

    # calculate stretch factor
    #
    #  the plots density heights is defined in range 0.0 ... 0.5
    #  we scale maximum estimated point to 0.4 per data
    #
    hscale <- 0.4/max(smout$estimate) * wex

    # add density curve x,y pair to lists
    base[[i]]   <- smout$eval.points
    height[[i]] <- smout$estimate * hscale

    # calculate min,max base ranges
    t <- range(base[[i]])
    baserange[1] <- min(baserange[1],t[1])
    baserange[2] <- max(baserange[2],t[2])

  }

  # pass 2
  #
  # - plot graphics

  # setup parameters for plot
  if(!add){
    xlim <- if(n==1)
      at + c(-.5, .5)
    else
      range(at) + min(diff(at))/2 * c(-1,1)

    if (is.null(ylim)) {
      ylim <- baserange
    }
  }
  if (is.null(names)) {
    label <- 1:n
  } else {
    label <- names
  }

  boxwidth <- 0.05 * wex

  # setup plot
  if(!add)
    plot.new()
  if(!horizontal) {
    if(!add){
      plot.window(xlim = xlim, ylim = ylim)
      axis(2)
      axis(1,at = at, label=label )
    }
```

```r
    box()
    for(i in 1:n) {
      # plot left/right density curve
      polygon( c(at[i]-height[[i]], rev(at[i]+height[[i]])),
               c(base[[i]], rev(base[[i]])),
               col = col[i %% length(col) + 1], border=border, lty=lty, lwd=lwd)

      if(drawRect){
        # plot IQR
        lines( at[c( i, i)], c(lower[i], upper[i]) ,lwd=lwd, lty=lty)

        # plot 50% KI box
        rect( at[i]-boxwidth/2, q1[i], at[i]+boxwidth/2, q3[i], col=rectCol)

        # plot median point
        points( at[i], med[i], pch=pchMed, col=colMed )
      }
    }

  }
  else {
    if(!add){
      plot.window(xlim = ylim, ylim = xlim)
      axis(1)
      axis(2,at = at, label=label )
    }

    box()
    for(i in 1:n) {
      # plot left/right density curve
      polygon( c(base[[i]], rev(base[[i]])),
               c(at[i]-height[[i]], rev(at[i]+height[[i]])),
               col = col[i %% length(col) + 1], border=border, lty=lty, lwd=lwd)

      if(drawRect){
        # plot IQR
        lines( c(lower[i], upper[i]), at[c(i,i)] ,lwd=lwd, lty=lty)

        # plot 50% KI box
        rect( q1[i], at[i]-boxwidth/2, q3[i], at[i]+boxwidth/2,  col=rectCol)

        # plot median point
        points( med[i], at[i], pch=pchMed, col=colMed )
      }
    }
  }
  invisible (list( upper=upper, lower=lower, median=med, q1=q1, q3=q3))
}
## END VIOPLOT SOURCE CODE

# Plot expression of DLK1 in beta cells
vioplot(t(nd.log)[,idx], t(t2d.log)[,idx],
        names = c(paste("NonT2D", name1, sep = " "), paste("T2D", name1, sep = " ")),
```

```
        col = c("#10d2f0", "#bda2e5"),
        ylim = c(0,20))
title(main = genes, ylab = "log2(RPKM)", xlab = "", cex.main = 2,
      cex.axis = 1.5, cex.lab = 1.5)

# Plot expression of CD36 in alpha cells
cell_type = "alpha cell"
name1 <- "Alpha"
# Use samples with phenotype info
nont2d.ids <- which(sand.anns$`Characteristics[cell type]` == cell_type & sand.anns$`Factor Value[diseas
sand.nont2d <- sand.exp[, nont2d.ids]
t2d.ids <- which(sand.anns$`Characteristics[cell type]` == cell_type & sand.anns$`Factor Value[disease]
sand.t2d <- sand.exp[, t2d.ids]
# Load genes to compare
genes <- "CD36"
idx <- which(rownames(sand.exp) == genes)
# log transform data
nd.log <- log2(sand.nont2d+1)
t2d.log <- log2(sand.t2d+1)

# plot expression
vioplot(t(nd.log)[,idx], t(t2d.log)[,idx],
        names = c(paste("NonT2D", name1, sep = " "), paste("T2D", name1, sep = " ")),
        col = c("#10d2f0", "#bda2e5"),
        ylim = c(0,20))
title(main = genes, ylab = "log2(RPKM)", xlab = "", cex.main = 2,
      cex.axis = 1.5, cex.lab = 1.5)


# load in Kaestner dataset
wang.exp <- read.csv("/Users/lawlon/Documents/Kaestner_GSE83139/GSE83139.ordered.expression.matrix.cpm.
                   check.names = F, row.names = 1, stringsAsFactors = F)
wang.exp <- as.matrix(wang.exp)

wang.anns <- read.csv("/Users/lawlon/Documents/Kaestner_GSE83139/GSE83139.ordered.sample.annotations.cs
                   check.names = F, row.names = 3)
# specify cell type
cell_type = "beta"
name1 <- "Beta"

# Use samples with phenotype info
w.cont <- wang.anns[wang.anns$Health == "control" & wang.anns$Cell_Type %in% cell_type & wang.anns$Age =
w.t2d <- wang.anns[wang.anns$Health == "T2D" & wang.anns$Cell_Type %in% cell_type & wang.anns$Age == "a
w.cont <- as.matrix(w.cont)
w.cont <- as.data.frame(w.cont)
w.t2d <- as.matrix(w.t2d)
w.t2d <- as.data.frame(w.t2d)

# combine anns
w.sel <- rbind(w.cont, w.t2d)
# selected counts
wang.exp.ND <- wang.exp[, rownames(w.cont)]
wang.exp.T2D <- wang.exp[, rownames(w.t2d)]
```

```r
# Load genes to compare
genes <- "DLK1"
idx <- which(rownames(wang.exp) == genes)

# log transform data
nd.log <- log2(wang.exp.ND+1)
t2d.log <- log2(wang.exp.T2D+1)

# plot expression of DLK1 in wang dataset beta cells
vioplot(t(nd.log)[,idx], t(t2d.log)[,idx],
        names = c(paste("NonT2D", name1, sep = " "), paste("T2D", name1, sep = " ")),
        col = c("#10d2f0", "#bda2e5"),
        ylim = c(0,20))
title(main = genes, ylab = "log2(RPKM)", xlab = "", cex.main = 2,
      cex.axis = 1.5, cex.lab = 1.5)

# plot CD36 expression in wang dataset alpha cells
# specify cell type
cell_type = "alpha"
name1 <- "Alpha"

# Use samples with phenotype info
w.cont <- wang.anns[wang.anns$Health == "control" & wang.anns$Cell_Type %in% cell_type & wang.anns$Age =
w.t2d <- wang.anns[wang.anns$Health == "T2D" & wang.anns$Cell_Type %in% cell_type & wang.anns$Age == "a
w.cont <- as.matrix(w.cont)
w.cont <- as.data.frame(w.cont)
w.t2d <- as.matrix(w.t2d)
w.t2d <- as.data.frame(w.t2d)

# combine anns
w.sel <- rbind(w.cont, w.t2d)
# selected counts
wang.exp.ND <- wang.exp[, rownames(w.cont)]
wang.exp.T2D <- wang.exp[, rownames(w.t2d)]
# Load genes to compare
genes <- "CD36"
idx <- which(rownames(wang.exp) == genes)

# log transform data
nd.log <- log2(wang.exp.ND+1)
t2d.log <- log2(wang.exp.T2D+1)

# plot expression of DLK1 in wang dataset beta cells
vioplot(t(nd.log)[,idx], t(t2d.log)[,idx],
        names = c(paste("NonT2D", name1, sep = " "), paste("T2D", name1, sep = " ")),
        col = c("#10d2f0", "#bda2e5"),
        ylim = c(0,20))
title(main = genes, ylab = "log2(RPKM)", xlab = "", cex.main = 2,
      cex.axis = 1.5, cex.lab = 1.5)
```

Session Information

```r
suppressPackageStartupMessages(library(venneuler))
suppressPackageStartupMessages(library(gridExtra))
suppressPackageStartupMessages(library(readxl))
suppressPackageStartupMessages(library(Biobase))
suppressPackageStartupMessages(library(vioplot))
suppressPackageStartupMessages(library(sm))
library(Biobase)
library(vioplot)
library(sm)
library(venneuler)
library(gridExtra)
library(readxl)


sessionInfo()
```

```
## R version 3.3.0 (2016-05-03)
## Platform: x86_64-apple-darwin13.4.0 (64-bit)
## Running under: OS X 10.11.6 (El Capitan)
##
## locale:
## [1] en_US.UTF-8/en_US.UTF-8/en_US.UTF-8/C/en_US.UTF-8/en_US.UTF-8
##
## attached base packages:
## [1] parallel  stats     graphics  grDevices utils     datasets  methods
## [8] base
##
## other attached packages:
## [1] vioplot_0.2         sm_2.2-5.4          Biobase_2.32.0
## [4] BiocGenerics_0.18.0 readxl_0.1.1.9000   gridExtra_2.2.1
## [7] venneuler_1.1-0     rJava_0.9-8
##
## loaded via a namespace (and not attached):
##  [1] Rcpp_0.12.7     digest_0.6.10   assertthat_0.1  grid_3.3.0
##  [5] gtable_0.2.0    formatR_1.4     magrittr_1.5    evaluate_0.10
##  [9] stringi_1.1.2   rmarkdown_1.1   tools_3.3.0     stringr_1.1.0
## [13] yaml_2.1.13     htmltools_0.3.5 knitr_1.14      tibble_1.2
```
