# Supplementary material for: Single-cell transcriptomes identify human islet cell signatures and reveal cell-type–specific expression changes in type 2 diabetes
Source: Genome Res. 2017 Feb;27(2):208–22. doi: 10.1101/gr.212720.116 (PMC5287227; doi:10.1101/gr.212720.116)
Supplement: Supplemental Material [file supp_gr.212720.116_Supplemental_Methods_Source_Code.zip › Supplemental_Methods_Source_Code/Supplemental_Figure_Source_Code/Supplemental_Fig_S2_Source_Code.pdf]

Transcript Coverage of Single Cell Ensemble Data

Introduction

We ranked all genes by their log2 transcript per million (TPM) expression level within each cell type and grouped them into quartiles. Moderately expressed genes may be considered to reside in the 2nd and 3rd quartiles. We then used the program "CollectRNASeqMetrics" from Picard Tools (https://broadinstitute.github.io/picard/command-line-overview.html) to obtain and plot the normalized transcript coverage (y-axis) as a function of normalized transcript length (x-axis).

Transcript Coverage Plots

```
suppressPackageStartupMessages(library(RColorBrewer))
# Plot picard metrics
rm(list = ls())
library(RColorBrewer)
# Load in metric data for BIN 2 for each cell representative cell and bulk sample
setwd("/Users/lawlon/Documents/Final_RNA_Seq_3/TPM_vs_Coverage/")
beta <- read.delim2("Single_Cell_Picard_Metrics/beta.bin2.6th-C63_S26.metrics.txt",
                    header = T, check.names = F, row.names = NULL, skip = 10, stringsAsFactors = F)
alpha <- read.delim2("Single_Cell_Picard_Metrics/alpha.bin2.2nd-C11_S20.metrics.txt",
                     header = T, check.names = F, row.names = NULL, skip = 10, stringsAsFactors = F)
delta <- read.delim2("Single_Cell_Picard_Metrics/delta.bin2.7th-C72_S42.metrics.txt",
                     header = T, check.names = F, row.names = NULL, skip = 10, stringsAsFactors = F)
gamma <- read.delim2("Single_Cell_Picard_Metrics/gamma.bin2.3rd-C47_S35.metrics.txt",
                     header = T, check.names = F, row.names = NULL, skip = 10, stringsAsFactors = F)
epsilon <- read.delim2("Single_Cell_Picard_Metrics/epsilon.bin2.9th-C84_S60.metrics.txt",
                       header = T, check.names = F, row.names = NULL, skip = 10, stringsAsFactors = F)
stellate <- read.delim2("Single_Cell_Picard_Metrics/stel.bin2.1st-C42_S71.metrics.txt",
                        header = T, check.names = F, row.names = NULL, skip = 10, stringsAsFactors = F)
acinar <- read.delim2("Single_Cell_Picard_Metrics/acinar.bin2.5th-C56_S95.metrics.txt",
                      header = T, check.names = F, row.names = NULL, skip = 10, stringsAsFactors = F)
ductal <- read.delim2("Single_Cell_Picard_Metrics/ductal.bin2.9th-C61_S25.metrics.txt",
                      header = T, check.names = F, row.names = NULL, skip = 10, stringsAsFactors = F)
# combine data, all have same normalized position
empty <- beta
# combine all other coverage data
res2 <- cbind(empty, alpha[,2], delta[,2], gamma[,2], epsilon[,2], stellate[,2],
             acinar[,2], ductal[,2])
# change columns to numeric
for (j in 1:dim(res2)[2]) {
  res2[,j] <- as.numeric(as.character(res2[,j]))
}

# load in metrics for bin 3
setwd("/Users/lawlon/Documents/Final_RNA_Seq_3/TPM_vs_Coverage/")
beta <- read.delim2("Single_Cell_Picard_Metrics/beta.bin3.6th-C63_S26.metrics.txt",
                    header = T, check.names = F, row.names = NULL, skip = 10, stringsAsFactors = F)
alpha <- read.delim2("Single_Cell_Picard_Metrics/alpha.bin3.2nd-C11_S20.metrics.txt",
                     header = T, check.names = F, row.names = NULL, skip = 10, stringsAsFactors = F)
delta <- read.delim2("Single_Cell_Picard_Metrics/delta.bin3.7th-C72_S42.metrics.txt",
```

```r
                     header = T, check.names = F, row.names = NULL, skip = 10, stringsAsFactors = F)
gamma <- read.delim2("Single_Cell_Picard_Metrics/gamma.bin3.3rd-C47_S35.metrics.txt",
                     header = T, check.names = F, row.names = NULL, skip = 10, stringsAsFactors = F)
epsilon <- read.delim2("Single_Cell_Picard_Metrics/epsilon.bin3.9th-C84_S60.metrics.txt",
                       header = T, check.names = F, row.names = NULL, skip = 10, stringsAsFactors = F)
stellate <- read.delim2("Single_Cell_Picard_Metrics/stel.bin3.1st-C42_S71.metrics.txt",
                        header = T, check.names = F, row.names = NULL, skip = 10, stringsAsFactors = F)
acinar <- read.delim2("Single_Cell_Picard_Metrics/acinar.bin3.5th-C56_S95.metrics.txt",
                      header = T, check.names = F, row.names = NULL, skip = 10, stringsAsFactors = F)
ductal <- read.delim2("Single_Cell_Picard_Metrics/ductal.bin3.9th-C61_S25.metrics.txt",
                      header = T, check.names = F, row.names = NULL, skip = 10, stringsAsFactors = F)

# combine data, all have same normalized position
empty <- beta
# combine all other coverage data
res3 <- cbind(empty, alpha[,2], delta[,2], gamma[,2], epsilon[,2], stellate[,2],
             acinar[,2], ductal[,2])
# change columns to numeric
for (j in 1:dim(res3)[2]) {
  res3[,j] <- as.numeric(as.character(res3[,j]))
}

# read in data for bin4
beta <- read.delim2("Single_Cell_Picard_Metrics/beta.bin4.6th-C63_S26.metrics.txt",
                    header = T, check.names = F, row.names = NULL, skip = 10, stringsAsFactors = F)
alpha <- read.delim2("Single_Cell_Picard_Metrics/alpha.bin4.2nd-C11_S20.metrics.txt",
                     header = T, check.names = F, row.names = NULL, skip = 10, stringsAsFactors = F)
delta <- read.delim2("Single_Cell_Picard_Metrics/delta.bin4.7th-C72_S42.metrics.txt",
                     header = T, check.names = F, row.names = NULL, skip = 10, stringsAsFactors = F)
gamma <- read.delim2("Single_Cell_Picard_Metrics/gamma.bin4.3rd-C47_S35.metrics.txt",
                     header = T, check.names = F, row.names = NULL, skip = 10, stringsAsFactors = F)
epsilon <- read.delim2("Single_Cell_Picard_Metrics/epsilon.bin4.9th-C84_S60.metrics.txt",
                       header = T, check.names = F, row.names = NULL, skip = 10, stringsAsFactors = F)
stellate <- read.delim2("Single_Cell_Picard_Metrics/stel.bin4.1st-C42_S71.metrics.txt",
                        header = T, check.names = F, row.names = NULL, skip = 10, stringsAsFactors = F)
acinar <- read.delim2("Single_Cell_Picard_Metrics/acinar.bin4.5th-C56_S95.metrics.txt",
                      header = T, check.names = F, row.names = NULL, skip = 10, stringsAsFactors = F)
ductal <- read.delim2("Single_Cell_Picard_Metrics/ductal.bin4.9th-C61_S25.metrics.txt",
                      header = T, check.names = F, row.names = NULL, skip = 10, stringsAsFactors = F)

# combine data, all have same normalized position
empty <- beta
# combine all other coverage data
res4 <- cbind(empty, alpha[,2], delta[,2], gamma[,2], epsilon[,2], stellate[,2],
             acinar[,2], ductal[,2])
# change columns to numeric
for (j in 1:dim(res4)[2]) {
  res4[,j] <- as.numeric(as.character(res4[,j]))
}

# color panel
grey <- brewer.pal(n=9, name="Greys")
cols <- c("#e41a1c", "#e41a1c", "#377eb8", "#4daf4a", "#984ea3", "#ff7f00", grey[9], grey[7], grey[5])
```

```r
# make line plot (length vs coverage)
par(mfrow = c(1,3))
plot(x = res2$normalized_position, y = res2$All_Reads.normalized_coverage, col="white", xlab = "",
     ylab = "Normalized Coverage",
     main = "Coverage vs. Transcript Position \n (TPM 26-50th Percentile)", ylim = c(0,2))

for (i in 2:dim(res2)[2]) {
  lines(x = res2$normalized_position, y = res2[,i], col = cols[i], lwd = 2)
}

legend("topleft", legend = c("Beta", "Alpha", "Delta", "PP/Gamma", "Epsilon",
                             "Stellate", "Acinar", "Ductal"), text.col = cols[2:10], col = cols[2:10])

plot(x = res3$normalized_position, y = res3$All_Reads.normalized_coverage,
     col="white", xlab = "Normalized Distance Along Transcript",
     ylab = "", main = "Coverage vs. Transcript Position \n (TPM 51-75th Percentile)",
     ylim = c(0,2))

legend("topleft", legend = c("Beta", "Alpha", "Delta", "PP/Gamma", "Epsilon",
                             "Stellate", "Acinar", "Ductal"), text.col = cols[2:10], col = cols[2:10])

for (i in 2:dim(res3)[2]) {
  lines(x = res3$normalized_position, y = res3[,i], col = cols[i], lwd = 2)
}

plot(x = res4$normalized_position, y = res4$All_Reads.normalized_coverage,
     col="white", xlab = "", ylab = "",
     main = "Coverage vs. Transcript Position \n (TPM 76-100th Percentile)", ylim = c(0,2))

legend("topleft", legend = c("Beta", "Alpha", "Delta", "PP/Gamma", "Epsilon",
                             "Stellate", "Acinar", "Ductal"), text.col = cols[2:10], col = cols[2:10])

for (i in 2:dim(res4)[2]) {
  lines(x = res4$normalized_position, y = res4[,i], col = cols[i], lwd = 2)
}
```

Session Information

```r
suppressPackageStartupMessages(library(RColorBrewer))
rm(list = ls())
library(RColorBrewer)
sessionInfo()
```

```
## R version 3.3.0 (2016-05-03)
## Platform: x86_64-apple-darwin13.4.0 (64-bit)
## Running under: OS X 10.11.6 (El Capitan)
##
## locale:
## [1] en_US.UTF-8/en_US.UTF-8/en_US.UTF-8/C/en_US.UTF-8/en_US.UTF-8
##
## attached base packages:
## [1] stats     graphics  grDevices utils     datasets  methods   base
```

```
##
## other attached packages:
## [1] RColorBrewer_1.1-2
##
## loaded via a namespace (and not attached):
##  [1] magrittr_1.5    assertthat_0.1  formatR_1.4     tools_3.3.0
##  [5] htmltools_0.3.5 yaml_2.1.13     tibble_1.2      Rcpp_0.12.7
##  [9] stringi_1.1.2   rmarkdown_1.1   knitr_1.14      stringr_1.1.0
## [13] digest_0.6.10   evaluate_0.10
```
