# Supplementary material for: Single-cell transcriptomes identify human islet cell signatures and reveal cell-type–specific expression changes in type 2 diabetes
Source: Genome Res. 2017 Feb;27(2):208–22. doi: 10.1101/gr.212720.116 (PMC5287227; doi:10.1101/gr.212720.116)
Supplement: Supplemental Material [file supp_gr.212720.116_Supplemental_Methods_Source_Code.zip › Supplemental_Methods_Source_Code/Supplemental_Figure_Source_Code/Supplemental_Fig_S3_Source_Code.pdf]

Correlations of Single Cell Ensemble Transcriptomes

Introduction

For each patient islet sample, a collection of single cells were captured on Fluidigm C1 Chips and sequenced. These collections of single cells, or "ensemble" transcriptomes from each patient were highly correlated with each other (Pearson's R-squared ranged from 0.91-0.98), regardless of disease state.

Correlation of Ensemble Single Cell Transcriptomes Across Patients

```
suppressPackageStartupMessages(library(Biobase))
suppressPackageStartupMessages(library(gplots))
suppressPackageStartupMessages(library(ggplot2))
suppressPackageStartupMessages(library(pheatmap))
rm(list = ls())
library(Biobase)
library(gplots)
library(ggplot2)
library(pheatmap)
setwd("/Users/lawlon/Documents/Final_RNA_Seq/")
load("islet_bulk_uniq_data.rdata")
# Patient sample information
s.anns <- pData(bulk.cnts)
setwd("/Users/lawlon/Documents/Final_RNA_Seq_3/Data/")
# Load in all single cell data
load("nonT2D.rdata")
ND.anns <- pData(cnts.eset)
ND.cnts <- exprs(cnts.eset)
ND.data <- log2(ND.cnts+1)

load("T2D.rdata")
T2D.anns <- pData(cnts.eset)
T2D.cnts <- exprs(cnts.eset)
T2D.data <- log2(T2D.cnts+1)

# Get single cell data for each patient
pat1 <- ND.anns[ND.anns$run %in% c("1st", "2nd", "3rd"),]
pat1.exp <- ND.data[,rownames(pat1)]
pat2 <- ND.anns[ND.anns$run %in% c("5th"),]
pat2.exp <- ND.data[,rownames(pat2)]
pat3 <- ND.anns[ND.anns$run %in% c("6th", "7th"),]
pat3.exp <- ND.data[,rownames(pat3)]
pat4 <- ND.anns[ND.anns$run %in% c("8th"),]
pat4.exp <- ND.data[,rownames(pat4)]
pat5 <- ND.anns[ND.anns$run %in% c("9th"),]
pat5.exp <- ND.data[,rownames(pat5)]

pat6 <- T2D.anns[T2D.anns$run %in% c("4th"),]
pat6.exp <- T2D.data[,rownames(pat6)]
pat7 <- T2D.anns[T2D.anns$run %in% c("10t","11t"),]
pat7.exp <- T2D.data[,rownames(pat7)]
```

```r
pat8 <- T2D.anns[T2D.anns$run %in% c("12t","13t"),]
pat8.exp <- T2D.data[,rownames(pat8)]

# Plot the log2tpm of Patient 1 vs Patient 2, etc
setwd("/Users/lawlon/Documents/Final_RNA_Seq_3/Patient_Single_Cell_Correlations/")

groups <- c("P1", "P2", "P3", "P4", "P5", "P6", "P7", "P8")
# list of patient expression matrices
graphs <- list(pat1.exp, pat2.exp,pat3.exp, pat4.exp,
               pat5.exp, pat6.exp, pat7.exp, pat8.exp)

# Pairwise comparison number = N(N-1)/2
M = length(groups) -1
num = (M*(M+1))/2
cnames <- character(length = num)
idx <- 1

# Make scatter plots comparing single cell transcriptomes across patients
for(i in 1:M)
{
  N = i+1

  pdf(file = paste(groups[i], "Single.Cell.vs.all.Patients.log2TPM.pdf", sep = "."))
  par(mfrow = c(3,3), pty = "s", cex.lab = 0.75, cex.axis=0.75,
      mgp = c(1.25,0.5,0), mar = c(2.1,2,0.5,0.5))
  for(j in N:length(groups))
  {
    plot(x = rowMeans(graphs[[i]]), y = rowMeans(graphs[[j]]),
        xlab = paste(groups[i], "Log2(TPM)", sep= " "),
        ylab = paste(groups[j], "Log2(TPM)", sep = " "))
    Rsq <- cor(rowMeans(graphs[[i]]), rowMeans(graphs[[j]]))**2
    msg <- paste("Rsq = ",format(Rsq,digits=2),sep="")
    text(12,1, msg)
  }
  dev.off()
}

# Make a matrix of the Patient Rsq values
empty <- matrix(data = 0, nrow = 8, ncol = 8)
colnames(empty) <- groups
rownames(empty) <- groups

# Loop through the list of patient expression matrices, calculate Rsq values
for (i in 1:length(graphs)) {
  # loop through list again
  for (j in 1:length(graphs)) {
    rsq <- cor(rowMeans(graphs[[i]]), rowMeans(graphs[[j]]))**2
    empty[i,j] <- rsq
  }
}
# Round to two digits
res <- round(empty, digits = 2)
```

```r
setwd("/Users/lawlon/Documents/RNA-seq/RNA-seq Data/Bulk Islet Data/")
# Load sample annotation info
sample.anns.sel <- read.csv(file = "Unique Bulk Data/sample_anns_unique_bulk_samples.csv",
                            header = TRUE, check.names = FALSE, row.names = 1)
# Extract Patient id info
p1.id <- which(sample.anns.sel$`Patient Number` == "P1")
p2.id <- which(sample.anns.sel$`Patient Number` == "P2")
p3.id <- which(sample.anns.sel$`Patient Number` == "P3")
p4.id <- which(sample.anns.sel$`Patient Number` == "P4")
p5.id <- which(sample.anns.sel$`Patient Number` == "P5")
p6.id <- which(sample.anns.sel$`Patient Number` == "P6")
p7.id <- which(sample.anns.sel$`Patient Number` == "P7")
p8.id <- which(sample.anns.sel$`Patient Number` == "P8")

# Combine list of id's in order
ids <- c(p1.id[1], p2.id[1], p3.id[1], p4.id[1],
         p5.id[1], p6.id[1], p7.id[1], p8.id[1])

# Obtain shortened annotation matrix
anns <- sample.anns.sel[ids,]

#Data frame of samp id, type (baseline, intact, dissociate), sex, and race
annotation_col = data.frame(Sex = anns$Sex,
                            Race = anns$Race,
                            Phenotype = anns$Phenotype)
# Add sample labels to annotation data frame
rownames(annotation_col) = anns$`Patient Number`

pdf(file = "Patient.single.cell.Rsquared.heatmap.with.numbers.red.tpm.pdf",
    height = 8, width = 8)

pheatmap(mat = res, color = colorRampPalette(rev(brewer.pal(n = 7, name="RdYlBu")))(50),
         annotation_col = annotation_col, cluster_rows = FALSE,
         cluster_cols = FALSE, fontsize = 8,
         breaks = seq(0.5,1,length=50), display_numbers = TRUE)

dev.off()
```

Session Information

```r
suppressPackageStartupMessages(library(Biobase))
suppressPackageStartupMessages(library(gplots))
suppressPackageStartupMessages(library(ggplot2))
suppressPackageStartupMessages(library(pheatmap))
rm(list = ls())
library(Biobase)
library(gplots)
library(ggplot2)
library(pheatmap)
sessionInfo()
```

```
## R version 3.3.0 (2016-05-03)
```

```
## Platform: x86_64-apple-darwin13.4.0 (64-bit)
## Running under: OS X 10.11.6 (El Capitan)
##
## locale:
## [1] en_US.UTF-8/en_US.UTF-8/en_US.UTF-8/C/en_US.UTF-8/en_US.UTF-8
##
## attached base packages:
## [1] parallel  stats     graphics  grDevices utils     datasets  methods
## [8] base
##
## other attached packages:
## [1] pheatmap_1.0.8      ggplot2_2.1.0       gplots_3.0.1
## [4] Biobase_2.32.0      BiocGenerics_0.18.0
##
## loaded via a namespace (and not attached):
##  [1] Rcpp_0.12.7        knitr_1.14         magrittr_1.5
##  [4] munsell_0.4.3      colorspace_1.2-7   stringr_1.1.0
##  [7] plyr_1.8.4         caTools_1.17.1     tools_3.3.0
## [10] grid_3.3.0         gtable_0.2.0       KernSmooth_2.23-15
## [13] htmltools_0.3.5    gtools_3.5.0       yaml_2.1.13
## [16] assertthat_0.1     digest_0.6.10      tibble_1.2
## [19] RColorBrewer_1.1-2 formatR_1.4        bitops_1.0-6
## [22] evaluate_0.10      rmarkdown_1.1      gdata_2.17.0
## [25] stringi_1.1.2      scales_0.4.0
```
