# Supplementary material for: Single-cell transcriptomes identify human islet cell signatures and reveal cell-type–specific expression changes in type 2 diabetes
Source: Genome Res. 2017 Feb;27(2):208–22. doi: 10.1101/gr.212720.116 (PMC5287227; doi:10.1101/gr.212720.116)
Supplement: Supplemental Material [file supp_gr.212720.116_Supplemental_Methods_Source_Code.zip › Supplemental_Methods_Source_Code/Supplemental_Figure_Source_Code/Supplemental_Fig_S4_S5_Source_Code.pdf]

# Bulk vs Single Cell Ensemble Transcriptome Correlations

## Introduction

Pearson correlation between patient single cell ensembles and corresponding "bulk" intact transcriptomes ranged from 0.76-0.86 and did not differ substantially between ND (rsq=0.87) and T2D (rsq=0.85) samples.

## Correlation of Ensemble Single Cell and Patient Bulk Intact Transcriptomes

```
suppressPackageStartupMessages(library(Biobase))
suppressPackageStartupMessages(library(readxl))
suppressPackageStartupMessages(library(ggplot2))
library(Biobase)
library(ggplot2)
library(readxl)
rm(list = ls())
setwd("/Users/lawlon/Documents/Final_RNA_Seq_3/Data/")
# Load in single cell data
load("nonT2D.rdata")
sc <- exprs(cnts.eset)
ND.anns <- pData(cnts.eset)
sc.nonT2D.sel <- sc

load("T2D.rdata")
sc <- exprs(cnts.eset)
T2D.anns <- pData(cnts.eset)
sc.T2D.sel <- sc

# Load bulk data
load("/Users/lawlon/Documents/Final_RNA_Seq/islet_bulk_uniq_data.rdata")
bulk.anns <- read_excel(path = "/Users/lawlon/Documents/Final_RNA_Seq_3/Data/Supplemental_Table_S1_Pati
                        col_names = TRUE, skip = 1)
type <- which(bulk.anns$Type == "Intact")
bulk.sel <- bulk.anns[type,]
bulk <- exprs(bulk.cnts)
bulk <- bulk[,type]

pdf(file = "Patient_Intact_vs_SC_by_run_correlations.pdf")
par(mfrow=c(3,2))

# C1 SC vs Patient 1 Intact
c1.sc <- sc.nonT2D.sel[, ND.anns$run=="1st"]
pat1 <- bulk[,bulk.sel$`Patient Number`=="P1"]
pat1 <- log2(pat1+1)
c1.sc.r.mu <- apply(c1.sc,1,mean)
c1.sc.r.mu.log2 <- log2(c1.sc.r.mu+1)
plot(c1.sc.r.mu.log2, pat1, xlab = "Log2(TPM) C1 SC Ensemble", ylab = "Log2(TPM) P1 Intact")
rsq <- cor(c1.sc.r.mu.log2, pat1)**2
msg <- paste("Rsq = ",format(rsq,digits=2),sep="")
```

```r
text(13,2, msg)

# C2 SC vs Patient 1 Intact
c2.sc <- sc.nonT2D.sel[, ND.anns$run=="2nd"]
c2.sc.r.mu <- apply(c2.sc,1,mean)
c2.sc.r.mu.log2 <- log2(c2.sc.r.mu+1)
plot(c2.sc.r.mu.log2, pat1, xlab = "Log2(TPM) C2 SC Ensemble", ylab = "Log2(TPM) P1 Intact")
rsq <- cor(c2.sc.r.mu.log2, pat1)**2
msg <- paste("Rsq = ",format(rsq,digits=2),sep="")
text(13,2, msg)

# C3 SC vs Patient 1 Intact
c3.sc <- sc.nonT2D.sel[, ND.anns$run=="3rd"]
c3.sc.r.mu <- apply(c3.sc,1,mean)
c3.sc.r.mu.log2 <- log2(c3.sc.r.mu+1)
plot(c3.sc.r.mu.log2, pat1, xlab = "Log2(TPM) C3 SC Ensemble", ylab = "Log2(TPM) P1 Intact")
rsq <- cor(c3.sc.r.mu.log2, pat1)**2
msg <- paste("Rsq = ",format(rsq,digits=2),sep="")
text(13,2, msg)

# C5 SC vs Patient 2
c5.sc <- sc.nonT2D.sel[, ND.anns$run=="5th"]
c5.sc.r.mu <- apply(c5.sc,1,mean)
c5.sc.r.mu.log2 <- log2(c5.sc.r.mu+1)
pat2 <- bulk[,bulk.sel$`Patient Number`=="P2"]
pat2 <- log2(pat2+1)
plot(c5.sc.r.mu.log2, pat2, xlab = "Log2(TPM) C5 SC Ensemble", ylab = "Log2(TPM) P2 Intact")
rsq <- cor(c5.sc.r.mu.log2, pat2)**2
msg <- paste("Rsq = ",format(rsq,digits=2),sep="")
text(13,2, msg)

# C6 SC vs Patient 3
c6.sc <- sc.nonT2D.sel[, ND.anns$run=="6th"]
c6.sc.r.mu <- apply(c6.sc,1,mean)
c6.sc.r.mu.log2 <- log2(c6.sc.r.mu+1)
pat3 <- bulk[,bulk.sel$`Patient Number`=="P3"]
pat3 <- log2(pat3+1)
plot(c6.sc.r.mu.log2, pat3, xlab = "Log2(TPM) C6 SC Ensemble", ylab = "Log2(TPM) P3 Intact")
rsq <- cor(c6.sc.r.mu.log2, pat3)**2
msg <- paste("Rsq = ",format(rsq,digits=2),sep="")
text(13,2, msg)

# C7 vs Patient 3
c7.sc <- sc.nonT2D.sel[, ND.anns$run=="7th"]
c7.sc.r.mu <- apply(c7.sc,1,mean)
c7.sc.r.mu.log2 <- log2(c7.sc.r.mu+1)
pat3 <- bulk[,bulk.sel$`Patient Number`=="P3"]
pat3 <- log2(pat3+1)
plot(c7.sc.r.mu.log2, pat3, xlab = "Log2(TPM) C7 SC Ensemble", ylab = "Log2(TPM) P3 Intact")
rsq <- cor(c7.sc.r.mu.log2, pat3)**2
msg <- paste("Rsq = ",format(rsq,digits=2),sep="")
text(13,2, msg)
```

```
# C8 vs Patient 4
c8.sc <- sc.nonT2D.sel[, ND.anns$run=="8th"]
c8.sc.r.mu <- apply(c8.sc,1,mean)
c8.sc.r.mu.log2 <- log2(c8.sc.r.mu+1)
pat4 <- bulk[,bulk.sel$`Patient Number`=="P4"]
pat4 <- log2(pat4+1)
plot(c8.sc.r.mu.log2, pat4, xlab = "Log2(TPM) C8 SC Ensemble", ylab = "Log2(TPM) P4 Intact")
rsq <- cor(c8.sc.r.mu.log2, pat4)**2
msg <- paste("Rsq = ",format(rsq,digits=2),sep="")
text(13,2, msg)

# C9 vs Patient 5
c9.sc <- sc.nonT2D.sel[, ND.anns$run=="9th"]
c9.sc.r.mu <- apply(c9.sc,1,mean)
c9.sc.r.mu.log2 <- log2(c9.sc.r.mu+1)
pat5 <- bulk[,bulk.sel$`Patient Number`=="P5"]
pat5 <- log2(pat5+1)
plot(c9.sc.r.mu.log2, pat5, xlab = "Log2(TPM) C9 SC Ensemble", ylab = "Log2(TPM) P5 Intact")
rsq <- cor(c9.sc.r.mu.log2, pat5)**2
msg <- paste("Rsq = ",format(rsq,digits=2),sep="")
text(13,2, msg)

# C4 vs Patient 6 (T2D)
c4.sc <- sc.T2D.sel[, T2D.anns$run=="4th"]
c4.sc.r.mu <- apply(c4.sc,1,mean)
c4.sc.r.mu.log2 <- log2(c4.sc.r.mu+1)
pat6 <- bulk[,bulk.sel$`Patient Number`=="P6"]
pat6 <- log2(pat6+1)
plot(c4.sc.r.mu.log2, pat6, xlab = "Log2(TPM) C4 SC Ensemble", ylab = "Log2(TPM) P6 Intact")
rsq <- cor(c4.sc.r.mu.log2, pat6)**2
msg <- paste("Rsq = ",format(rsq,digits=2),sep="")
text(13,2, msg)

# C10 vs Patient 7 (T2D)
c10.sc <- sc.T2D.sel[, T2D.anns$run=="10t"]
c10.sc.r.mu <- apply(c10.sc,1,mean)
c10.sc.r.mu.log2 <- log2(c10.sc.r.mu+1)
pat7 <- bulk[,bulk.sel$`Patient Number`=="P7"]
pat7 <- log2(pat7+1)
plot(c10.sc.r.mu.log2, pat7, xlab = "Log2(TPM) C10 SC Ensemble", ylab = "Log2(TPM) P7 Intact")
rsq <- cor(c10.sc.r.mu.log2, pat7)**2
msg <- paste("Rsq = ",format(rsq,digits=2),sep="")
text(13,2, msg)

# C11 vs Patient 7 (T2D)
c11.sc <- sc.T2D.sel[, T2D.anns$run=="11t"]
c11.sc.r.mu <- apply(c11.sc,1,mean)
c11.sc.r.mu.log2 <- log2(c11.sc.r.mu+1)
plot(c11.sc.r.mu.log2, pat7, xlab = "Log2(TPM) C11 SC Ensemble", ylab = "Log2(TPM) P7 Intact")
rsq <- cor(c11.sc.r.mu.log2, pat7)**2
msg <- paste("Rsq = ",format(rsq,digits=2),sep="")
text(13,2, msg)
```

```r
# C12 vs Patient 8 (T2D)
c12.sc <- sc.T2D.sel[, T2D.anns$run=="12t"]
c12.sc.r.mu <- apply(c12.sc,1,mean)
c12.sc.r.mu.log2 <- log2(c12.sc.r.mu+1)
pat8 <- bulk[,bulk.sel$`Patient Number`=="P8"]
pat8 <- log2(pat8+1)
plot(c12.sc.r.mu.log2, pat8, xlab = "Log2(TPM) C12 SC Ensemble", ylab = "Log2(TPM) P8 Intact")
rsq <- cor(c12.sc.r.mu.log2, pat8)**2
msg <- paste("Rsq = ",format(rsq,digits=2),sep="")
text(13,2, msg)

# C13 vs Patient 8 (T2D)
c13.sc <- sc.T2D.sel[, T2D.anns$run=="13t"]
c13.sc.r.mu <- apply(c13.sc,1,mean)
c13.sc.r.mu.log2 <- log2(c13.sc.r.mu+1)
pat8 <- bulk[,bulk.sel$`Patient Number`=="P8"]
pat8 <- log2(pat8+1)
plot(c13.sc.r.mu.log2, pat8, xlab = "Log2(TPM) C13 SC Ensemble", ylab = "Log2(TPM) P8 Intact")
rsq <- cor(c13.sc.r.mu.log2, pat8)**2
msg <- paste("Rsq = ",format(rsq,digits=2),sep="")
text(13,2, msg)

dev.off()

# Make correlation plots of NonT2D and T2D bulk vs sc ensemble
pdf(file = "Patient_Intact_vs_SC_by_disease_correlations.pdf")
par(mfrow=c(1,2), pty="s")

# NonT2D
sc.nd <- sc.nonT2D.sel
sc.nd.r.mu <- apply(sc.nd,1,mean)
sc.nd.r.mu.log2 <- log2(sc.nd.r.mu+1)
nd.pat <- bulk[,bulk.sel$`Patient Number` %in% c("P1", "P2", "P3", "P4", "P5")]
nd.pat.r.mu <- apply(nd.pat,1,mean)
nd.pat.log2 <- log2(nd.pat.r.mu+1)
plot(sc.nd.r.mu.log2, nd.pat.log2, xlab = "Log2(TPM) NonT2D SC Ensemble", ylab = "Log2(TPM) NonT2D Inta
rsq <- cor(sc.nd.r.mu.log2, nd.pat.log2)**2
msg <- paste("Rsq = ",format(rsq,digits=2),sep="")
text(13,2, msg)

# T2D
sc.t2d <- sc.T2D.sel
sc.t2d.r.mu <- apply(sc.t2d,1,mean)
sc.t2d.r.mu.log2 <- log2(sc.t2d.r.mu+1)
t2d.pat <- bulk[,bulk.sel$`Patient Number` %in% c("P6", "P7", "P8")]
t2d.pat.r.mu <- apply(t2d.pat,1,mean)
t2d.pat.log2 <- log2(t2d.pat.r.mu+1)
plot(sc.t2d.r.mu.log2, t2d.pat.log2, xlab = "Log2(TPM) T2D SC Ensemble", ylab = "Log2(TPM) T2D Intact")
rsq <- cor(sc.t2d.r.mu.log2, t2d.pat.log2)**2
msg <- paste("Rsq = ",format(rsq,digits=2),sep="")
text(13,2, msg)

dev.off()
```

## Session Information

```
suppressPackageStartupMessages(library(Biobase))
suppressPackageStartupMessages(library(readxl))
suppressPackageStartupMessages(library(ggplot2))
rm(list = ls())
library(Biobase)
library(readxl)
library(ggplot2)
sessionInfo()
```

```
## R version 3.3.0 (2016-05-03)
## Platform: x86_64-apple-darwin13.4.0 (64-bit)
## Running under: OS X 10.11.6 (El Capitan)
##
## locale:
## [1] en_US.UTF-8/en_US.UTF-8/en_US.UTF-8/C/en_US.UTF-8/en_US.UTF-8
##
## attached base packages:
## [1] parallel  stats     graphics  grDevices utils     datasets  methods
## [8] base
##
## other attached packages:
## [1] ggplot2_2.1.0       readxl_0.1.1.9000   Biobase_2.32.0
## [4] BiocGenerics_0.18.0
##
## loaded via a namespace (and not attached):
##  [1] Rcpp_0.12.7      digest_0.6.10    assertthat_0.1   plyr_1.8.4
##  [5] grid_3.3.0       gtable_0.2.0     formatR_1.4      magrittr_1.5
##  [9] scales_0.4.0     evaluate_0.10    stringi_1.1.2    rmarkdown_1.1
## [13] tools_3.3.0      stringr_1.1.0    munsell_0.4.3    yaml_2.1.13
## [17] colorspace_1.2-7 htmltools_0.3.5  knitr_1.14       tibble_1.2
```
