# Supplementary material for: Single-cell transcriptomes identify human islet cell signatures and reveal cell-type–specific expression changes in type 2 diabetes
Source: Genome Res. 2017 Feb;27(2):208–22. doi: 10.1101/gr.212720.116 (PMC5287227; doi:10.1101/gr.212720.116)
Supplement: Supplemental Material [file supp_gr.212720.116_Supplemental_Methods_Source_Code.zip › Supplemental_Methods_Source_Code/Supplemental_Figure_Source_Code/Supplemental_Fig_S6_Source_Code.pdf]

3-Dimensional t-SNE Analysis of Non-diabetic Single Cell Ensemble Transcriptomes

Introduction

This file will detail the steps used to perform unsupervised t-SNE analysis on the non-diabetic single cell transcriptomes. The data was reduced to three dimensions in order to produce a 3D gif animation.

t-SNE Analysis

```
suppressPackageStartupMessages(library(Biobase))
suppressPackageStartupMessages(library(edgeR))
suppressPackageStartupMessages(library(Rtsne))
suppressPackageStartupMessages(library(scatterplot3d))
suppressPackageStartupMessages(library(rgl))
suppressPackageStartupMessages(library(pca3d))
suppressPackageStartupMessages(library(RColorBrewer))
library(Biobase)
library(edgeR)
library(Rtsne)
library(scatterplot3d)
library(rgl)
library(pca3d)
library(RColorBrewer)
rm(list = ls())
set.seed(125342)
# Load in Single Cell RNA-seq data
setwd("/Users/lawlon/Documents/Final_RNA_Seq_3/Data/")
load("nonT2D.rdata")
# Name of output file
name = "NonT2D.log2.cpm"
# Probe annotation data
p.anns <- as(featureData(cnts.eset), "data.frame")
# Sample annotation data
s.anns <- pData(cnts.eset)
# Remove multiples and keep all other groups
s.anns.sel <- s.anns[s.anns$cell.type %in% c("INS", "PPY", "GCG",
                              "SST", "COL1A1", "KRT19", "PRSS1", "none"),]
# Expression data
counts <- exprs(cnts.eset)

# Calculate cpm of data
cpm <- cpm(x = counts)
cpm.vals <- log2(cpm+1)
cpm.vals <- cpm.vals[,rownames(s.anns.sel)]

# Change name of one KRT19 cell to ghrelin cell
g <- which(p.anns$Associated.Gene.Name == "GHRL")
ghrl <- cpm.vals[g,]
samp <- which(ghrl > 15)
```

```r
g.idx <- which(rownames(s.anns.sel) == names(samp))

# Change sample anns of cell to GHRL
s.anns.sel$cell.type[g.idx] <- "GHRL"

r.max <- apply(cpm.vals,1,max)
# Use highly expressed genes for tSNE
cpm.sel <- cpm.vals[r.max > 10.5,]
# Transpose the matrix
cpm1 <- t(cpm.sel)
# Remove groups that are all zeros
df <- cpm1[,apply(cpm1, 2, var, na.rm=TRUE) != 0]
#Run tsne with defaults
rtsne_out <- Rtsne(as.matrix(df), dims = 3)

# Set rownames of matrix to tsne matrix
rownames(rtsne_out$Y) <- rownames(cpm1)

# write tsne matrix to file
write.csv(rtsne_out$Y, file = paste(name, "tsne.matrix.data.3D.csv", sep="."))

# Color Schema
grey <- brewer.pal(n=9, name="Greys")
colorCodes <- c(INS="#e41a1c", GCG = "#377eb8", SST = "#4daf4a",
                PPY = "#984ea3", GHRL = "#ff7f00",
                COL1A1 = grey[9], PRSS1 = grey[7], KRT19 = grey[5],
                none = grey[3])

namelist <- c("Beta", "Alpha", "Delta", "Gamma",
              "Epsilon", "Stellate", "Acinar", "Ductal", "none")


# Make variable of endocrine vs exocrine for 3D plot
type = NULL
for (i in 1:length(s.anns.sel$cell.type)){
  if ((s.anns.sel$cell.type[i] %in% c("INS","GCG","SST","PPY", "GHRL")) == TRUE) {
    idx = "sphere"
    type = c(type, idx)
  } else {
    idx = "tetrahedron"
    type = c(type, idx)
  }
}

# Match up cell type name with hormone type
cellnames = NULL
for (i in 1:length(s.anns.sel$cell.type)) {
  if (s.anns.sel$cell.type[i] %in% names(namelist) == TRUE) {
    cellnames = c(cellnames, as.character(namelist[s.anns.sel$cell.type[i]]))
  }
}

# Match up colors and hormone labels
```

```r
cols = NULL
for (i in 1:length(s.anns.sel$cell.type)) {
  if ((s.anns.sel$cell.type[i] %in% names(colorCodes)) == TRUE) {
    cols <- c(cols, colorCodes[s.anns.sel$cell.type[i]])
  }
}

# Match up cell name with hormone name
# Have cell type name and color
for (i in 1:length(cols)) {
  if (names(cols)[i] %in% names(namelist) == TRUE) {
    names(cols)[i] <- namelist[names(cols)[i]]
  }
}

# Make movie
pca3d(rtsne_out$Y, components = c(1:3), col = cols, title=name,
      legend = TRUE, new = TRUE, shape = type,
      axe.titles = c("t-SNE 1", "t-SNE 2", "t-SNE 3"))
legend3d("topright", c("Beta (INS)", "Alpha (GCG)",
              "Delta (SST)", "Gamma (PPY)", "Epsilon (GHRL)",
              "Stellate (COL1A1)", "Acinar (PRSS1)", "Ductal (KRT19)", "None"),
         text.col = colorCodes, pch = c(20,20,20,20,20,17,17,17,17), col = colorCodes)

#Take picture of the 3d ploot
rgl.snapshot(filename = paste(name,"3D.TSNE.plot.png", sep = "."), fmt = "png")

#Take pictures of multiple angles of the plot to create a gif
for (i in 1:360) {
  rgl.viewpoint(i, 20)
  filename <- paste(name, formatC(i, digits = 1, width = 3,
                                  flag = "0"), ".png", sep = "")
  rgl.snapshot(filename)
}

# Make a gif animation using ImageMagick
system(command = "convert -delay 10 *.png -loop 0 pic.gif")
```

Session Information

```r
suppressPackageStartupMessages(library(Biobase))
suppressPackageStartupMessages(library(edgeR))
```

```
## Warning: package 'limma' was built under R version 3.3.1
```

```r
suppressPackageStartupMessages(library(Rtsne))
suppressPackageStartupMessages(library(scatterplot3d))
suppressPackageStartupMessages(library(rgl))
suppressPackageStartupMessages(library(pca3d))
suppressPackageStartupMessages(library(RColorBrewer))
library(Biobase)
library(edgeR)
library(Rtsne)
```

```r
library(scatterplot3d)
library(rgl)
library(pca3d)
library(RColorBrewer)
sessionInfo()
```

```
## R version 3.3.0 (2016-05-03)
## Platform: x86_64-apple-darwin13.4.0 (64-bit)
## Running under: OS X 10.11.6 (El Capitan)
##
## locale:
## [1] en_US.UTF-8/en_US.UTF-8/en_US.UTF-8/C/en_US.UTF-8/en_US.UTF-8
##
## attached base packages:
## [1] parallel  stats     graphics  grDevices utils     datasets  methods
## [8] base
##
## other attached packages:
## [1] RColorBrewer_1.1-2   pca3d_0.8            rgl_0.96.0
## [4] scatterplot3d_0.3-37 Rtsne_0.11           edgeR_3.14.0
## [7] limma_3.28.21        Biobase_2.32.0       BiocGenerics_0.18.0
##
## loaded via a namespace (and not attached):
##  [1] Rcpp_0.12.7     knitr_1.14      magrittr_1.5    ellipse_0.3-8
##  [5] xtable_1.8-2    R6_2.2.0        stringr_1.1.0   tools_3.3.0
##  [9] htmltools_0.3.5 yaml_2.1.13     assertthat_0.1  digest_0.6.10
## [13] tibble_1.2      shiny_0.14.1    formatR_1.4     htmlwidgets_0.7
## [17] evaluate_0.10   mime_0.5        rmarkdown_1.1   stringi_1.1.2
## [21] jsonlite_1.1    httpuv_1.3.3
```
