# Supplementary material for: Single-cell transcriptomes identify human islet cell signatures and reveal cell-type–specific expression changes in type 2 diabetes
Source: Genome Res. 2017 Feb;27(2):208–22. doi: 10.1101/gr.212720.116 (PMC5287227; doi:10.1101/gr.212720.116)
Supplement: Supplemental Material [file supp_gr.212720.116_Supplemental_Methods_Source_Code.zip › Supplemental_Methods_Source_Code/Supplemental_Figure_Source_Code/Supplemental_Fig_S7_Source_Code.pdf]

t-SNE Analysis of Non-diabetic Single Cell Ensemble Transcriptomes Without Hormone Marker Genes

Introduction

This file will detail the steps used to perform unsupervised t-SNE analysis on the non-diabetic single cell transcriptomes without using pancreatic cell hormone marker genes (INS, GCG, SST, PPY, GHRL, COL1A1, PRSS1, and KRT19) as shown in Supplemental Fig S6. In addition, this file details steps used to color samples in the t-SNE plot by sequencing depth.

t-SNE Analysis without Hormone Markers

```
suppressPackageStartupMessages(library(Biobase))
suppressPackageStartupMessages(library(edgeR))
suppressPackageStartupMessages(library(Rtsne))
suppressPackageStartupMessages(library(RColorBrewer))
library(Biobase)
library(edgeR)
library(Rtsne)
library(RColorBrewer)
rm(list = ls())
set.seed(125342)
# Load in Single Cell RNA-seq data
setwd("/Users/lawlon/Documents/Final_RNA_Seq_3/Data/")
load("nonT2D.rdata")
# Probe annotation data
p.anns <- as(featureData(cnts.eset), "data.frame")
# Sample annotation data
s.anns <- pData(cnts.eset)
# Remove multiples and keep all other groups
s.anns.sel <- s.anns[s.anns$cell.type %in% c("INS", "PPY", "GCG", "SST",
                          "COL1A1", "KRT19", "PRSS1", "none"),]
# Expression data
counts <- exprs(cnts.eset)

# Calculate cpm of data
cpm <- cpm(x = counts)
cpm.vals <- log2(cpm+1)
cpm.vals <- cpm.vals[,rownames(s.anns.sel)]

# Change name of one KRT19 cell to ghrelin cell
g <- which(p.anns$Associated.Gene.Name == "GHRL")
ghrl <- cpm.vals[g,]
samp <- which(ghrl > 15)
g.idx <- which(rownames(s.anns.sel) == names(samp))

# Change sample anns of cell to GHRL
s.anns.sel$cell.type[g.idx] <- "GHRL"
r.max <- apply(cpm.vals,1,max)
# Use highly expressed genes
```

```
cpm.sel <- cpm.vals[r.max > 10.5,]

# Remove hormonal genes from list
horm <- which(p.anns$Associated.Gene.Name %in% c("INS","GCG","PPY","SST", "GHRL",
    "COL1A1", "PRSS1", "KRT19"))
ids <- rownames(p.anns)[horm]
indices <- which(rownames(cpm.sel) %in% ids)
cpm.sel <- cpm.sel[-indices,]

# Transpose the matrix
cpm1 <- t(cpm.sel)
# Remove groups that are all zeros
df <- cpm1[,apply(cpm1, 2, var, na.rm=TRUE) != 0]
#Run tsne with defaults
rtsne_out <- Rtsne(as.matrix(df), dims = 2)
# Set rownames of matrix to tsne matrix
rownames(rtsne_out$Y) <- rownames(cpm1)
# Write tsne matrix to file
write.csv(rtsne_out$Y, file = paste(name, "tsne.matrix.data.2D.csv", sep="."))

# Color Schema
grey <- brewer.pal(n=9, name="Greys")

colorCodes <- c(INS="#e41a1c", GCG = "#377eb8", SST = "#4daf4a",
                PPY = "#984ea3", GHRL = "#ff7f00",
                COL1A1 = grey[9], PRSS1 = grey[7], KRT19 = grey[5],
                none = grey[3])

namelist <- c("Beta", "Alpha", "Delta", "Gamma", "Epsilon",
              "Stellate", "Acinar", "Ductal", "none")

# Shapes for 2D plot
type1 <- NULL
for (i in 1:length(s.anns.sel$cell.type)){
  if ((s.anns.sel$cell.type[i] %in% c("INS","GCG","SST","PPY", "GHRL")) == TRUE) {
    idx = 20
    type1 = c(type1, idx)
  } else {
    idx = 17
    type1 = c(type1, idx)
  }
}

# Match up cell type name with hormone type
cellnames = NULL
for (i in 1:length(s.anns.sel$cell.type)) {
  if (s.anns.sel$cell.type[i] %in% names(namelist) == TRUE) {
    cellnames = c(cellnames, as.character(namelist[s.anns.sel$cell.type[i]]))
  }
}

# Match up colors and hormone labels
cols = NULL
```

```
for (i in 1:length(s.anns.sel$cell.type)) {
  if ((s.anns.sel$cell.type[i] %in% names(colorCodes)) == TRUE) {
    cols <- c(cols, colorCodes[s.anns.sel$cell.type[i]])
  }
}

# Match up cell name with hormone name
# Have cell type name and color
for (i in 1:length(cols)) {
  if (names(cols)[i] %in% names(namelist) == TRUE) {
    names(cols)[i] <- namelist[names(cols)[i]]
  }
}

# Plot the t-sne in 2-D
plot(rtsne_out$Y[,1], rtsne_out$Y[,2], col = cols, pch = type1,
     xlab = "t-SNE 1", ylab = "t-SNE 2")
legend("topleft", legend = as.character(namelist),
  text.col = colorCodes, pch = c(20,20,20,20,20,17,17,17,17), col = colorCodes,
  cex = 1)
```

t-SNE Coloring by Sequencing Depth

```
suppressPackageStartupMessages(library(Biobase))
suppressPackageStartupMessages(library(edgeR))
suppressPackageStartupMessages(library(readxl))
suppressPackageStartupMessages(library(RColorBrewer))
rm(list = ls())
library(RColorBrewer)
library(edgeR)
library(Biobase)
library(readxl)
# load in the t-SNE data used to make plot
setwd("/Users/lawlon/Documents/Final_RNA_Seq_3/TSNE_3/NonT2D/")
tsne <- read.csv("NonT2D.log2.cpm.tsne.matrix.data.2D.csv",
                 header = TRUE, row.names = 1, check.names = FALSE)

# Load in nonT2D data
setwd("/Users/lawlon/Documents/Final_RNA_Seq_3/Data/")
load("nonT2D.rdata")
s.anns <- pData(cnts.eset)
p.anns <- as(featureData(cnts.eset), "data.frame")
s.sel <- s.anns[s.anns$cell.type %in% c("INS", "GCG", "SST", "PPY",
                                        "COL1A1", "PRSS1", "KRT19", "none"),]

# read in sequencing depth supp table
depth <- read_excel(path = "/Users/lawlon/Documents/Final_RNA_Seq_3/Supplemental_Tables/Revised_Supp_Ta[illegible]
depth <- as.data.frame(depth)
rownames(depth) <- depth$sampleid
depth[,1] <- NULL

# color the cells by their sequencing depth
```

```
  # find samples in table
s.id <- NULL
for (i in 1:dim(s.sel)[1]) {
  samp.id <- which(rownames(depth) == rownames(s.sel)[i])
  s.id <- c(s.id, samp.id)
}

read.depth <- depth[s.id,]

num.reads <- data.frame(Name = rownames(read.depth),
                        Num.reads = read.depth$number_of_reads)
rownames(num.reads) <- rownames(s.sel)

# Sort the values
o <- rank(x = num.reads$Num.reads)
ordering <- data.frame(Name = rownames(s.sel), Order = o)
# find indices in sorted order
sort.id <- NULL
for (i in 1:length(ordering$Order)) {
  s.idx <- which(ordering$Order == i)
  sort.id <- c(sort.id, s.idx)
}

sorted.exp <- num.reads[sort.id, 2]
sorted.mat <- num.reads[sort.id,]

# short cells by their coverage value
beta.ids <- NULL
for (i in 1:length(sorted.exp)) {
  idx <- which(rownames(tsne) == rownames(sorted.mat)[i])
  beta.ids <- c(beta.ids, idx)
}

#Create a function to generate a continuous color palette
rbPal <- colorRampPalette(c('blue', 'yellow', 'red'))

#This adds a column of color values
Col <- rbPal(6)[as.numeric(cut(sorted.exp,breaks = 6))]
plot(tsne$V1[beta.ids], tsne$V2[beta.ids],pch = 20,col = Col,
     xlab = "t-SNE 1", ylab = "t-SNE 2", main = "")
cuts <- cut(sorted.exp, breaks = 6)
legend("bottomright",title="Read Depth",legend=levels(cuts),col =rbPal(6),pch=20)
```

Session Information

```
suppressPackageStartupMessages(library(Biobase))
suppressPackageStartupMessages(library(edgeR))
```

```
## Warning: package 'limma' was built under R version 3.3.1
```

```
suppressPackageStartupMessages(library(Rtsne))
suppressPackageStartupMessages(library(RColorBrewer))
library(Biobase)
```

```r
library(edgeR)
library(Rtsne)
library(RColorBrewer)

sessionInfo()
```

```
## R version 3.3.0 (2016-05-03)
## Platform: x86_64-apple-darwin13.4.0 (64-bit)
## Running under: OS X 10.11.6 (El Capitan)
##
## locale:
## [1] en_US.UTF-8/en_US.UTF-8/en_US.UTF-8/C/en_US.UTF-8/en_US.UTF-8
##
## attached base packages:
## [1] parallel  stats     graphics  grDevices utils     datasets  methods
## [8] base
##
## other attached packages:
## [1] RColorBrewer_1.1-2  Rtsne_0.11          edgeR_3.14.0
## [4] limma_3.28.21       Biobase_2.32.0      BiocGenerics_0.18.0
##
## loaded via a namespace (and not attached):
##  [1] Rcpp_0.12.7     digest_0.6.10   assertthat_0.1  formatR_1.4
##  [5] magrittr_1.5    evaluate_0.10   stringi_1.1.2   rmarkdown_1.1
##  [9] tools_3.3.0     stringr_1.1.0   yaml_2.1.13     htmltools_0.3.5
## [13] knitr_1.14      tibble_1.2
```
