# Supplementary material for: Single-cell transcriptomes identify human islet cell signatures and reveal cell-type–specific expression changes in type 2 diabetes
Source: Genome Res. 2017 Feb;27(2):208–22. doi: 10.1101/gr.212720.116 (PMC5287227; doi:10.1101/gr.212720.116)
Supplement: Supplemental Material [file supp_gr.212720.116_Supplemental_Methods_Source_Code.zip › Supplemental_Methods_Source_Code/Supplemental_Figure_Source_Code/Supplemental_Fig_S9_Source_Code.pdf]

Investigation of beta cell heterogeneity did not reveal distinct subpopulations

Introduction

Previously in Dorrell et al. 2016, four antigenic beta cell subtypes were identified by differing patterns of cell surface expression of CD9 and ST8SIA1 proteins: B1 (CD9-/ST8SIA1-), B2 (CD9+/ST8SIA1-), B3 (CD9-/ST8SIA1+), B4 (CD9+/ST8SIA1+). 29 genes were differentially expressed between subtypes B1/B2 and B3/B4, and 30 genes distinguished subtypes B1/B3 and B2/B4. Using the lists of differentially expressed genes between subtypes B1/B2 and B3/B4, we performed unsupervised t-SNE and hierarchical clustering analyses of all non-diabetic beta cell transcriptomes (n =168) to attempt to separate distinct sub-populations of CD9+/CD9- and ST8SIA+/ST8SIA- beta cells. In addition, we performed the same unsupervised analyses with the combined gene sets (59 genes) to attempt to identify the four described subpopulations. In Bader et al. 2016, they characterized proliferative (Fltp+/FVR+) and mature (Fltp-/FVR-) mouse beta cells that showed differential expression of 996 transcripts. Using the Mouse Genome Informatics (MGI; http://www.informatics.jax.org) database, these 996 transcripts corresponded to 768 genes. 726/768 genes corresponded to an MGI-annotated human orthologue, and 691 were detected/expressed in our human islet single cell data. These genes were used for unsupervised hierarchical clustering of all beta cell transcriptomes to attempty to identify mature human beta cell subpopulations.

Unsupervised t-SNE does not reveal distinct subpopulations of beta cells based on CD9 or ST8SIA1 genesets (as defined in Dorrell et al. 2016)

```
suppressPackageStartupMessages(library(Biobase))
suppressPackageStartupMessages(library(edgeR))
suppressPackageStartupMessages(library(Rtsne))
rm(list = ls())
library(Biobase)
library(edgeR)
library(Rtsne)
set.seed(125342)
# Load in Single Cell RNA-seq data
setwd("/Users/lawlon/Documents/Final_RNA_Seq_3/Data/")
load("nonT2D.rdata")
celltype <- "INS"
# Probe annotation data
p.anns <- as(featureData(cnts.eset), "data.frame")
# Sample annotation data
s.anns <- pData(cnts.eset)
s.anns.sel <- s.anns[s.anns$cell.type %in% celltype,]
# Expression data
counts <- exprs(cnts.eset)
counts <- counts[, rownames(s.anns.sel)]
# Calculate cpm of data
cpm <- edgeR::cpm(x = counts)
cpm.vals <- log2(cpm+1)

###### ANALYSIS FOR ST8SIA1 Geneset
# Load in gene set (ST8SIA1)
```

```
genlist <- read.csv("/Users/lawlon/Documents/Final_RNA_Seq_3/Subpopulations/Pheatmap_Clustering/Beta_ce
gen.sym <- genlist[,1]

# Get ensl ids for genes
en.ids <- NULL
for (j in 1:length(gen.sym)) {
  idz <- which(p.anns$Associated.Gene.Name == gen.sym[j])
  en.ids <- c(en.ids,idz)
}
cpm.sel <- cpm.vals[en.ids,]
# Transpose the matrix
cpm1 <- t(cpm.sel)
# Remove groups that are all zeros
df <- cpm1[,apply(cpm1, 2, var, na.rm=TRUE) != 0]
#Run tsne with defaults
rtsne_out <- Rtsne(as.matrix(df), dims = 2)
# Set rownames of matrix to tsne matrix
rownames(rtsne_out$Y) <- rownames(cpm1)
# specify gene of interest
sym <- "ST8SIA1"
gene.idx <- which(p.anns$Associated.Gene.Name == sym)
# color the cells by their number of expressed genes
num.genes <- data.frame(Name = rownames(s.anns.sel), Num.genes = cpm.vals[gene.idx,])
rownames(num.genes) <- rownames(s.anns.sel)
# Sort the values
o <- rank(x = num.genes$Num.genes, ties.method = "first")
ordering <- data.frame(Name = rownames(s.anns.sel), Order = o)
# find indices in sorted order
sort.id <- NULL
for (i in 1:length(ordering$Order)) {
  s.idx <- which(ordering$Order == i)
  sort.id <- c(sort.id, s.idx)
}
sorted.exp <- num.genes[sort.id, 2]
sorted.mat <- num.genes[sort.id,]
# Find the beta cells in the tsne data
beta.ids <- NULL
for (i in 1:length(sorted.exp)) {
  idx <- which(rownames(rtsne_out$Y) == rownames(sorted.mat)[i])
  beta.ids <- c(beta.ids, idx)
}
#Create a function to generate a continuous color palette
rbPal <- colorRampPalette(c('blue', 'yellow', 'red'))
Col <- rbPal(6)[as.numeric(cut(sorted.exp,breaks = 6))]
plot(rtsne_out$Y[beta.ids,1], rtsne_out$Y[beta.ids,2],pch = 20,col = Col,
     xlab = "t-SNE 1", ylab = "t-SNE 2", main = paste("Shaded by", sym, sep = " "))
cuts <- cut(sorted.exp, breaks = 6)
legend("bottomright",title="Log2 CPM Expression",legend=levels(cuts),col =rbPal(6),pch=20)

## Analysis for CD9 Geneset
genlist <- read.csv("/Users/lawlon/Documents/Final_RNA_Seq_3/Subpopulations/Pheatmap_Clustering/Beta_ce
                     header = F, check.names = F, row.names = NULL)
gen.sym <- genlist[,1]
```

```r
# Get ensl ids for genes
en.ids <- NULL
for (j in 1:length(gen.sym)) {
  idz <- which(p.anns$Associated.Gene.Name == gen.sym[j])
  en.ids <- c(en.ids,idz)
}
cpm.sel <- cpm.vals[en.ids,]
# Transpose the matrix
cpm1 <- t(cpm.sel)
# Remove groups that are all zeros
df <- cpm1[,apply(cpm1, 2, var, na.rm=TRUE) != 0]
#Run tsne with defaults
rtsne_out <- Rtsne(as.matrix(df), dims = 2)
# Set rownames of matrix to tsne matrix
rownames(rtsne_out$Y) <- rownames(cpm1)

# specify gene of interest
sym <- "CD9"
gene.idx <- which(p.anns$Associated.Gene.Name == sym)

# color the cells by their number of expressed genes
num.genes <- data.frame(Name = rownames(s.anns.sel), Num.genes = cpm.vals[gene.idx,])
rownames(num.genes) <- rownames(s.anns.sel)
# Sort the values
o <- rank(x = num.genes$Num.genes, ties.method = "first")
ordering <- data.frame(Name = rownames(s.anns.sel), Order = o)
# find indices in sorted order
sort.id <- NULL
for (i in 1:length(ordering$Order)) {
  s.idx <- which(ordering$Order == i)
  sort.id <- c(sort.id, s.idx)
}
sorted.exp <- num.genes[sort.id, 2]
sorted.mat <- num.genes[sort.id,]
# Find the beta cells in the tsne data
beta.ids <- NULL
for (i in 1:length(sorted.exp)) {
  idx <- which(rownames(rtsne_out$Y) == rownames(sorted.mat)[i])
  beta.ids <- c(beta.ids, idx)
}

#Create a function to generate a continuous color palette
rbPal <- colorRampPalette(c('blue', 'yellow', 'red'))
Col <- rbPal(6)[as.numeric(cut(sorted.exp,breaks = 6))]
plot(rtsne_out$Y[beta.ids,1], rtsne_out$Y[beta.ids,2],pch = 20,col = Col,
     xlab = "t-SNE 1", ylab = "t-SNE 2", main = paste("Shaded by", sym, sep = " "))
cuts <- cut(sorted.exp, breaks = 6)
legend("bottomright",title="Log2 CPM Expression",legend=levels(cuts),col =rbPal(6),pch=20)
```

Unsupervised hierarchical clustering does not reveal distinct subpopulations of beta cells (as defined by Dorrell et al. 2016)

```
rm(list = ls())
set.seed(53079239)
suppressPackageStartupMessages(library(edgeR))
suppressPackageStartupMessages(library(Biobase))
suppressPackageStartupMessages(library(RColorBrewer))
suppressPackageStartupMessages(library(pheatmap))
library(edgeR)
library(Biobase)
library(RColorBrewer)
library(pheatmap)
setwd("/Users/lawlon/Documents/Final_RNA_Seq_3/Data/")
load("nonT2D.rdata")
p.anns <- featureData(cnts.eset)
probe.anns <- as(p.anns,"data.frame")
ND.anns <- pData(cnts.eset)
# Remove multiples and keep all other groups
ND.sel <- ND.anns[ND.anns$cell.type %in% c("INS"),]
ND.counts <- exprs(cnts.eset)
cpms <- edgeR::cpm(x = ND.counts)
data <- log2(cpms+1)
data <- data[,rownames(ND.sel)]
# Combine sample anns and expression data
s.anns.sel <- ND.sel

#### load in ST8SIA1 gene set
genlist <- read.csv("/Users/lawlon/Documents/Final_RNA_Seq_3/Subpopulations/Pheatmap_Clustering/Beta_cel
gen.sym <- genlist[,1]

# Get ensl ids for genes
en.ids <- NULL
for (j in 1:length(gen.sym)) {
  idz <- which(probe.anns$Associated.Gene.Name == gen.sym[j])
  en.ids <- c(en.ids,idz)
}

ND.data.sel<- data[en.ids, rownames(ND.sel)]

# Change rownames back to symbols
rownames(ND.data.sel) <- gen.sym

# Save a copy of the data
exp.sel <- ND.data.sel
# Change column name labels to cell type
colnames(ND.data.sel)[1:dim(ND.sel)[1]] <- ND.sel$cell.type

# scaling of data
# Mean center by row (gene)
center_apply <- function(x) {
  apply(x, 1, function(y) y - mean(y))
}
```

```
mat.center <- center_apply(ND.data.sel)
mat.center <- t(mat.center)

# Scale the data between -1 and 1
nor.min.max <- function(x) {
  if (is.numeric(x) == FALSE) {
    stop("Please input numeric for x")
  }
  x.min <- min(x)
  x.max <- max(x)
  x <- 2*((x - x.min) / (x.max - x.min)) - 1
  return (x)
}

mat.scale <- t(apply(mat.center, 1, nor.min.max))
colnames(mat.scale) <- colnames(exp.sel)

# Annotation matrix
annotation_col = data.frame(Cell_Type = colnames(ND.data.sel))
rownames(annotation_col) <- colnames(exp.sel)

# Specify cell type colors
ann_colors <- list(
  Cell_Type = c(INS="#e41a1c"))

# Make heatmap
pheatmap(mat = mat.scale, cluster_rows = FALSE, cluster_cols = TRUE,
         color =  colorRampPalette(rev(brewer.pal(n = 7, name ="RdYlBu")))(20), annotation_col = annota[illegible]
         annotation_colors = ann_colors,
         clustering_distance_cols = "euclidean", clustering_distance_rows = "euclidean",
         clustering_method="ward.D2",show_rownames=TRUE,
         show_colnames = FALSE, annotation_names_row = FALSE, annotation_names_col = FALSE, trace = "no[illegible]
         annotation_legend = FALSE)

# do same analysis for CD9 gene set
genlist <- read.csv("/Users/lawlon/Documents/Final_RNA_Seq_3/Subpopulations/Pheatmap_Clustering/Beta_ce[illegible]
                    header = F, check.names = F, row.names = NULL)
gen.sym <- genlist[,1]
# Get ensl ids for genes
en.ids <- NULL
for (j in 1:length(gen.sym)) {
  idz <- which(probe.anns$Associated.Gene.Name == gen.sym[j])
  en.ids <- c(en.ids,idz)
}
ND.data.sel<- data[en.ids, rownames(ND.sel)]
# Change rownames back to symbols
rownames(ND.data.sel) <- gen.sym
# Save a copy of the data
exp.sel <- ND.data.sel
# Change column name labels to cell type
colnames(ND.data.sel)[1:dim(ND.sel)[1]] <- ND.sel$cell.type
# scaling of data
# Mean center by row (gene)
```

```
center_apply <- function(x) {
  apply(x, 1, function(y) y - mean(y))
}
mat.center <- center_apply(ND.data.sel)
mat.center <- t(mat.center)
# Scale the data between -1 and 1
mat.scale <- t(apply(mat.center, 1, nor.min.max))
colnames(mat.scale) <- colnames(exp.sel)

# Annotation matrix
annotation_col = data.frame(Cell_Type = colnames(ND.data.sel))
rownames(annotation_col) <- colnames(exp.sel)

# Specify cell type colors
ann_colors <- list(
  Cell_Type = c(INS="#e41a1c"))

# Make heatmap
pheatmap(mat = mat.scale, cluster_rows = FALSE, cluster_cols = TRUE,
         color =  colorRampPalette(rev(brewer.pal(n = 7, name ="RdYlBu")))(20), annotation_col = annotat
         annotation_colors = ann_colors,
         clustering_distance_cols = "euclidean", clustering_distance_rows = "euclidean",
         clustering_method="ward.D2",show_rownames=TRUE,
         show_colnames = FALSE, annotation_names_row = FALSE, annotation_names_col = FALSE, trace = "nor
         annotation_legend = FALSE)

# do same analysis for combined gene sets (CD9 and ST8SIA1 gene sets)
genlist <- read.csv("/Users/lawlon/Documents/Final_RNA_Seq_3/Subpopulations/Pheatmap_Clustering/Beta_cel
gen.sym <- genlist[,1]

# Get ensl ids for genes
en.ids <- NULL
for (j in 1:length(gen.sym)) {
  idz <- which(probe.anns$Associated.Gene.Name == gen.sym[j])
  en.ids <- c(en.ids,idz)
}

ND.data.sel<- data[en.ids, rownames(ND.sel)]
# Change rownames back to symbols
rownames(ND.data.sel) <- gen.sym
# Save a copy of the data
exp.sel <- ND.data.sel
# Change column name labels to cell type
colnames(ND.data.sel)[1:dim(ND.sel)[1]] <- ND.sel$cell.type
# scaling of data
# Mean center by row (gene)
center_apply <- function(x) {
  apply(x, 1, function(y) y - mean(y))
}
mat.center <- center_apply(ND.data.sel)
mat.center <- t(mat.center)

# Scale the data between -1 and 1
```

```
mat.scale <- t(apply(mat.center, 1, nor.min.max))
colnames(mat.scale) <- colnames(exp.sel)

# Annotation matrix
annotation_col = data.frame(Cell_Type = colnames(ND.data.sel))
rownames(annotation_col) <- colnames(exp.sel)

# Specify cell type colors
ann_colors <- list(
  Cell_Type = c(INS="#e41a1c"))

# Make heatmap
pheatmap(mat = mat.scale, cluster_rows = FALSE, cluster_cols = TRUE,
         color =  colorRampPalette(rev(brewer.pal(n = 7, name ="RdYlBu")))(20), annotation_col = annota
         annotation_colors = ann_colors,
         clustering_distance_cols = "euclidean", clustering_distance_rows = "euclidean",
         clustering_method="ward.D2",show_rownames=TRUE,
         show_colnames = FALSE, annotation_names_row = FALSE, annotation_names_col = FALSE, trace = "non
         annotation_legend = FALSE)
```

Unsupervised hierarchical clustering does not reveal distinct subpopulations of mature and proliferating beta cells (as defined in Bader et al. 2016)

```
suppressPackageStartupMessages(library(edgeR))
suppressPackageStartupMessages(library(Biobase))
suppressPackageStartupMessages(library(RColorBrewer))
suppressPackageStartupMessages(library(pheatmap))
library(edgeR)
library(Biobase)
library(RColorBrewer)
library(pheatmap)
rm(list = ls())

celltype <- "INS"
setwd("/Users/lawlon/Documents/Final_RNA_Seq_3/Data/")
load("nonT2D.rdata")
p.anns <- featureData(cnts.eset)
probe.anns <- as(p.anns,"data.frame")
ND.anns <- pData(cnts.eset)
# Remove multiples and keep all other groups
ND.sel <- ND.anns[ND.anns$cell.type %in% c(celltype),]
ND.counts <- exprs(cnts.eset)
ND.counts <- ND.counts[, rownames(ND.sel)]
ND.cpms <- edgeR::cpm(x = ND.counts)

all.cpms <- ND.cpms
all.log <- log2(all.cpms+1)
all.anns <- ND.sel

# Load in gene set
genelist <- read.csv("/Users/lawlon/Documents/Final_RNA_Seq_3/Subpopulations/Pheatmap_Clustering/Beta_c
# remove NA's
```

```r
genelist <- genelist[complete.cases(genelist),]
gen.dups <- duplicated(genelist[,3])
# remove duplicates
genelist <- genelist[!gen.dups,]

# Find gene symbols we have data for
gen.sel <- which(probe.anns$Associated.Gene.Name %in% genelist[,3])
gen.anns.sel <- probe.anns[gen.sel,]

data.sel <- all.log[gen.sel,]
sym.ids <- gen.anns.sel$Associated.Gene.Name

# Change rownames back to symbols
rownames(data.sel) <- sym.ids

# Save a copy of the data
exp.sel <- data.sel
# Change column name labels to cell type
colnames(data.sel)[1:dim(all.anns)[1]] <- all.anns$cell.type

# scaling of data
# Mean center by row (gene)
center_apply <- function(x) {
  apply(x, 1, function(y) y - mean(y))
}

mat.center <- center_apply(data.sel)
mat.center <- t(mat.center)

# Scale the data between -1 and 1
nor.min.max <- function(x) {
  if (is.numeric(x) == FALSE) {
    stop("Please input numeric for x")
  }
  x.min <- min(x)
  x.max <- max(x)
  x <- 2*((x - x.min) / (x.max - x.min)) - 1
  return (x)
}

mat.scale <- t(apply(mat.center, 1, nor.min.max))
colnames(mat.scale) <- colnames(exp.sel)

# Annotation matrix
annotation_col = data.frame(Cell_Type = colnames(data.sel))
rownames(annotation_col) <- colnames(exp.sel)

# Specify cell type colors
ann_colors <- list(Cell_Type = c(INS="#e41a1c"))

# Remove any cases of NAs
mat.scale <- mat.scale[complete.cases(mat.scale),]
pheatmap(mat = mat.scale, cluster_rows = TRUE, cluster_cols = TRUE,
```

```
        color =  colorRampPalette(rev(brewer.pal(n = 7, name ="RdYlBu")))(20), annotation_col = annota
        annotation_colors = ann_colors,
        clustering_distance_cols = "euclidean", clustering_distance_rows = "euclidean",
        clustering_method="ward.D2",show_rownames=FALSE,
        show_colnames = FALSE, annotation_names_row = FALSE, annotation_names_col = FALSE, trace = "no
        annotation_legend = TRUE)
```

Session Information

```
suppressPackageStartupMessages(library(edgeR))
```

```
## Warning: package 'limma' was built under R version 3.3.1
```

```
suppressPackageStartupMessages(library(Biobase))
suppressPackageStartupMessages(library(RColorBrewer))
suppressPackageStartupMessages(library(pheatmap))
suppressPackageStartupMessages(library(Rtsne))
library(edgeR)
library(Biobase)
library(RColorBrewer)
library(pheatmap)
library(Rtsne)
sessionInfo()
```

```
## R version 3.3.0 (2016-05-03)
## Platform: x86_64-apple-darwin13.4.0 (64-bit)
## Running under: OS X 10.11.6 (El Capitan)
##
## locale:
## [1] en_US.UTF-8/en_US.UTF-8/en_US.UTF-8/C/en_US.UTF-8/en_US.UTF-8
##
## attached base packages:
## [1] parallel  stats     graphics  grDevices utils     datasets  methods
## [8] base
##
## other attached packages:
## [1] Rtsne_0.11          pheatmap_1.0.8      RColorBrewer_1.1-2
## [4] Biobase_2.32.0      BiocGenerics_0.18.0 edgeR_3.14.0
## [7] limma_3.28.21
##
## loaded via a namespace (and not attached):
##  [1] Rcpp_0.12.7      digest_0.6.10    assertthat_0.1   plyr_1.8.4
##  [5] grid_3.3.0       gtable_0.2.0     formatR_1.4      magrittr_1.5
##  [9] scales_0.4.0     evaluate_0.10    stringi_1.1.2    rmarkdown_1.1
## [13] tools_3.3.0      stringr_1.1.0    munsell_0.4.3    yaml_2.1.13
## [17] colorspace_1.2-7 htmltools_0.3.5  knitr_1.14       tibble_1.2
```
