# Supplementary material for: Single-cell transcriptomes identify human islet cell signatures and reveal cell-type–specific expression changes in type 2 diabetes
Source: Genome Res. 2017 Feb;27(2):208–22. doi: 10.1101/gr.212720.116 (PMC5287227; doi:10.1101/gr.212720.116)
Supplement: Supplemental Material [file supp_gr.212720.116_Supplemental_Methods_Source_Code.zip › Supplemental_Methods_Source_Code/Supplemental_Table_Source_Code/Supplemental_Table_S11_Source_Code.pdf]

Investigating the specificity of Genome-wide association study (GWAS) and expression quantitative trait loci (eQTL) target gene expression in islet cell types

Introduction

This report will explain the steps used to determine if a list of GWAS target genes and reported cell specific genes are expressed uniquely or collectively in the different non-diabetic islet cell types. Analysis of variance (ANOVA) and post-hoc Tukey's Honest Significant Difference (HSD) tests were used to determine if there were statistically significant differences in expression of target genes between the islet cell populations. These methods were used to compare the mean expression of a specific gene in one islet cell type against the mean expression of that gene in another islet cell type. This process was repeated for every possible pairwise comparison.

Preprocessing Steps

For each gene examined, One-way analysis of variance (ANOVA) was used to determine if there were any statistically significant differences in average log2(CPM) expression of the gene in the four islet cell types (beta, alpha, delta, and gamma). Following this we performed a post hoc analysis using a Tukey's Honest Significant Difference (THSD) test to determine which islet cell types had significant differences in average expression of each gene. If the significant fold difference in average expression of a gene in an islet cell type compared to another cell type was greater than 4 (log2 > 2) and had an FDR-adjusted p-value less than 5%, that gene was considered to be significantly enriched in the respective cell group. This method was repeated for each gene to identify those that were expressed in a particular cell type or collection of islet cell types.

Genes that did not show any statistically significant differences in average expression based on our ANOVA test were classified as "pan-islet" if they had an average log2(CPM) expression greater than 4 in all islet cell types. Genes that were not determined to be cell type-specific or pan-islet were classified into two groups. If the average expression of the gene was log2CPM < 2 across all islet cell types, the gene was classified as "lowly expressed". The remaining genes that did not show enrichment in an islet cell type were classified as "$<$ 4-fold change".

Analysis of Variance (ANOVA) and Tukey Honest Significant Difference (HSD) Testing of Reported GWAS Genes

```
# Load libraries
rm(list = ls())
suppressPackageStartupMessages(library(Biobase))
suppressPackageStartupMessages(library(edgeR))
suppressPackageStartupMessages(library(readxl))
library(Biobase)
library(edgeR)
library(readxl)

setwd("/Users/lawlon/Documents/Final_RNA_Seq_3/Data/")
# name of data type and study
pheno <- "NonT2D"
study <- "All_GWAS_Genes"
```

```r
# Load in NonT2D single cell data
load("nonT2D.rdata")
s.anns <- pData(cnts.eset)
p.anns <- as(featureData(cnts.eset),"data.frame")
cnts <- exprs(cnts.eset)

# Calculate the cpm of the data
cpms <- cpm(x = cnts)
# Log transform
data <- log2(cpms+1)

# Obtain expression info for endocrine cells only
s.sel <- s.anns[s.anns$cell.type %in% c("INS", "GCG", "PPY", "SST"),]
exp.log2 <- data[, rownames(s.sel)]

# Load in gene list
setwd("/Users/lawlon/Documents/Final_RNA_Seq_3/Supplemental_Tables/")
genes <- read_excel(path = "Supplemental_Table_S10_Reported_GWAS_Genes_ANOVA.xlsx",
                    col_names = TRUE, sheet = 5)

# Choose the GWAS genes to study
genes.sel <- genes[,1]

p.anns.sel <- p.anns[p.anns$Associated.Gene.Name %in% genes.sel, ]
# Remove duplicate gene names
dups <- duplicated(p.anns.sel$Associated.Gene.Name)
p.anns.sel <- p.anns.sel[!dups,]

# Output lists of genes used in anova
#write.csv(x = p.anns.sel, file = paste(pheno,study, "genes.used.in.ANOVA.csv", sep = "."))

###### START OF ANOVA CODE
# Names for violin plot and ANOVA spreadsheet
fname <- paste(pheno,study, "logFC.filtered.adj.pval.expressed.genes.csv", sep=".")
vname <- paste(pheno,study, "log2CPM.violin.plots.pdf", sep = ".")

# effective size of genes
eff.size <- dim(p.anns.sel)[1]

# Obtain selected gene expression info for overlap genes
exp.log2.sel <- exp.log2[rownames(p.anns.sel),]
# Make ANOVA empty matrix
aov.res <- matrix(0,nrow= nrow(exp.log2.sel),ncol=13)

# ANOVA for loop
for(k in 1:nrow(exp.log2.sel)) {
  dat <- data.frame(exp = exp.log2.sel[k,],cell.type = s.sel$cell.type)
  results = aov(exp ~ cell.type, data=dat)
  p.val <- summary(results)[[1]][[5]][1]
  mc.res <- TukeyHSD(results, conf.level = 0.95)$cell.type
  aov.res[k,1] <- p.val
  aov.res[k,2:7] <- mc.res[,1]
  aov.res[k,8:13] <- mc.res[,4]
```

```
}

colnames(aov.res) <- c("p.value",paste("logFC",rownames(mc.res),sep="."),
                       paste("adjPvalue",rownames(mc.res),sep="."))
aov.res.ext <- data.frame(symbol = p.anns.sel$`Associated.Gene.Name`,aov.res)


#to count max logFC
r.max.fc <- apply(aov.res.ext[,3:8],1,max)
r.min.fc <- apply(aov.res.ext[,3:8],1,min)
#to select all genes with maximal fold change greatre than 2
# Matrix of genes with logFC that were at least greater than 2 in one comparison
aov.max <- aov.res.ext[r.max.fc > 2,]
aov.min <- aov.res.ext[r.min.fc < -2,]
aov.res.ext.sel <- rbind(aov.max, aov.min)

# Count all logFC greater than/equal to 2 or less than 2
fc.mat <- aov.res.ext[,3:8]
rownames(fc.mat) <- aov.res.ext$symbol
fc.bin <- fc.mat
fc.bin[fc.mat > -2 & fc.mat < 2] <- 0
fc.bin[fc.mat <= -2] <- 1
fc.bin[fc.mat >=2] <- 1
num.cells <- apply(fc.bin,1,sum)

#column order is INS, GCG, PPY, SST
# Count in how many different cells a gene is positively expressed
cell <- matrix(0,nrow = nrow(fc.mat),ncol=4)
# Logic for assigning expression for "first" cell groups
cell[fc.mat[,1] > 2,1] <- 1
cell[fc.mat[,2] > 2,3] <- 1
cell[fc.mat[,3] > 2,4] <- 1
cell[fc.mat[,4] > 2,3] <- 1
cell[fc.mat[,5] > 2,4] <- 1
cell[fc.mat[,6] > 2,4] <- 1

# Logic for assigning expression for "second" cell groups
cell[fc.mat[,1] < -2,2] <- 1
cell[fc.mat[,2] < -2,2] <- 1
cell[fc.mat[,3] < -2,2] <- 1
cell[fc.mat[,4] < -2,1] <- 1
cell[fc.mat[,5] < -2,1] <- 1
cell[fc.mat[,6] < -2,3] <- 1

colnames(cell) <- c("Beta", "Alpha", "Gamma", "Delta")
rownames(cell) <- aov.res.ext$symbol

# Remove duplicated gene symbols
dups <- duplicated(aov.res.ext.sel$symbol)
aov.res.ext.sel <- aov.res.ext.sel[!dups,]

n=1
idxs <- NULL
```

```r
for (n in 1:length(aov.res.ext.sel$symbol)) {
  vals <- which(aov.res.ext.sel$symbol[n] == rownames(cell))
  idxs <- c(idxs, vals)
}

cell.sel <- cell[idxs,]
i=1
nam <- NULL
for (i in 1:dim(cell.sel)[1]){
  colmz <- which(cell.sel[i,] == 1)
  if (length(colmz == 4)) {
    nam <- cbind(nam, paste(names(colmz)[1], names(colmz)[2],
                            names(colmz)[3], names(colmz)[4],sep="."))
  } else if (length(colmz == 3)) {
    nam <- cbind(nam, paste(names(colmz)[1], names(colmz)[2], names(colmz)[3],sep="."))
  } else if (length(colmz == 2)) {
    nam <- cbind(nam, paste(names(colmz)[1], names(colmz)[2], sep="."))
  }
}

# Remove "NA" from values
nam <- gsub(pattern = ".NA.NA.NA", replacement = "", nam)
nam <- gsub(pattern = ".NA.NA", replacement = "", nam)
nam <- gsub(pattern = ".NA", replacement = "", nam)
# combine names with multiples
nam1 <- t(nam)

cell.comb <- cbind(cell.sel, nam1)
colnames(cell.comb)[5] <- "Cell_Expressing_Gene"
cell.comb <- as.data.frame(cell.comb)

# Adjust the pvalue
adj.pval <- p.adjust(p=aov.res.ext.sel$p.value, "fdr")
adj.p <- which(adj.pval < 0.05)
aov.final <- aov.res.ext.sel[adj.p,]
rownames(aov.final) <- rownames(cell.comb)[adj.p]

cell.final <- cbind(cell.comb[adj.p,], aov.final[,2:14], adj.pval[adj.p])
colnames(cell.final)[19] <- "FDR.adj.p.value"
cell.final <- cell.final[, c(1:6,19,7:18)]

# Sort by which are in only one cell type
multis <- grepl(x = cell.final[,5], pattern = "\\w\\.", perl = TRUE)
# Extract only genes in single cell type
singles <- cell.final[!multis,]
multiples <- cell.final[multis,]

# Sort singles by cell type
beta.ids <- which(singles[,5] == "Beta")
alp.ids <- which(singles[,5] == "Alpha")
del.ids <- which(singles[,5] == "Delta")
gam.ids <- which(singles[,5] == "Gamma")
```

```
# Combine all ids in order
all.ids <- c(beta.ids, alp.ids, gam.ids, del.ids)
sing.ord <- singles[all.ids,]

# Sort the multiples by genes in 2 groups, then genes in 3
threes.id <- grepl(x = multiples[,5], pattern = "\\w\\.\\w*\\.", perl = TRUE)
twos <- multiples[!threes.id,]

# Find out which two cells express the genes
b.a.id <- which(twos[,5] == "Beta.Alpha")
b.d.id <- which(twos[,5] == "Beta.Delta")
b.g.id <- which(twos[,5] == "Beta.Gamma")
a.d.id <- which(twos[,5] == "Alpha.Delta")
a.g.id <- which(twos[,5] == "Alpha.Gamma")
g.d.id <- which(twos[,5] == "Gamma.Delta")
all.twos <- c(b.a.id, b.g.id, b.d.id, a.g.id, a.d.id, g.d.id)
twos <- twos[all.twos,]

# Find out which three cells express the genes
# Ordering was Beta, Alpha, Gamma,Delta
threes <- multiples[threes.id,]
b.a.d.id <- which(threes[,5] == "Beta.Alpha.Delta")
b.a.g.id <- which(threes[,5] == "Beta.Alpha.Gamma")
b.g.d.id <- which(threes[,5] == "Beta.Gamma.Delta")
a.g.d.id <- which(threes[,5] == "Alpha.Gamma.Delta")
all.threes <- c(b.a.d.id, b.a.g.id, b.g.d.id, a.g.d.id)
threes <- threes[all.threes,]
res <- rbind(sing.ord, twos,threes)

## Output final spreadsheet
write.csv(x=res, file = fname)

# Obtain stats of the ANOVA results (how many genes not
  #expressed in islets, how many pan-islet, how many specific)
ins <- which(s.sel$cell.type == "INS")
gcg <- which(s.sel$cell.type == "GCG")
sst <- which(s.sel$cell.type == "SST")
ppy <- which(s.sel$cell.type == "PPY")
# Obtain expression for each cell group
ins.exp <- exp.log2.sel[, ins]
gcg.exp <- exp.log2.sel[,gcg]
sst.exp <- exp.log2.sel[,sst]
ppy.exp <- exp.log2.sel[,ppy]
# Get average expression for each gene in each group
ins.rm <- rowMeans(ins.exp)
gcg.rm <- rowMeans(gcg.exp)
sst.rm <- rowMeans(sst.exp)
ppy.rm <- rowMeans(ppy.exp)

# Define pan-islet genes as those with average log2cpm expression > 4 in each cell type
pans <- which(ins.rm > 4 & gcg.rm > 4 & sst.rm > 4 & ppy.rm > 4)
pan.genes <- p.anns.sel$`Associated.Gene.Name`[pans]
# Make sure there are no genes being called pan from ANOVA list
```

```
pan.sel <- setdiff(pan.genes, rownames(res))

# Combine ANOVA and pan genes
comb <- union(pan.genes, rownames(res))
comb.ids <- which(p.anns.sel$`Associated.Gene.Name` %in% comb)
undet <- p.anns.sel$`Associated.Gene.Name`[-comb.ids]

# Find the  genes that are below expression threshold
undet.ids <- p.anns.sel[-comb.ids,]
sub.exp <- which(ins.rm[rownames(undet.ids)] < 2 & gcg.rm[rownames(undet.ids)] < 2 & sst.rm[rownames(und[illegible]
sub.exp.genes <- undet.ids$Associated.Gene.Name[sub.exp]

#  genes that are sub-fold-change threshold are the remaining genes
sub.fold <- undet.ids[-sub.exp,]
sub.fold.genes <- sub.fold$Associated.Gene.Name

# Report all stats: the number of genes in one, two, three cell types, pan-islet, not-exprssed
tab.stats <- matrix(data=0, nrow = 17, ncol = 3)
tab.stats[,1] <- c("Beta", "Alpha", "Delta", "Gamma",
                   "Beta.Alpha", "Beta.Delta", "Beta.Gamma", "Alpha.Delta",
                   "Alpha.Gamma", "Gamma.Delta", "Beta.Alpha.Delta",
                   "Beta.Alpha.Gamma", "Beta.Gamma.Delta", "Alpha.Gamma.Delta",
                   "Pan-Islet", "Sub-Expression-Threshold", "Sub-Fold-Change-Threshold")
tab.stats[,2] <- c(length(beta.ids), length(alp.ids), length(del.ids),
                   length(gam.ids), length(b.a.id), length(b.d.id), length(b.g.id), length(a.d.id),
                   length(a.g.id), length(g.d.id), length(b.a.d.id), length(b.a.g.id),
                   length(b.g.d.id), length(a.g.d.id), length(pan.sel), length(sub.exp.genes),
                   length(sub.fold.genes))

# Column three will contain gene symbols
# Loop through results table, get genes
for (i in 1:dim(tab.stats)[1]) {
  # which genes in beta
  betas <- which(res[,5] == tab.stats[i,1])
  beta.genes <- rownames(res)[betas]
  # Concatenate genes together
  nams <- ""
  for (n in 1:length(beta.genes)) {
    nams <- paste(beta.genes[n], nams, sep=",")
  }
  # Remove the last comma from the names
  beta.nams <- gsub(x = nams, pattern = "\\,+$", replacement = "")
  tab.stats[i,3] <- beta.nams
}

# Add pan-islet and  genes to table
nams <- ""
for (n in 1:length(pan.sel)) {
  nams <- paste(pan.sel[n], nams, sep=",")
}
# Remove the last comma from the names
pan.nams <- gsub(x = nams, pattern = "\\,+$", replacement = "")
tab.stats[15,3] <- pan.nams
```

```r
# Sub-expression threshold genes
nams <- ""
for (n in 1:length(sub.exp.genes)) {
  nams <- paste(sub.exp.genes[n], nams, sep=",")
}
# Remove the last comma from the names
sub.exp.nams <- gsub(x = nams, pattern = "\\,+$", replacement = "")
tab.stats[16,3] <- sub.exp.nams

# Sub-fold change threshold genes
nams <- ""
for (n in 1:length(sub.fold.genes)) {
  nams <- paste(sub.fold.genes[n], nams, sep=",")
}
# Remove the last comma from the names
sub.fold.nams <- gsub(x = nams, pattern = "\\,+$", replacement = "")
tab.stats[17,3] <- sub.fold.nams

# remove any rows which are zero
zeros <- which(tab.stats[,2] == 0)

if (length(zeros) > 0) {
  tab.sel <- tab.stats[-zeros,]
} else {
  tab.sel <- tab.stats
}

colnames(tab.sel) <- c("Expression in Endocrine Cells",
      paste("Number of Genes (out of total ", eff.size, " genes)", sep = ""), "Gene Symbol")

# Write ANOVA stats to file
write.csv(tab.sel, file = paste(pheno,study,"ANOVA.stats.csv", sep = "."))
# Write pan-islet genes and other genes to files
write.csv(pan.sel, file = paste(pheno,study, "Pan.islet.genes.csv", sep = "."))
write.csv(sub.exp.genes, file = paste(pheno, study, "sub.expression.threshold.genes.csv", sep = "."))
write.csv(sub.fold.genes, file = paste(pheno, study, "sub.fold.change.threshold.genes.csv", sep = "."))
```

Analysis of Variance (ANOVA) and Tukey Honest Significant Difference (HSD) Testing of Reported eQTL Genes

```r
# Load libraries
rm(list = ls())
suppressPackageStartupMessages(library(Biobase))
suppressPackageStartupMessages(library(edgeR))
suppressPackageStartupMessages(library(readxl))
library(Biobase)
library(edgeR)
library(readxl)

setwd("/Users/lawlon/Documents/Final_RNA_Seq_3/Data/")
# name of data type and study
```

```
pheno <- "NonT2D"
study <- "All_eQTL_Genes"
# Load in NonT2D single cell data
load("nonT2D.rdata")
s.anns <- pData(cnts.eset)
p.anns <- as(featureData(cnts.eset),"data.frame")
cnts <- exprs(cnts.eset)

# Calculate the cpm of the data
cpms <- cpm(x = cnts)
# Log transform
data <- log2(cpms+1)

# Obtain expression info for endocrine cells only
s.sel <- s.anns[s.anns$cell.type %in% c("INS", "GCG", "PPY", "SST"),]
exp.log2 <- data[, rownames(s.sel)]

# Load in gene list
setwd("/Users/lawlon/Documents/Final_RNA_Seq_3/Supplemental_Tables/")
genes <- read_excel(path = "Supplemental_Table_S9_Reported_GWAS_Genes_ANOVA.xlsx",
                    col_names = TRUE, sheet = 6)

# Choose the GWAS genes to study
genes.sel <- genes[,1]

p.anns.sel <- p.anns[p.anns$Associated.Gene.Name %in% genes.sel, ]
# Remove duplicate gene names
dups <- duplicated(p.anns.sel$Associated.Gene.Name)
p.anns.sel <- p.anns.sel[!dups,]

# Output lists of genes used in anova
#write.csv(x = p.anns.sel, file = paste(pheno,study, "genes.used.in.ANOVA.csv", sep = "."))

###### START OF ANOVA CODE
# Names for violin plot and ANOVA spreadsheet
fname <- paste(pheno,study, "logFC.filtered.adj.pval.expressed.genes.csv", sep=".")
vname <- paste(pheno,study, "log2CPM.violin.plots.pdf", sep = ".")

# effective size of genes
eff.size <- dim(p.anns.sel)[1]

# Obtain selected gene expression info for overlap genes
exp.log2.sel <- exp.log2[rownames(p.anns.sel),]
# Make ANOVA empty matrix
aov.res <- matrix(0,nrow= nrow(exp.log2.sel),ncol=13)

# ANOVA for loop
for(k in 1:nrow(exp.log2.sel)) {
  dat <- data.frame(exp = exp.log2.sel[k,],cell.type = s.sel$cell.type)
  results = aov(exp ~ cell.type, data=dat)
  p.val <- summary(results)[[1]][[5]][1]
  mc.res <- TukeyHSD(results, conf.level = 0.95)$cell.type
  aov.res[k,1] <- p.val
```

```r
  aov.res[k,2:7] <- mc.res[,1]
  aov.res[k,8:13] <- mc.res[,4]
}

colnames(aov.res) <- c("p.value",paste("logFC",rownames(mc.res),sep="."),
                       paste("adjPvalue",rownames(mc.res),sep="."))
aov.res.ext <- data.frame(symbol = p.anns.sel$`Associated.Gene.Name`,aov.res)


#to count max logFC
r.max.fc <- apply(aov.res.ext[,3:8],1,max)
r.min.fc <- apply(aov.res.ext[,3:8],1,min)
#to select all genes with maximal fold change greatre than 2
# Matrix of genes with logFC that were at least greater than 2 in one comparison
aov.max <- aov.res.ext[r.max.fc > 2,]
aov.min <- aov.res.ext[r.min.fc < -2,]
aov.res.ext.sel <- rbind(aov.max, aov.min)

# Count all logFC greater than/equal to 2 or less than 2
fc.mat <- aov.res.ext[,3:8]
rownames(fc.mat) <- aov.res.ext$symbol
fc.bin <- fc.mat
fc.bin[fc.mat > -2 & fc.mat < 2] <- 0
fc.bin[fc.mat <= -2] <- 1
fc.bin[fc.mat >=2] <- 1
num.cells <- apply(fc.bin,1,sum)

#column order is INS, GCG, PPY, SST
# Count in how many different cells a gene is positively expressed
cell <- matrix(0,nrow = nrow(fc.mat),ncol=4)
# Logic for assigning expression for "first" cell groups
cell[fc.mat[,1] > 2,1] <- 1
cell[fc.mat[,2] > 2,3] <- 1
cell[fc.mat[,3] > 2,4] <- 1
cell[fc.mat[,4] > 2,3] <- 1
cell[fc.mat[,5] > 2,4] <- 1
cell[fc.mat[,6] > 2,4] <- 1

# Logic for assigning expression for "second" cell groups
cell[fc.mat[,1] < -2,2] <- 1
cell[fc.mat[,2] < -2,2] <- 1
cell[fc.mat[,3] < -2,2] <- 1
cell[fc.mat[,4] < -2,1] <- 1
cell[fc.mat[,5] < -2,1] <- 1
cell[fc.mat[,6] < -2,3] <- 1

colnames(cell) <- c("Beta", "Alpha", "Gamma", "Delta")
rownames(cell) <- aov.res.ext$symbol

# Remove duplicated gene symbols
dups <- duplicated(aov.res.ext.sel$symbol)
aov.res.ext.sel <- aov.res.ext.sel[!dups,]
```

```r
n=1
idxs <- NULL
for (n in 1:length(aov.res.ext.sel$symbol)) {
  vals <- which(aov.res.ext.sel$symbol[n] == rownames(cell))
  idxs <- c(idxs, vals)
}

cell.sel <- cell[idxs,]
i=1
nam <- NULL
for (i in 1:dim(cell.sel)[1]){
  colmz <- which(cell.sel[i,] == 1)
  if (length(colmz == 4)) {
    nam <- cbind(nam, paste(names(colmz)[1], names(colmz)[2],
                            names(colmz)[3], names(colmz)[4],sep="."))
  } else if (length(colmz == 3)) {
    nam <- cbind(nam, paste(names(colmz)[1], names(colmz)[2], names(colmz)[3],sep="."))
  } else if (length(colmz == 2)) {
    nam <- cbind(nam, paste(names(colmz)[1], names(colmz)[2], sep="."))
  }
}

# Remove "NA" from values
nam <- gsub(pattern = ".NA.NA.NA", replacement = "", nam)
nam <- gsub(pattern = ".NA.NA", replacement = "", nam)
nam <- gsub(pattern = ".NA", replacement = "", nam)
# combine names with multiples
nam1 <- t(nam)

cell.comb <- cbind(cell.sel, nam1)
colnames(cell.comb)[5] <- "Cell_Expressing_Gene"
cell.comb <- as.data.frame(cell.comb)

# Adjust the pvalue
adj.pval <- p.adjust(p=aov.res.ext.sel$p.value, "fdr")
adj.p <- which(adj.pval < 0.05)
aov.final <- aov.res.ext.sel[adj.p,]
rownames(aov.final) <- rownames(cell.comb)[adj.p]

cell.final <- cbind(cell.comb[adj.p,], aov.final[,2:14], adj.pval[adj.p])
colnames(cell.final)[19] <- "FDR.adj.p.value"
cell.final <- cell.final[, c(1:6,19,7:18)]

# Sort by which are in only one cell type
multis <- grepl(x = cell.final[,5], pattern = "\\w\\.", perl = TRUE)
# Extract only genes in single cell type
singles <- cell.final[!multis,]
multiples <- cell.final[multis,]

# Sort singles by cell type
beta.ids <- which(singles[,5] == "Beta")
alp.ids <- which(singles[,5] == "Alpha")
del.ids <- which(singles[,5] == "Delta")
```

```r
gam.ids <- which(singles[,5] == "Gamma")

# Combine all ids in order
all.ids <- c(beta.ids, alp.ids, gam.ids, del.ids)
sing.ord <- singles[all.ids,]

# Sort the multiples by genes in 2 groups, then genes in 3
threes.id <- grepl(x = multiples[,5], pattern = "\\w\\.\\w*\\.", perl = TRUE)
twos <- multiples[!threes.id,]

# Find out which two cells express the genes
b.a.id <- which(twos[,5] == "Beta.Alpha")
b.d.id <- which(twos[,5] == "Beta.Delta")
b.g.id <- which(twos[,5] == "Beta.Gamma")
a.d.id <- which(twos[,5] == "Alpha.Delta")
a.g.id <- which(twos[,5] == "Alpha.Gamma")
g.d.id <- which(twos[,5] == "Gamma.Delta")
all.twos <- c(b.a.id, b.g.id, b.d.id, a.g.id, a.d.id, g.d.id)
twos <- twos[all.twos,]

# Find out which three cells express the genes
# Ordering was Beta, Alpha, Gamma,Delta
threes <- multiples[threes.id,]
b.a.d.id <- which(threes[,5] == "Beta.Alpha.Delta")
b.a.g.id <- which(threes[,5] == "Beta.Alpha.Gamma")
b.g.d.id <- which(threes[,5] == "Beta.Gamma.Delta")
a.g.d.id <- which(threes[,5] == "Alpha.Gamma.Delta")
all.threes <- c(b.a.d.id, b.a.g.id, b.g.d.id, a.g.d.id)
threes <- threes[all.threes,]
res <- rbind(sing.ord, twos,threes)

## Output final spreadsheet
write.csv(x=res, file = fname)

# Obtain stats of the ANOVA results (how many genes not expressed in islets,
  #how many pan-islet, how many specific)
ins <- which(s.sel$cell.type == "INS")
gcg <- which(s.sel$cell.type == "GCG")
sst <- which(s.sel$cell.type == "SST")
ppy <- which(s.sel$cell.type == "PPY")
# Obtain expression for each cell group
ins.exp <- exp.log2.sel[, ins]
gcg.exp <- exp.log2.sel[,gcg]
sst.exp <- exp.log2.sel[,sst]
ppy.exp <- exp.log2.sel[,ppy]
# Get average expression for each gene in each group
ins.rm <- rowMeans(ins.exp)
gcg.rm <- rowMeans(gcg.exp)
sst.rm <- rowMeans(sst.exp)
ppy.rm <- rowMeans(ppy.exp)

# Define pan-islet genes as those with average log2cpm expression > 4 in each cell type
pans <- which(ins.rm > 4 & gcg.rm > 4 & sst.rm > 4 & ppy.rm > 4)
```

```
pan.genes <- p.anns.sel$`Associated.Gene.Name`[pans]
# Make sure there are no genes being called pan from ANOVA list
pan.sel <- setdiff(pan.genes, rownames(res))

# Call the other genes as
# Combine ANOVA and pan genes
comb <- union(pan.genes, rownames(res))
comb.ids <- which(p.anns.sel$`Associated.Gene.Name` %in% comb)
undet <- p.anns.sel$`Associated.Gene.Name`[-comb.ids]

# Find the  genes that are below expression threshold
undet.ids <- p.anns.sel[-comb.ids,]
sub.exp <- which(ins.rm[rownames(undet.ids)] < 2 & gcg.rm[rownames(undet.ids)] < 2 & sst.rm[rownames(und
sub.exp.genes <- undet.ids$Associated.Gene.Name[sub.exp]

#  genes that are sub-fold-change threshold are the remaining genes
sub.fold <- undet.ids[-sub.exp,]
sub.fold.genes <- sub.fold$Associated.Gene.Name

# Report all stats: the number of genes in one, two, three cell types, pan-islet, not-exprssed
tab.stats <- matrix(data=0, nrow = 17, ncol = 3)
tab.stats[,1] <- c("Beta", "Alpha", "Delta", "Gamma",
                   "Beta.Alpha", "Beta.Delta", "Beta.Gamma", "Alpha.Delta",
                   "Alpha.Gamma", "Gamma.Delta", "Beta.Alpha.Delta",
                   "Beta.Alpha.Gamma", "Beta.Gamma.Delta", "Alpha.Gamma.Delta",
                   "Pan-Islet", "Sub-Expression-Threshold", "Sub-Fold-Change-Threshold")
tab.stats[,2] <- c(length(beta.ids), length(alp.ids), length(del.ids),
                   length(gam.ids), length(b.a.id), length(b.d.id), length(b.g.id), length(a.d.id),
                   length(a.g.id), length(g.d.id), length(b.a.d.id), length(b.a.g.id),
                   length(b.g.d.id), length(a.g.d.id), length(pan.sel), length(sub.exp.genes),
                   length(sub.fold.genes))

# Column three will contain gene symbols
# Loop through results table, get genes
for (i in 1:dim(tab.stats)[1]) {
  # which genes in beta
  betas <- which(res[,5] == tab.stats[i,1])
  beta.genes <- rownames(res)[betas]
  # Concatenate genes together
  nams <- ""
  for (n in 1:length(beta.genes)) {
    nams <- paste(beta.genes[n], nams, sep=",")
  }
  # Remove the last comma from the names
  beta.nams <- gsub(x = nams, pattern = "\\,+$", replacement = "")
  tab.stats[i,3] <- beta.nams
}

# Add pan-islet and  genes to table
nams <- ""
for (n in 1:length(pan.sel)) {
  nams <- paste(pan.sel[n], nams, sep=",")
}
```

```r
# Remove the last comma from the names
pan.nams <- gsub(x = nams, pattern = "\\,+$", replacement = "")
tab.stats[15,3] <- pan.nams

# Sub-expression threshold genes
nams <- ""
for (n in 1:length(sub.exp.genes)) {
  nams <- paste(sub.exp.genes[n], nams, sep=",")
}
# Remove the last comma from the names
sub.exp.nams <- gsub(x = nams, pattern = "\\,+$", replacement = "")
tab.stats[16,3] <- sub.exp.nams

# Sub-fold change threshold genes
nams <- ""
for (n in 1:length(sub.fold.genes)) {
  nams <- paste(sub.fold.genes[n], nams, sep=",")
}
# Remove the last comma from the names
sub.fold.nams <- gsub(x = nams, pattern = "\\,+$", replacement = "")
tab.stats[17,3] <- sub.fold.nams

# remove any rows which are zero
zeros <- which(tab.stats[,2] == 0)

if (length(zeros) > 0) {
  tab.sel <- tab.stats[-zeros,]
} else {
  tab.sel <- tab.stats
}

colnames(tab.sel) <- c("Expression in Endocrine Cells",
        paste("Number of Genes (out of total ", eff.size, " genes)", sep = ""), "Gene Symbol")

# Write ANOVA stats to file
write.csv(tab.sel, file = paste(pheno,study,"ANOVA.stats.csv", sep = "."))
# Write pan-islet genes and other genes to files
write.csv(pan.sel, file = paste(pheno,study, "Pan.islet.genes.csv", sep = "."))
write.csv(sub.exp.genes, file = paste(pheno, study, "sub.expression.threshold.genes.csv", sep = "."))
write.csv(sub.fold.genes, file = paste(pheno, study, "sub.fold.change.threshold.genes.csv", sep = "."))
```

Session Information

```r
rm(list = ls())
suppressPackageStartupMessages(library(Biobase))
suppressPackageStartupMessages(library(edgeR))
```

```
## Warning: package 'limma' was built under R version 3.3.1
```

```r
suppressPackageStartupMessages(library(readxl))
library(Biobase)
library(edgeR)
library(readxl)
```

```r
sessionInfo()
```

```
## R version 3.3.0 (2016-05-03)
## Platform: x86_64-apple-darwin13.4.0 (64-bit)
## Running under: OS X 10.11.6 (El Capitan)
##
## locale:
## [1] en_US.UTF-8/en_US.UTF-8/en_US.UTF-8/C/en_US.UTF-8/en_US.UTF-8
##
## attached base packages:
## [1] parallel  stats     graphics  grDevices utils     datasets  methods
## [8] base
##
## other attached packages:
## [1] readxl_0.1.1.9000   edgeR_3.14.0        limma_3.28.21
## [4] Biobase_2.32.0      BiocGenerics_0.18.0
##
## loaded via a namespace (and not attached):
##  [1] Rcpp_0.12.7     digest_0.6.10   assertthat_0.1  formatR_1.4
##  [5] magrittr_1.5    evaluate_0.10   stringi_1.1.2   rmarkdown_1.1
##  [9] tools_3.3.0     stringr_1.1.0   yaml_2.1.13     htmltools_0.3.5
## [13] knitr_1.14      tibble_1.2
```
