# Supplementary material for: Single-cell transcriptomes identify human islet cell signatures and reveal cell-type–specific expression changes in type 2 diabetes
Source: Genome Res. 2017 Feb;27(2):208–22. doi: 10.1101/gr.212720.116 (PMC5287227; doi:10.1101/gr.212720.116)
Supplement: Supplemental Material [file supp_gr.212720.116_Supplemental_Methods_Source_Code.zip › Supplemental_Methods_Source_Code/Supplemental_Table_Source_Code/Supplemental_Table_S12_Source_Code.pdf]

Investigating the expression specificity of genes located nearby Genome-wide association study (GWAS) single nucleotide polymorphisms (SNPs) in islet cell types

Introduction

For each gene examined, one-way analysis of variance (ANOVA) was used to determine if there were any statistically significant differences in average log2(CPM) expression of the gene in the four islet cell types (beta, alpha, delta, and gamma). Following this we performed a post hoc analysis using a Tukey's Honest Significant Difference (THSD) test to determine which islet cell types had significant differences in average expression of each gene. If the significant fold difference in average expression of a gene in an islet cell type compared to another cell type was greater than 4 (log2 > 2) and had an FDR-adjusted p-value less than 5%, that gene was considered to be significantly enriched in the respective cell group. This method was repeated for each gene to identify those that were expressed in a particular cell type or collection of islet cell types.

Genes that did not show any statistically significant differences in average expression based on our ANOVA test were classified as "pan-islet" if they had an average log2(CPM) expression greater than 4 in all islet cell types. Genes that were not determined to be cell type-specific or pan-islet were classified into two groups. If the average expression of the gene was log2CPM < 2 across all islet cell types, the gene was classified as "lowly expressed". The remaining genes that did not show enrichment in an islet cell type were classified as "< 4-fold change".

We examined the expression of genes nearby diabetes related GWAS single nucleotide polymorphisms (SNPs) in our islet cell types. Lists of the various diabetes related SNPs are available in Folder/Table_S14. We performed a separate analysis for each diabetes related disease/trait. First, we identified all SNPs associated with the respective trait/disease. Next, we obtained all genes from our list of 26,616 protein coding genes and lincRNAs that overlapped within 1 megabase upstream and downstream of the chromosomal locus of each SNP. Afterwards, we performed a similar ANOVA test and THSD post hoc analysis to characterize the expression of these genes in our islet cell types.

Analysis of Variance (ANOVA) and Tukey Honest Significant Difference (HSD) Testing

```r
suppressPackageStartupMessages(library(Biobase))
suppressPackageStartupMessages(library(edgeR))
suppressPackageStartupMessages(library(readxl))
suppressPackageStartupMessages(library(RColorBrewer))
suppressPackageStartupMessages(library(GenomicRanges))
rm(list = ls())
library(Biobase)
library(edgeR)
library(readxl)
library(RColorBrewer)
library(GenomicRanges)

setwd("/Users/lawlon/Documents/Final_RNA_Seq_2/ANOVA_2/GWAS_Catalog/")
# Read in the catalog
catalog <- read.delim2(file = "gwas_catalog_v1.0-associations_e84_r2016-05-01.tsv",
                       header = T, row.names = NULL,
```

```r
                        check.names = F, sep = "\t")

setwd("/Users/lawlon/Documents/Final_RNA_Seq_3/ANOVA_3/GWAS_Catlog_3/")
# Name of study to examine
study <- c("Type 2 diabetes", "Type 1 diabetes", "Proinsulin",
           "Fasting insulin", "glucose")

# Loop through study list
for (q in 1:length(study)) {
  # Grab any results containing pattern "Type 2 diabetes"
T2D.ids <- which(grepl(x = catalog$`DISEASE/TRAIT`,
                       pattern = study[q]) == TRUE)
T2D.snps <- catalog[T2D.ids,]

# Obtain only the unique SNP regions
region.dups <- duplicated(T2D.snps$REGION)
T2D.res <- T2D.snps[!region.dups,]

# Write snps used to file
write.csv(T2D.res, file = paste(study[q],
                                "SNPs.used.in.ANOVA.csv", sep = "."))

#SNP number used
snp.num <- dim(T2D.res)[1]

# Load in gene annotations
setwd("/Users/lawlon/Documents/Final_RNA_Seq_3/Data/")

#### MAKE SURE DATA TYPE IS CORRECT ####
pheno <- "NonT2D"

load("nonT2D.rdata")
s.anns <- pData(cnts.eset)
old.anns <- as(featureData(cnts.eset),"data.frame")
cnts <- exprs(cnts.eset)

# Calculate the cpm of the data
cpms <- cpm(x = cnts)
data <- log2(cpms+1)

# Obtain expression info for endocrine cells only
s.sel <- s.anns[s.anns$cell.type %in% c("INS", "GCG", "PPY", "SST"),]
exp.log2 <- data[, rownames(s.sel)]

# Read in gene annotations for GRCH38 (26,616 protein coding and lincRNAs)
p.anns <- read.csv("GRCH38.protein.coding.lincRNA.gene.annotations.csv",
                   header = TRUE, check.names = FALSE, row.names = 1)
# Set ensembl ids to rownames
rownames(p.anns) <- p.anns[,1]
p.anns[,1] <- NULL

# For each chromosome position in selected gwas list,
  #make a list of all genes that are 1000000 bases before or after location
```

```r
# Grab CHR_POS, CHR_ID, make a granges object
chr_pos <- T2D.res$CHR_POS
chr_id <- T2D.res$CHR_ID
snp_nams <- as.character(T2D.res$SNPS)
# Make empty matrix of 4 columnsm will hold chr names, start, and end, names
empty <- matrix(data = NA, nrow = length(chr_pos), ncol = 4)
for (i in 1:dim(empty)[1]) {
  # Add chromosome name to column 1
  empty[i,1] <- as.character(chr_id[i])
  # start position -1000000 bases
  empty[i,2] <- chr_pos[i] - 1000000
  # end position +1000000 bases
  empty[i,3] <- chr_pos[i] + 1000000
  # add snp names
  empty[i,4] <- snp_nams[i]
}

# Remove any NA entries
na.ids <- is.na(empty)
nas <- which(na.ids[,2] == TRUE)
if (length(nas) > 0) {
  res <- empty[-nas,]
} else {
  res <- empty
}


# Add column names
colnames(res) <- c("chr", "start", "end", "names")

# Make a granges object from SNP indices
res.df <- as.data.frame(res)
# Change columns 2 and 3 (start and end) to numeric
res.df[,2] <- as.numeric(as.character(res.df[,2]))
res.df[,3] <- as.numeric(as.character(res.df[,3]))
# Make the granges object
gr.T2D <- makeGRangesFromDataFrame(df = res.df, keep.extra.columns = TRUE)

# Get gene anns information from p.anns
gene_chr <- as.character(p.anns$`Chromosome Name`)
gene_start <- p.anns$`Gene Start (bp)`
gene_end <- p.anns$`Gene End (bp)`
gene_id <- rownames(p.anns)

genes_res <- cbind(gene_chr, gene_start, gene_end, gene_id)
# add column names
colnames(genes_res) <- c("chr", "start", "end", "names")
genes_df <- as.data.frame(genes_res)
# Change columns 2 and 3 (start and end) to numeric
genes_df[,2] <- as.numeric(as.character(genes_df[,2]))
genes_df[,3] <- as.numeric(as.character(genes_df[,3]))

# Make granges object from gene anns
```

```r
gr.genes <- makeGRangesFromDataFrame(df = genes_df, keep.extra.columns = TRUE)

# Find which genes from annotation information overlap with each of snp coordinates
overlaps <- subsetByOverlaps(query = gr.genes, subject = gr.T2D)

# Get names of genes that overlap
ov.genes <- overlaps$names


###### START OF ANOVA CODE

setwd("/Users/lawlon/Documents/Final_RNA_Seq_3/ANOVA_3/GWAS_Catlog_3/")
# Names for violin plot and ANOVA spreadsheet
fname <- paste(pheno,study[q], "logFC.filtered.adj.pval.expressed.genes.csv", sep=".")
vname <- paste(pheno,study[q], "log2CPM.violin.plots.pdf", sep = ".")

# Match up overlap genes with ids
ov.ids <- NULL
for (i in 1:length(ov.genes)) {
  ind <- which(rownames(p.anns) == ov.genes[i])
  ov.ids <- c(ov.ids, ind)
}

# Get overlap gene annotations
p.anns.ov <- p.anns[ov.ids,]

# Remove duplicate gene names
dups <- duplicated(p.anns.ov$`Associated Gene Name`)
p.anns.sel <- p.anns.ov[!dups,]
# effective size of genes
eff.size <- dim(p.anns.sel)[1]

# Output list of genes that will be used in ANOVA/THSD
write.csv(x = p.anns.sel, file = paste(study[q], "genes.used.in.ANOVA.csv", sep = "."))

# Obtain selected gene expression info for overlap genes
exp.log2.sel <- exp.log2[rownames(p.anns.sel),]
# Make ANOVA empty matrix
aov.res <- matrix(0,nrow= nrow(exp.log2.sel),ncol=13)

# ANOVA for loop
for(k in 1:nrow(exp.log2.sel)) {
  dat <- data.frame(exp = exp.log2.sel[k,],cell.type = s.sel$cell.type)
  results = aov(exp ~ cell.type, data=dat)
  p.val <- summary(results)[[1]][[5]][1]
  mc.res <- TukeyHSD(results, conf.level = 0.95)$cell.type
  aov.res[k,1] <- p.val
  aov.res[k,2:7] <- mc.res[,1]
  aov.res[k,8:13] <- mc.res[,4]
}

colnames(aov.res) <- c("p.value",paste("logFC",rownames(mc.res),sep="."),
                       paste("adjPvalue",rownames(mc.res),sep="."))
```

```r
aov.res.ext <- data.frame(symbol = p.anns.sel$`Associated Gene Name`,aov.res)

#to count max logFC
r.max.fc <- apply(aov.res.ext[,3:8],1,max)
r.min.fc <- apply(aov.res.ext[,3:8],1,min)
#to select all genes with maximal fold change greatre than 2
# Matrix of genes with logFC that were at least greater than 2 in one comparison
aov.max <- aov.res.ext[r.max.fc > 2,]
aov.min <- aov.res.ext[r.min.fc < -2,]
aov.res.ext.sel <- rbind(aov.max, aov.min)

# Count all logFC greater than/equal to 2 or less than 2
fc.mat <- aov.res.ext[,3:8]
rownames(fc.mat) <- aov.res.ext$symbol
fc.bin <- fc.mat
fc.bin[fc.mat > -2 & fc.mat < 2] <- 0
fc.bin[fc.mat <= -2] <- 1
fc.bin[fc.mat >=2] <- 1
num.cells <- apply(fc.bin,1,sum)

#column order is INS, GCG, PPY, SST
# Count in how many different cells a gene is positively expressed
cell <- matrix(0,nrow = nrow(fc.mat),ncol=4)
# Logic for assigning expression for "first" cell groups
cell[fc.mat[,1] > 2,1] <- 1
cell[fc.mat[,2] > 2,3] <- 1
cell[fc.mat[,3] > 2,4] <- 1
cell[fc.mat[,4] > 2,3] <- 1
cell[fc.mat[,5] > 2,4] <- 1
cell[fc.mat[,6] > 2,4] <- 1
# Logic for assigning expression for "second" cell groups
cell[fc.mat[,1] < -2,2] <- 1
cell[fc.mat[,2] < -2,2] <- 1
cell[fc.mat[,3] < -2,2] <- 1
cell[fc.mat[,4] < -2,1] <- 1
cell[fc.mat[,5] < -2,1] <- 1
cell[fc.mat[,6] < -2,3] <- 1
colnames(cell) <- c("Beta", "Alpha", "Gamma", "Delta")
rownames(cell) <- aov.res.ext$symbol

# Remove duplicated gene symbols
dups <- duplicated(aov.res.ext.sel$symbol)
aov.res.ext.sel <- aov.res.ext.sel[!dups,]

n=1
idxs <- NULL
for (n in 1:length(aov.res.ext.sel$symbol)) {
  vals <- which(aov.res.ext.sel$symbol[n] == rownames(cell))
  idxs <- c(idxs, vals)
}

cell.sel <- cell[idxs,]
i=1
```

```r
nam <- NULL
for (i in 1:dim(cell.sel)[1]){
  colmz <- which(cell.sel[i,] == 1)
  if (length(colmz == 4)) {
    nam <- cbind(nam, paste(names(colmz)[1], names(colmz)[2],
                          names(colmz)[3], names(colmz)[4],sep="."))
  } else if (length(colmz == 3)) {
    nam <- cbind(nam, paste(names(colmz)[1], names(colmz)[2], names(colmz)[3],sep="."))
  } else if (length(colmz == 2)) {
    nam <- cbind(nam, paste(names(colmz)[1], names(colmz)[2], sep="."))
  }
}

# Remove "NA" from values
nam <- gsub(pattern = ".NA.NA.NA", replacement = "", nam)
nam <- gsub(pattern = ".NA.NA", replacement = "", nam)
nam <- gsub(pattern = ".NA", replacement = "", nam)
# combine names with multiples
nam1 <- t(nam)

cell.comb <- cbind(cell.sel, nam1)
colnames(cell.comb)[5] <- "Cell_Expressing_Gene"
cell.comb <- as.data.frame(cell.comb)

# Adjust the pvalue
adj.pval <- p.adjust(p=aov.res.ext.sel$p.value, "fdr")
adj.p <- which(adj.pval < 0.05)
aov.final <- aov.res.ext.sel[adj.p,]
rownames(aov.final) <- rownames(cell.comb)[adj.p]

cell.final <- cbind(cell.comb[adj.p,], aov.final[,2:14], adj.pval[adj.p])
colnames(cell.final)[19] <- "FDR.adj.p.value"
cell.final <- cell.final[, c(1:6,19,7:18)]

# Sort by which are in only one cell type
multis <- grepl(x = cell.final[,5], pattern = "\\w\\.", perl = TRUE)
# Extract only genes in single cell type
singles <- cell.final[!multis,]
multiples <- cell.final[multis,]

# Sort singles by cell type
beta.ids <- which(singles[,5] == "Beta")
alp.ids <- which(singles[,5] == "Alpha")
del.ids <- which(singles[,5] == "Delta")
gam.ids <- which(singles[,5] == "Gamma")

# Combine all ids in order
all.ids <- c(beta.ids, alp.ids, gam.ids, del.ids)
sing.ord <- singles[all.ids,]

# Sort the multiples by genes in 2 groups, then genes in 3
threes.id <- grepl(x = multiples[,5], pattern = "\\w\\.\\w*\\.", perl = TRUE)
twos <- multiples[!threes.id,]
```

```r
# Find out which two cells express the genes
b.a.id <- which(twos[,5] == "Beta.Alpha")
b.d.id <- which(twos[,5] == "Beta.Delta")
b.g.id <- which(twos[,5] == "Beta.Gamma")

a.d.id <- which(twos[,5] == "Alpha.Delta")
a.g.id <- which(twos[,5] == "Alpha.Gamma")

g.d.id <- which(twos[,5] == "Gamma.Delta")

all.twos <- c(b.a.id, b.g.id, b.d.id, a.g.id, a.d.id, g.d.id)
twos <- twos[all.twos,]

# Find out which three cells express the genes
# Ordering was Beta, Alpha, Gamma,Delta
threes <- multiples[threes.id,]
b.a.d.id <- which(threes[,5] == "Beta.Alpha.Delta")
b.a.g.id <- which(threes[,5] == "Beta.Alpha.Gamma")
b.g.d.id <- which(threes[,5] == "Beta.Gamma.Delta")

a.g.d.id <- which(threes[,5] == "Alpha.Gamma.Delta")


all.threes <- c(b.a.d.id, b.a.g.id, b.g.d.id, a.g.d.id)
threes <- threes[all.threes,]

res <- rbind(sing.ord, twos,threes)

## Output final spreadsheet
write.csv(x=res, file = fname)

# Obtain stats of the ANOVA results (how many genes not expressed
  #in islets, how many pan-islet, how many specific)
ins <- which(s.sel$cell.type == "INS")
gcg <- which(s.sel$cell.type == "GCG")
sst <- which(s.sel$cell.type == "SST")
ppy <- which(s.sel$cell.type == "PPY")
# Obtain expression for each cell group
ins.exp <- exp.log2.sel[, ins]
gcg.exp <- exp.log2.sel[,gcg]
sst.exp <- exp.log2.sel[,sst]
ppy.exp <- exp.log2.sel[,ppy]
# Get average expression for each gene in each group
ins.rm <- rowMeans(ins.exp)
gcg.rm <- rowMeans(gcg.exp)
sst.rm <- rowMeans(sst.exp)
ppy.rm <- rowMeans(ppy.exp)

# Define pan-islet genes as those with average
  #log2cpm expression > 4 in each cell type
pans <- which(ins.rm > 4 & gcg.rm > 4 & sst.rm > 4 & ppy.rm > 4)
pan.genes <- p.anns.sel$`Associated Gene Name`[pans]
# Make sure there are no genes being called pan from ANOVA list
```

```r
pan.sel <- setdiff(pan.genes, rownames(res))

# Call the other genes as undetermined
# Combine ANOVA and pan genes
comb <- union(pan.genes, rownames(res))
comb.ids <- which(p.anns.sel$`Associated Gene Name` %in% comb)
undet <- p.anns.sel$`Associated Gene Name`[-comb.ids]

# Find the genes that are below expression threshold
undet.ids <- p.anns.sel[-comb.ids,]
sub.exp <- which(ins.rm[rownames(undet.ids)] < 2 & gcg.rm[rownames(undet.ids)] < 2 & sst.rm[rownames(und[illegible]
sub.exp.genes <- undet.ids$`Associated Gene Name`[sub.exp]

# genes that are sub-fold-change threshold are the remaining genes
sub.fold <- undet.ids[-sub.exp,]
sub.fold.genes <- sub.fold$`Associated Gene Name`

# Report all stats: the number of genes in one, two, three cell types, pan-islet, not-exprssed
tab.stats <- matrix(data=0, nrow = 17, ncol = 3)
tab.stats[,1] <- c("Beta", "Alpha", "Delta", "Gamma",
                   "Beta.Alpha", "Beta.Delta", "Beta.Gamma", "Alpha.Delta",
                   "Alpha.Gamma", "Gamma.Delta", "Beta.Alpha.Delta",
                   "Beta.Alpha.Gamma", "Beta.Gamma.Delta", "Alpha.Gamma.Delta",
                   "Pan-Islet", "Sub-Expression-Threshold", "Sub-Fold-Change-Threshold")
tab.stats[,2] <- c(length(beta.ids), length(alp.ids), length(del.ids),
                   length(gam.ids), length(b.a.id), length(b.d.id), length(b.g.id), length(a.d.id),
                   length(a.g.id), length(g.d.id), length(b.a.d.id), length(b.a.g.id),
                   length(b.g.d.id), length(a.g.d.id), length(pan.sel), length(sub.exp.genes),
                   length(sub.fold.genes))

# Column three will contain gene symbols
# Loop through results table, get genes
for (i in 1:dim(tab.stats)[1]) {
  # which genes in beta
  betas <- which(res[,5] == tab.stats[i,1])
  beta.genes <- rownames(res)[betas]
  # Concatenate genes together
  nams <- ""
  for (n in 1:length(beta.genes)) {
    nams <- paste(beta.genes[n], nams, sep=",")
  }
  # Remove the last comma from the names
  beta.nams <- gsub(x = nams, pattern = "\\,+$", replacement = "")
  tab.stats[i,3] <- beta.nams
}

# Add pan-islet and undetermined genes to table
nams <- ""
for (n in 1:length(pan.sel)) {
  nams <- paste(pan.sel[n], nams, sep=",")
}
# Remove the last comma from the names
pan.nams <- gsub(x = nams, pattern = "\\,+$", replacement = "")
```

```
tab.stats[15,3] <- pan.nams

# Sub-expression threshold genes
nams <- ""
for (n in 1:length(sub.exp.genes)) {
  nams <- paste(sub.exp.genes[n], nams, sep=",")
}
# Remove the last comma from the names
sub.exp.nams <- gsub(x = nams, pattern = "\\,+$", replacement = "")
tab.stats[16,3] <- sub.exp.nams

# Sub-fold change threshold genes
nams <- ""
for (n in 1:length(sub.fold.genes)) {
  nams <- paste(sub.fold.genes[n], nams, sep=",")
}
# Remove the last comma from the names
sub.fold.nams <- gsub(x = nams, pattern = "\\,+$", replacement = "")
tab.stats[17,3] <- sub.fold.nams

# remove any rows which are zero
zeros <- which(tab.stats[,2] == 0)

if (length(zeros) > 0) {
  tab.sel <- tab.stats[-zeros,]
} else {
  tab.sel <- tab.stats
}

colnames(tab.stats) <- c("Expression in Endocrine Cells",
            paste("Number of Genes (out of ", eff.size, " genes, from ", snp.num,
            " SNP indices)", sep = ""), "Gene Symbol")
colnames(tab.sel) <- colnames(tab.stats)

# Write ANOVA stats to file
write.csv(tab.sel, file = paste(pheno,study,"ANOVA.stats.csv", sep = "."))
# Write pan-islet genes to files
write.csv(pan.sel, file = paste(pheno,study, "Pan.islet.genes.csv", sep = "."))
write.csv(sub.exp.genes, file = paste(pheno, study, "sub.expression.threshold.genes.csv", sep = "."))
write.csv(sub.fold.genes, file = paste(pheno, study, "sub.fold.change.threshold.genes.csv", sep = "."))

}
```

Session Information

```
suppressPackageStartupMessages(library(Biobase))
suppressPackageStartupMessages(library(edgeR))
suppressPackageStartupMessages(library(readxl))
suppressPackageStartupMessages(library(RColorBrewer))
suppressPackageStartupMessages(library(GenomicRanges))
rm(list = ls())
library(Biobase)
```

```r
library(edgeR)
library(readxl)
library(RColorBrewer)
library(GenomicRanges)
sessionInfo()
```

```
## R version 3.3.0 (2016-05-03)
## Platform: x86_64-apple-darwin13.4.0 (64-bit)
## Running under: OS X 10.11.3 (El Capitan)
##
## locale:
## [1] en_US.UTF-8/en_US.UTF-8/en_US.UTF-8/C/en_US.UTF-8/en_US.UTF-8
##
## attached base packages:
## [1] stats4    parallel  stats     graphics  grDevices utils     datasets
## [8] methods   base
##
## other attached packages:
##  [1] GenomicRanges_1.24.2 GenomeInfoDb_1.8.1   IRanges_2.6.1
##  [4] S4Vectors_0.10.1     RColorBrewer_1.1-2   readxl_0.1.1
##  [7] edgeR_3.14.0         limma_3.28.11        Biobase_2.32.0
## [10] BiocGenerics_0.18.0
##
## loaded via a namespace (and not attached):
##  [1] Rcpp_0.12.5     digest_0.6.9    formatR_1.4     magrittr_1.5
##  [5] evaluate_0.9    zlibbioc_1.18.0 stringi_1.1.1   XVector_0.12.0
##  [9] rmarkdown_0.9.6 tools_3.3.0     stringr_1.0.0   yaml_2.1.13
## [13] htmltools_0.3.5 knitr_1.13
```
