# Supplementary material for: Single-cell transcriptomes identify human islet cell signatures and reveal cell-type–specific expression changes in type 2 diabetes
Source: Genome Res. 2017 Feb;27(2):208–22. doi: 10.1101/gr.212720.116 (PMC5287227; doi:10.1101/gr.212720.116)
Supplement: Supplemental Material [file supp_gr.212720.116_Supplemental_Methods_Source_Code.zip › Supplemental_Methods_Source_Code/Supplemental_Table_Source_Code/Supplemental_Table_S13_Source_Code.pdf]

Comparison of T2D and Non-diabetic Cell Type Specific Differentially Expressed Genes between Lawlor et al. 2016, Segerstolpe et al. 2016, and Wang et al. 2016

Introduction

Using a two-sided Wilcoxon rank sum test, we sought to validate the observed cell type-specific differences in T2D islets from recent, independent islet single cell RNA-seq studies (Wang et al. 2016; Segerstolpe et al., 2016).

Comparing trends in expression of defined beta, alpha, and delta cell differentially expressed genes from Lawlor et al. 2016 with other datasets

```
suppressPackageStartupMessages(library(Biobase))
suppressPackageStartupMessages(library(gtools))
suppressPackageStartupMessages(library(xlsx))
library(Biobase)
library(gtools)
library(xlsx)
# Load in segerstolpe et al. 2016 (Sandberg group) dataset
setwd("/Users/lawlon/Documents/Sandberg_Islet/")
load("Sandberg.processed.FPKM.rdata")
sand.dat <- exprs(eset)
# make expression matrix numeric
sand.exp <- t(apply(sand.dat,1, as.numeric))
# add column names
colnames(sand.exp) <- colnames(sand.dat)
# sample annotations
sand.anns <- read.csv("/Users/lawlon/Documents/Sandberg_Islet/Sandberg.islet.sample.annotations.csv", he
                      check.names = F, row.names = NULL, stringsAsFactors = F)
# set column names
col.names <- as.character(sand.anns[1,])
colnames(sand.anns) <- col.names
sand.anns <- sand.anns[-1,]
# set rownames as sample names
rownames(sand.anns) <- sand.anns[,1]
sand.anns[,1] <- NULL

# Specify markers and cell names
cell_type <- "beta cell"

# nont2d and t2d data
nont2d.anns <- sand.anns[sand.anns$`Characteristics[disease]` == "normal" & sand.anns$`Characteristics[c
t2d.anns <- sand.anns[sand.anns$`Characteristics[disease]` == "type II diabetes mellitus" & sand.anns$`C
# separate nont2d and t2d datasets
sand.nont2d <- sand.exp[, rownames(nont2d.anns)]
sand.t2d <- sand.exp[, rownames(t2d.anns)]
name <- paste(cell_type, "DEGs_in_Segerstolpe" , sep = "_")
```

```
# Load genes to compare
genelist <- read.csv("/Users/lawlon/Documents/Final_RNA_Seq_3/Differential_Expression_3/Single_Cell/T2D_
                     header = T, check.names = F, row.names = 1, stringsAsFactors = F)

# find genes that intersect between lists
gene.int <- intersect(genelist$Associated.Gene.Name, rownames(sand.exp))
# extract genes of interest for both groups
sand.nont2d.sel <- sand.nont2d[gene.int,]
sand.t2d.sel <- sand.t2d[gene.int,]
pvals <- numeric(length = nrow(sand.nont2d.sel))
ttest.pvals <- numeric(length = nrow(sand.nont2d.sel))
# average fold change of T2D vs ND
fc <- numeric(length = nrow(sand.nont2d.sel))
# perform rank test calculations
for(i in 1:length(pvals))
{
  pvals[i] <- wilcox.test(sand.nont2d.sel[i,],sand.t2d.sel[i,])$p.value
  fc[i] <- foldchange(num = mean(sand.t2d.sel[i,]), denom = mean(sand.nont2d.sel[i,]))
}
# obtain gene symbol names
symbol <- rownames(sand.nont2d.sel)
# create resulting data frame
res <- data.frame(symbol = symbol, fold_change = fc, rank.test.pvals = pvals)

# convert logFC to linear fold change of genes in our dataset
ids <- NULL
for (i in 1:length(res$symbol)) {
  idx <- which(genelist$Associated.Gene.Name == res$symbol[i])
  ids <- c(ids, idx)
}

# get selected logFC
logfc.sel <- genelist$logFC[ids]
# calculate normal fold change
linfc <- logfc.sel
for (x in 1:length(logfc.sel)) {
  if (logfc.sel[x] < 0 ) {
    fc <- 2**(abs(logfc.sel[x]))
    linfc[x] <- fc*-1
  } else {
    linfc[x] <- 2**logfc.sel[x]
  }
}

# combine results to data frame
res.ext <- cbind(res, linfc)
# re-order columns
res.ext <- res.ext[, c(1,4,2,3)]
colnames(res.ext) <- c("Gene_symbol", "fold_change_in_Lawlor_et_al_2016", "fold_change_in_Segerstolpe_e
                       "rank_test_pvals_in_Segerstolpe_et_al_2016")
# sort results by p value
ord <- order(res.ext$`rank_test_pvals_in_Segerstolpe _et_al_2016`, decreasing = FALSE)
res.fin <- res.ext[ord,]
```

```
# write results to file
write.xlsx(x = res.fin, file = "Supplemental_Table_S12.xlsx", sheetName = name)

# Load in Wang et al. 2016 dataset (Kaestner group)
wang.exp <- read.csv("/Users/lawlon/Documents/Kaestner_GSE83139/GSE83139.ordered.expression.matrix.cpm.c
                     header = T, check.names = F, row.names = 1, stringsAsFactors = F)
wang.exp <- as.matrix(wang.exp)
# sample annotations
wang.anns <- read.csv("/Users/lawlon/Documents/Kaestner_GSE83139/GSE83139.ordered.sample.annotations.csv
                      header = T, check.names = F, row.names = 3)
# specify cell type
cell_type = "beta"

# Use samples with phenotype info
w.cont <- wang.anns[wang.anns$Health == "control" & wang.anns$Cell_Type %in% cell_type & wang.anns$Age =
w.t2d <- wang.anns[wang.anns$Health == "T2D" & wang.anns$Cell_Type %in% cell_type & wang.anns$Age == "ad
w.cont <- as.matrix(w.cont)
w.cont <- as.data.frame(w.cont)
w.t2d <- as.matrix(w.t2d)
w.t2d <- as.data.frame(w.t2d)
# combine anns
w.sel <- rbind(w.cont, w.t2d)
# selected counts
wang.exp.ND <- wang.exp[, rownames(w.cont)]
wang.exp.T2D <- wang.exp[, rownames(w.t2d)]
name <- paste(cell_type, "DEGs_in_Wang", sep = "_")

# Load genes to compare
genelist <- read.csv("/Users/lawlon/Documents/Final_RNA_Seq_3/Differential_Expression_3/Single_Cell/T2D_
                     header = T, check.names = F, row.names = 1, stringsAsFactors = F)

# find genes that intersect between lists
gene.int <- intersect(genelist$Associated.Gene.Name, rownames(wang.exp.ND))
# extract genes of interest for both groups
wang.nont2d.sel <- wang.exp.ND[gene.int,]
wang.t2d.sel <- wang.exp.T2D[gene.int,]

pvals <- numeric(length = nrow(wang.nont2d.sel))
# average fold change of T2D vs ND
fc <- numeric(length = nrow(wang.nont2d.sel))
for(i in 1:length(pvals))
{
  pvals[i] <- wilcox.test(wang.nont2d.sel[i,],wang.t2d.sel[i,])$p.value
  fc[i] <- foldchange(num = mean(wang.t2d.sel[i,]), denom = mean(wang.nont2d.sel[i,]))
}
symbol <- rownames(wang.nont2d.sel)
# create resulting data frame
wang.res <- data.frame(symbol = symbol, fold_change = fc, rank.test.pvals = pvals)
# convert logFC to linear fold change of genes in our dataset
ids <- NULL
for (i in 1:length(wang.res$symbol)) {
  idx <- which(genelist$Associated.Gene.Name == wang.res$symbol[i])
  ids <- c(ids, idx)
```

```
}

# get selected logFC
logfc.sel <- genelist$logFC[ids]
# calculate normal fold change
linfc <- logfc.sel
for (x in 1:length(logfc.sel)) {
  if (logfc.sel[x] < 0 ) {
    fc <- 2**(abs(logfc.sel[x]))
    linfc[x] <- fc*-1
  } else {
    linfc[x] <- 2**logfc.sel[x]
  }
}

# combine results to data frame
wang.res.ext <- cbind(wang.res, linfc)
# re-order columns
wang.res.ext <- wang.res.ext[, c(1,4,2,3)]
colnames(wang.res.ext) <- c("Gene_symbol", "fold_change_in_Lawlor_et_al_2016", "fold_change_in_Wang_et_a
                       "rank_test_pvals_in_Wang_et_al_2016")
# sort results by p value
wang.ord <- order(wang.res.ext$rank_test_pvals_in_Wang_et_al_2016, decreasing = FALSE)
wang.res.fin <- wang.res.ext[wang.ord,]
# write results to file
write.xlsx(x = wang.res.fin, file = "Supplemental_Table_S12.xlsx", sheetName = name,
           append = TRUE)

# Find genes with same trend in fold change and have rank test p-value less than 0.05
# For segerstolpe dataset (Sandberg group)
sand.res.pos <- res.fin[res.fin[,3] > 0,]
sand.res.pos.sig <- sand.res.pos[sand.res.pos[,4] < 0.05,]
sand.res.neg <- res.fin[res.fin[,3] < 0,]
sand.res.neg.sig <- sand.res.neg[sand.res.neg[,4] < 0.05,]
# genelist pos and neg enriched
genelist.pos <- genelist[genelist$logFC > 0,]
genelist.neg <- genelist[genelist$logFC < 0,]
# find intersection of genes
sand.int.pos <- intersect(sand.res.pos.sig[,1], genelist.pos$Associated.Gene.Name)
sand.int.neg <- intersect(sand.res.neg.sig[,1], genelist.neg$Associated.Gene.Name)
sand.int.genes <- c(sand.int.neg, sand.int.pos)
# extract overlapping genes
sand.gen.ids <- which(res.fin[,1] %in% sand.int.genes)
res.fin.ov.beta <- res.fin[sand.gen.ids,]
# rank by p-value
sand.ord <- order(res.fin.ov.beta[,4], decreasing = FALSE)
sand.res.fin.ov.beta <- res.fin.ov.beta[sand.ord,]
# write results to file
write.xlsx(x = sand.res.fin.ov.beta, file = "Supplemental_Table_S12.xlsx",
           sheetName = "beta_DEGs_Lawlor_Segerstolpe_Ov", append = TRUE)

# For wang dataset (Kaestner group)
wang.res.pos <- wang.res.fin[wang.res.fin[,3] > 0,]
```

```
wang.res.pos.sig <- wang.res.pos[wang.res.pos[,4] < 0.05,]
wang.res.neg <- wang.res.fin[wang.res.fin[,3] < 0,]
wang.res.neg.sig <- wang.res.neg[wang.res.neg[,4] < 0.05,]
# genelist pos and neg enriched
genelist.pos <- genelist[genelist$logFC > 0,]
genelist.neg <- genelist[genelist$logFC < 0,]
# find intersection of genes
wang.int.pos <- intersect(wang.res.pos.sig[,1], genelist.pos$Associated.Gene.Name)
wang.int.neg <- intersect(wang.res.neg.sig[,1], genelist.neg$Associated.Gene.Name)
wang.int.genes <- c(wang.int.neg, wang.int.pos)
# extract overlapping genes
wang.gen.ids <- which(wang.res.fin[,1] %in% wang.int.genes)
wang.res.fin.ov.beta <- wang.res.fin[wang.gen.ids,]
# rank by p-value
wang.ord <- order(wang.res.fin.ov.beta[,4], decreasing = FALSE)
wang.res.fin.ov.beta <- wang.res.fin.ov.beta[wang.ord,]
# write results to file
write.xlsx(x = wang.res.fin.ov.beta, file = "Supplemental_Table_S12.xlsx",
           sheetName = "beta_DEGs_Lawlor_Wang_Ov", append = TRUE)

# Find genes that overlap all datasets
all.study.genes <- intersect(wang.res.fin.ov.beta[,1], sand.res.fin.ov.beta[,1])
# extract ids from wang data frame
all.wang.ids <- which(wang.res.fin.ov.beta[,1] %in% all.study.genes)
wang.beta.df <- wang.res.fin.ov.beta[all.wang.ids,]
# find same genes from segerstolpe data frame
all.sand.ids <- NULL
for (s in 1:length(wang.beta.df[,1])) {
  s.idx <- which(as.character(sand.res.fin.ov.beta[,1]) == as.character(wang.beta.df[s,1]))
  all.sand.ids <- c(all.sand.ids, s.idx)
}

sand.beta.df <- sand.res.fin.ov.beta[all.sand.ids,]
# combine data frames
rownames(sand.beta.df) <- sand.beta.df[,1]
rownames(wang.beta.df) <- wang.beta.df[,1]
beta.all.ov <- cbind(wang.beta.df, sand.beta.df[,c(3:4)])
# write results to file
write.xlsx(x = beta.all.ov, file = "Supplemental_Table_S12.xlsx",
           sheetName = "beta_DEGs_All_studies", append = TRUE)

### Analysis for Alpha cell DEGs
# Specify markers and cell names
cell_type <- "alpha cell"

# nont2d and t2d data
nont2d.anns <- sand.anns[sand.anns$`Characteristics[disease]` == "normal" & sand.anns$`Characteristics[
t2d.anns <- sand.anns[sand.anns$`Characteristics[disease]` == "type II diabetes mellitus" & sand.anns$`
# separate nont2d and t2d datasets
sand.nont2d <- sand.exp[, rownames(nont2d.anns)]
sand.t2d <- sand.exp[, rownames(t2d.anns)]
name <- paste(cell_type, "DEGs_in_Segerstolpe" , sep = "_")
```

```r
# Load genes to compare
genelist <- read.csv("/Users/lawlon/Documents/Final_RNA_Seq_3/Differential_Expression_3/Single_Cell/T2D
                     header = T, check.names = F, row.names = 1, stringsAsFactors = F)

# find genes that intersect between lists
gene.int <- intersect(genelist$Associated.Gene.Name, rownames(sand.exp))
# extract genes of interest for both groups
sand.nont2d.sel <- sand.nont2d[gene.int,]
sand.t2d.sel <- sand.t2d[gene.int,]
pvals <- numeric(length = nrow(sand.nont2d.sel))
ttest.pvals <- numeric(length = nrow(sand.nont2d.sel))
# average fold change of T2D vs ND
fc <- numeric(length = nrow(sand.nont2d.sel))
# perform rank test calculations
for(i in 1:length(pvals))
{
  pvals[i] <- wilcox.test(sand.nont2d.sel[i,],sand.t2d.sel[i,])$p.value
  fc[i] <- foldchange(num = mean(sand.t2d.sel[i,]), denom = mean(sand.nont2d.sel[i,]))
}
# obtain gene symbol names
symbol <- rownames(sand.nont2d.sel)
# create resulting data frame
res <- data.frame(symbol = symbol, fold_change = fc, rank.test.pvals = pvals)

# convert logFC to linear fold change of genes in our dataset
ids <- NULL
for (i in 1:length(res$symbol)) {
  idx <- which(genelist$Associated.Gene.Name == res$symbol[i])
  ids <- c(ids, idx)
}

# get selected logFC
logfc.sel <- genelist$logFC[ids]
# calculate normal fold change
linfc <- logfc.sel
for (x in 1:length(logfc.sel)) {
  if (logfc.sel[x] < 0 ) {
    fc <- 2**(abs(logfc.sel[x]))
    linfc[x] <- fc*-1
  } else {
    linfc[x] <- 2**logfc.sel[x]
  }
}

# combine results to data frame
res.ext <- cbind(res, linfc)
# re-order columns
res.ext <- res.ext[, c(1,4,2,3)]
colnames(res.ext) <- c("Gene_symbol", "fold_change_in_Lawlor_et_al_2016", "fold_change_in_Segerstolpe_e
                       "rank_test_pvals_in_Segerstolpe_et_al_2016")
# sort results by p value
ord <- order(res.ext$`rank_test_pvals_in_Segerstolpe _et_al_2016`, decreasing = FALSE)
res.fin <- res.ext[ord,]
```

```r
# write results to file
write.xlsx(x = res.fin, file = "Supplemental_Table_S12.xlsx", sheetName = name)

# specify cell type
cell_type = "alpha"

# Use samples with phenotype info
w.cont <- wang.anns[wang.anns$Health == "control" & wang.anns$Cell_Type %in% cell_type & wang.anns$Age =
w.t2d <- wang.anns[wang.anns$Health == "T2D" & wang.anns$Cell_Type %in% cell_type & wang.anns$Age == "a
w.cont <- as.matrix(w.cont)
w.cont <- as.data.frame(w.cont)
w.t2d <- as.matrix(w.t2d)
w.t2d <- as.data.frame(w.t2d)
# combine anns
w.sel <- rbind(w.cont, w.t2d)
# selected counts
wang.exp.ND <- wang.exp[, rownames(w.cont)]
wang.exp.T2D <- wang.exp[, rownames(w.t2d)]
name <- paste(cell_type, "DEGs_in_Wang", sep = "_")

# Load genes to compare
genelist <- read.csv("/Users/lawlon/Documents/Final_RNA_Seq_3/Differential_Expression_3/Single_Cell/T2D
                     header = T, check.names = F, row.names = 1, stringsAsFactors = F)

# find genes that intersect between lists
gene.int <- intersect(genelist$Associated.Gene.Name, rownames(wang.exp.ND))
# extract genes of interest for both groups
wang.nont2d.sel <- wang.exp.ND[gene.int,]
wang.t2d.sel <- wang.exp.T2D[gene.int,]

pvals <- numeric(length = nrow(wang.nont2d.sel))
# average fold change of T2D vs ND
fc <- numeric(length = nrow(wang.nont2d.sel))
for(i in 1:length(pvals))
{
  pvals[i] <- wilcox.test(wang.nont2d.sel[i,],wang.t2d.sel[i,])$p.value
  fc[i] <- foldchange(num = mean(wang.t2d.sel[i,]), denom = mean(wang.nont2d.sel[i,]))
}
symbol <- rownames(wang.nont2d.sel)
# create resulting data frame
wang.res <- data.frame(symbol = symbol, fold_change = fc, rank.test.pvals = pvals)
# convert logFC to linear fold change of genes in our dataset
ids <- NULL
for (i in 1:length(wang.res$symbol)) {
  idx <- which(genelist$Associated.Gene.Name == wang.res$symbol[i])
  ids <- c(ids, idx)
}

# get selected logFC
logfc.sel <- genelist$logFC[ids]
# calculate normal fold change
linfc <- logfc.sel
for (x in 1:length(logfc.sel)) {
```

```
  if (logfc.sel[x] < 0 ) {
    fc <- 2**(abs(logfc.sel[x]))
    linfc[x] <- fc*-1
  } else {
    linfc[x] <- 2**logfc.sel[x]
  }
}

# combine results to data frame
wang.res.ext <- cbind(wang.res, linfc)
# re-order columns
wang.res.ext <- wang.res.ext[, c(1,4,2,3)]
colnames(wang.res.ext) <- c("Gene_symbol", "fold_change_in_Lawlor_et_al_2016", "fold_change_in_Wang_et_a
                       "rank_test_pvals_in_Wang_et_al_2016")
# sort results by p value
wang.ord <- order(wang.res.ext$rank_test_pvals_in_Wang_et_al_2016, decreasing = FALSE)
wang.res.fin <- wang.res.ext[wang.ord,]
# write results to file
write.xlsx(x = wang.res.fin, file = "Supplemental_Table_S12.xlsx", sheetName = name,
           append = TRUE)

# Find genes with same trend in fold change and have rank test p-value less than 0.05
# For segerstolpe dataset (Sandberg group)
sand.res.pos <- res.fin[res.fin[,3] > 0,]
sand.res.pos.sig <- sand.res.pos[sand.res.pos[,4] < 0.05,]
sand.res.neg <- res.fin[res.fin[,3] < 0,]
sand.res.neg.sig <- sand.res.neg[sand.res.neg[,4] < 0.05,]
# genelist pos and neg enriched
genelist.pos <- genelist[genelist$logFC > 0,]
genelist.neg <- genelist[genelist$logFC < 0,]
# find intersection of genes
sand.int.pos <- intersect(sand.res.pos.sig[,1], genelist.pos$Associated.Gene.Name)
sand.int.neg <- intersect(sand.res.neg.sig[,1], genelist.neg$Associated.Gene.Name)
sand.int.genes <- c(sand.int.neg, sand.int.pos)
# extract overlapping genes
sand.gen.ids <- which(res.fin[,1] %in% sand.int.genes)
res.fin.ov.beta <- res.fin[sand.gen.ids,]
# rank by p-value
sand.ord <- order(res.fin.ov.beta[,4], decreasing = FALSE)
sand.res.fin.ov.beta <- res.fin.ov.beta[sand.ord,]
# write results to file
write.xlsx(x = sand.res.fin.ov.beta, file = "Supplemental_Table_S12.xlsx",
           sheetName = "alpha_DEGs_Lawlor_Segerstolpe_Ov", append = TRUE)

# For wang dataset (Kaestner group)
wang.res.pos <- wang.res.fin[wang.res.fin[,3] > 0,]
wang.res.pos.sig <- wang.res.pos[wang.res.pos[,4] < 0.05,]
wang.res.neg <- wang.res.fin[wang.res.fin[,3] < 0,]
wang.res.neg.sig <- wang.res.neg[wang.res.neg[,4] < 0.05,]
# genelist pos and neg enriched
genelist.pos <- genelist[genelist$logFC > 0,]
genelist.neg <- genelist[genelist$logFC < 0,]
# find intersection of genes
```

```
wang.int.pos <- intersect(wang.res.pos.sig[,1], genelist.pos$Associated.Gene.Name)
wang.int.neg <- intersect(wang.res.neg.sig[,1], genelist.neg$Associated.Gene.Name)
wang.int.genes <- c(wang.int.neg, wang.int.pos)
# extract overlapping genes
wang.gen.ids <- which(wang.res.fin[,1] %in% wang.int.genes)
wang.res.fin.ov.beta <- wang.res.fin[wang.gen.ids,]
# rank by p-value
wang.ord <- order(wang.res.fin.ov.beta[,4], decreasing = FALSE)
wang.res.fin.ov.beta <- wang.res.fin.ov.beta[wang.ord,]
# write results to file
write.xlsx(x = wang.res.fin.ov.beta, file = "Supplemental_Table_S12.xlsx",
           sheetName = "alpha_DEGs_Lawlor_Wang_Ov", append = TRUE)

# Find genes that overlap all datasets
all.study.genes <- intersect(wang.res.fin.ov.beta[,1], sand.res.fin.ov.beta[,1])
# extract ids from wang data frame
all.wang.ids <- which(wang.res.fin.ov.beta[,1] %in% all.study.genes)
wang.beta.df <- wang.res.fin.ov.beta[all.wang.ids,]
# find same genes from segerstolpe data frame
all.sand.ids <- NULL
for (s in 1:length(wang.beta.df[,1])) {
  s.idx <- which(as.character(sand.res.fin.ov.beta[,1]) == as.character(wang.beta.df[s,1]))
  all.sand.ids <- c(all.sand.ids, s.idx)
}

sand.beta.df <- sand.res.fin.ov.beta[all.sand.ids,]
# combine data frames
rownames(sand.beta.df) <- sand.beta.df[,1]
rownames(wang.beta.df) <- wang.beta.df[,1]
beta.all.ov <- cbind(wang.beta.df, sand.beta.df[,c(3:4)])
# write results to file
write.xlsx(x = beta.all.ov, file = "Supplemental_Table_S12.xlsx",
           sheetName = "alpha_DEGs_All_studies", append = TRUE)

#### Analyses for delta cells
# Specify markers and cell names
cell_type <- "delta cell"

# nont2d and t2d data
nont2d.anns <- sand.anns[sand.anns$`Characteristics[disease]` == "normal" & sand.anns$`Characteristics[c
t2d.anns <- sand.anns[sand.anns$`Characteristics[disease]` == "type II diabetes mellitus" & sand.anns$`C
# separate nont2d and t2d datasets
sand.nont2d <- sand.exp[, rownames(nont2d.anns)]
sand.t2d <- sand.exp[, rownames(t2d.anns)]
name <- paste(cell_type, "DEGs_in_Segerstolpe" , sep = "_")

# Load genes to compare
genelist <- read.csv("/Users/lawlon/Documents/Final_RNA_Seq_3/Differential_Expression_3/Single_Cell/T2D_
                     header = T, check.names = F, row.names = 1, stringsAsFactors = F)

# find genes that intersect between lists
gene.int <- intersect(genelist$Associated.Gene.Name, rownames(sand.exp))
# extract genes of interest for both groups
```

```
sand.nont2d.sel <- sand.nont2d[gene.int,]
sand.t2d.sel <- sand.t2d[gene.int,]
pvals <- numeric(length = nrow(sand.nont2d.sel))
ttest.pvals <- numeric(length = nrow(sand.nont2d.sel))
# average fold change of T2D vs ND
fc <- numeric(length = nrow(sand.nont2d.sel))
# perform rank test calculations
for(i in 1:length(pvals))
{
  pvals[i] <- wilcox.test(sand.nont2d.sel[i,],sand.t2d.sel[i,])$p.value
  fc[i] <- foldchange(num = mean(sand.t2d.sel[i,]), denom = mean(sand.nont2d.sel[i,]))
}
# obtain gene symbol names
symbol <- rownames(sand.nont2d.sel)
# create resulting data frame
res <- data.frame(symbol = symbol, fold_change = fc, rank.test.pvals = pvals)

# convert logFC to linear fold change of genes in our dataset
ids <- NULL
for (i in 1:length(res$symbol)) {
  idx <- which(genelist$Associated.Gene.Name == res$symbol[i])
  ids <- c(ids, idx)
}

# get selected logFC
logfc.sel <- genelist$logFC[ids]
# calculate normal fold change
linfc <- logfc.sel
for (x in 1:length(logfc.sel)) {
  if (logfc.sel[x] < 0 ) {
    fc <- 2**(abs(logfc.sel[x]))
    linfc[x] <- fc*-1
  } else {
    linfc[x] <- 2**logfc.sel[x]
  }
}

# combine results to data frame
res.ext <- cbind(res, linfc)
# re-order columns
res.ext <- res.ext[, c(1,4,2,3)]
colnames(res.ext) <- c("Gene_symbol", "fold_change_in_Lawlor_et_al_2016", "fold_change_in_Segerstolpe_e
                       "rank_test_pvals_in_Segerstolpe_et_al_2016")
# sort results by p value
ord <- order(res.ext$`rank_test_pvals_in_Segerstolpe _et_al_2016`, decreasing = FALSE)
res.fin <- res.ext[ord,]
# write results to file
write.xlsx(x = res.fin, file = "Supplemental_Table_S12.xlsx", sheetName = name)

# specify cell type
cell_type = "delta"

# Use samples with phenotype info
```

```
w.cont <- wang.anns[wang.anns$Health == "control" & wang.anns$Cell_Type %in% cell_type & wang.anns$Age =
w.t2d <- wang.anns[wang.anns$Health == "T2D" & wang.anns$Cell_Type %in% cell_type & wang.anns$Age == "ad
w.cont <- as.matrix(w.cont)
w.cont <- as.data.frame(w.cont)
w.t2d <- as.matrix(w.t2d)
w.t2d <- as.data.frame(w.t2d)
# combine anns
w.sel <- rbind(w.cont, w.t2d)
# selected counts
wang.exp.ND <- wang.exp[, rownames(w.cont)]
wang.exp.T2D <- wang.exp[, rownames(w.t2d)]
name <- paste(cell_type, "DEGs_in_Wang", sep = "_")

# Load genes to compare
genelist <- read.csv("/Users/lawlon/Documents/Final_RNA_Seq_3/Differential_Expression_3/Single_Cell/T2D_
                     header = T, check.names = F, row.names = 1, stringsAsFactors = F)

# find genes that intersect between lists
gene.int <- intersect(genelist$Associated.Gene.Name, rownames(wang.exp.ND))
# extract genes of interest for both groups
wang.nont2d.sel <- wang.exp.ND[gene.int,]
wang.t2d.sel <- wang.exp.T2D[gene.int,]

pvals <- numeric(length = nrow(wang.nont2d.sel))
# average fold change of T2D vs ND
fc <- numeric(length = nrow(wang.nont2d.sel))
for(i in 1:length(pvals))
{
  pvals[i] <- wilcox.test(wang.nont2d.sel[i,],wang.t2d.sel[i,])$p.value
  fc[i] <- foldchange(num = mean(wang.t2d.sel[i,]), denom = mean(wang.nont2d.sel[i,]))
}
symbol <- rownames(wang.nont2d.sel)
# create resulting data frame
wang.res <- data.frame(symbol = symbol, fold_change = fc, rank.test.pvals = pvals)
# convert logFC to linear fold change of genes in our dataset
ids <- NULL
for (i in 1:length(wang.res$symbol)) {
  idx <- which(genelist$Associated.Gene.Name == wang.res$symbol[i])
  ids <- c(ids, idx)
}

# get selected logFC
logfc.sel <- genelist$logFC[ids]
# calculate normal fold change
linfc <- logfc.sel
for (x in 1:length(logfc.sel)) {
  if (logfc.sel[x] < 0 ) {
    fc <- 2**(abs(logfc.sel[x]))
    linfc[x] <- fc*-1
  } else {
    linfc[x] <- 2**logfc.sel[x]
  }
}
```

```
# combine results to data frame
wang.res.ext <- cbind(wang.res, linfc)
# re-order columns
wang.res.ext <- wang.res.ext[, c(1,4,2,3)]
colnames(wang.res.ext) <- c("Gene_symbol", "fold_change_in_Lawlor_et_al_2016", "fold_change_in_Wang_et_a
                        "rank_test_pvals_in_Wang_et_al_2016")
# sort results by p value
wang.ord <- order(wang.res.ext$rank_test_pvals_in_Wang_et_al_2016, decreasing = FALSE)
wang.res.fin <- wang.res.ext[wang.ord,]
# write results to file
write.xlsx(x = wang.res.fin, file = "Supplemental_Table_S12.xlsx", sheetName = name,
           append = TRUE)

# Find genes with same trend in fold change and have rank test p-value less than 0.05
# For segerstolpe dataset (Sandberg group)
sand.res.pos <- res.fin[res.fin[,3] > 0,]
sand.res.pos.sig <- sand.res.pos[sand.res.pos[,4] < 0.05,]
sand.res.neg <- res.fin[res.fin[,3] < 0,]
sand.res.neg.sig <- sand.res.neg[sand.res.neg[,4] < 0.05,]
# genelist pos and neg enriched
genelist.pos <- genelist[genelist$logFC > 0,]
genelist.neg <- genelist[genelist$logFC < 0,]
# find intersection of genes
sand.int.pos <- intersect(sand.res.pos.sig[,1], genelist.pos$Associated.Gene.Name)
sand.int.neg <- intersect(sand.res.neg.sig[,1], genelist.neg$Associated.Gene.Name)
sand.int.genes <- c(sand.int.neg, sand.int.pos)
# extract overlapping genes
sand.gen.ids <- which(res.fin[,1] %in% sand.int.genes)
res.fin.ov.beta <- res.fin[sand.gen.ids,]
# rank by p-value
sand.ord <- order(res.fin.ov.beta[,4], decreasing = FALSE)
sand.res.fin.ov.beta <- res.fin.ov.beta[sand.ord,]
# write results to file
write.xlsx(x = sand.res.fin.ov.beta, file = "Supplemental_Table_S12.xlsx",
           sheetName = "delta_DEGs_Lawlor_Segerstolpe_Ov", append = TRUE)

# No delta cell overlap found with wang dataset
```

Session Information

```
suppressPackageStartupMessages(library(Biobase))
suppressPackageStartupMessages(library(gtools))
suppressPackageStartupMessages(library(xlsx))
rm(list = ls())
library(Biobase)
library(gtools)
library(xlsx)
sessionInfo()
```

```
## R version 3.3.0 (2016-05-03)
## Platform: x86_64-apple-darwin13.4.0 (64-bit)
## Running under: OS X 10.11.6 (El Capitan)
```

```
##
## locale:
## [1] en_US.UTF-8/en_US.UTF-8/en_US.UTF-8/C/en_US.UTF-8/en_US.UTF-8
##
## attached base packages:
## [1] parallel  stats     graphics  grDevices utils     datasets  methods
## [8] base
##
## other attached packages:
## [1] xlsx_0.5.7          xlsxjars_0.6.1      rJava_0.9-8
## [4] gtools_3.5.0        Biobase_2.32.0      BiocGenerics_0.18.0
##
## loaded via a namespace (and not attached):
##  [1] Rcpp_0.12.7     digest_0.6.10   assertthat_0.1  formatR_1.4
##  [5] magrittr_1.5    evaluate_0.10   stringi_1.1.2   rmarkdown_1.1
##  [9] tools_3.3.0     stringr_1.1.0   yaml_2.1.13     htmltools_0.3.5
## [13] knitr_1.14      tibble_1.2
```
