# Supplementary material for: Single-cell transcriptomes identify human islet cell signatures and reveal cell-type–specific expression changes in type 2 diabetes
Source: Genome Res. 2017 Feb;27(2):208–22. doi: 10.1101/gr.212720.116 (PMC5287227; doi:10.1101/gr.212720.116)
Supplement: Supplemental Material [file supp_gr.212720.116_Supplemental_Methods_Source_Code.zip › Supplemental_Methods_Source_Code/Supplemental_Table_Source_Code/Supplemental_Table_S4_Source_Code.pdf]

Unsupervised Hierarchical Clustering of Single Cell Data

Introduction

This report describes the methods used to obtain the highly expressed genes used for unsupervised hierarchical clustering of the single cell data. Three different gene sets were chosen to cluster the non-diabetic, diabetic, and combined cohorts respectively. For each cohort, genes with log2(CPM) expression values greater than 10.5 in at least one sample were selected.

```
# Load libraries
suppressPackageStartupMessages(library(Biobase))
suppressPackageStartupMessages(library(edgeR))
suppressPackageStartupMessages(library(xlsx))
library(Biobase)
library(edgeR)
library(xlsx)
set.seed(53079239)

# Load NonT2D single cell data
fname = "NonT2D"
setwd("/Users/lawlon/Documents/Final_RNA_Seq_3/Data/")
load("nonT2D.rdata")
p.anns <- featureData(cnts.eset)
probe.anns <- as(p.anns,"data.frame")
ND.anns <- pData(cnts.eset)
# Remove multiples and keep all other groups
ND.sel <- ND.anns[ND.anns$cell.type %in% c("INS", "PPY", "GCG", "SST", "COL1A1", "KRT19", "PRSS1", "none[illegible]

# Obtain counts
ND.counts <- exprs(cnts.eset)
# Calculate log2 cpm
cpms <- cpm(x = ND.counts)
data <- log2(cpms+1)
data <- data[, rownames(ND.sel)]

# Filter gene by those with max CPM value greater than 10 (high expressed genes)
r.max <- apply(data,1,max)
ND.data.sel <- data[r.max > 10.5,]

# Combine probe anns with selected fpkm values
p.res <- probe.anns[rownames(ND.data.sel),]
ND.data.sel.exp <- cbind(p.res,ND.data.sel)

# Write selected gene matrix to file
write.xlsx(ND.data.sel.exp, file = "Supplemental_Table_S4.xlsx",
           sheetName = paste(fname, "genes_selected", sep = "_"))

# Select genes for T2D data set
fname = "T2D"
setwd("/Users/lawlon/Documents/Final_RNA_Seq_3/Data/")
load("T2D.rdata")
p.anns <- featureData(cnts.eset)
```

```
probe.anns <- as(p.anns,"data.frame")
T2D.anns <- pData(cnts.eset)
# Remove multiples and keep all other groups
T2D.sel <- T2D.anns[T2D.anns$cell.type %in% c("INS", "PPY", "GCG", "SST", "COL1A1", "KRT19", "PRSS1", "

# Obtain counts
T2D.counts <- exprs(cnts.eset)
# Calculate log2 cpm
T2D.cpms <- cpm(x = T2D.counts)
T2D.data <- log2(T2D.cpms+1)
T2D.data <- T2D.data[, rownames(T2D.sel)]

# Filter gene by those with max CPM value greater than 10 (high expressed genes)
r.max <- apply(T2D.data,1,max)
T2D.data.sel <- T2D.data[r.max > 10.5,]

# Combine probe anns with selected fpkm values
p.res <- probe.anns[rownames(T2D.data.sel),]
T2D.data.sel.exp <- cbind(p.res,T2D.data.sel)

# Write selected gene matrix to file
write.xlsx(T2D.data.sel.exp, file = "Supplemental_Table_S4.xlsx",
           sheetName = paste(fname, "genes_selected", sep = "_"),
           append = TRUE)

# Select genes for combined NonT2D and T2D dataset
# combine expression datasets
fname = "NonT2D_and_T2D"
comb.data <- cbind(data, T2D.data)

# Filter gene by those with max CPM value greater than 10 (high expressed genes)
r.max <- apply(comb.data,1,max)
comb.data.sel <- comb.data[r.max > 10.5,]

# Combine probe anns with selected fpkm values
p.res <- probe.anns[rownames(comb.data.sel),]
comb.data.sel.exp <- cbind(p.res,comb.data.sel)

# Write selected gene matrix to file
write.xlsx(comb.data.sel.exp, file = "Supplemental_Table_S4.xlsx",
           sheetName = paste(fname, "genes_selected", sep = "_"),
           append = TRUE)
```

Session Information

```
suppressPackageStartupMessages(library(Biobase))
suppressPackageStartupMessages(library(edgeR))
```

```
## Warning: package 'limma' was built under R version 3.3.1
```

```
suppressPackageStartupMessages(library(xlsx))
library(Biobase)
```

```r
library(edgeR)
library(xlsx)
sessionInfo()
```

```
## R version 3.3.0 (2016-05-03)
## Platform: x86_64-apple-darwin13.4.0 (64-bit)
## Running under: OS X 10.11.6 (El Capitan)
##
## locale:
## [1] en_US.UTF-8/en_US.UTF-8/en_US.UTF-8/C/en_US.UTF-8/en_US.UTF-8
##
## attached base packages:
## [1] parallel  stats     graphics  grDevices utils     datasets  methods
## [8] base
##
## other attached packages:
## [1] xlsx_0.5.7          xlsxjars_0.6.1      rJava_0.9-8
## [4] edgeR_3.14.0        limma_3.28.21       Biobase_2.32.0
## [7] BiocGenerics_0.18.0
##
## loaded via a namespace (and not attached):
##  [1] Rcpp_0.12.7     digest_0.6.10   assertthat_0.1  formatR_1.4
##  [5] magrittr_1.5    evaluate_0.10   stringi_1.1.2   rmarkdown_1.1
##  [9] tools_3.3.0     stringr_1.1.0   yaml_2.1.13     htmltools_0.3.5
## [13] knitr_1.14      tibble_1.2
```
