# Supplementary material for: Single-cell transcriptomes identify human islet cell signatures and reveal cell-type–specific expression changes in type 2 diabetes
Source: Genome Res. 2017 Feb;27(2):208–22. doi: 10.1101/gr.212720.116 (PMC5287227; doi:10.1101/gr.212720.116)
Supplement: Supplemental Material [file supp_gr.212720.116_Supplemental_Methods_Source_Code.zip › Supplemental_Methods_Source_Code/Supplemental_Table_Source_Code/Supplemental_Table_S5_Source_Code.pdf]

Computational assignment of cell cycle phases shows islet single cell samples are not actively undergoing mitosis

Introduction

We implemented the R-package scran_1.04 to investigate whether our dataset contained proliferative islet cells. Specifically, we implemented the function "cyclone" to computationally assign single cell samples into cell cycle phases (G1, G2/M, or S phase) based on their expression of human cell cycle markers.

Islet Single Cells are not actively dividing

```
rm(list = ls())
suppressPackageStartupMessages(library(Biobase))
suppressPackageStartupMessages(library(scran))
library(Biobase)
library(scran)
# Load in single cell data
setwd("/Users/lawlon/Documents/Final_RNA_Seq_3/Data/")
load("nonT2D.rdata")
p.anns <- featureData(cnts.eset)
probe.anns <- as(p.anns,"data.frame")
ND.anns <- pData(cnts.eset)
# Remove multiples and keep all other groups
ND.sel <- ND.anns[ND.anns$cell.type %in% c("INS", "PPY", "GCG", "SST", "COL1A1", "KRT19", "PRSS1", "non
# selected exp data
ND.counts <- exprs(cnts.eset)
ND.cnts.sel <- ND.counts[, rownames(ND.sel)]
load("T2D.rdata")
T2D.anns <- pData(cnts.eset)
# Remove multiples and keep all other groups
T2D.sel <- T2D.anns[T2D.anns$cell.type %in% c("INS", "PPY", "GCG", "SST", "COL1A1", "KRT19", "PRSS1", "
# selected exp data
T2D.counts <- exprs(cnts.eset)
T2D.cnts.sel <- T2D.counts[, rownames(T2D.sel)]
# Combine sample anns and expression data
all.counts <- cbind(ND.cnts.sel, T2D.cnts.sel)
s.anns.sel <- rbind(ND.sel, T2D.sel)
# create sce object
sce <- newSCESet(countData=data.frame(all.counts))
# normalize cell specific biases
sce <- computeSumFactors(sce, sizes=c(20, 40, 60, 80))
summary(sizeFactors(sce))
sce <- normalize(sce)
colnames(sce) <- colnames(all.counts)
# Get normalized exp data
scran.data <- exprs(sce)
colnames(scran.data) <- colnames(all.counts)
```

```r
# load human cell cycle markers
hs.pairs <- readRDS(system.file("exdata", "human_cycle_markers.rds", package="scran"))
assigned <- cyclone(sce, pairs=hs.pairs)
head(assigned$scores)

phase <- rep("S", ncol(sce))
phase[assigned$scores$G1 > 0.5] <- "G1"
phase[assigned$scores$G2M > 0.5] <- "G2M"
phase[assigned$scores$G1 > 0.5 & assigned$scores$G2M > 0.5] <- "unknown"
table(phase)

# append cell cycle info to sample anns
s.cc <- cbind(s.anns.sel, phase)
# table of cell type vs cell cycle phase
tab <- table(s.cc$cell.type, s.cc$phase)
```

Session Information

```r
suppressPackageStartupMessages(library(Biobase))
suppressPackageStartupMessages(library(scran))
```

```
## Warning: package 'scran' was built under R version 3.3.1
```

```
## Warning: package 'BiocParallel' was built under R version 3.3.1
```

```r
library(Biobase)
library(scran)
sessionInfo()
```

```
## R version 3.3.0 (2016-05-03)
## Platform: x86_64-apple-darwin13.4.0 (64-bit)
## Running under: OS X 10.11.6 (El Capitan)
##
## locale:
## [1] en_US.UTF-8/en_US.UTF-8/en_US.UTF-8/C/en_US.UTF-8/en_US.UTF-8
##
## attached base packages:
## [1] parallel  stats     graphics  grDevices utils     datasets  methods
## [8] base
##
## other attached packages:
## [1] scran_1.0.4         scater_1.0.4        ggplot2_2.1.0
## [4] BiocParallel_1.6.6  Biobase_2.32.0      BiocGenerics_0.18.0
##
## loaded via a namespace (and not attached):
##  [1] Rcpp_0.12.7           formatR_1.4           plyr_1.8.4
##  [4] zlibbioc_1.18.0       viridis_0.3.4         bitops_1.0-6
##  [7] tools_3.3.0           biomaRt_2.28.0        digest_0.6.10
## [10] lattice_0.20-34       rhdf5_2.16.0          evaluate_0.10
## [13] RSQLite_1.0.0         tibble_1.2            gtable_0.2.0
## [16] Matrix_1.2-7.1        shiny_0.14.1          DBI_0.5-1
## [19] yaml_2.1.13           gridExtra_2.2.1       dplyr_0.5.0
## [22] stringr_1.1.0         knitr_1.14            IRanges_2.6.1
```

```
## [25] S4Vectors_0.10.3      stats4_3.3.0          grid_3.3.0
## [28] shinydashboard_0.5.3  data.table_1.9.6      R6_2.2.0
## [31] AnnotationDbi_1.34.4  XML_3.98-1.4          rmarkdown_1.1
## [34] limma_3.28.21         reshape2_1.4.1        edgeR_3.14.0
## [37] magrittr_1.5          matrixStats_0.51.0    scales_0.4.0
## [40] htmltools_0.3.5       dynamicTreeCut_1.63-1 tximport_1.0.3
## [43] assertthat_0.1        mime_0.5              colorspace_1.2-7
## [46] xtable_1.8-2          httpuv_1.3.3          stringi_1.1.2
## [49] RCurl_1.95-4.8        munsell_0.4.3         rjson_0.2.15
## [52] chron_2.3-47          zoo_1.7-13
```
