# Supplementary material for: Single-cell transcriptomes identify human islet cell signatures and reveal cell-type–specific expression changes in type 2 diabetes
Source: Genome Res. 2017 Feb;27(2):208–22. doi: 10.1101/gr.212720.116 (PMC5287227; doi:10.1101/gr.212720.116)
Supplement: Supplemental Material [file supp_gr.212720.116_Supplemental_Methods_Source_Code.zip › Supplemental_Methods_Source_Code/Supplemental_Table_Source_Code/Supplemental_Table_S6_Source_Code.pdf]

Differential Gene Expression Analysis

Introduction

Differential expression analysis of raw RNA-seq counts was performed using the Bioconductor package, edgeR. This software uses an overdispersed Poisson model to account for the biological and technical variability as well as empirical Bayes methods to moderate the degree of dispersion across genes (REFERENCE). For all single cell analyses, gender was used as a blocking factor to account for variability between male and female patient islets. In each edgeR comparison, protein coding genes and long non-coding RNAs with 20 or more counts in at least 20% of either cell type population being compared or at least a minimum of 3 cells were used. Differentially expressed genes with a false discovery rate (FDR) value less than 5% were regarded as significant results.

Endocrine cell signature genes were identified by first performing the above differential expression analysis procedure between each endocrine cell type (e.g. Beta vs Alpha, Beta vs Delta, and Beta vs Gamma) in our non-diabetic data. For each pairwise comparison, a list of differentially expressed genes was produced. Afterwards, the intersection of these pairwise comparison gene lists was performed to identify genes that were exclusively differentially expressed in the cell type. Intersected genes with a positive log fold change were classified as being uniquely expressed in the cell type. Exocrine cell signature genes were identified in a similar manner. Similar pairwise differential expression analyses were performed using the single cell data from Type 2 diabetic patients.

In addition, differential expression analyses between Type 2 diabetic and non-diabetic single cell data were performed for the same cell types (e.g. T2D Beta cells vs ND Beta cells) to identify cell type specific differences in gene expression that may be contributing to the Type 2 diabetes pathology.

Reference: Robinson, M. D., D. J. Mccarthy, and G. K. Smyth. "EdgeR: A Bioconductor Package for Differential Expression Analysis of Digital Gene Expression Data." Bioinformatics 26.1 (2009): 139-40.

Differential Expression Analyses of Endocrine Non-Diabetic Single Cells using EdgeR

```r
# Load libraries
suppressPackageStartupMessages(library(Biobase))
suppressPackageStartupMessages(library(edgeR))
suppressPackageStartupMessages(library(locfit))
rm(list = ls())
# Load libraries
library(Biobase)
library(edgeR)
library(locfit)
# Set working directory
setwd("/Users/lawlon/Documents/Final_RNA_Seq_3/Data/")
# Load in Single Cell RNA-seq data
load("nonT2D.rdata")
# Probe annotation data
p.anns <- as(featureData(cnts.eset), "data.frame")
# Sample annotation data
s.anns <- pData(cnts.eset)
# Expression data
cnts.exprs <- exprs(cnts.eset)
```

```
# Specify target of interest (grp2 vs grp1)
name = "EdgeR.Robust.NonT2D"

# Specify markers and cell names
grp2 <- c("INS", "GCG", "SST", "PPY")
grp1 <- c("INS", "GCG", "SST", "PPY")

name2 <- c("Beta", "Alpha", "Delta", "Gamma")
name1 <- c("Beta", "Alpha", "Delta", "Gamma")

# Loop
for (i in 1: length(grp2)) {
  for (j in 1:length(grp1)) {
    if (grp2[i] != grp1[j]) {
      # Do Analysis
      # Extract cell type of interest
      index <- which(s.anns[, "cell.type"] == grp2[i])
      s2.anns <- s.anns[index,]
      # Male and female samples
      s2.m <- s2.anns[s2.anns$run %in% c("1st","2nd","3rd","4th","8th","6th","7th"),]
      s2.f <- s2.anns[s2.anns$run %in% c("5th","9th","10t","11t","12t","13t"),]

      # Reform eset to only sample of interest
      exprs <- cnts.exprs[, index]
      # Reform other eset to have all other endocrine non-INS samples
      index1 <- which(s.anns[, "cell.type"] == grp1[j])
      others <- cnts.exprs[, index1]

      # Obtain sample anns for all other endocrine
      s1.anns <- s.anns[colnames(others),]
      # Male and female samples
      s1.m <- s1.anns[s1.anns$run %in% c("1st","2nd","3rd","4th","8th","6th","7th"),]
      s1.f <- s1.anns[s1.anns$run %in% c("5th","9th","10t","11t","12t","13t"),]

      # assign groups to the samples in the counts table
      g <- factor(c(rep.int("grp1", times=dim(others)[2]),
                    rep.int("grp2", times=dim(exprs)[2])))

      # Control group first then treatment group
      both <- cbind(others, exprs)

      # Extract sample anns of all endocrine cells
      s.endo <- s.anns[colnames(both),]

      # Make a design matrix of cell type and gender
      cell <- character(length = nrow(s.endo))
      # Need to make sure grp variables are as character, not factor
      cell[s.endo$cell.type %in% c(as.character(grp2[i]))] <- as.character(grp2[i])
      cell[s.endo$cell.type %in% c(as.character(grp1[j]))] <- as.character(grp1[j])

      #Make vector of gender info
      # Runs 5,9-13 are female, 1-4, 7-8 are male
      gender <- character(length = nrow(s.endo))
```

```r
gender[s.endo$run %in% c("5th","9th","10t","11t","12t","13t")] <- "F"
gender[s.endo$run %in% c("1st","2nd","3rd","4th","8th","6th","7th")] <- "M"

# Info for generalized linear model
Status <- factor(cell)
# Specify which group is control
contrasts(Status) <- contr.treatment(levels(Status),
                                     base = which(levels(Status) == grp1[j]))
# Vector of gender information for each sample
Gender <- factor(gender)
# Design matrix has column of intercepts all 1's
# Also columns for gender and phenotype indicating assignment of samples
design <- model.matrix(~Gender + Status)

# Counts filtering for each sample
# Set threshold so that you get max 3 cells or at least 20% of all cells
exprs.bin <- exprs
exprs.bin[exprs < 20] <- 0
exprs.bin[exprs >= 20] <- 1
exp <- apply(exprs.bin,1,sum)
exprs.th <- max(3,0.2*ncol(exprs))
exprs.sel <- exprs[exp > exprs.th,]

# Counts filtering for other sample type
# Set threshold so that you get max 3 cells or at least 20% of all cells
others.bin <- others
others.bin[others < 20] <- 0
others.bin[others >= 20] <- 1
others.exp <- apply(others.bin,1,sum)
others.th <- max(3,0.2*ncol(others))
others.sel <- others[others.exp > others.th,]

# Find union of genes
un <- union(rownames(others.sel), rownames(exprs.sel))
cnts.sel <- both[un,]

# EdgeR analysis, using robust settings
cds <- DGEList(counts = cnts.sel, group = g)
y <- calcNormFactors(cds)
y <- estimateDisp(y,design, robust = TRUE)
fit <- glmFit(y,design)
lrt <- glmLRT(fit,coef=3)
diff.dat <- topTags(lrt,n = nrow(cnts.sel))
res <- diff.dat$table

p.sel <- p.anns[rownames(res),c("Description",
                                "Associated.Gene.Name","Gene.Biotype")]
res.ext <- cbind(p.sel,res)

# Calculate avelogCPM for nonT2D male
if (dim(s1.m)[1] == 0) {
  e1.cpm <- rep.int(0, times = dim(cnts.sel)[1])
} else {
```

```
    e1.cpm <- aveLogCPM(cnts.sel[,rownames(s1.m)],
                        normalized.lib.sizes = FALSE, prior.count = 2)
  }
  # Assign names to vectors
  names(e1.cpm) <- rownames(cnts.sel)
  # T2D males
  if (dim(s2.m)[1] == 0) {
    e2.cpm <- rep.int(0, times = dim(cnts.sel)[1])
  } else {
    e2.cpm <- aveLogCPM(cnts.sel[,rownames(s2.m)],
                        normalized.lib.sizes = FALSE, prior.count = 2)
    names(e2.cpm) <- rownames(cnts.sel)
  }
  # nonT2D females
  if (dim(s1.f)[1] == 0) {
    e3.cpm <- rep.int(0, times = dim(cnts.sel)[1])
  } else {
    e3.cpm <- aveLogCPM(cnts.sel[,rownames(s1.f)],
                        normalized.lib.sizes = FALSE, prior.count = 2)
  }
  names(e3.cpm) <- rownames(cnts.sel)
  # T2D females
  if (dim(s2.f)[1] == 0) {
    e4.cpm <- rep.int(0, times = dim(cnts.sel)[1])
  } else {
    e4.cpm <- aveLogCPM(cnts.sel[,rownames(s2.f)],
                        normalized.lib.sizes = FALSE, prior.count = 2)
  }
  names(e4.cpm) <- rownames(cnts.sel)

  # Compute ave log cpm for the first cell group (grp2)
  grp2.cpm <- aveLogCPM(cnts.sel[, rownames(s2.anns)],
                        normalized.lib.sizes = FALSE, prior.count = 2)
  names(grp2.cpm) <- rownames(cnts.sel)

  # Compute ave log cpm for the second cell group (grp1)
  grp1.cpm <- aveLogCPM(cnts.sel[, rownames(s1.anns)],
                        normalized.lib.sizes = FALSE, prior.count = 2)
  names(grp1.cpm) <- rownames(cnts.sel)

  # Extract rownames of DE genes
  rn <- rownames(res.ext)
  e1.cpm <- e1.cpm[rn]
  e2.cpm <- e2.cpm[rn]
  e3.cpm <- e3.cpm[rn]
  e4.cpm <- e4.cpm[rn]
  grp2.cpm <- grp2.cpm[rn]
  grp1.cpm <- grp1.cpm[rn]

  res.ext <- cbind(res.ext, e1.cpm, e2.cpm, e3.cpm, e4.cpm, grp2.cpm, grp1.cpm)

  # Combine average logCPM for each group into results table
  names(res.ext)[names(res.ext) == "e2.cpm"] <- paste(name2[i], "Male", "avelogCPM", sep = " ")
```

```
      names(res.ext)[names(res.ext) == "e1.cpm"] <- paste(name1[j], "Male", "avelogCPM", sep = " ")
      names(res.ext)[names(res.ext) == "e3.cpm"] <- paste(name1[j], "Female", "avelogCPM", sep = " ")
      names(res.ext)[names(res.ext) == "e4.cpm"] <- paste(name2[i], "Female", "avelogCPM", sep = " ")
      names(res.ext)[names(res.ext) == "grp2.cpm"] <- paste(name2[i], "avelogCPM", sep = " ")
      names(res.ext)[names(res.ext) == "grp1.cpm"] <- paste(name1[j], "avelogCPM", sep = " ")
      # re-order the results matrix
      df <- res.ext[c("Description", "Associated.Gene.Name", "Gene.Biotype", "logFC",
                      "logCPM", paste(name2[i], " Male avelogCPM", sep=""),
                      paste(name1[j]," Male avelogCPM", sep = ""),
                      paste(name2[i], " Female avelogCPM", sep =""),
                      paste(name1[j]," Female avelogCPM", sep=""),
                      paste(name2[i], " avelogCPM", sep=""),
                      paste(name1[j], " avelogCPM", sep = ""), "LR", "PValue", "FDR")]

      # Output results table to text
      write.csv(x=df,file=paste(name, name2[i], "vs", name1[j], "all.genes.csv", sep="."))
      # Filter by FDR of less than 0.05, no logFC filter
      df.filter <- df[df$FDR < 0.05,]
      write.csv(x=df.filter, file = paste(name, name2[i], "vs", name1[j], "FDR.0.05.csv", sep = "."))
    }
  }
}
```

Differential Expression Analyses of Exocrine Non-Diabetic Single Cells using EdgeR

```
# Load libraries
rm(list = ls())
suppressPackageStartupMessages(library(Biobase))
suppressPackageStartupMessages(library(edgeR))
suppressPackageStartupMessages(library(locfit))
library(Biobase)
library(edgeR)
library(locfit)
# Set working directory
setwd("/Users/lawlon/Documents/Final_RNA_Seq_3/Data/")
# Load in Single Cell RNA-seq data
load("nonT2D.rdata")
# Probe annotation data
p.anns <- as(featureData(cnts.eset), "data.frame")
# Sample annotation data
s.anns <- pData(cnts.eset)
# Expression data
cnts.exprs <- exprs(cnts.eset)

# Specify target of interest (grp2 vs grp1)
name = "EdgeR.Robust.NonT2D"

# Specify markers and cell names
grp2 <- c("COL1A1", "PRSS1", "KRT19")
grp1 <- c("COL1A1", "PRSS1", "KRT19")
```

```r
name2 <- c("Stellate", "Acinar", "Ductal")
name1 <- c("Stellate", "Acinar", "Ductal")

# Loop
for (i in 1: length(grp2)) {
  for (j in 1:length(grp1)) {
    if (grp2[i] != grp1[j]) {
      # Do Analysis
      # Extract cell type of interest
      index <- which(s.anns[, "cell.type"] == grp2[i])
      s2.anns <- s.anns[index,]
      # Male and female samples
      s2.m <- s2.anns[s2.anns$run %in% c("1st","2nd","3rd","4th","8th","6th","7th"),]
      s2.f <- s2.anns[s2.anns$run %in% c("5th","9th","10t","11t","12t","13t"),]

      # Reform eset to only sample of interest
      exprs <- cnts.exprs[, index]
      # Reform other eset to have all other endocrine non-INS samples
      index1 <- which(s.anns[, "cell.type"] == grp1[j])
      others <- cnts.exprs[, index1]

      # Obtain sample anns for all other endocrine
      s1.anns <- s.anns[colnames(others),]
      # Male and female samples
      s1.m <- s1.anns[s1.anns$run %in% c("1st","2nd","3rd","4th","8th","6th","7th"),]
      s1.f <- s1.anns[s1.anns$run %in% c("5th","9th","10t","11t","12t","13t"),]

      # assign groups to the samples in the counts table
      g <- factor(c(rep.int("grp1", times=dim(others)[2]),
                   rep.int("grp2", times=dim(exprs)[2])))

      # Control group first then treatment group
      both <- cbind(others, exprs)

      # Extract sample anns of all endocrine cells
      s.endo <- s.anns[colnames(both),]

      # Make a design matrix of cell type and gender
      cell <- character(length = nrow(s.endo))
      # Need to make sure grp variables are as character, not factor
      cell[s.endo$cell.type %in% c(as.character(grp2[i]))] <- as.character(grp2[i])
      cell[s.endo$cell.type %in% c(as.character(grp1[j]))] <- as.character(grp1[j])

      #Make vector of gender info
      # Runs 5,9-13 are female, 1-4, 7-8 are male
      gender <- character(length = nrow(s.endo))
      gender[s.endo$run %in% c("5th","9th","10t","11t","12t","13t")] <- "F"
      gender[s.endo$run %in% c("1st","2nd","3rd","4th","8th","6th","7th")] <- "M"

      # Info for generalized linear model
      Status <- factor(cell)
      # Specify which group is control
      contrasts(Status) <- contr.treatment(levels(Status),
```

```
                                   base = which(levels(Status) == grp1[j]))
# Vector of gender information for each sample
Gender <- factor(gender)
# Design matrix has column of intercepts all 1's
# Also columns for gender and phenotype indicating assignment of samples
design <- model.matrix(~Gender + Status)

# Counts filtering for each sample
# Set threshold so that you get max 3 cells or at least 20% of all cells
exprs.bin <- exprs
exprs.bin[exprs < 20] <- 0
exprs.bin[exprs >= 20] <- 1
exp <- apply(exprs.bin,1,sum)
exprs.th <- max(3,0.2*ncol(exprs))
exprs.sel <- exprs[exp > exprs.th,]

# Counts filtering for other sample type
# Set threshold so that you get max 3 cells or at least 20% of all cells
others.bin <- others
others.bin[others < 20] <- 0
others.bin[others >= 20] <- 1
others.exp <- apply(others.bin,1,sum)
others.th <- max(3,0.2*ncol(others))
others.sel <- others[others.exp > others.th,]

# Find union of genes
un <- union(rownames(others.sel), rownames(exprs.sel))
cnts.sel <- both[un,]

# EdgeR analysis, using robust settings
cds <- DGEList(counts = cnts.sel, group = g)
y <- calcNormFactors(cds)
y <- estimateDisp(y,design, robust = TRUE)
fit <- glmFit(y,design)
lrt <- glmLRT(fit,coef=3)
diff.dat <- topTags(lrt,n = nrow(cnts.sel))
res <- diff.dat$table

p.sel <- p.anns[rownames(res),c("Description",
                                "Associated.Gene.Name","Gene.Biotype")]
res.ext <- cbind(p.sel,res)

# Calculate avelogCPM for nonT2D male
if (dim(s1.m)[1] == 0) {
  e1.cpm <- rep.int(0, times = dim(cnts.sel)[1])
} else {
  e1.cpm <- aveLogCPM(cnts.sel[,rownames(s1.m)],
                      normalized.lib.sizes = FALSE, prior.count = 2)
}
# Assign names to vectors
names(e1.cpm) <- rownames(cnts.sel)
# T2D males
if (dim(s2.m)[1] == 0) {
```

```
    e2.cpm <- rep.int(0, times = dim(cnts.sel)[1])
  } else {
    e2.cpm <- aveLogCPM(cnts.sel[,rownames(s2.m)],
                        normalized.lib.sizes = FALSE, prior.count = 2)
    names(e2.cpm) <- rownames(cnts.sel)
  }
  # nonT2D females
  if (dim(s1.f)[1] == 0) {
    e3.cpm <- rep.int(0, times = dim(cnts.sel)[1])
  } else {
    e3.cpm <- aveLogCPM(cnts.sel[,rownames(s1.f)],
                        normalized.lib.sizes = FALSE, prior.count = 2)
  }
  names(e3.cpm) <- rownames(cnts.sel)
  # T2D females
  if (dim(s2.f)[1] == 0) {
    e4.cpm <- rep.int(0, times = dim(cnts.sel)[1])
  } else {
    e4.cpm <- aveLogCPM(cnts.sel[,rownames(s2.f)],
                        normalized.lib.sizes = FALSE, prior.count = 2)
  }
  names(e4.cpm) <- rownames(cnts.sel)

  # Compute ave log cpm for the first cell group (grp2)
  grp2.cpm <- aveLogCPM(cnts.sel[, rownames(s2.anns)],
                        normalized.lib.sizes = FALSE, prior.count = 2)
  names(grp2.cpm) <- rownames(cnts.sel)

  # Compute ave log cpm for the second cell group (grp1)
  grp1.cpm <- aveLogCPM(cnts.sel[, rownames(s1.anns)],
                        normalized.lib.sizes = FALSE, prior.count = 2)
  names(grp1.cpm) <- rownames(cnts.sel)

  # Extract rownames of DE genes
  rn <- rownames(res.ext)
  e1.cpm <- e1.cpm[rn]
  e2.cpm <- e2.cpm[rn]
  e3.cpm <- e3.cpm[rn]
  e4.cpm <- e4.cpm[rn]
  grp2.cpm <- grp2.cpm[rn]
  grp1.cpm <- grp1.cpm[rn]

  res.ext <- cbind(res.ext, e1.cpm, e2.cpm, e3.cpm, e4.cpm, grp2.cpm, grp1.cpm)

  # Combine average logCPM for each group into results table
  names(res.ext)[names(res.ext) == "e2.cpm"] <- paste(name2[i], "Male", "avelogCPM", sep = " ")
  names(res.ext)[names(res.ext) == "e1.cpm"] <- paste(name1[j], "Male", "avelogCPM", sep = " ")
  names(res.ext)[names(res.ext) == "e3.cpm"] <- paste(name1[j], "Female", "avelogCPM", sep = " ")
  names(res.ext)[names(res.ext) == "e4.cpm"] <- paste(name2[i], "Female", "avelogCPM", sep = " ")
  names(res.ext)[names(res.ext) == "grp2.cpm"] <- paste(name2[i], "avelogCPM", sep = " ")
  names(res.ext)[names(res.ext) == "grp1.cpm"] <- paste(name1[j], "avelogCPM", sep = " ")
  # re-order the results matrix
  df <- res.ext[c("Description", "Associated.Gene.Name", "Gene.Biotype", "logFC",
```

```
                      "logCPM", paste(name2[i], " Male avelogCPM", sep=""),
                      paste(name1[j]," Male avelogCPM", sep = ""),
                      paste(name2[i], " Female avelogCPM", sep =""),
                      paste(name1[j]," Female avelogCPM", sep=""),
                      paste(name2[i], " avelogCPM", sep=""),
                      paste(name1[j], " avelogCPM", sep = ""), "LR", "PValue", "FDR")]

      # Output results table to text
      write.csv(x=df,file=paste(name, name2[i], "vs", name1[j], "all.genes.csv", sep="."))
      # Filter by FDR of less than 0.05, no logFC filter
      df.filter <- df[df$FDR < 0.05,]
      write.csv(x=df.filter, file = paste(name, name2[i], "vs", name1[j], "FDR.0.05.csv", sep = "."))
    }
  }
}
```

Differential Expression Analyses of Type 2 Diabetic and Non-Diabetic Single Cells using EdgeR

```
# Load libraries
rm(list=ls())
suppressPackageStartupMessages(library(Biobase))
suppressPackageStartupMessages(library(edgeR))
suppressPackageStartupMessages(library(locfit))
library(edgeR)
library(Biobase)
library(locfit)
setwd("/Users/lawlon/Documents/Final_RNA_Seq_3/Data/")

# Specify cell type of interest
name = "EdgeR.Robust.T2D.vs.NonT2D"

# List marker genes
type <- c("INS", "GCG", "SST", "PPY", "COL1A1", "PRSS1", "KRT19")
name1 <- c("Beta", "Alpha", "Delta", "Gamma", "Stellate", "Acinar", "Ductal")

for (i in 1:length(type)) {
  # Load the ND data, get probe anns, sample anns
  load("nonT2D.rdata")
  probe.anns <- as(featureData(cnts.eset),"data.frame")
  nonT2D.beta.eset <- cnts.eset[,cnts.eset$cell.type %in% c(type[i])]
  nonT2D.anns <- pData(nonT2D.beta.eset)
  # Male and female samples
  nonT2D.m <- nonT2D.anns[nonT2D.anns$run %in% c("1st","2nd","3rd","4th","8th","6th","7th"),]
  nonT2D.f <- nonT2D.anns[nonT2D.anns$run %in% c("5th","9th","10t","11t","12t","13t"),]

  # Separate cpm
  ND.counts <- exprs(cnts.eset)

  #Load the T2D data
  load("T2D.rdata")
  t2d.probe.anns <- as(featureData(cnts.eset),"data.frame")
```

```r
T2D.beta.eset <- cnts.eset[,cnts.eset$cell.type %in% c(type[i])]
T2D.anns <- pData(T2D.beta.eset)
# Divide into male and female samples
T2D.m <- T2D.anns[T2D.anns$run %in% c("1st","2nd","3rd","4th","8th","6th","7th"),]
T2D.f <- T2D.anns[T2D.anns$run %in% c("5th","9th","10t","11t","12t","13t"),]

# Separate counts
T2D.counts <- exprs(cnts.eset)

# Combine sample anns for ND and T2D
sample.anns <- rbind(nonT2D.anns,T2D.anns)

# Combine counts for ND and T2D
cnts <- cbind(exprs(nonT2D.beta.eset),exprs(T2D.beta.eset))
# Make vector of phenotype info
# Runs 1,-3, 5-9 are ND, runs 10-13 and 4 are T2D
phenotype <- character(length = nrow(sample.anns))
phenotype[sample.anns$run %in% c("1st","2nd","3rd","5th","6th","7th","8th","9th")] <- "nonT2D"
phenotype[sample.anns$run %in% c("10t","11t","12t","13t","4th")] <- "T2D"

#Make vector of gender info
# Runs 5,9-13 are female, 1-4, 7-8 are male
gender <- character(length = nrow(sample.anns))
gender[sample.anns$run %in% c("5th","9th","10t","11t","12t","13t")] <- "F"
gender[sample.anns$run %in% c("1st","2nd","3rd","4th","8th","6th","7th")] <- "M"

# Info for generalized linear model
Status <- factor(phenotype)
Gender <- factor(gender)
# Design matrix has column of intercepts all 1's
# Also columns for gender and phenotype indicating assignment of samples
design <- model.matrix(~Gender + Status)

# Apply filter: counts > 20 in at least 20 percent of cells
T2D.cnts <- exprs(T2D.beta.eset)
ND.cnts <- exprs(nonT2D.beta.eset)
# Counts filtering for T2D
T2D.bin <- T2D.cnts
T2D.bin[T2D.cnts < 20] <- 0
T2D.bin[T2D.cnts >= 20] <- 1
T2D.exp <- apply(T2D.bin,1,sum)
T2D.th <- max(3,0.2*ncol(T2D.cnts))
T2D.sel <- T2D.cnts[T2D.exp > T2D.th,]

# Counts filtering for NonT2D
ND.bin <- ND.cnts
ND.bin[ND.cnts < 20] <- 0
ND.bin[ND.cnts >= 20] <- 1
ND.exp <- apply(ND.bin,1,sum)
ND.th <- max(3,0.2*ncol(ND.cnts))
ND.sel <- ND.cnts[ND.exp > ND.th,]

# Find union of genes
```

```r
un <- union(rownames(ND.sel), rownames(T2D.sel))
cnts.sel <- cnts[un,]

cds <- DGEList(counts = cnts.sel)
y <- calcNormFactors(cds)
y <- estimateDisp(y,design, robust = TRUE)
fit <- glmFit(y,design)
lrt <- glmLRT(fit,coef=3)
diff.dat <- topTags(lrt,n = nrow(cnts.sel))
res <- diff.dat$table

p.sel <- probe.anns[rownames(res),c("Description",
                                    "Associated.Gene.Name","Gene.Biotype")]
res.ext <- cbind(p.sel,res)

# Calculate avelogCPM for nonT2D male
e1.cpm <- aveLogCPM(cnts[,rownames(nonT2D.m)],
                    normalized.lib.sizes = FALSE, prior.count = 2)
# Assign names to vectors
names(e1.cpm) <- rownames(cnts)
# T2D males
if (dim(T2D.m)[1] == 0) {
  e2.cpm <- rep.int(0, times = dim(cnts)[1])
} else {
  e2.cpm <- aveLogCPM(cnts[,rownames(T2D.m)],
                      normalized.lib.sizes = FALSE, prior.count = 2)
  names(e2.cpm) <- rownames(cnts)
}
# nonT2D females
e3.cpm <- aveLogCPM(cnts[,rownames(nonT2D.f)],
                    normalized.lib.sizes = FALSE, prior.count = 2)
names(e3.cpm) <- rownames(cnts)
# T2D females
e4.cpm <- aveLogCPM(cnts[,rownames(T2D.f)],
                    normalized.lib.sizes = FALSE, prior.count = 2)
names(e4.cpm) <- rownames(cnts)

# Compute ave log cpm for the first cell group (grp2)
grp2.cpm <- aveLogCPM(cnts[, rownames(T2D.anns)],
                      normalized.lib.sizes = FALSE, prior.count = 2)
names(grp2.cpm) <- rownames(cnts)

# Compute ave log cpm for the second cell group (grp1)
grp1.cpm <- aveLogCPM(cnts[, rownames(nonT2D.anns)],
                      normalized.lib.sizes = FALSE, prior.count = 2)
names(grp1.cpm) <- rownames(cnts)

# Extract rownames of DE genes
rn <- rownames(res.ext)
e1.cpm <- e1.cpm[rn]
e2.cpm <- e2.cpm[rn]
e3.cpm <- e3.cpm[rn]
e4.cpm <- e4.cpm[rn]
```

```
  grp2.cpm <- grp2.cpm[rn]
  grp1.cpm <- grp1.cpm[rn]

  res.ext <- cbind(res.ext, e1.cpm, e2.cpm, e3.cpm, e4.cpm, grp2.cpm, grp1.cpm)

  # Combine average logCPM for each group into results table
  names(res.ext)[names(res.ext) == "e2.cpm"] <- paste("T2D Male", "avelogCPM", sep = " ")
  names(res.ext)[names(res.ext) == "e1.cpm"] <- paste("NonT2D Male", "avelogCPM", sep = " ")
  names(res.ext)[names(res.ext) == "e3.cpm"] <- paste("NonT2D Female", "avelogCPM", sep = " ")
  names(res.ext)[names(res.ext) == "e4.cpm"] <- paste("T2D Female", "avelogCPM", sep = " ")
  names(res.ext)[names(res.ext) == "grp2.cpm"] <- paste("T2D", "avelogCPM", sep = " ")
  names(res.ext)[names(res.ext) == "grp1.cpm"] <- paste("NonT2D", "avelogCPM", sep = " ")

  df <- res.ext[c("Description", "Associated.Gene.Name", "Gene.Biotype", "logFC",
                  "logCPM", "T2D Male avelogCPM", "NonT2D Male avelogCPM", "T2D Female avelogCPM",
                  "NonT2D Female avelogCPM", "T2D avelogCPM",
                  "NonT2D avelogCPM", "LR", "PValue", "FDR")]

  write.csv(df, file = paste(name, name1[i], "csv", sep = "."))

  # Filter by FDR of less than 0.05, no logFC filter
  df.filter <- df[df$FDR < 0.05,]
  write.csv(df.filter, file = paste(name, name1[i], "FDR.0.05.csv", sep="."))

}
```

Differential Expression Analyses of Type 2 Diabetic and Non-Diabetic Endocrine Ensemble Single Cells (Reconstituted Islet) using EdgeR

```
# load libraries
rm(list=ls())
suppressPackageStartupMessages(library(Biobase))
suppressPackageStartupMessages(library(edgeR))
suppressPackageStartupMessages(library(locfit))
library(edgeR)
library(Biobase)
library(locfit)
setwd("/Users/lawlon/Documents/Final_RNA_Seq_3/Data/")

# Specify cell type of interest
name = "EdgeR.Robust.T2D.vs.NonT2D"
type <- c("INS", "GCG", "SST", "PPY")
name1 = "Reconstituted.Islet"

# Load the ND data, get probe anns, sample anns
load("nonT2D.rdata")
probe.anns <- as(featureData(cnts.eset),"data.frame")
nonT2D.beta.eset <- cnts.eset[,cnts.eset$cell.type %in% c(type)]
nonT2D.anns <- pData(nonT2D.beta.eset)
# Male and female samples
nonT2D.m <- nonT2D.anns[nonT2D.anns$run %in% c("1st","2nd","3rd","4th","8th","6th","7th"),]
nonT2D.f <- nonT2D.anns[nonT2D.anns$run %in% c("5th","9th","10t","11t","12t","13t"),]
```

```r
# Separate cpm
ND.counts <- exprs(cnts.eset)

#Load the T2D data
load("T2D.rdata")
t2d.probe.anns <- as(featureData(cnts.eset),"data.frame")
T2D.beta.eset <- cnts.eset[,cnts.eset$cell.type %in% c(type)]
T2D.anns <- pData(T2D.beta.eset)
# Divide into male and female samples
T2D.m <- T2D.anns[T2D.anns$run %in% c("1st","2nd","3rd","4th","8th","6th","7th"),]
T2D.f <- T2D.anns[T2D.anns$run %in% c("5th","9th","10t","11t","12t","13t"),]

# Separate counts
T2D.counts <- exprs(cnts.eset)

# Combine sample anns for ND and T2D
sample.anns <- rbind(nonT2D.anns,T2D.anns)

# Combine counts for ND and T2D
cnts <- cbind(exprs(nonT2D.beta.eset),exprs(T2D.beta.eset))
# Make vector of phenotype info
# Runs 1,-3, 5-9 are ND, runs 10-13 and 4 are T2D
phenotype <- character(length = nrow(sample.anns))
phenotype[sample.anns$run %in% c("1st","2nd","3rd","5th","6th","7th","8th","9th")] <- "nonT2D"
phenotype[sample.anns$run %in% c("10t","11t","12t","13t","4th")] <- "T2D"

#Make vector of gender info
# Runs 5,9-13 are female, 1-4, 7-8 are male
gender <- character(length = nrow(sample.anns))
gender[sample.anns$run %in% c("5th","9th","10t","11t","12t","13t")] <- "F"
gender[sample.anns$run %in% c("1st","2nd","3rd","4th","8th","6th","7th")] <- "M"

# Info for generalized linear model
Status <- factor(phenotype)
Gender <- factor(gender)
# Design matrix has column of intercepts all 1's
  # Also columns for gender and phenotype indicating assignment of samples
design <- model.matrix(~Gender + Status)

# Apply cpm filter: counts > 20 in at least 20 percent of cells
T2D.cnts <- exprs(T2D.beta.eset)
ND.cnts <- exprs(nonT2D.beta.eset)
# Counts filtering for T2D
T2D.bin <- T2D.cnts
T2D.bin[T2D.cnts < 20] <- 0
T2D.bin[T2D.cnts >= 20] <- 1
T2D.exp <- apply(T2D.bin,1,sum)
T2D.th <- max(3,0.2*ncol(T2D.cnts))
T2D.sel <- T2D.cnts[T2D.exp > T2D.th,]

# Counts filtering for NonT2D
ND.bin <- ND.cnts
ND.bin[ND.cnts < 20] <- 0
```

```r
ND.bin[ND.cnts >= 20] <- 1
ND.exp <- apply(ND.bin,1,sum)
ND.th <- max(3,0.2*ncol(ND.cnts))
ND.sel <- ND.cnts[ND.exp > ND.th,]

# Find union of genes
un <- union(rownames(ND.sel), rownames(T2D.sel))
cnts.sel <- cnts[un,]

cds <- DGEList(counts = cnts.sel)
y <- calcNormFactors(cds)
y <- estimateDisp(y,design, robust = TRUE)
fit <- glmFit(y,design)
lrt <- glmLRT(fit,coef=3)
diff.dat <- topTags(lrt,n = nrow(cnts.sel))
res <- diff.dat$table

p.sel <- probe.anns[rownames(res),c("Description",
                                    "Associated.Gene.Name","Gene.Biotype")]
res.ext <- cbind(p.sel,res)

# Calculate avelogCPM for nonT2D male
e1.cpm <- aveLogCPM(cnts[,rownames(nonT2D.m)],
                    normalized.lib.sizes = FALSE, prior.count = 2)
# Assign names to vectors
names(e1.cpm) <- rownames(cnts)
# T2D males
if (dim(T2D.m)[1] == 0) {
  e2.cpm <- rep.int(0, times = dim(cnts)[1])
} else {
  e2.cpm <- aveLogCPM(cnts[,rownames(T2D.m)],
                      normalized.lib.sizes = FALSE, prior.count = 2)
  names(e2.cpm) <- rownames(cnts)
}
# nonT2D females
e3.cpm <- aveLogCPM(cnts[,rownames(nonT2D.f)],
                    normalized.lib.sizes = FALSE, prior.count = 2)
names(e3.cpm) <- rownames(cnts)
# T2D females
e4.cpm <- aveLogCPM(cnts[,rownames(T2D.f)],
                    normalized.lib.sizes = FALSE, prior.count = 2)
names(e4.cpm) <- rownames(cnts)

# Compute ave log cpm for the first cell group (grp2)
grp2.cpm <- aveLogCPM(cnts[, rownames(T2D.anns)],
                      normalized.lib.sizes = FALSE, prior.count = 2)
names(grp2.cpm) <- rownames(cnts)

# Compute ave log cpm for the second cell group (grp1)
grp1.cpm <- aveLogCPM(cnts[, rownames(nonT2D.anns)],
                      normalized.lib.sizes = FALSE, prior.count = 2)
names(grp1.cpm) <- rownames(cnts)
```

```r
# Extract rownames of DE genes
rn <- rownames(res.ext)
e1.cpm <- e1.cpm[rn]
e2.cpm <- e2.cpm[rn]
e3.cpm <- e3.cpm[rn]
e4.cpm <- e4.cpm[rn]
grp2.cpm <- grp2.cpm[rn]
grp1.cpm <- grp1.cpm[rn]

res.ext <- cbind(res.ext, e1.cpm, e2.cpm, e3.cpm, e4.cpm, grp2.cpm, grp1.cpm)

# Combine average logCPM for each group into results table
names(res.ext)[names(res.ext) == "e2.cpm"] <- paste("T2D Male", "avelogCPM", sep = " ")
names(res.ext)[names(res.ext) == "e1.cpm"] <- paste("NonT2D Male", "avelogCPM", sep = " ")
names(res.ext)[names(res.ext) == "e3.cpm"] <- paste("NonT2D Female", "avelogCPM", sep = " ")
names(res.ext)[names(res.ext) == "e4.cpm"] <- paste("T2D Female", "avelogCPM", sep = " ")
names(res.ext)[names(res.ext) == "grp2.cpm"] <- paste("T2D", "avelogCPM", sep = " ")
names(res.ext)[names(res.ext) == "grp1.cpm"] <- paste("NonT2D", "avelogCPM", sep = " ")

df <- res.ext[c("Description", "Associated.Gene.Name", "Gene.Biotype", "logFC",
             "logCPM", "T2D Male avelogCPM", "NonT2D Male avelogCPM", "T2D Female avelogCPM",
             "NonT2D Female avelogCPM", "T2D avelogCPM",
             "NonT2D avelogCPM", "LR", "PValue", "FDR")]

write.csv(df, file = paste(name, name1, "csv", sep = "."))

# Filter by FDR of less than 0.05, no logFC filter
df.filter <- df[df$FDR < 0.05,]
write.csv(df.filter, file = paste(name, name1, "FDR.0.05.csv", sep="."))
```

Session Information

```r
# Load libraries
suppressPackageStartupMessages(library(Biobase))
suppressPackageStartupMessages(library(edgeR))
```

```
## Warning: package 'limma' was built under R version 3.3.1
```

```r
suppressPackageStartupMessages(library(locfit))
library(Biobase)
library(edgeR)
library(locfit)
library(RColorBrewer)

sessionInfo()
```

```
## R version 3.3.0 (2016-05-03)
## Platform: x86_64-apple-darwin13.4.0 (64-bit)
## Running under: OS X 10.11.6 (El Capitan)
##
## locale:
## [1] en_US.UTF-8/en_US.UTF-8/en_US.UTF-8/C/en_US.UTF-8/en_US.UTF-8
##
```

```
## attached base packages:
## [1] parallel  stats     graphics  grDevices utils     datasets  methods
## [8] base
##
## other attached packages:
## [1] RColorBrewer_1.1-2  locfit_1.5-9.1      edgeR_3.14.0
## [4] limma_3.28.21       Biobase_2.32.0      BiocGenerics_0.18.0
##
## loaded via a namespace (and not attached):
##  [1] Rcpp_0.12.7     lattice_0.20-34 digest_0.6.10   assertthat_0.1
##  [5] grid_3.3.0      formatR_1.4     magrittr_1.5    evaluate_0.10
##  [9] stringi_1.1.2   rmarkdown_1.1   tools_3.3.0     stringr_1.1.0
## [13] yaml_2.1.13     htmltools_0.3.5 knitr_1.14      tibble_1.2
```
