# Supplementary material for: Single-cell transcriptomes identify human islet cell signatures and reveal cell-type–specific expression changes in type 2 diabetes
Source: Genome Res. 2017 Feb;27(2):208–22. doi: 10.1101/gr.212720.116 (PMC5287227; doi:10.1101/gr.212720.116)
Supplement: Supplemental Material [file supp_gr.212720.116_Supplemental_Methods_Source_Code.zip › Supplemental_Methods_Source_Code/Supplemental_Table_Source_Code/Supplemental_Table_S7_Source_Code.pdf]

Identification of Cell Type Signature Genes

Introduction

Endocrine cell signature genes were identified by first performing the differential expression analysis procedure as described in "Differential.Expression.Rmd" between each endocrine cell type (e.g. Beta vs Alpha, Beta vs Delta, and Beta vs Gamma) in our non-diabetic data. For each pairwise comparison, a list of differentially expressed genes was produced. Afterwards, the intersection of these pairwise differential gene lists was performed to identify genes that were exclusively differentially expressed in the cell type. Intersected genes with a positive log fold change were classified as being uniquely expressed in the cell type (signature genes). The intersected genes with a negative log fold change were classified as being uniquely absent or forbidden in the cell type. Exocrine cell signature genes were identified in a similar manner. For example, signature genes for the Stellate cells were defined by performing pairwise differential expression analyses of Stellate vs Acinar and Stellate vs Ductal cells. Following this, the intersection of these two differential gene lists was performed to yield signature genes for the Stellate cells as shown in the example below.

Endocrine Cell Type Signature Genes

```
# Script to find intersection of differentially expressed genes. The final output
  # is a spreadsheet of signature genes with the log2 fold change and log2 counts
  # per million information for each cell type comparison.

# Load in cell type differential expression lists for beta cells
setwd("/Users/lawlon/Documents/Final_RNA_Seq_3/Differential_Expression_3/Single_Cell/NonT2D_Cell_Type_Co
# Indicate comparison groups (grp1 vs grp2)
cell = "Beta"
name = "NonT2D.SC.Positive.FC.intersect"

grp1 = paste(cell, "-Alpha", sep ="")
grp2 = paste(cell, "-Delta", sep ="")
grp3 = paste(cell, "-Gamma", sep="")

# Load gene lists
d1 <- read.csv(file="EdgeR.Robust.NonT2D.Beta.vs.Alpha.FDR.0.05.csv", header=TRUE,
               row.names=1, check.names = FALSE)
d2 <- read.csv(file="EdgeR.Robust.NonT2D.Beta.vs.Delta.FDR.0.05.csv", header=TRUE,
               row.names = 1, check.names = FALSE)
d3 <- read.csv(file="EdgeR.Robust.NonT2D.Beta.vs.Gamma.FDR.0.05.csv", header=TRUE,
               row.names = 1, check.names = FALSE)

# Find similar genes
inter1 <- intersect(rownames(d1), rownames(d2))
inter2 <- intersect(rownames(d3), inter1)

# Obtain selected genes for each group
d1.sel <- d1[inter2,]
d2.sel <- d2[inter2,]
d3.sel <- d3[inter2,]

# Re-name columns to specify which group is which
```

```
names(d1.sel)[names(d1.sel) == "logFC"] <- paste(grp1, "logFC", sep = " ")
names(d1.sel)[names(d1.sel) == "logCPM"] <- paste(grp1, "logCPM", sep = " ")

names(d2.sel)[names(d2.sel) == "logFC"] <- paste(grp2, "logFC", sep = " ")
names(d2.sel)[names(d2.sel) == "logCPM"] <- paste(grp2, "logCPM", sep = " ")

names(d3.sel)[names(d3.sel) == "logFC"] <- paste(grp3, "logFC", sep = " ")
names(d3.sel)[names(d3.sel) == "logCPM"] <- paste(grp3, "logCPM", sep = " ")

# Combine tables
# Gene info and first group logFC
res <- d1.sel[,1:4]
# Second group logFC
res <- cbind(res, d2.sel[,4])
names(res)[names(res) == "d2.sel[, 4]"] <- paste(grp2, "logFC", sep = " ")
res <- cbind(res, d3.sel[,4])
names(res)[names(res) == "d3.sel[, 4]"] <- paste(grp3, "logFC", sep = " ")

# first group logCPM
res <- cbind(res, d1.sel[,5])
names(res)[names(res) == "d1.sel[, 5]"] <- paste(grp1, "logCPM", sep = " ")
# second group logCPM
res <- cbind(res, d2.sel[,5])
names(res)[names(res) == "d2.sel[, 5]"] <- paste(grp2, "logCPM", sep = " ")
res <- cbind(res, d3.sel[,5])
names(res)[names(res) == "d3.sel[, 5]"] <- paste(grp3, "logCPM", sep = " ")

# Get genes with positive logFC
res.up.idx <- which(res[,4] > 0 & res[,5] > 0 & res[,6] > 0)
res.up <- res[res.up.idx,]

# write table to file
write.csv(res.up, file = paste(name, cell, "csv", sep = "."))


# Load in cell type differential expression lists for alpha cells
setwd("/Users/lawlon/Documents/Final_RNA_Seq_3/Differential_Expression_3/Single_Cell/NonT2D_Cell_Type_C
# Indicate comparison groups (grp1 vs grp2)
cell = "Alpha"
name = "NonT2D.SC.Positive.FC.intersect"

grp1 = paste(cell, "-Beta", sep ="")
grp2 = paste(cell, "-Delta", sep ="")
grp3 = paste(cell, "-Gamma", sep="")

# Load gene lists
d1 <- read.csv(file="EdgeR.Robust.NonT2D.Alpha.vs.Beta.FDR.0.05.csv", header=TRUE,
               row.names=1, check.names = FALSE)
d2 <- read.csv(file="EdgeR.Robust.NonT2D.Alpha.vs.Delta.FDR.0.05.csv", header=TRUE,
               row.names = 1, check.names = FALSE)
d3 <- read.csv(file="EdgeR.Robust.NonT2D.Alpha.vs.Gamma.FDR.0.05.csv", header=TRUE,
               row.names = 1, check.names = FALSE)
```

```
# Find similar genes
inter1 <- intersect(rownames(d1), rownames(d2))
inter2 <- intersect(rownames(d3), inter1)

# Obtain selected genes for each group
d1.sel <- d1[inter2,]
d2.sel <- d2[inter2,]
d3.sel <- d3[inter2,]

# Re-name columns to specify which group is which
names(d1.sel)[names(d1.sel) == "logFC"] <- paste(grp1, "logFC", sep = " ")
names(d1.sel)[names(d1.sel) == "logCPM"] <- paste(grp1, "logCPM", sep = " ")

names(d2.sel)[names(d2.sel) == "logFC"] <- paste(grp2, "logFC", sep = " ")
names(d2.sel)[names(d2.sel) == "logCPM"] <- paste(grp2, "logCPM", sep = " ")

names(d3.sel)[names(d3.sel) == "logFC"] <- paste(grp3, "logFC", sep = " ")
names(d3.sel)[names(d3.sel) == "logCPM"] <- paste(grp3, "logCPM", sep = " ")

# Combine tables
# Gene info and first group logFC
res <- d1.sel[,1:4]
# Second group logFC
res <- cbind(res, d2.sel[,4])
names(res)[names(res) == "d2.sel[, 4]"] <- paste(grp2, "logFC", sep = " ")
res <- cbind(res, d3.sel[,4])
names(res)[names(res) == "d3.sel[, 4]"] <- paste(grp3, "logFC", sep = " ")

# first group logCPM
res <- cbind(res, d1.sel[,5])
names(res)[names(res) == "d1.sel[, 5]"] <- paste(grp1, "logCPM", sep = " ")
# second group logCPM
res <- cbind(res, d2.sel[,5])
names(res)[names(res) == "d2.sel[, 5]"] <- paste(grp2, "logCPM", sep = " ")
res <- cbind(res, d3.sel[,5])
names(res)[names(res) == "d3.sel[, 5]"] <- paste(grp3, "logCPM", sep = " ")

# Get genes with positive logFC
res.up.idx <- which(res[,4] > 0 & res[,5] > 0 & res[,6] > 0)
res.up <- res[res.up.idx,]

# write table to file
write.csv(res.up, file = paste(name, cell, "csv", sep = "."))


# Load in cell type differential expression lists for delta cells
setwd("/Users/lawlon/Documents/Final_RNA_Seq_3/Differential_Expression_3/Single_Cell/NonT2D_Cell_Type_C
# Indicate comparison groups (grp1 vs grp2)
cell = "Delta"
name = "NonT2D.SC.Positive.FC.intersect"

grp1 = paste(cell, "-Beta", sep ="")
grp2 = paste(cell, "-Alpha", sep ="")
```

```r
grp3 = paste(cell, "-Gamma", sep="")

# Load gene lists
d1 <- read.csv(file="EdgeR.Robust.NonT2D.Delta.vs.Beta.FDR.0.05.csv", header=TRUE,
               row.names=1, check.names = FALSE)
d2 <- read.csv(file="EdgeR.Robust.NonT2D.Delta.vs.Alpha.FDR.0.05.csv", header=TRUE,
               row.names = 1, check.names = FALSE)
d3 <- read.csv(file="EdgeR.Robust.NonT2D.Delta.vs.Gamma.FDR.0.05.csv", header=TRUE,
               row.names = 1, check.names = FALSE)

# Find similar genes
inter1 <- intersect(rownames(d1), rownames(d2))
inter2 <- intersect(rownames(d3), inter1)

# Obtain selected genes for each group
d1.sel <- d1[inter2,]
d2.sel <- d2[inter2,]
d3.sel <- d3[inter2,]

# Re-name columns to specify which group is which
names(d1.sel)[names(d1.sel) == "logFC"] <- paste(grp1, "logFC", sep = " ")
names(d1.sel)[names(d1.sel) == "logCPM"] <- paste(grp1, "logCPM", sep = " ")

names(d2.sel)[names(d2.sel) == "logFC"] <- paste(grp2, "logFC", sep = " ")
names(d2.sel)[names(d2.sel) == "logCPM"] <- paste(grp2, "logCPM", sep = " ")

names(d3.sel)[names(d3.sel) == "logFC"] <- paste(grp3, "logFC", sep = " ")
names(d3.sel)[names(d3.sel) == "logCPM"] <- paste(grp3, "logCPM", sep = " ")

# Combine tables
# Gene info and first group logFC
res <- d1.sel[,1:4]
# Second group logFC
res <- cbind(res, d2.sel[,4])
names(res)[names(res) == "d2.sel[, 4]"] <- paste(grp2, "logFC", sep = " ")
res <- cbind(res, d3.sel[,4])
names(res)[names(res) == "d3.sel[, 4]"] <- paste(grp3, "logFC", sep = " ")

# first group logCPM
res <- cbind(res, d1.sel[,5])
names(res)[names(res) == "d1.sel[, 5]"] <- paste(grp1, "logCPM", sep = " ")
# second group logCPM
res <- cbind(res, d2.sel[,5])
names(res)[names(res) == "d2.sel[, 5]"] <- paste(grp2, "logCPM", sep = " ")
res <- cbind(res, d3.sel[,5])
names(res)[names(res) == "d3.sel[, 5]"] <- paste(grp3, "logCPM", sep = " ")

# Get genes with positive logFC
res.up.idx <- which(res[,4] > 0 & res[,5] > 0 & res[,6] > 0)
res.up <- res[res.up.idx,]

# write table to file
write.csv(res.up, file = paste(name, cell, "csv", sep = "."))
```

```
# Load in cell type differential expression lists for gamma cells
setwd("/Users/lawlon/Documents/Final_RNA_Seq_3/Differential_Expression_3/Single_Cell/NonT2D_Cell_Type_C
# Indicate comparison groups (grp1 vs grp2)
cell = "Gamma"
name = "NonT2D.SC.Positive.FC.intersect"

grp1 = paste(cell, "-Beta", sep ="")
grp2 = paste(cell, "-Alpha", sep ="")
grp3 = paste(cell, "-Delta", sep="")

# Load gene lists
d1 <- read.csv(file="EdgeR.Robust.NonT2D.Gamma.vs.Beta.FDR.0.05.csv", header=TRUE,
               row.names=1, check.names = FALSE)
d2 <- read.csv(file="EdgeR.Robust.NonT2D.Gamma.vs.Alpha.FDR.0.05.csv", header=TRUE,
               row.names = 1, check.names = FALSE)
d3 <- read.csv(file="EdgeR.Robust.NonT2D.Gamma.vs.Delta.FDR.0.05.csv", header=TRUE,
               row.names = 1, check.names = FALSE)

# Find similar genes
inter1 <- intersect(rownames(d1), rownames(d2))
inter2 <- intersect(rownames(d3), inter1)

# Obtain selected genes for each group
d1.sel <- d1[inter2,]
d2.sel <- d2[inter2,]
d3.sel <- d3[inter2,]

# Re-name columns to specify which group is which
names(d1.sel)[names(d1.sel) == "logFC"] <- paste(grp1, "logFC", sep = " ")
names(d1.sel)[names(d1.sel) == "logCPM"] <- paste(grp1, "logCPM", sep = " ")

names(d2.sel)[names(d2.sel) == "logFC"] <- paste(grp2, "logFC", sep = " ")
names(d2.sel)[names(d2.sel) == "logCPM"] <- paste(grp2, "logCPM", sep = " ")

names(d3.sel)[names(d3.sel) == "logFC"] <- paste(grp3, "logFC", sep = " ")
names(d3.sel)[names(d3.sel) == "logCPM"] <- paste(grp3, "logCPM", sep = " ")

# Combine tables
# Gene info and first group logFC
res <- d1.sel[,1:4]
# Second group logFC
res <- cbind(res, d2.sel[,4])
names(res)[names(res) == "d2.sel[, 4]"] <- paste(grp2, "logFC", sep = " ")
res <- cbind(res, d3.sel[,4])
names(res)[names(res) == "d3.sel[, 4]"] <- paste(grp3, "logFC", sep = " ")

# first group logCPM
res <- cbind(res, d1.sel[,5])
names(res)[names(res) == "d1.sel[, 5]"] <- paste(grp1, "logCPM", sep = " ")
# second group logCPM
res <- cbind(res, d2.sel[,5])
names(res)[names(res) == "d2.sel[, 5]"] <- paste(grp2, "logCPM", sep = " ")
res <- cbind(res, d3.sel[,5])
```

```
names(res)[names(res) == "d3.sel[, 5]"] <- paste(grp3, "logCPM", sep = " ")

# Get genes with positive logFC
res.up.idx <- which(res[,4] > 0 & res[,5] > 0 & res[,6] > 0)
res.up <- res[res.up.idx,]

# write table to file
write.csv(res.up, file = paste(name, cell, "csv", sep = "."))
```

Exocrine Cell Type Signature Genes

```
# Identify signature genes for Acinar cells
setwd("/Users/lawlon/Documents/Final_RNA_Seq_3/Differential_Expression_3/Single_Cell/NonT2D_Cell_Type_Co
# Indicate comparison groups (grp1 vs grp2)
cell = "Acinar"
name = "NonT2D.SC.Positive.FC.intersect"

grp1 = paste(cell, "-Ductal", sep ="")
grp2 = paste(cell, "-Stellate", sep ="")

# Load gene lists
d1 <- read.csv(file="EdgeR.Robust.NonT2D.Acinar.vs.Ductal.FDR.0.05.csv", header=TRUE,
               row.names=1, check.names = FALSE)
d2 <- read.csv(file="EdgeR.Robust.NonT2D.Acinar.vs.Stellate.FDR.0.05.csv", header=TRUE,
               row.names = 1, check.names = FALSE)

# Find similar genes
inter1 <- intersect(rownames(d1), rownames(d2))
d1.sel <- d1[inter1,]
d2.sel <- d2[inter1,]

# Re-name columns to specify which group is which
names(d1.sel)[names(d1.sel) == "logFC"] <- paste(grp1, "logFC", sep = " ")
names(d1.sel)[names(d1.sel) == "logCPM"] <- paste(grp1, "logCPM", sep = " ")

names(d2.sel)[names(d2.sel) == "logFC"] <- paste(grp2, "logFC", sep = " ")
names(d2.sel)[names(d2.sel) == "logCPM"] <- paste(grp2, "logCPM", sep = " ")

# Combine tables
# Gene info and first group logFC
res <- d1.sel[,1:4]
# Second group logFC
res <- cbind(res, d2.sel[,4])
names(res)[names(res) == "d2.sel[, 4]"] <- paste(grp2, "logFC", sep = " ")
#res <- cbind(res, d3.sel[,4])
#names(res)[names(res) == "d3.sel[, 4]"] <- paste(grp3, "logFC", sep = " ")

# first group logCPM
res <- cbind(res, d1.sel[,5])
names(res)[names(res) == "d1.sel[, 5]"] <- paste(grp1, "logCPM", sep = " ")
# second group logCPM
```

```
res <- cbind(res, d2.sel[,5])
names(res)[names(res) == "d2.sel[, 5]"] <- paste(grp2, "logCPM", sep = " ")

# Get genes with positive logFC
res.up.idx <- which(res[,4] > 0 & res[,5] > 0)
res.up <- res[res.up.idx,]

# write table to file
write.csv(res.up, file = paste(name, cell, "csv", sep = "."))


# Identify signature genes for Stellate cells
setwd("/Users/lawlon/Documents/Final_RNA_Seq_3/Differential_Expression_3/Single_Cell/NonT2D_Cell_Type_C
# Indicate comparison groups (grp1 vs grp2)
cell = "Stellate"
name = "NonT2D.SC.Positive.FC.intersect"

grp1 = paste(cell, "-Ductal", sep ="")
grp2 = paste(cell, "-Acinar", sep ="")

# Load gene lists
d1 <- read.csv(file="EdgeR.Robust.NonT2D.Stellate.vs.Ductal.FDR.0.05.csv", header=TRUE,
               row.names=1, check.names = FALSE)
d2 <- read.csv(file="EdgeR.Robust.NonT2D.Stellate.vs.Acinar.FDR.0.05.csv", header=TRUE,
               row.names = 1, check.names = FALSE)

# Find similar genes
inter1 <- intersect(rownames(d1), rownames(d2))
d1.sel <- d1[inter1,]
d2.sel <- d2[inter1,]

# Re-name columns to specify which group is which
names(d1.sel)[names(d1.sel) == "logFC"] <- paste(grp1, "logFC", sep = " ")
names(d1.sel)[names(d1.sel) == "logCPM"] <- paste(grp1, "logCPM", sep = " ")

names(d2.sel)[names(d2.sel) == "logFC"] <- paste(grp2, "logFC", sep = " ")
names(d2.sel)[names(d2.sel) == "logCPM"] <- paste(grp2, "logCPM", sep = " ")

# Combine tables
# Gene info and first group logFC
res <- d1.sel[,1:4]
# Second group logFC
res <- cbind(res, d2.sel[,4])
names(res)[names(res) == "d2.sel[, 4]"] <- paste(grp2, "logFC", sep = " ")
#res <- cbind(res, d3.sel[,4])
#names(res)[names(res) == "d3.sel[, 4]"] <- paste(grp3, "logFC", sep = " ")

# first group logCPM
res <- cbind(res, d1.sel[,5])
names(res)[names(res) == "d1.sel[, 5]"] <- paste(grp1, "logCPM", sep = " ")
# second group logCPM
res <- cbind(res, d2.sel[,5])
names(res)[names(res) == "d2.sel[, 5]"] <- paste(grp2, "logCPM", sep = " ")
```

```
# Get genes with positive logFC
res.up.idx <- which(res[,4] > 0 & res[,5] > 0)
res.up <- res[res.up.idx,]

# write table to file
write.csv(res.up, file = paste(name, cell, "csv", sep = "."))


# Identify signature genes for Ductal cells
setwd("/Users/lawlon/Documents/Final_RNA_Seq_3/Differential_Expression_3/Single_Cell/NonT2D_Cell_Type_C
# Indicate comparison groups (grp1 vs grp2)
cell = "Ductal"
name = "NonT2D.SC.Positive.FC.intersect"

grp1 = paste(cell, "-Acinar", sep ="")
grp2 = paste(cell, "-Stellate", sep ="")

# Load gene lists
d1 <- read.csv(file="EdgeR.Robust.NonT2D.Ductal.vs.Acinar.FDR.0.05.csv", header=TRUE,
               row.names=1, check.names = FALSE)
d2 <- read.csv(file="EdgeR.Robust.NonT2D.Ductal.vs.Stellate.FDR.0.05.csv", header=TRUE,
               row.names = 1, check.names = FALSE)

# Find similar genes
inter1 <- intersect(rownames(d1), rownames(d2))
d1.sel <- d1[inter1,]
d2.sel <- d2[inter1,]

# Re-name columns to specify which group is which
names(d1.sel)[names(d1.sel) == "logFC"] <- paste(grp1, "logFC", sep = " ")
names(d1.sel)[names(d1.sel) == "logCPM"] <- paste(grp1, "logCPM", sep = " ")

names(d2.sel)[names(d2.sel) == "logFC"] <- paste(grp2, "logFC", sep = " ")
names(d2.sel)[names(d2.sel) == "logCPM"] <- paste(grp2, "logCPM", sep = " ")

# Combine tables
# Gene info and first group logFC
res <- d1.sel[,1:4]
# Second group logFC
res <- cbind(res, d2.sel[,4])
names(res)[names(res) == "d2.sel[, 4]"] <- paste(grp2, "logFC", sep = " ")
#res <- cbind(res, d3.sel[,4])
#names(res)[names(res) == "d3.sel[, 4]"] <- paste(grp3, "logFC", sep = " ")

# first group logCPM
res <- cbind(res, d1.sel[,5])
names(res)[names(res) == "d1.sel[, 5]"] <- paste(grp1, "logCPM", sep = " ")
# second group logCPM
res <- cbind(res, d2.sel[,5])
names(res)[names(res) == "d2.sel[, 5]"] <- paste(grp2, "logCPM", sep = " ")

# Get genes with positive logFC
res.up.idx <- which(res[,4] > 0 & res[,5] > 0)
```

```r
res.up <- res[res.up.idx,]

# write table to file
write.csv(res.up, file = paste(name, cell, "csv", sep = "."))
```

Session Information

```r
sessionInfo()
```

```
## R version 3.3.0 (2016-05-03)
## Platform: x86_64-apple-darwin13.4.0 (64-bit)
## Running under: OS X 10.11.6 (El Capitan)
##
## locale:
## [1] en_US.UTF-8/en_US.UTF-8/en_US.UTF-8/C/en_US.UTF-8/en_US.UTF-8
##
## attached base packages:
## [1] stats     graphics  grDevices utils     datasets  methods   base
##
## loaded via a namespace (and not attached):
##  [1] magrittr_1.5    assertthat_0.1  formatR_1.4     tools_3.3.0
##  [5] htmltools_0.3.5 yaml_2.1.13     tibble_1.2      Rcpp_0.12.7
##  [9] stringi_1.1.2   rmarkdown_1.1   knitr_1.14      stringr_1.1.0
## [13] digest_0.6.10   evaluate_0.10
```
