# Supplementary material for: Single-cell transcriptomes identify human islet cell signatures and reveal cell-type–specific expression changes in type 2 diabetes
Source: Genome Res. 2017 Feb;27(2):208–22. doi: 10.1101/gr.212720.116 (PMC5287227; doi:10.1101/gr.212720.116)
Supplement: Supplemental Material [file supp_gr.212720.116_Supplemental_Methods_Source_Code.zip › Supplemental_Methods_Source_Code/Supplemental_Table_Source_Code/Supplemental_Table_S8_Source_Code.pdf]

Gene Set Analysis of NonT2D Islet Cell Types using GAGE

Introduction

Gene set analysis (GSA) is a powerful strategy to infer functional and mechanistic changes from high through microarray or sequencing data. GSA focuses on sets of related genes and has established major advantages over per-gene based different expression analyses, including greater robustness, sensitivity and biological relevance. This report will detail the analyses performed to identify Kyoto Encyclopedia of Genes and Genomes (KEGG) pathways enriched in each of the islet cell types using the method Generally Applicable Gene-set Enrichment (GAGE). Specifically, we used the R-package "gage" to conduct our analyses. To determine enriched pathways for each islet cell type we compared each cell type against the other collective islet cell types (e.g. Beta vs Alpha, Delta, and Gamma cells). Pathways with an FDR adjusted p-value (q.val) < 0.05 were considered to be significantly enriched.

Reference: Luo, W., Friedman, M., Shedden K., Hankenson, K. and Woolf, P GAGE: Generally Applicable Gene Set Enrichment for Pathways Analysis. BMC Bioinformatics, 2009, 10:161

Gene Set Analysis of Beta Cells

```r
# Load in libraries
suppressPackageStartupMessages(library(Biobase))
suppressPackageStartupMessages(library(org.Hs.eg.db))
suppressPackageStartupMessages(library(gage))
suppressPackageStartupMessages(library(gageData))
library(Biobase)
library(org.Hs.eg.db)
library(gage)
library(gageData)
rm(list=ls())
setwd("/Users/lawlon/Documents/Final_RNA_Seq_3/Data/")
# Declare cell type of interest and name of comparison
name <- "NonT2D"
cell1 = c("INS")
all.grps = c("INS", "GCG", "SST", "PPY")
# cell2 is all other islet cell types to be compared against cell1
cell2 <- all.grps[cell1!= all.grps]
name1 = "Beta"
name2 = "Endocrine"
# Load NonT2D data
load("nonT2D.rdata")
# probe annotations and sample annotations
p.anns <- featureData(cnts.eset)
probe.anns <- as(p.anns,"data.frame")
s.anns1 <- pData(cnts.eset)
exp1 <- exprs(cnts.eset)
cell.anns1 <- s.anns1[s.anns1$cell.type %in% cell1,]
# Extract expression data of interest for cell1
exp1.sel <- exp1[, rownames(cell.anns1)]
cell.anns2 <- s.anns1[s.anns1$cell.type %in% cell2,]
```

```r
exp2.sel <- exp1[,rownames(cell.anns2)]
# Combine expression data in single matrix
exp <- cbind(exp1.sel, exp2.sel)
# Function to truncate the sample names
# Result is run number and sample: 12th-C7
grp <- sapply(colnames(exp),function(dat) { strsplit(as.character(dat),"_")[[1]][1]})
group <- as.character(grp)
# Determine which genes have zero counts across all samples
sel.rn=rowSums(exp) != 0
# Extract only those genes with more than zero counts across samples
cnts=exp[sel.rn,]
# Library sizes = sum of column (sample) counts
libsizes=colSums(cnts)
# Normalize the library sizes
size.factor=libsizes/exp(mean(log(libsizes)))
# Normalize the counts and log transform
cnts.norm=t(t(cnts)/size.factor)
cnts.norm=log2(cnts.norm+8)

# Get entrez ID's from ensembl ids
entid <- mget(rownames(cnts.norm),org.Hs.egENSEMBL2EG,ifnotfound=NA)
# Make empty list
entrezid <- character(length = nrow(cnts.norm))
# Add NA's to empty list
entrezid[] <- NA
# Loop to only extract the entez ID's that are not NA
for(i in 1:length(entrezid))
{
  if(!is.na(entid[i]))
  {
    entrezid[i] <- entid[[i]][1]
  }
}

# Find out which entrezid id's are NA's
idx <- is.na(entrezid)
# Extract the genes with entrez id's
cnts.norm.sel <- cnts.norm[!idx,]
# Add entrezid names to the end of the probe anns table
probe.anns.sel <- data.frame(probe.anns[rownames(cnts.norm.sel),],
                             entrezid = entrezid[!idx],stringsAsFactors=FALSE)
# Change ensembl id's to entrez id's in counts matrix
rownames(cnts.norm.sel) <- entrezid[!idx]

# Load pathway gene sets
data("go.sets.hs")
data("kegg.sets.hs")
# Declare which samples are of interest
samp.idx <- 1:dim(exp1.sel)[2]
# Declare background samples
ref.idx <- (dim(exp1.sel)[2]+1):dim(cnts.norm.sel)[2]
# Pathway analysis
cnts.kegg.p <- gage(cnts.norm.sel, gsets = kegg.sets.hs,
```

```
                    ref = ref.idx, samp = samp.idx, compare ="unpaired")
cnts.d= cnts.norm.sel[, samp.idx]-rowMeans(cnts.norm.sel[, ref.idx])
# Combine probe annotation and normalized count data
diff.dat <- data.frame(probe.anns.sel, cnts.d)
# Select for upregulated kegg pathways that are significant and not NA
sel.up <- cnts.kegg.p$greater[,1:5]
# Select for less (downregulated) pathways
sel.down <- cnts.kegg.p$less[, 1:5]
# Write pathways to file
write.csv(sel.up, file=paste(name, name1, "vs", name2,"KEGG.upregulated.csv", sep = "."))
write.csv(sel.down, file=paste(name, name1, "vs", name2,"KEGG.downregulated.csv", sep = "."))
```

Gene Set Analysis of Alpha Cells

```
# Load in libraries
suppressPackageStartupMessages(library(Biobase))
suppressPackageStartupMessages(library(org.Hs.eg.db))
suppressPackageStartupMessages(library(gage))
suppressPackageStartupMessages(library(gageData))
library(Biobase)
library(org.Hs.eg.db)
library(gage)
library(gageData)
rm(list=ls())
setwd("/Users/lawlon/Documents/Final_RNA_Seq_3/Data/")
# Declare cell type of interest and name of comparison
name <- "NonT2D"
cell1 = c("GCG")
all.grps = c("INS", "GCG", "SST", "PPY")
# cell2 is all other islet cell types to be compared against cell1
cell2 <- all.grps[cell1!= all.grps]
name1 = "Alpha"
name2 = "Endocrine"
# Load NonT2D data
load("nonT2D.rdata")
# probe annotations and sample annotations
p.anns <- featureData(cnts.eset)
probe.anns <- as(p.anns,"data.frame")
s.anns1 <- pData(cnts.eset)
exp1 <- exprs(cnts.eset)
cell.anns1 <- s.anns1[s.anns1$cell.type %in% cell1,]
# Extract expression data of interest for cell1
exp1.sel <- exp1[, rownames(cell.anns1)]
cell.anns2 <- s.anns1[s.anns1$cell.type %in% cell2,]
exp2.sel <- exp1[,rownames(cell.anns2)]
# Combine expression data in single matrix
exp <- cbind(exp1.sel, exp2.sel)
# Function to truncate the sample names
# Result is run number and sample: 12th-C7
grp <- sapply(colnames(exp),function(dat) { strsplit(as.character(dat),"_")[[1]][1]})
group <- as.character(grp)
# Determine which genes have zero counts across all samples
```

```r
sel.rn=rowSums(exp) != 0
# Extract only those genes with more than zero counts across samples
cnts=exp[sel.rn,]
# Library sizes = sum of column (sample) counts
libsizes=colSums(cnts)
# Normalize the library sizes
size.factor=libsizes/exp(mean(log(libsizes)))
# Normalize the counts and log transform
cnts.norm=t(t(cnts)/size.factor)
cnts.norm=log2(cnts.norm+8)

# Get entrez ID's from ensembl ids
entid <- mget(rownames(cnts.norm),org.Hs.egENSEMBL2EG,ifnotfound=NA)
# Make empty list
entrezid <- character(length = nrow(cnts.norm))
# Add NA's to empty list
entrezid[] <- NA
# Loop to only extract the entez ID's that are not NA
for(i in 1:length(entrezid))
{
  if(!is.na(entid[i]))
  {
    entrezid[i] <- entid[[i]][1]
  }
}

# Find out which entrezid id's are NA's
idx <- is.na(entrezid)
# Extract the genes with entrez id's
cnts.norm.sel <- cnts.norm[!idx,]
# Add entrezid names to the end of the probe anns table
probe.anns.sel <- data.frame(probe.anns[rownames(cnts.norm.sel),],
                             entrezid = entrezid[!idx],stringsAsFactors=FALSE)
# Change ensembl id's to entrez id's in counts matrix
rownames(cnts.norm.sel) <- entrezid[!idx]

# Load pathway gene sets
data("go.sets.hs")
data("kegg.sets.hs")
# Declare which samples are of interest
samp.idx <- 1:dim(exp1.sel)[2]
# Declare background samples
ref.idx <- (dim(exp1.sel)[2]+1):dim(cnts.norm.sel)[2]
# Pathway analysis
cnts.kegg.p <- gage(cnts.norm.sel, gsets = kegg.sets.hs,
                    ref = ref.idx, samp = samp.idx, compare ="unpaired")
cnts.d= cnts.norm.sel[, samp.idx]-rowMeans(cnts.norm.sel[, ref.idx])
# Combine probe annotation and normalized count data
diff.dat <- data.frame(probe.anns.sel, cnts.d)
# Select for upregulated kegg pathways that are significant and not NA
sel.up <- cnts.kegg.p$greater[,1:5]
# Select for less (downregulated) pathways
sel.down <- cnts.kegg.p$less[, 1:5]
```

```
# Write pathways to file
write.csv(sel.up, file=paste(name, name1, "vs", name2,"KEGG.upregulated.csv", sep = "."))
write.csv(sel.down, file=paste(name, name1, "vs", name2,"KEGG.downregulated.csv", sep = "."))
```

Gene Set Analysis of Delta Cells

```
# Load in libraries
suppressPackageStartupMessages(library(Biobase))
suppressPackageStartupMessages(library(org.Hs.eg.db))
suppressPackageStartupMessages(library(gage))
suppressPackageStartupMessages(library(gageData))
library(Biobase)
library(org.Hs.eg.db)
library(gage)
library(gageData)
rm(list=ls())
setwd("/Users/lawlon/Documents/Final_RNA_Seq_3/Data/")
# Declare cell type of interest and name of comparison
name <- "NonT2D"
cell1 = c("SST")
all.grps = c("INS", "GCG", "SST", "PPY")
# cell2 is all other islet cell types to be compared against cell1
cell2 <- all.grps[cell1!= all.grps]
name1 = "Delta"
name2 = "Endocrine"
# Load NonT2D data
load("nonT2D.rdata")
# probe annotations and sample annotations
p.anns <- featureData(cnts.eset)
probe.anns <- as(p.anns,"data.frame")
s.anns1 <- pData(cnts.eset)
exp1 <- exprs(cnts.eset)
cell.anns1 <- s.anns1[s.anns1$cell.type %in% cell1,]
# Extract expression data of interest for cell1
exp1.sel <- exp1[, rownames(cell.anns1)]
cell.anns2 <- s.anns1[s.anns1$cell.type %in% cell2,]
exp2.sel <- exp1[,rownames(cell.anns2)]
# Combine expression data in single matrix
exp <- cbind(exp1.sel, exp2.sel)
# Function to truncate the sample names
# Result is run number and sample: 12th-C7
grp <- sapply(colnames(exp),function(dat) { strsplit(as.character(dat),"_")[[1]][1]})
group <- as.character(grp)
# Determine which genes have zero counts across all samples
sel.rn=rowSums(exp) != 0
# Extract only those genes with more than zero counts across samples
cnts=exp[sel.rn,]
# Library sizes = sum of column (sample) counts
libsizes=colSums(cnts)
# Normalize the library sizes
size.factor=libsizes/exp(mean(log(libsizes)))
# Normalize the counts and log transform
```

```r
cnts.norm=t(t(cnts)/size.factor)
cnts.norm=log2(cnts.norm+8)

# Get entrez ID's from ensembl ids
entid <- mget(rownames(cnts.norm),org.Hs.egENSEMBL2EG,ifnotfound=NA)
# Make empty list
entrezid <- character(length = nrow(cnts.norm))
# Add NA's to empty list
entrezid[] <- NA
# Loop to only extract the entez ID's that are not NA
for(i in 1:length(entrezid))
{
  if(!is.na(entid[i]))
  {
    entrezid[i] <- entid[[i]][1]
  }
}

# Find out which entrezid id's are NA's
idx <- is.na(entrezid)
# Extract the genes with entrez id's
cnts.norm.sel <- cnts.norm[!idx,]
# Add entrezid names to the end of the probe anns table
probe.anns.sel <- data.frame(probe.anns[rownames(cnts.norm.sel),],
                             entrezid = entrezid[!idx],stringsAsFactors=FALSE)
# Change ensembl id's to entrez id's in counts matrix
rownames(cnts.norm.sel) <- entrezid[!idx]

# Load pathway gene sets
data("go.sets.hs")
data("kegg.sets.hs")
# Declare which samples are of interest
samp.idx <- 1:dim(exp1.sel)[2]
# Declare background samples
ref.idx <- (dim(exp1.sel)[2]+1):dim(cnts.norm.sel)[2]
# Pathway analysis
cnts.kegg.p <- gage(cnts.norm.sel, gsets = kegg.sets.hs,
                    ref = ref.idx, samp = samp.idx, compare ="unpaired")
cnts.d= cnts.norm.sel[, samp.idx]-rowMeans(cnts.norm.sel[, ref.idx])
# Combine probe annotation and normalized count data
diff.dat <- data.frame(probe.anns.sel, cnts.d)
# Select for upregulated kegg pathways that are significant and not NA
sel.up <- cnts.kegg.p$greater[,1:5]
# Select for less (downregulated) pathways
sel.down <- cnts.kegg.p$less[, 1:5]
# Write pathways to file
write.csv(sel.up, file=paste(name, name1, "vs", name2,"KEGG.upregulated.csv", sep = "."))
write.csv(sel.down, file=paste(name, name1, "vs", name2,"KEGG.downregulated.csv", sep = "."))
```

Gene Set Analysis of Gamma Cells

```
# Load in libraries
suppressPackageStartupMessages(library(Biobase))
suppressPackageStartupMessages(library(org.Hs.eg.db))
suppressPackageStartupMessages(library(gage))
suppressPackageStartupMessages(library(gageData))
library(Biobase)
library(org.Hs.eg.db)
library(gage)
library(gageData)
rm(list=ls())
setwd("/Users/lawlon/Documents/Final_RNA_Seq_3/Data/")
# Declare cell type of interest and name of comparison
name <- "NonT2D"
cell1 = c("PPY")
all.grps = c("INS", "GCG", "SST", "PPY")
# cell2 is all other islet cell types to be compared against cell1
cell2 <- all.grps[cell1!= all.grps]
name1 = "Gamma"
name2 = "Endocrine"
# Load NonT2D data
load("nonT2D.rdata")
# probe annotations and sample annotations
p.anns <- featureData(cnts.eset)
probe.anns <- as(p.anns,"data.frame")
s.anns1 <- pData(cnts.eset)
exp1 <- exprs(cnts.eset)
cell.anns1 <- s.anns1[s.anns1$cell.type %in% cell1,]
# Extract expression data of interest for cell1
exp1.sel <- exp1[, rownames(cell.anns1)]
cell.anns2 <- s.anns1[s.anns1$cell.type %in% cell2,]
exp2.sel <- exp1[,rownames(cell.anns2)]
# Combine expression data in single matrix
exp <- cbind(exp1.sel, exp2.sel)
# Function to truncate the sample names
# Result is run number and sample: 12th-C7
grp <- sapply(colnames(exp),function(dat) { strsplit(as.character(dat),"_")[[1]][1]})
group <- as.character(grp)
# Determine which genes have zero counts across all samples
sel.rn=rowSums(exp) != 0
# Extract only those genes with more than zero counts across samples
cnts=exp[sel.rn,]
# Library sizes = sum of column (sample) counts
libsizes=colSums(cnts)
# Normalize the library sizes
size.factor=libsizes/exp(mean(log(libsizes)))
# Normalize the counts and log transform
cnts.norm=t(t(cnts)/size.factor)
cnts.norm=log2(cnts.norm+8)

# Get entrez ID's from ensembl ids
entid <- mget(rownames(cnts.norm),org.Hs.egENSEMBL2EG,ifnotfound=NA)
```

```r
# Make empty list
entrezid <- character(length = nrow(cnts.norm))
# Add NA's to empty list
entrezid[] <- NA
# Loop to only extract the entez ID's that are not NA
for(i in 1:length(entrezid))
{
  if(!is.na(entid[i]))
  {
    entrezid[i] <- entid[[i]][1]
  }
}

# Find out which entrezid id's are NA's
idx <- is.na(entrezid)
# Extract the genes with entrez id's
cnts.norm.sel <- cnts.norm[!idx,]
# Add entrezid names to the end of the probe anns table
probe.anns.sel <- data.frame(probe.anns[rownames(cnts.norm.sel),],
                             entrezid = entrezid[!idx],stringsAsFactors=FALSE)
# Change ensembl id's to entrez id's in counts matrix
rownames(cnts.norm.sel) <- entrezid[!idx]

# Load pathway gene sets
data("go.sets.hs")
data("kegg.sets.hs")
# Declare which samples are of interest
samp.idx <- 1:dim(exp1.sel)[2]
# Declare background samples
ref.idx <- (dim(exp1.sel)[2]+1):dim(cnts.norm.sel)[2]
# Pathway analysis
cnts.kegg.p <- gage(cnts.norm.sel, gsets = kegg.sets.hs,
                    ref = ref.idx, samp = samp.idx, compare ="unpaired")
cnts.d= cnts.norm.sel[, samp.idx]-rowMeans(cnts.norm.sel[, ref.idx])
# Combine probe annotation and normalized count data
diff.dat <- data.frame(probe.anns.sel, cnts.d)
# Select for upregulated kegg pathways that are significant and not NA
sel.up <- cnts.kegg.p$greater[,1:5]
# Select for less (downregulated) pathways
sel.down <- cnts.kegg.p$less[, 1:5]
# Write pathways to file
write.csv(sel.up, file=paste(name, name1, "vs", name2,"KEGG.upregulated.csv", sep = "."))
write.csv(sel.down, file=paste(name, name1, "vs", name2,"KEGG.downregulated.csv", sep = "."))
```

Session Information

```r
# Load in libraries
suppressPackageStartupMessages(library(Biobase))
suppressPackageStartupMessages(library(org.Hs.eg.db))
suppressPackageStartupMessages(library(gage))
suppressPackageStartupMessages(library(gageData))
library(Biobase)
```

```r
library(org.Hs.eg.db)
library(gage)
library(gageData)
sessionInfo()
```

```
## R version 3.3.0 (2016-05-03)
## Platform: x86_64-apple-darwin13.4.0 (64-bit)
## Running under: OS X 10.11.3 (El Capitan)
##
## locale:
## [1] en_US.UTF-8/en_US.UTF-8/en_US.UTF-8/C/en_US.UTF-8/en_US.UTF-8
##
## attached base packages:
## [1] stats4    parallel  stats     graphics  grDevices utils     datasets
## [8] methods   base
##
## other attached packages:
## [1] gageData_2.10.0      gage_2.22.0          org.Hs.eg.db_3.3.0
## [4] AnnotationDbi_1.34.3 IRanges_2.6.0        S4Vectors_0.10.1
## [7] Biobase_2.32.0       BiocGenerics_0.18.0
##
## loaded via a namespace (and not attached):
##  [1] graph_1.50.0      Rcpp_0.12.5       knitr_1.13
##  [4] XVector_0.12.0    magrittr_1.5      zlibbioc_1.18.0
##  [7] R6_2.1.2          stringr_1.0.0     httr_1.1.0
## [10] tools_3.3.0       png_0.1-7         DBI_0.4-1
## [13] htmltools_0.3.5   yaml_2.1.13       digest_0.6.9
## [16] formatR_1.4       KEGGREST_1.12.2   evaluate_0.9
## [19] RSQLite_1.0.0     rmarkdown_0.9.6   stringi_1.1.1
## [22] Biostrings_2.40.2
```
