# Supplementary material for: Single-cell transcriptomes identify human islet cell signatures and reveal cell-type–specific expression changes in type 2 diabetes
Source: Genome Res. 2017 Feb;27(2):208–22. doi: 10.1101/gr.212720.116 (PMC5287227; doi:10.1101/gr.212720.116)
Supplement: Supplemental Material [file supp_gr.212720.116_Supplemental_Methods_Source_Code.zip › Supplemental_Methods_Source_Code/Supplemental_Table_Source_Code/Supplemental_Table_S9_Source_Code.pdf]

Signature Gene Overlap with Cell Surface Markers

Introduction

This file will detail the steps used to determine which of the defined endocrine and exocrine signature genes were also cell surface markers or receptors. A list of approximately 3000 cell surface markers or receptors from Swiss Prot were used and are provided in Supplemental Table S8. Furthermore, the results of this analysis are provided in Supplemental Table S8.

Signature Gene Overlap with Surface Markers or Receptors

```
suppressPackageStartupMessages(library(readxl))
library(readxl)
rm(list=ls())
# Load in signature genes
sig <- read.csv("/Users/lawlon/Documents/Final_RNA_Seq_3/Signature_Genes/NonT2D.Endo.and.Exo.Signature.g
                header = FALSE, check.names = FALSE, row.names = NULL)
sig.ens <- sig[,1]

# Load in gene annotations, convert to gene symbol
load("/Users/lawlon/Documents/Final_RNA_Seq_3/Data/nonT2D.rdata")
p.anns <- as(featureData(cnts.eset), "data.frame")

sig.ids <- NULL
for (i in 1:length(sig.ens)) {
  idx <- which(rownames(p.anns)==sig.ens[i])
  sig.ids <- c(sig.ids,idx)
}

sig.gen <- p.anns$Associated.Gene.Name[sig.ids]
# Combine symbols with ensembl and cell type
comb <- cbind(sig, sig.gen)
comb <- comb[,c(1,3,2)]
colnames(comb) <- c("Ensembl_ID", "Gene_Symbol", "Signature_Cell_Type")

# Load in surface marker list
library(readxl)
setwd("/Users/lawlon/Documents/Final_RNA_Seq_3/Swiss_Prot/")
surf <- read_excel(path = "uniprot-%28cell+membrane+reviewed%3Ayes+organism%3A%22Homo+sapiens+%28Human%
                   col_names = TRUE)
surf.genes <- unique(surf$`Gene names`)

# Split the genes by a space
surf.spl <- strsplit(x = surf.genes, split = " ")

overlaps <- NULL
# Loop through the list, find intersection of sig genes and markers
  # Then take the unique list of overlaps
for (i in 1:length(surf.spl)) {
  ov <- intersect(sig.gen, surf.spl[[i]])
  overlaps <- c(overlaps, ov)
```

```r
}

# Take unique of overlaps
uniq.ov <- unique(overlaps)

# Determine which genes these are signature to
un.id <- NULL
for (j in 1:length(uniq.ov)) {
  idx <- which(comb$Gene_Symbol == uniq.ov[j])
  un.id <- c(un.id, idx)
}

# Extract overlapped sig genes
ov.sel <- comb[un.id,]

# Sort by signature cell type
ov.sort <- ov.sel[order(ov.sel$Signature_Cell_Type),]

# write file to name
write.csv(ov.sort, file = "Signature.Gene.Overlap.with.Cell.Surface.Proteins.csv")
```

Average Expression of Surface Markers across Cell Types

```r
suppressPackageStartupMessages(library(edgeR))
suppressPackageStartupMessages(library(Biobase))
library(Biobase)
library(edgeR)
# Load in single cell data
setwd("/Users/lawlon/Documents/Final_RNA_Seq_3/Data/")
load("nonT2D.rdata")
p.anns <- as(featureData(cnts.eset), "data.frame")
s.anns <- pData(cnts.eset)
counts <- exprs(cnts.eset)
# Calculate the cpm of the data
cpms <- cpm(x = counts)
data <- log2(cpms+1)
# Load in bulk data
load("/Users/lawlon/Documents/Final_RNA_Seq/islet_bulk_uniq_data.rdata")
bulk.counts <- exprs(bulk.cnts)
bulk.anns <- pData(bulk.cnts)
# Get bulk intact, ND samples
intact <- which(bulk.anns$Type == "Intact" & bulk.anns$Phenotype == "ND")
# Calculate cpm
bulk.cpm <- cpm(x = bulk.counts)
bulk.log <- log2(bulk.cpm+1)
bulk.sel <- bulk.log[, intact]
bulk.avg <- rowMeans(bulk.sel)

# Get cell types in order
s.1 <- s.anns[s.anns$cell.type %in% c("INS"),]
s.2 <- s.anns[s.anns$cell.type %in% c("GCG"),]
s.3 <- s.anns[s.anns$cell.type %in% c("SST"),]
```

```r
s.4 <- s.anns[s.anns$cell.type %in% c("PPY"),]
s.5 <- s.anns[s.anns$cell.type %in% c("PRSS1"),]
s.6 <- s.anns[s.anns$cell.type %in% c("KRT19"),]
s.7 <- s.anns[s.anns$cell.type %in% c("COL1A1"),]

# Get Expression matrices and average mean expression
f.1 <- data[, rownames(s.1)]
avg1 <- rowMeans(f.1)
f.2 <- data[, rownames(s.2)]
avg2 <- rowMeans(f.2)
f.3 <- data[, rownames(s.3)]
avg3 <- rowMeans(f.3)
f.4 <- data[, rownames(s.4)]
avg4 <- rowMeans(f.4)
f.5 <- data[, rownames(s.5)]
avg5 <- rowMeans(f.5)
f.6 <- data[, rownames(s.6)]
avg6 <- rowMeans(f.6)
f.7 <- data[, rownames(s.7)]
avg7 <- rowMeans(f.7)

# Match up cell type with hormone marker
namelist <- c(INS="Beta", GCG="Alpha", SST="Delta", PPY="Gamma",
              GHRL="Epsilon", COL1A1="Stellate", PRSS1="Acinar", KRT19="Ductal", none="None")
# Combine all cell expression data into one matrix
mat.avg <- cbind(avg1, avg2, avg3, avg4, avg5, avg6, avg7, bulk.avg)
colnames(mat.avg) <- c("Beta", "Alpha", "Delta", "Gamma","Acinar", "Ductal", "Stellate", "Bulk")
# Change rownames of mat to gene symbol
rownames(mat.avg) <- p.anns$Associated.Gene.Name
# read in cell surface genes
surf <- read.csv("/Users/lawlon/Documents/Final_RNA_Seq_3/Swiss_Prot/Signature.Gene.Overlap.with.Cell.Su
                 header = TRUE, check.names = FALSE, row.names = 1, stringsAsFactors = F)
genes.sel <- surf$Gene_Symbol
genes.sel <- unique(genes.sel)

genes.ids <- NULL
for (i in 1:length(genes.sel)) {
  idx <- which(rownames(mat.avg) == genes.sel[i])
  genes.ids <- c(genes.ids, idx)
}
# Extract genes of interest
mat.sel <- mat.avg[genes.ids,]
# Write expression matrix to file
write.csv(x = mat.sel, file = paste(fname, "average.log2cpm.csv", sep = "."))
```

Session Information

```r
suppressPackageStartupMessages(library(readxl))
suppressPackageStartupMessages(library(edgeR))
suppressPackageStartupMessages(library(Biobase))
library(Biobase)
library(edgeR)
```

```r
library(readxl)
sessionInfo()
```

```
## R version 3.3.0 (2016-05-03)
## Platform: x86_64-apple-darwin13.4.0 (64-bit)
## Running under: OS X 10.11.3 (El Capitan)
##
## locale:
## [1] en_US.UTF-8/en_US.UTF-8/en_US.UTF-8/C/en_US.UTF-8/en_US.UTF-8
##
## attached base packages:
## [1] parallel  stats     graphics  grDevices utils     datasets  methods
## [8] base
##
## other attached packages:
## [1] Biobase_2.32.0      BiocGenerics_0.18.0 edgeR_3.14.0
## [4] limma_3.28.11       readxl_0.1.1
##
## loaded via a namespace (and not attached):
##  [1] Rcpp_0.12.5     digest_0.6.9    formatR_1.4     magrittr_1.5
##  [5] evaluate_0.9    stringi_1.1.1   rmarkdown_0.9.6 tools_3.3.0
##  [9] stringr_1.0.0   yaml_2.1.13     htmltools_0.3.5 knitr_1.13
```
