# Supplementary material for: Single-cell transcriptomes identify human islet cell signatures and reveal cell-type–specific expression changes in type 2 diabetes
Source: Genome Res. 2017 Feb;27(2):208–22. doi: 10.1101/gr.212720.116 (PMC5287227; doi:10.1101/gr.212720.116)
Supplement: Supplemental Material [file supp_gr.212720.116_Supplemental_Fig_S1.pdf]

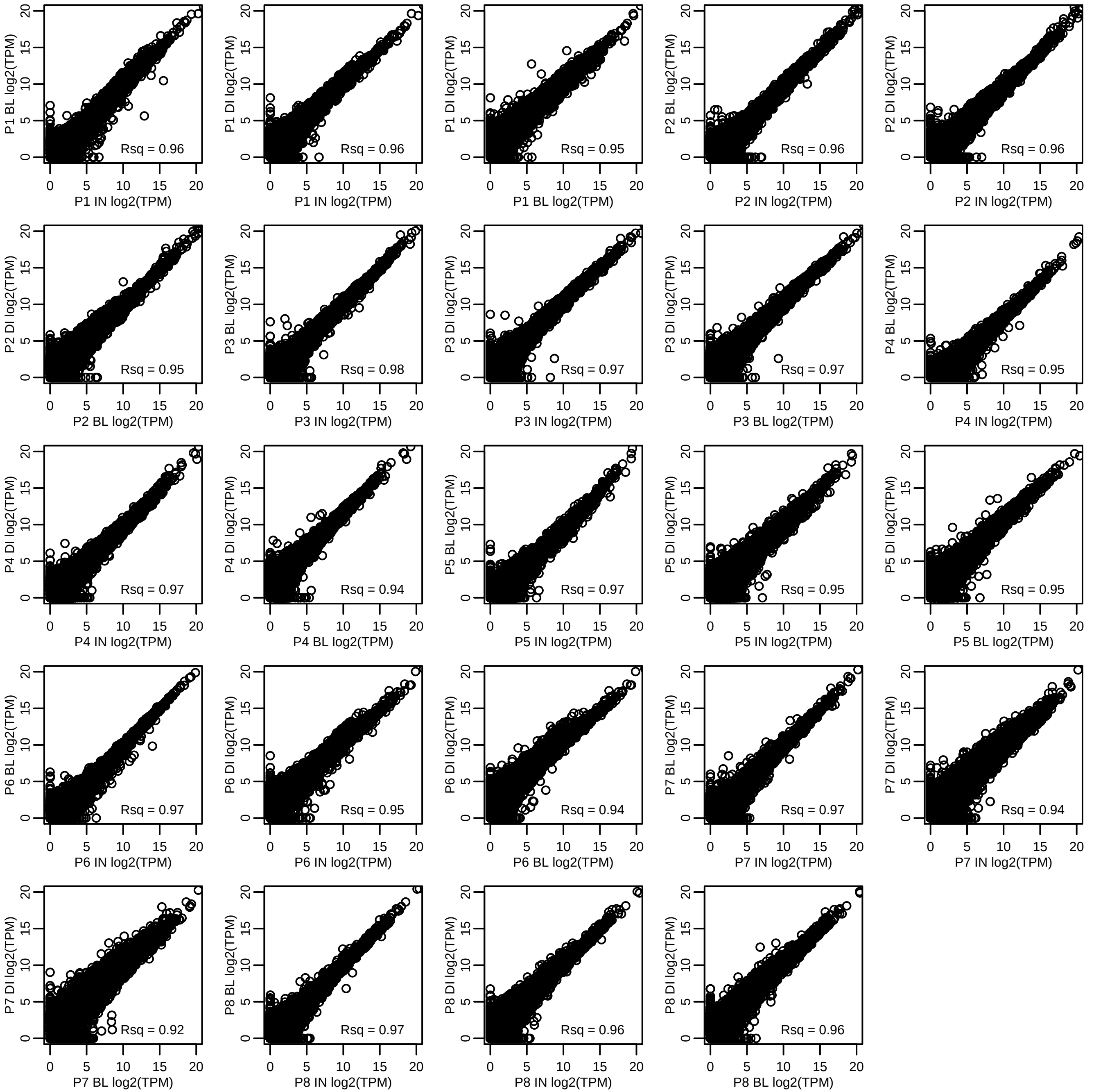
P1 BL log2(TPM)
Rsq = 0.96
P1 IN log2(TPM)
P1 DI log2(TPM)
Rsq = 0.96
P1 IN log2(TPM)
P1 DI log2(TPM)
Rsq = 0.95
P1 BL log2(TPM)
P2 BL log2(TPM)
Rsq = 0.96
P2 IN log2(TPM)
P2 DI log2(TPM)
Rsq = 0.96
P2 IN log2(TPM)
P2 DI log2(TPM)
Rsq = 0.95
P2 BL log2(TPM)
P3 BL log2(TPM)
Rsq = 0.98
P3 IN log2(TPM)
P3 DI log2(TPM)
Rsq = 0.97
P3 IN log2(TPM)
P3 DI log2(TPM)
Rsq = 0.97
P3 BL log2(TPM)
P4 BL log2(TPM)
Rsq = 0.95
P4 IN log2(TPM)
P4 DI log2(TPM)
Rsq = 0.97
P4 IN log2(TPM)
P4 DI log2(TPM)
Rsq = 0.94
P4 BL log2(TPM)
P5 BL log2(TPM)
Rsq = 0.97
P5 IN log2(TPM)
P5 DI log2(TPM)
Rsq = 0.95
P5 IN log2(TPM)
P5 DI log2(TPM)
Rsq = 0.95
P5 BL log2(TPM)
P6 BL log2(TPM)
Rsq = 0.97
P6 IN log2(TPM)
P6 DI log2(TPM)
Rsq = 0.95
P6 IN log2(TPM)
P6 DI log2(TPM)
Rsq = 0.94
P6 BL log2(TPM)
P7 BL log2(TPM)
Rsq = 0.97
P7 IN log2(TPM)
P7 DI log2(TPM)
Rsq = 0.94
P7 IN log2(TPM)
P7 DI log2(TPM)
Rsq = 0.92
P7 BL log2(TPM)
P8 BL log2(TPM)
Rsq = 0.97
P8 IN log2(TPM)
P8 DI log2(TPM)
Rsq = 0.96
P8 IN log2(TPM)
P8 DI log2(TPM)
Rsq = 0.96
P8 BL log2(TPM)

Supplemental_Fig_S1: Bulk baseline, intact, and dissociated transcriptomes within each patient islet correlate strongly regardless of experimental condition.

Correlation scatter plots comparing the average $\log_2$(TPM) gene expression of each patient islet bulk baseline, intact, and dissociated transcriptomes all demonstrate Pearson's R-squared values greater than 0.90. Processing and dissociation of islet samples did not drastically influence transcriptome profiles. IN = Bulk Intact, DI = Bulk Dissociated, BL = Bulk Baseline, P1 = Patient Donor 1, TPM = Transcripts per million, Rsq = Pearson's R-squared.
