# Supplementary material for: Single-cell transcriptomes identify human islet cell signatures and reveal cell-type–specific expression changes in type 2 diabetes
Source: Genome Res. 2017 Feb;27(2):208–22. doi: 10.1101/gr.212720.116 (PMC5287227; doi:10.1101/gr.212720.116)
Supplement: Supplemental Material [file supp_gr.212720.116_Supplemental_Fig_S2.pdf]

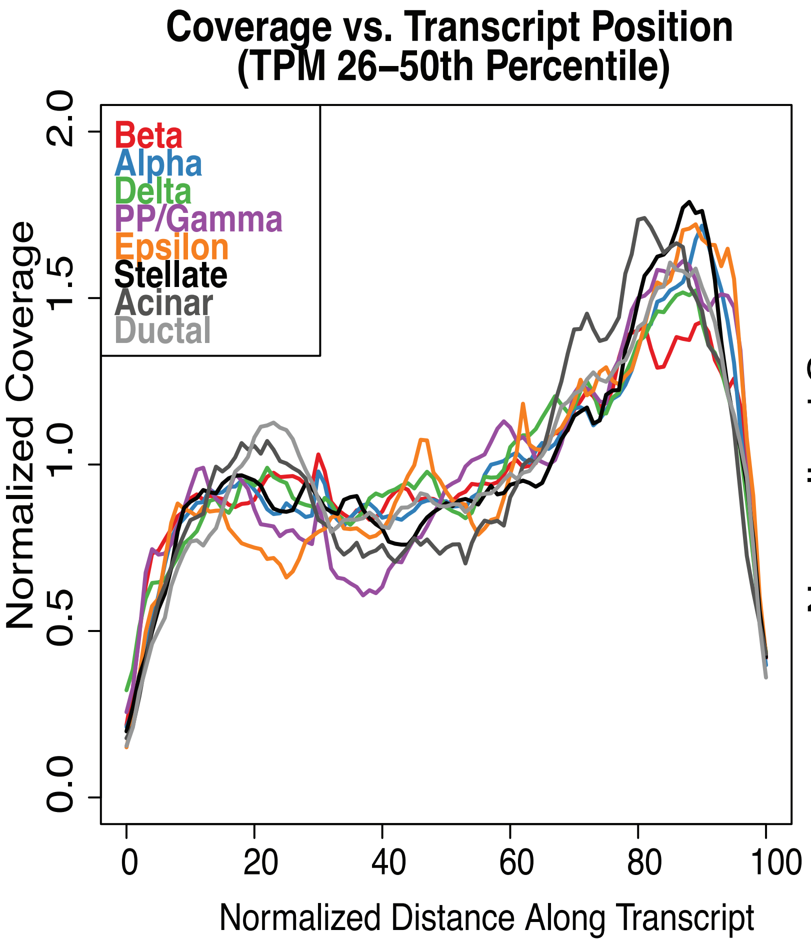

Coverage vs. Transcript Position
(TPM 26–50th Percentile)
Beta
Alpha
Delta
PP/Gamma
Epsilon
Stellate
Acinar
Ductal
Normalized Coverage
0.0
0.5
1.0
1.5
2.0
0
20
40
60
80
100
Normalized Distance Along Transcript

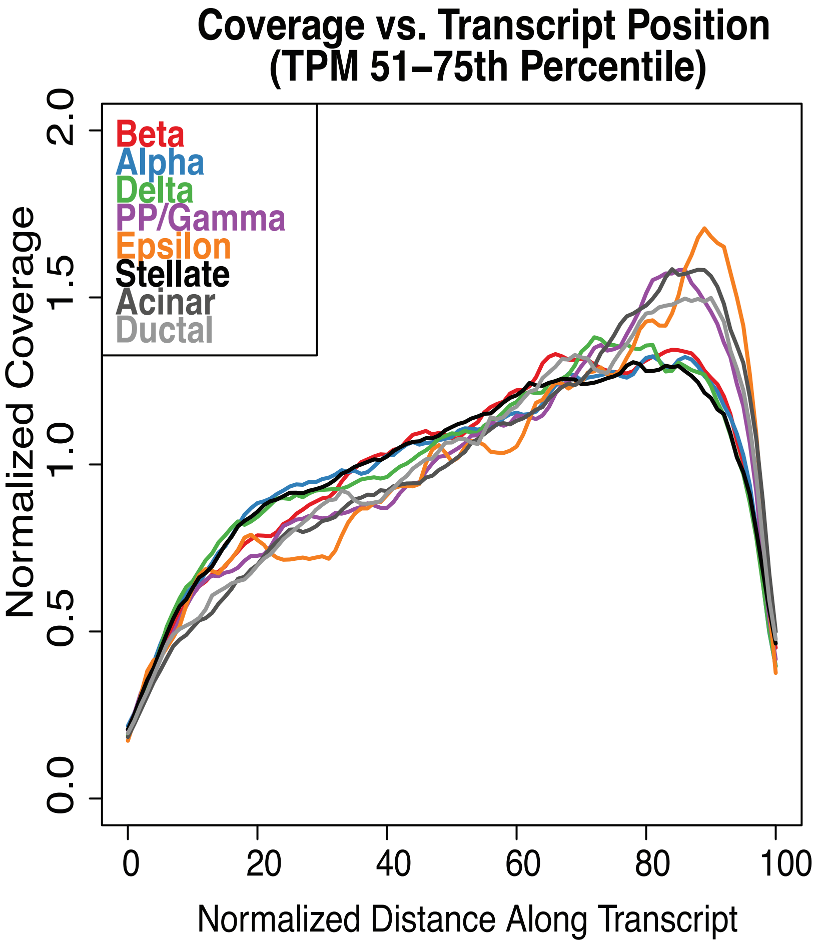

Coverage vs. Transcript Position
(TPM 51–75th Percentile)
Beta
Alpha
Delta
PP/Gamma
Epsilon
Stellate
Acinar
Ductal
Normalized Coverage
0.0
0.5
1.0
1.5
2.0
0
20
40
60
80
100
Normalized Distance Along Transcript

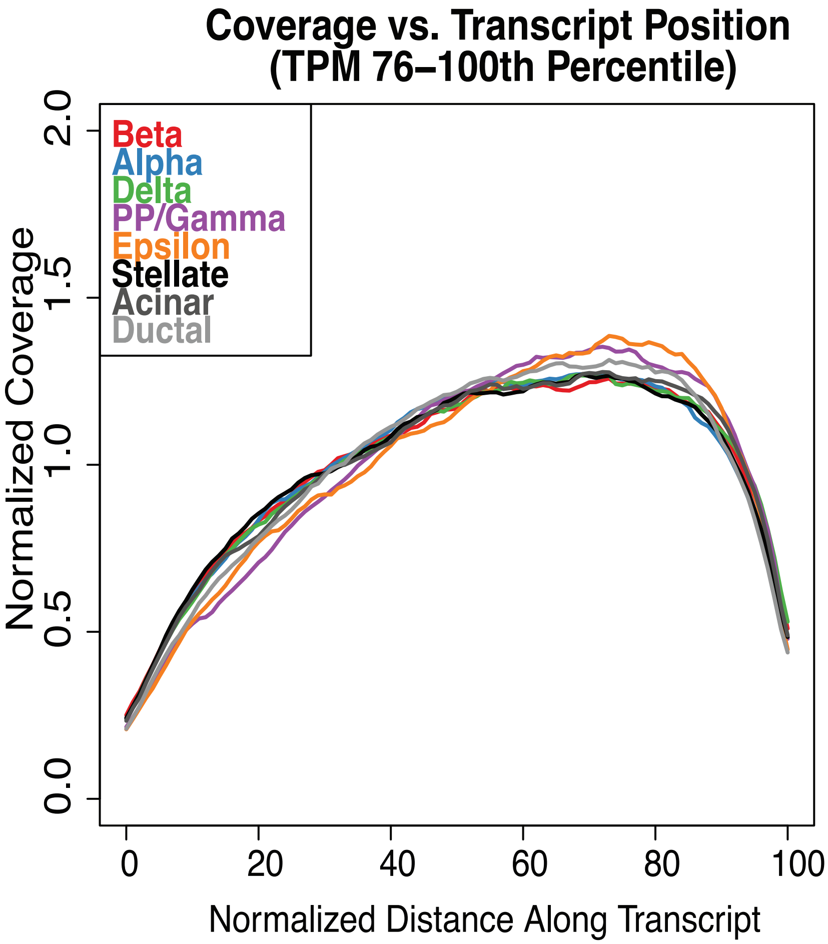

Coverage vs. Transcript Position
(TPM 76–100th Percentile)
Beta
Alpha
Delta
PP/Gamma
Epsilon
Stellate
Acinar
Ductal
Normalized Coverage
0.0
0.5
1.0
1.5
2.0
0
20
40
60
80
100
Normalized Distance Along Transcript

Supplemental_Fig_S2: Transcript coverage across single cell transcriptomes.

All genes were ranked by their log_2 transcript per million (TPM) expression level within each cell type and grouped into quartiles. Moderately expressed genes may be considered to reside in the 2nd (26 – 50th percentile) and 3rd (51 – 75th percentile) quartiles. We then used the program "CollectRNASeqMetrics" from Picard Tools (https://broadinstitute.github.io/picard/command-line-overview.html) to obtain and plot the normalized transcript coverage (y-axis) as a function of normalized transcript length (x-axis).
