# Supplementary material for: Single-cell transcriptomes identify human islet cell signatures and reveal cell-type–specific expression changes in type 2 diabetes
Source: Genome Res. 2017 Feb;27(2):208–22. doi: 10.1101/gr.212720.116 (PMC5287227; doi:10.1101/gr.212720.116)
Supplement: Supplemental Material [file supp_gr.212720.116_Supplemental_Fig_S3.pdf]

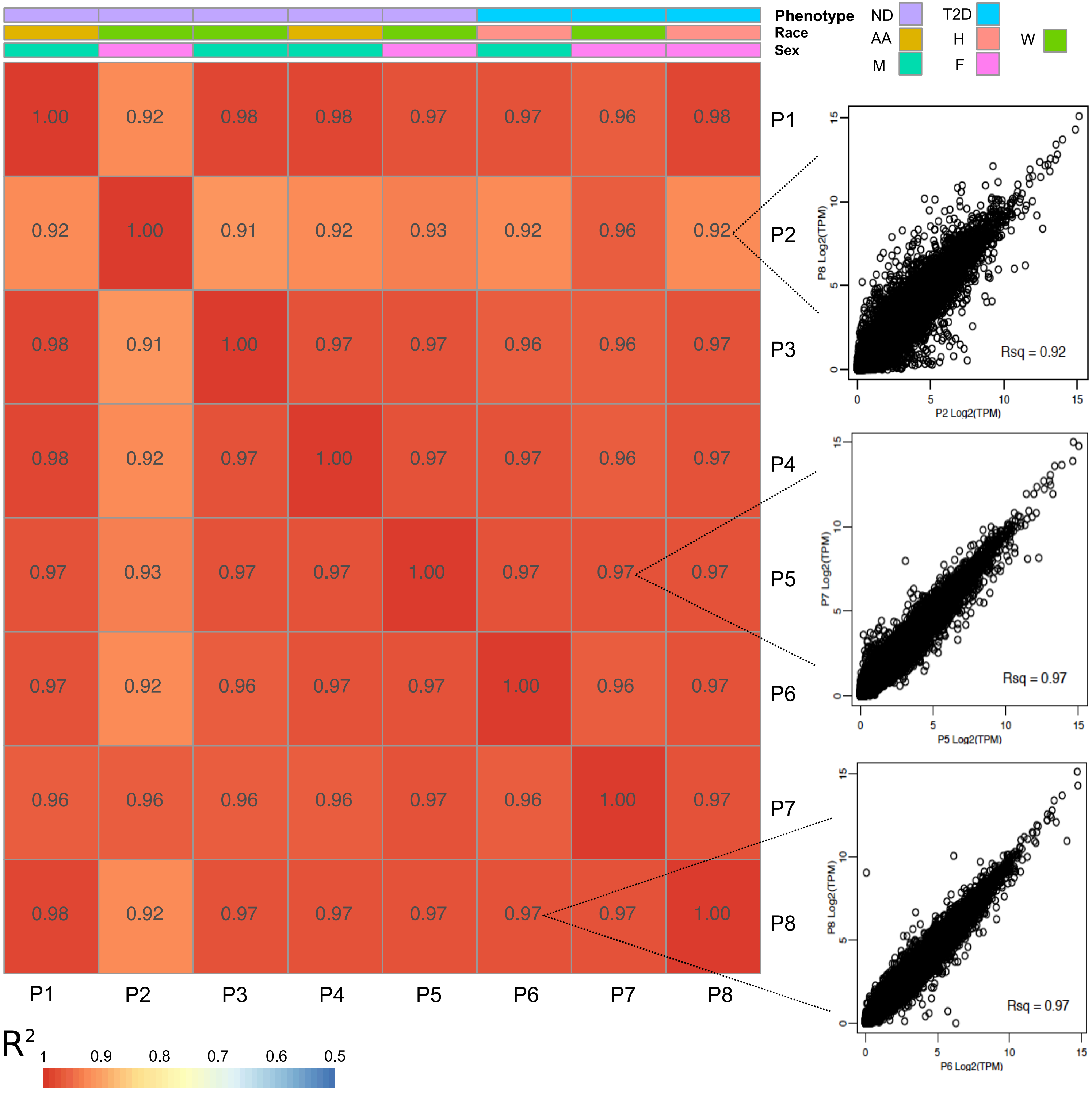

Phenotype
Race
Sex
ND
T2D
AA
H
W
M
F
P1 1.00 0.92 0.98 0.98 0.97 0.97 0.96 0.98
P2 0.92 1.00 0.91 0.92 0.93 0.92 0.96 0.92
P3 0.98 0.91 1.00 0.97 0.97 0.96 0.96 0.97
P4 0.98 0.92 0.97 1.00 0.97 0.97 0.96 0.97
P5 0.97 0.93 0.97 0.97 1.00 0.97 0.97 0.97
P6 0.97 0.92 0.96 0.97 0.97 1.00 0.96 0.97
P7 0.96 0.96 0.96 0.96 0.97 0.96 1.00 0.97
P8 0.98 0.92 0.97 0.97 0.97 0.97 0.97 1.00
P1 P2 P3 P4 P5 P6 P7 P8
R2
1 0.9 0.8 0.7 0.6 0.5
P8 Log2(TPM)
P2 Log2(TPM)
Rsq = 0.92
P7 Log2(TPM)
P5 Log2(TPM)
Rsq = 0.97
P8 Log2(TPM)
P6 Log2(TPM)
Rsq = 0.97
0 5 10 15

Supplemental_Fig_S3: Ensemble single cell transcriptomes from each patient islet are highly correlated.

Heatmap of Pearson's R-squared values between patient ensemble single cell transcriptomes. R-squared values ranged from 0.91 to 0.98 implying minimal batch effects across patient samples. Also shown are inset scatter plots for specific correlations providing further evidence that the single cell transcriptomes are well correlated between patients. TPM = Transcripts per million, R^2 = Pearson's R-squared, P = Patient Donor, ND = non-diabetic, T2D = Type 2 diabetic, H = Hispanic, W = White, AA = African American, M = Male, F = Female.
