# Supplementary material for: Single-cell transcriptomes identify human islet cell signatures and reveal cell-type–specific expression changes in type 2 diabetes
Source: Genome Res. 2017 Feb;27(2):208–22. doi: 10.1101/gr.212720.116 (PMC5287227; doi:10.1101/gr.212720.116)
Supplement: Supplemental Material [file supp_gr.212720.116_Supplemental_Fig_S4.pdf]

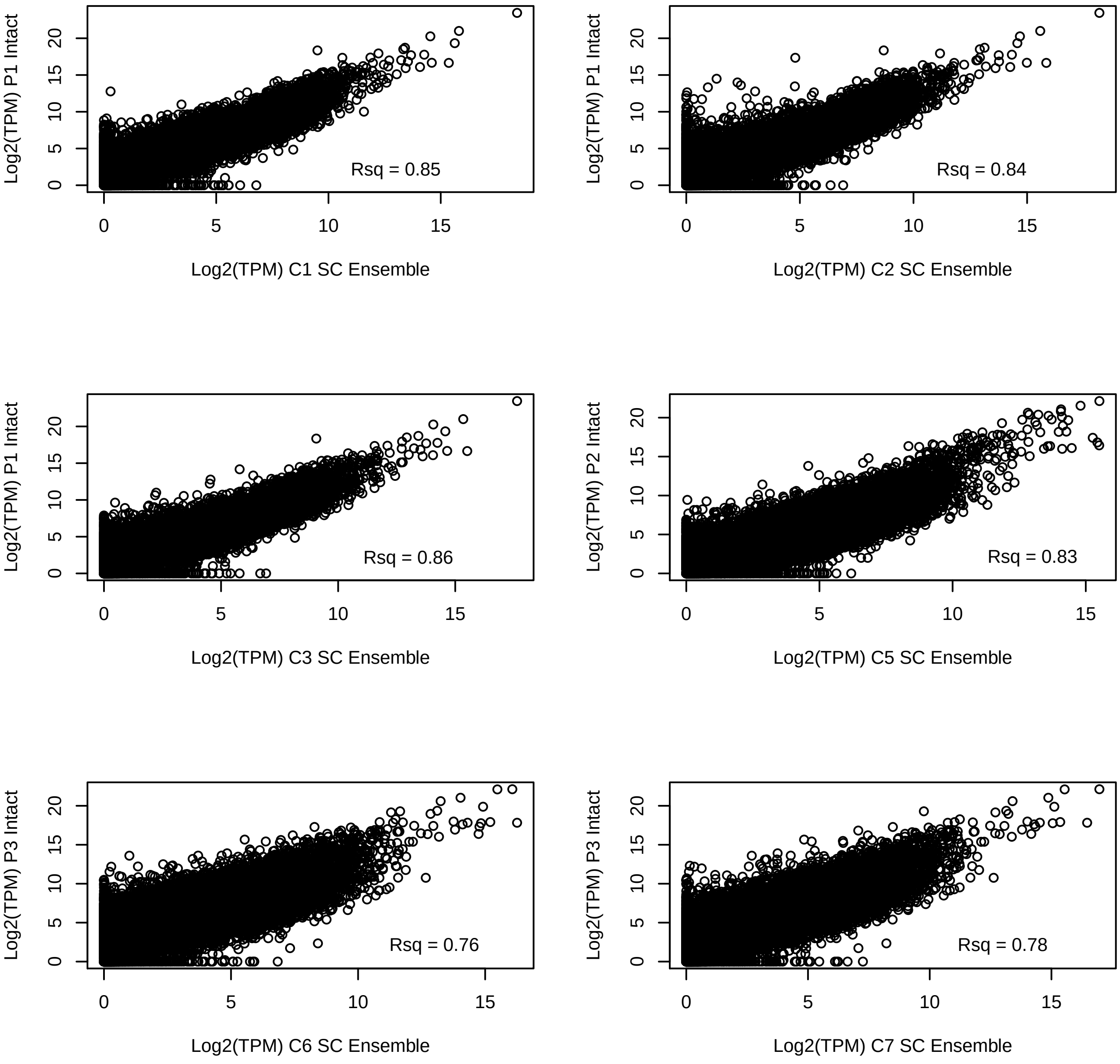

Log2(TPM) P1 Intact
Rsq = 0.85
Log2(TPM) C1 SC Ensemble
Log2(TPM) P1 Intact
Rsq = 0.84
Log2(TPM) C2 SC Ensemble
Log2(TPM) P1 Intact
Rsq = 0.86
Log2(TPM) C3 SC Ensemble
Log2(TPM) P2 Intact
Rsq = 0.83
Log2(TPM) C5 SC Ensemble
Log2(TPM) P3 Intact
Rsq = 0.76
Log2(TPM) C6 SC Ensemble
Log2(TPM) P3 Intact
Rsq = 0.78
Log2(TPM) C7 SC Ensemble

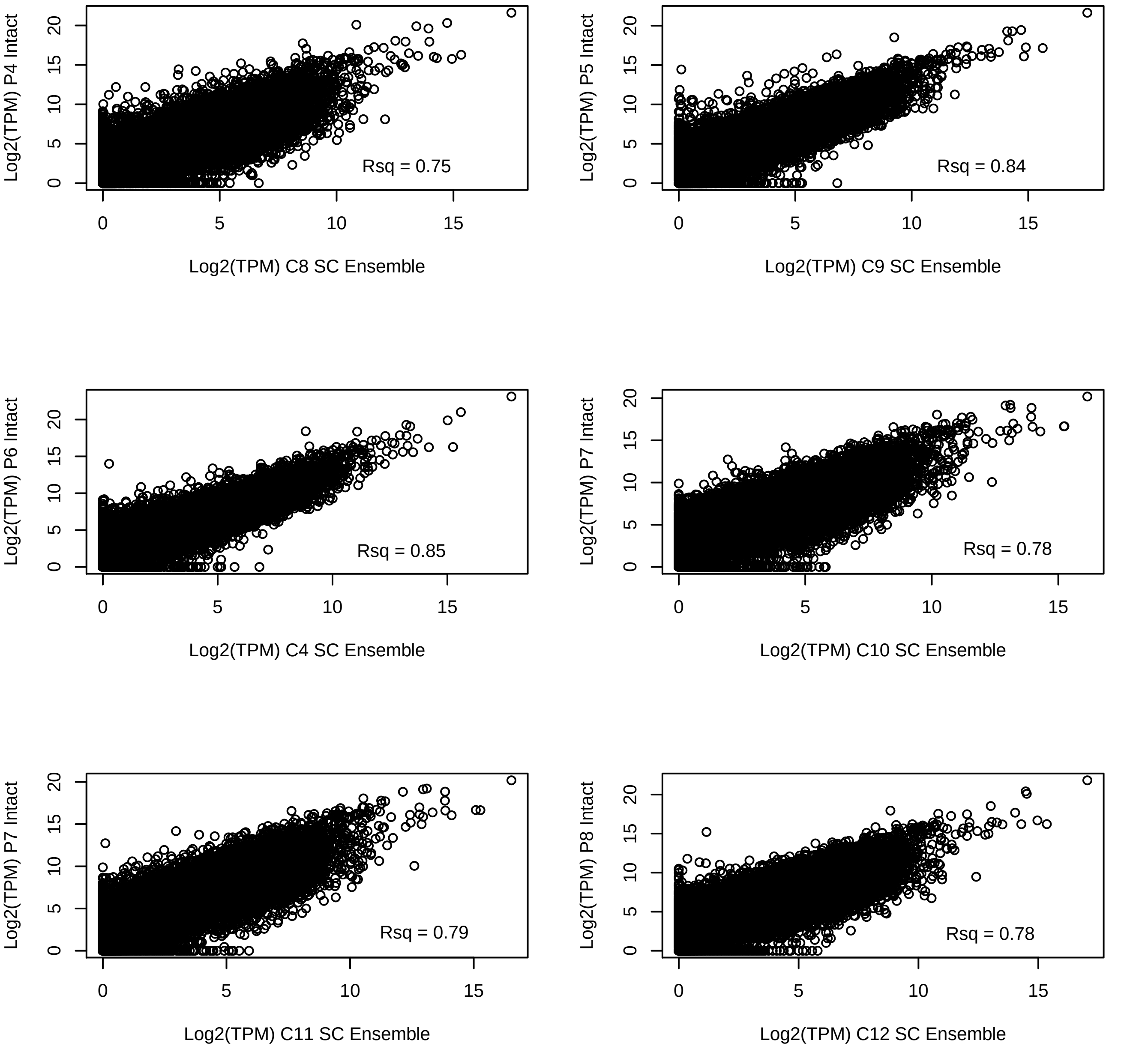

Log2(TPM) P4 Intact
Log2(TPM) C8 SC Ensemble
Rsq = 0.75
Log2(TPM) P5 Intact
Log2(TPM) C9 SC Ensemble
Rsq = 0.84
Log2(TPM) P6 Intact
Log2(TPM) C4 SC Ensemble
Rsq = 0.85
Log2(TPM) P7 Intact
Log2(TPM) C10 SC Ensemble
Rsq = 0.78
Log2(TPM) P7 Intact
Log2(TPM) C11 SC Ensemble
Rsq = 0.79
Log2(TPM) P8 Intact
Log2(TPM) C12 SC Ensemble
Rsq = 0.78

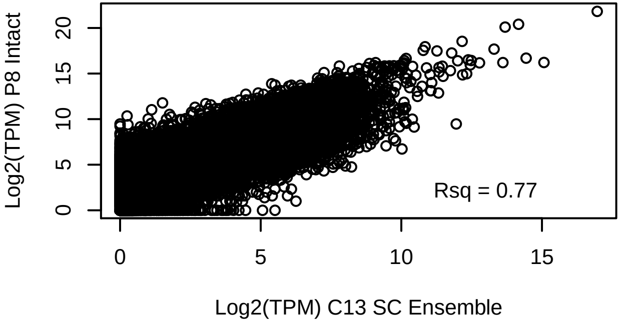

Log2(TPM) P8 Intact
0
5
10
15
20
Rsq = 0.77
0
5
10
15
Log2(TPM) C13 SC Ensemble

Supplemental_Fig_S4: Pairwise comparisons of patient bulk intact and ensemble single cell transcriptomes are highly correlated regardless of microfluidic chip preparation.

Scatter plots of average log_2(TPM) expression of bulk intact and ensemble single cell transcriptomes for each patient islet. The C1, C2, etc, labels correspond to the single cell microfluidic chip preparation(s). Pearson's R-square values range from 0.75 to 0.86. TPM = Transcripts per million, Rsq = Pearson's R-squared, SC = Single cell ensemble, P = Patient Donor.
