# Supplementary material for: Single-cell transcriptomes identify human islet cell signatures and reveal cell-type–specific expression changes in type 2 diabetes
Source: Genome Res. 2017 Feb;27(2):208–22. doi: 10.1101/gr.212720.116 (PMC5287227; doi:10.1101/gr.212720.116)
Supplement: Supplemental Material [file supp_gr.212720.116_Supplemental_Fig_S5.pdf]

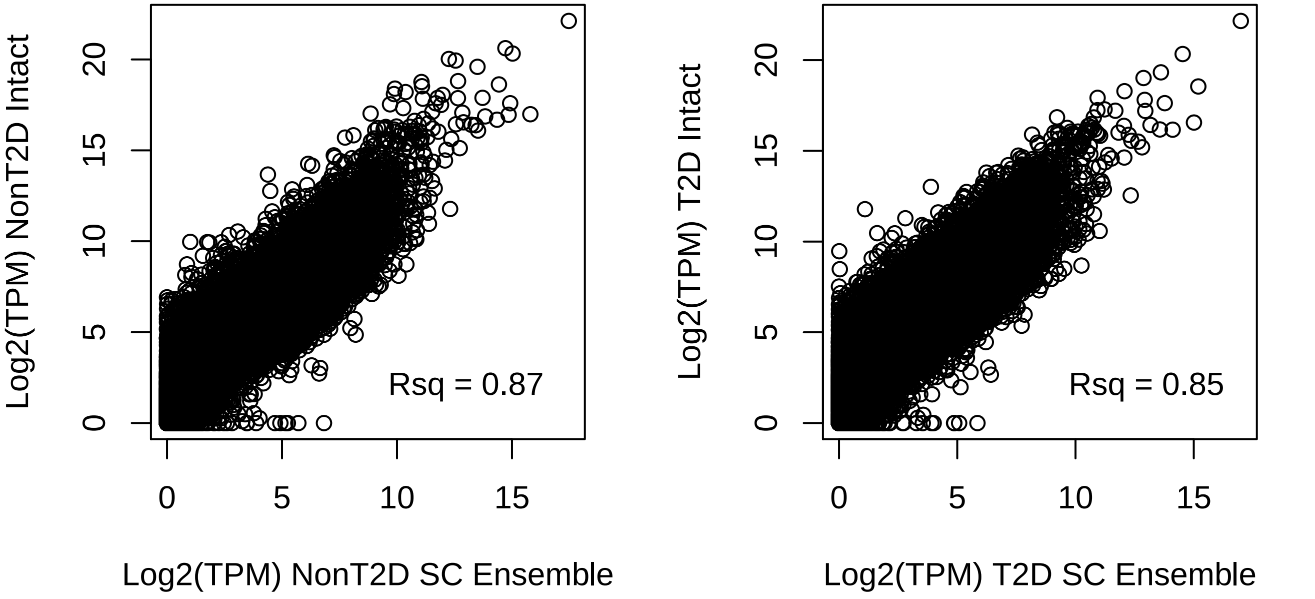

Log2(TPM) NonT2D Intact
Rsq = 0.87
Log2(TPM) NonT2D SC Ensemble
Log2(TPM) T2D Intact
Rsq = 0.85
Log2(TPM) T2D SC Ensemble

Supplemental_Fig_S5: Correlation of patient bulk intact and ensemble single cell transcriptomes is not influenced by disease state.

Correlation scatter plots of average log_2(TPM) gene expression of bulk intact and ensemble single cell transcriptomes from non-diabetic islets and those from Type 2 diabetic islets. The similar Pearson's R-squared values observed suggest that the correlation between bulk and single cell transcriptomes was not affected by disease state. TPM = Transcripts per million, Rsq = Pearson's R-squared, NonT2D = non-diabetic, T2D = Type 2 diabetic, SC = Single cell.
