# Supplementary figures and images for: Single-cell transcriptomes identify human islet cell signatures and reveal cell-type–specific expression changes in type 2 diabetes
Source: Genome Res. 2017 Feb;27(2):208–22. doi: 10.1101/gr.212720.116 (PMC5287227; doi:10.1101/gr.212720.116)

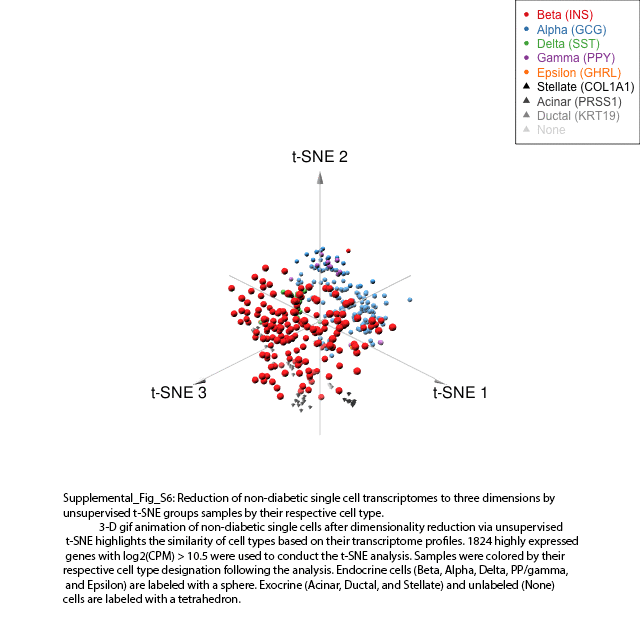

Supplement: Supplemental Material [file supp_gr.212720.116_Supplemental_Fig_S6.gif]

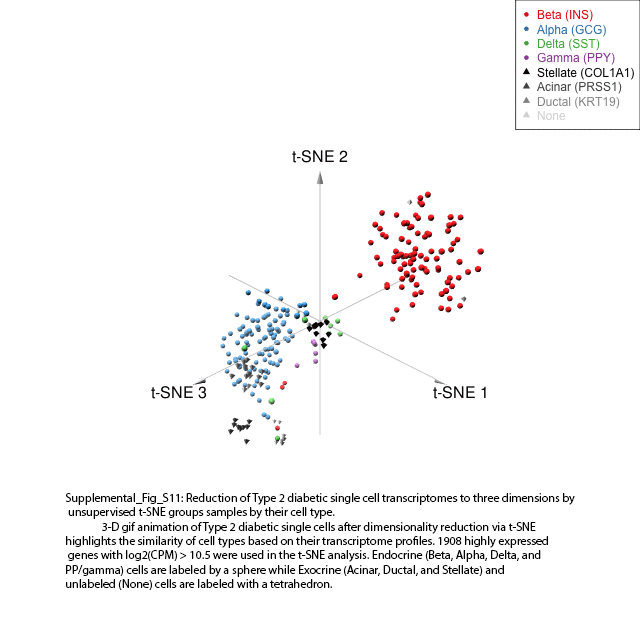

Supplement: Supplemental Material [file supp_gr.212720.116_Supplemental_Fig_S11.gif]

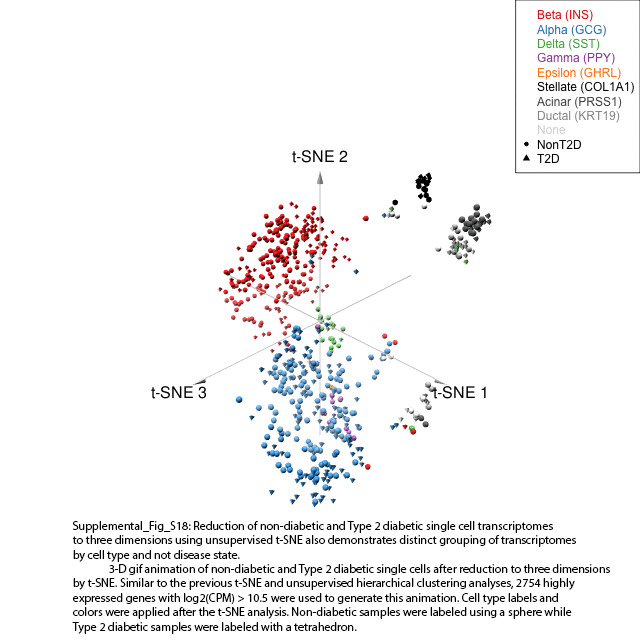

Supplement: Supplemental Material [file supp_gr.212720.116_Supplemental_Fig_S18.gif]
