# Supplementary material for: Single-cell transcriptomes identify human islet cell signatures and reveal cell-type–specific expression changes in type 2 diabetes
Source: Genome Res. 2017 Feb;27(2):208–22. doi: 10.1101/gr.212720.116 (PMC5287227; doi:10.1101/gr.212720.116)
Supplement: Supplemental Material [file supp_gr.212720.116_Supplemental_Fig_S7.pdf]

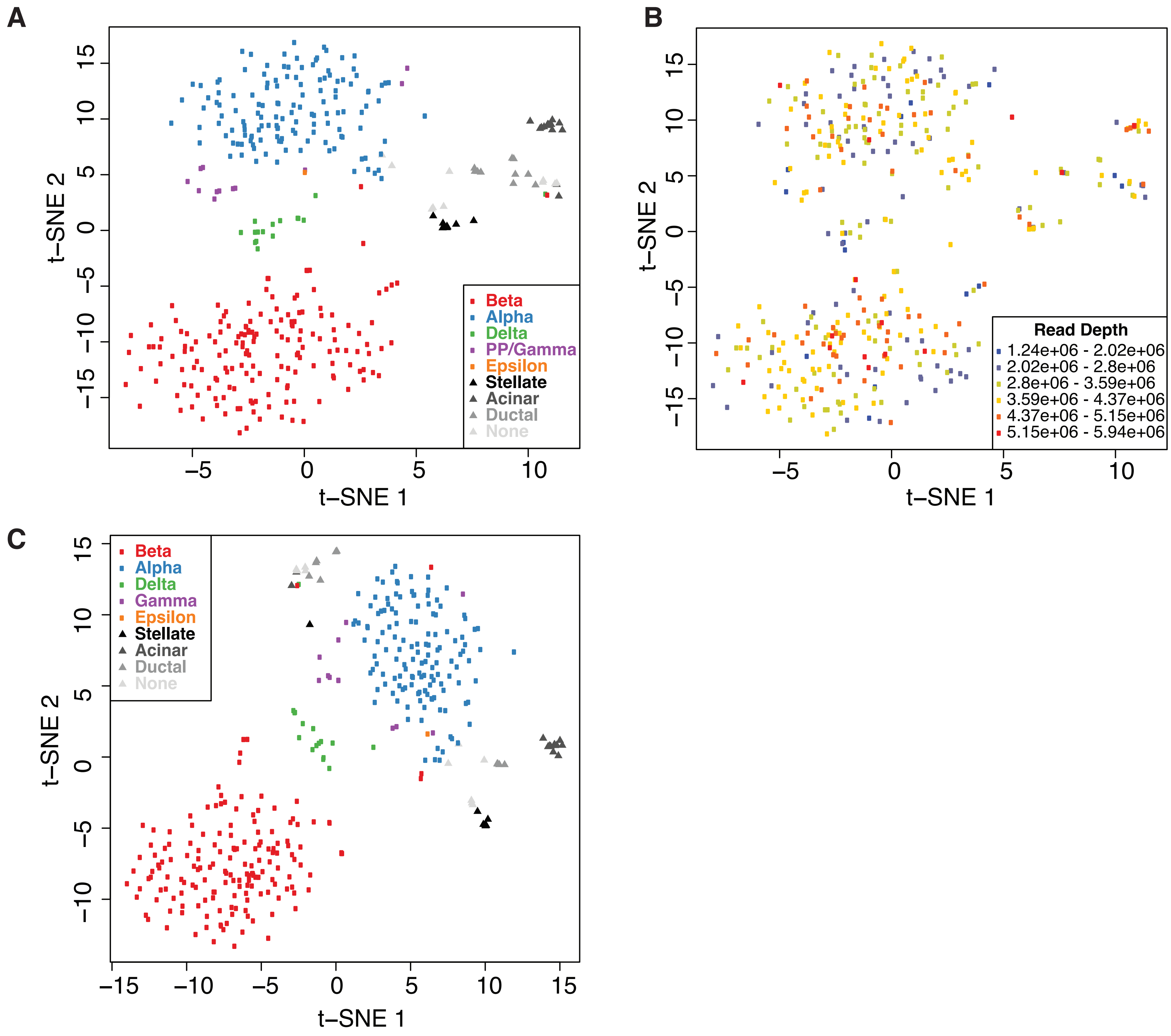
A
t–SNE 2
t–SNE 1
Beta
Alpha
Delta
PP/Gamma
Epsilon
Stellate
Acinar
Ductal
None
B
Read Depth
1.24e+06 - 2.02e+06
2.02e+06 - 2.8e+06
2.8e+06 - 3.59e+06
3.59e+06 - 4.37e+06
4.37e+06 - 5.15e+06
5.15e+06 - 5.94e+06
C
Gamma

Supplemental_Fig_S7: Reduction of non-diabetic single cell transcriptomes to two dimensions by unsupervised t-SNE separates samples by cell type regardless of marker gene expression or sequencing depth.

(A) Scatter plot of non-diabetic single cell transcriptomes in two dimensions after unsupervised t-SNE with 1824 of the most highly expression genes (log_2(CPM) >10.5) in at least one sample as shown in Figure 3A of the manuscript. (B) Scatter plot of the same t-SNE analysis with each cell shaded by their respective sequencing depth (number of reads). (C) Scatter plot of non-diabetic single cell samples in two dimensions after unsupervised t-SNE and excluding marker genes (*INS, GCG, SST, PPY, GHRL, COL1A1, PRSS1*, and *KRT19*). 1816 highly expressed genes with log_2(CPM) > 10.5 were used to conduct the t-SNE analysis. The color labels remain the same as seen in Figure 3A.
