# Supplementary material for: Single-cell transcriptomes identify human islet cell signatures and reveal cell-type–specific expression changes in type 2 diabetes
Source: Genome Res. 2017 Feb;27(2):208–22. doi: 10.1101/gr.212720.116 (PMC5287227; doi:10.1101/gr.212720.116)
Supplement: Supplemental Material [file supp_gr.212720.116_Supplemental_Fig_S8.pdf]

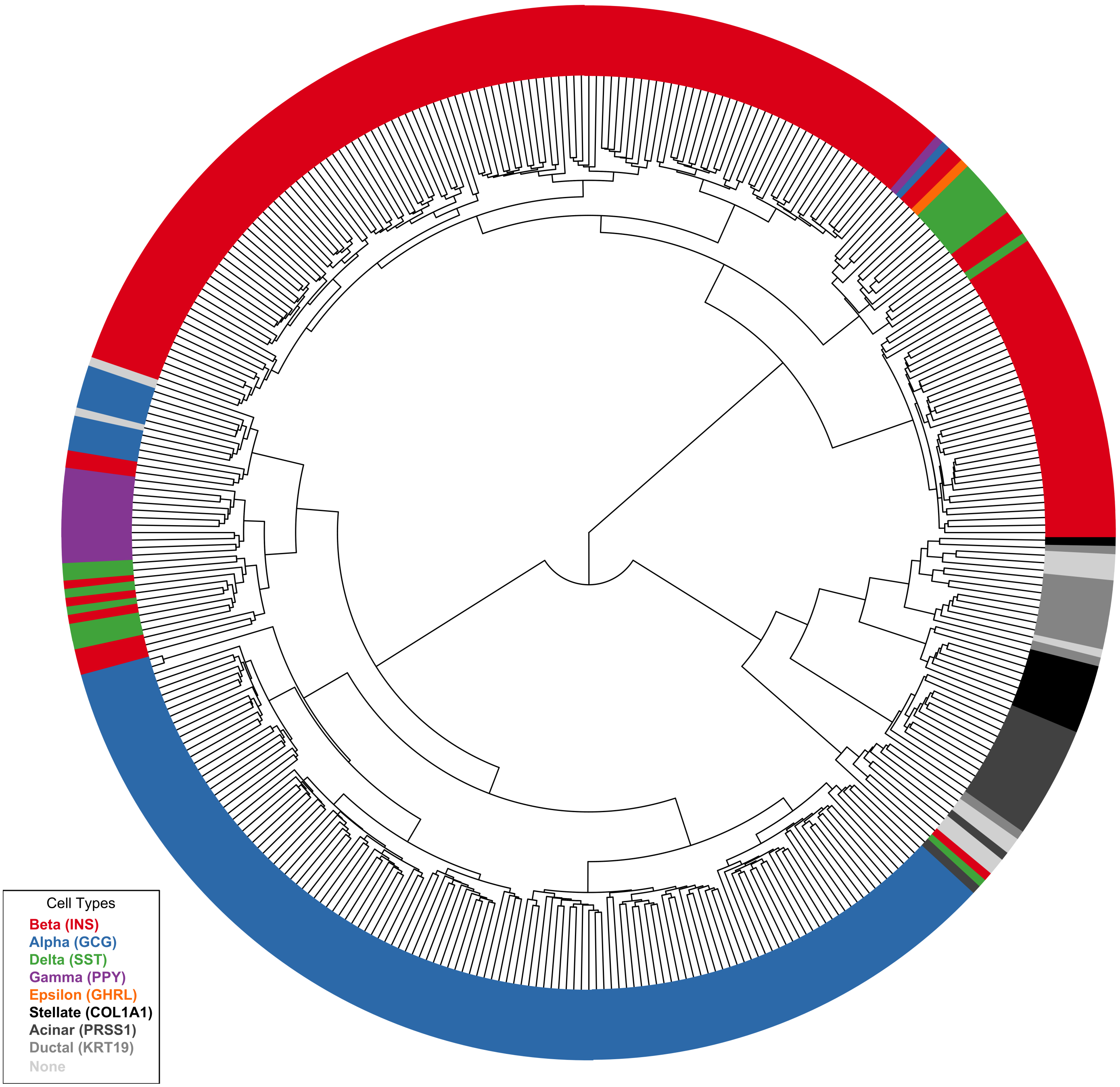

Cell Types
Beta (INS)
Alpha (GCG)
Delta (SST)
Gamma (PPY)
Epsilon (GHRL)
Stellate (COL1A1)
Acinar (PRSS1)
Ductal (KRT19)
None

Supplemental_Fig_S8: Unsupervised hierarchical clustering of non-diabetic single cell transcriptomes is not driven by marker gene expression.

Circular dendrogram illustrates unsupervised clustering of samples by cell type even after excluding marker genes (*INS, GCG, SST, PPY, GHRL, COL1A1, PRSS1*, and *KRT19*) from analysis. 1816 highly expressed genes with $\log_2(\mathrm{CPM}) > 10.5$ were used in the analysis and the color labels remained the same as shown in Figure 3B.
