# Supplementary material for: Single-cell transcriptomes identify human islet cell signatures and reveal cell-type–specific expression changes in type 2 diabetes
Source: Genome Res. 2017 Feb;27(2):208–22. doi: 10.1101/gr.212720.116 (PMC5287227; doi:10.1101/gr.212720.116)
Supplement: Supplemental Material [file supp_gr.212720.116_Supplemental_Fig_S9.pdf]

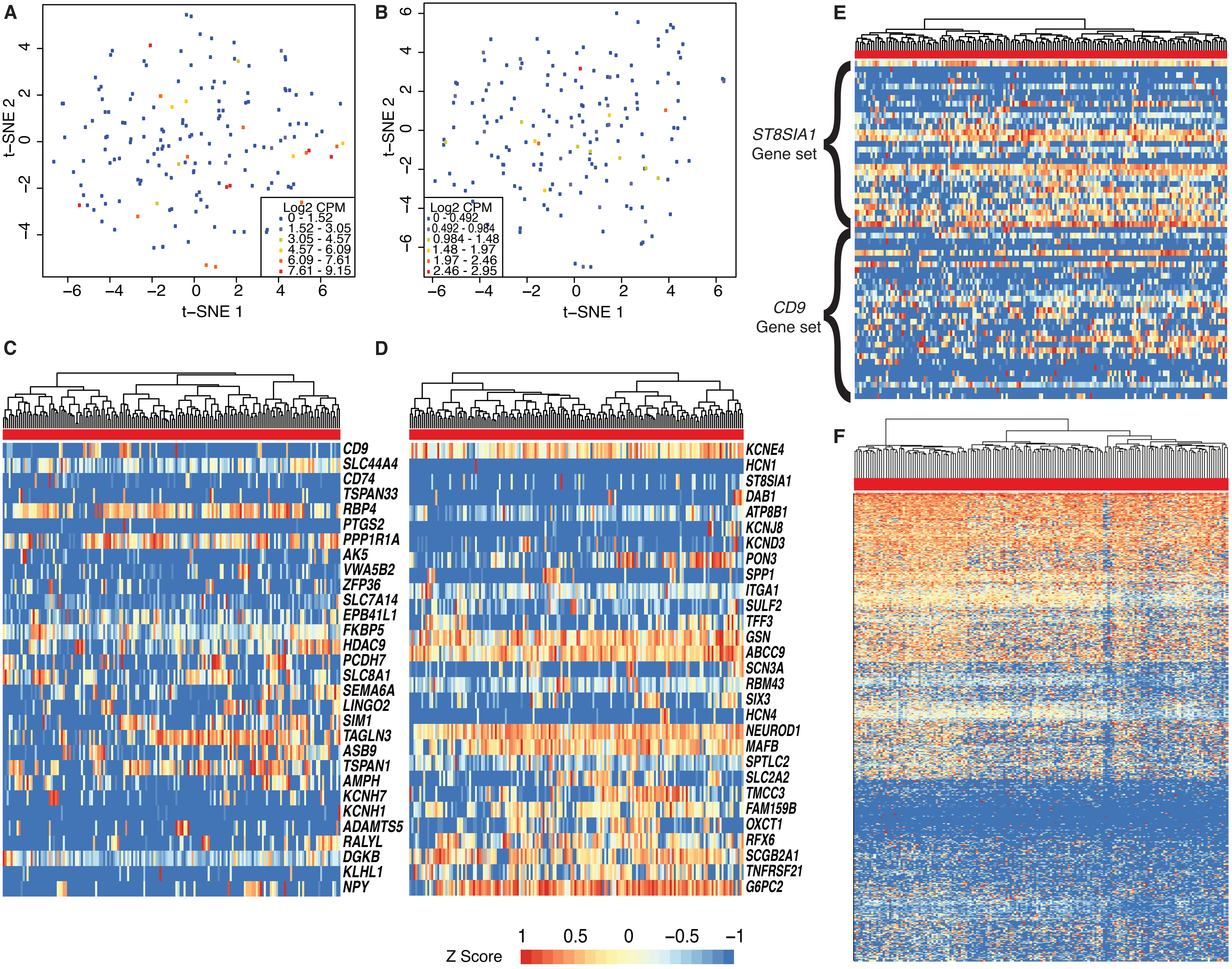
A
B
C
D
E
F
t−SNE 1
t−SNE 2
Log2 CPM
0 - 1.52
1.52 - 3.05
3.05 - 4.57
4.57 - 6.09
6.09 - 7.61
7.61 - 9.15
0 - 0.492
0.492 - 0.984
0.984 - 1.48
1.48 - 1.97
1.97 - 2.46
2.46 - 2.95
ST8SIA1 Gene set
CD9 Gene set
CD9
SLC44A4
CD74
TSPAN33
RBP4
PTGS2
PPP1R1A
AK5
VWA5B2
ZFP36
SLC7A14
EPB41L1
FKBP5
HDAC9
PCDH7
SLC8A1
SEMA6A
LINGO2
SIM1
TAGLN3
ASB9
TSPAN1
AMPH
KCNH7
KCNH1
ADAMTS5
RALYL
DGKB
KLHL1
NPY
KCNE4
HCN1
ST8SIA1
DAB1
ATP8B1
KCNJ8
KCND3
PON3
SPP1
ITGA1
SULF2
TFF3
GSN
ABCC9
SCN3A
RBM43
SIX3
HCN4
NEUROD1
MAFB
SPTLC2
SLC2A2
TMCC3
FAM159B
OXCT1
RFX6
SCGB2A1
TNFRSF21
G6PC2
Z Score
1
0.5
0
−0.5
−1

Supplemental_Fig_S9: Investigation of beta cell heterogeneity did not reveal distinct subpopulations.

(A) Unsupervised t-SNE and hierarchical clustering (C) analysis of non-diabetic beta cell transcriptomes (n=168) using 30 differential genes between subtypes B1/B3 and B2/B4 (Dorrell et al. 2016) does not reveal two distinct clusters. In (A) Samples are shaded by log_2CPM expression of *CD9*. (B) Unsupervised t-SNE and hierarchical clustering (D) analysis of the same beta cells using 29 differential genes between subtypes B1/B2 and B3/B4 (Dorrell et al. 2016) does not reveal two distinct clusters. In (B) Samples are shaded by log_2CPM expression of *ST8SIA1*. (E) Unsupervised hierarchical clustering with the combined gene sets (n=59 genes) does not identify four distinct subpopulations of beta cells. (F) Unsupervised hierarchical clustering of beta cell transcriptomes using differential transcripts distinguishing proliferating and mature mouse beta cells (Bader et al. 2016) does not identify two subpopulations of beta cells. Using the Mouse Genome Informatics (MGI; http://www.informatics.jax.org) database, these 996 transcripts corresponded to 768 genes. 726/768 genes corresponded to an MGI-annotated human orthologue, and 691 were detected/expressed in our human islet single cell data and were ultimately used for the clustering. Heat map values represent log_2(CPM) expression after mean-centering and scaling between -1 and 1.
