# Supplementary material for: Single-cell transcriptomes identify human islet cell signatures and reveal cell-type–specific expression changes in type 2 diabetes
Source: Genome Res. 2017 Feb;27(2):208–22. doi: 10.1101/gr.212720.116 (PMC5287227; doi:10.1101/gr.212720.116)
Supplement: Supplemental Material [file supp_gr.212720.116_Supplemental_Fig_S10.pdf]

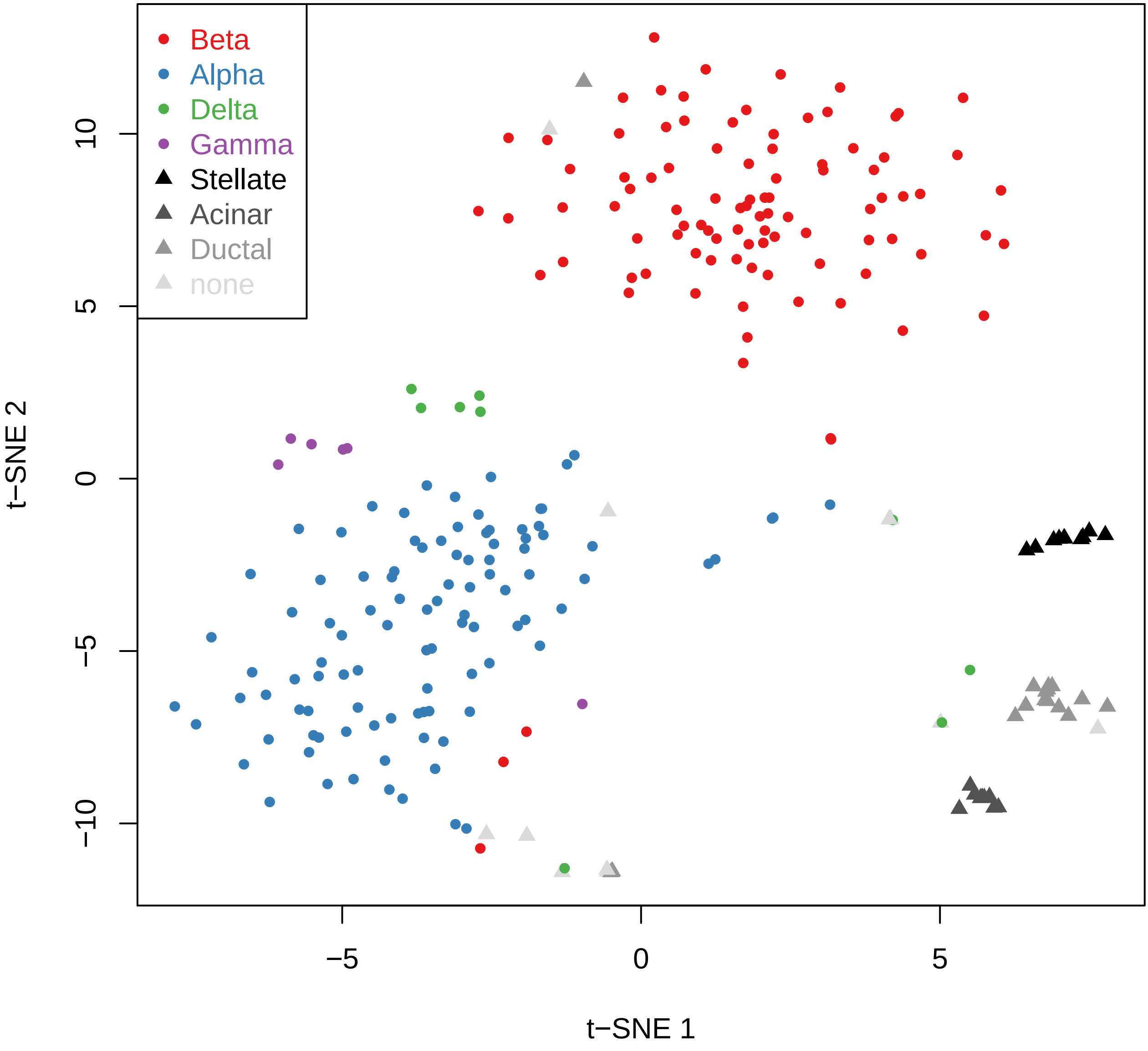

Beta
Alpha
Delta
Gamma
Stellate
Acinar
Ductal
none
10
5
0
−5
−10
t−SNE 2
−5
0
5
t−SNE 1

Supplemental_Fig_S10: Single cell transcriptomes from Type 2 diabetic islets group by cell type after reduction to two dimensions by unsupervised t-SNE.

Scatter plot of Type 2 diabetic single cells in two dimensions after t-SNE analysis highlights the similarity of cell types based on their transcriptome profiles. 1908 highly expressed genes with $\log_2(CPM) > 10.5$ were used in the t-SNE analysis. Similar to Figure 3A, Endocrine cells (Beta, Alpha, Delta, and PP/gamma) are labeled by a circle while Exocrine (Acinar, Ductal, and Stellate) and unlabeled (None) cells are labeled with a triangle.
