# Supplementary material for: Single-cell transcriptomes identify human islet cell signatures and reveal cell-type–specific expression changes in type 2 diabetes
Source: Genome Res. 2017 Feb;27(2):208–22. doi: 10.1101/gr.212720.116 (PMC5287227; doi:10.1101/gr.212720.116)
Supplement: Supplemental Material [file supp_gr.212720.116_Supplemental_Fig_S12.pdf]

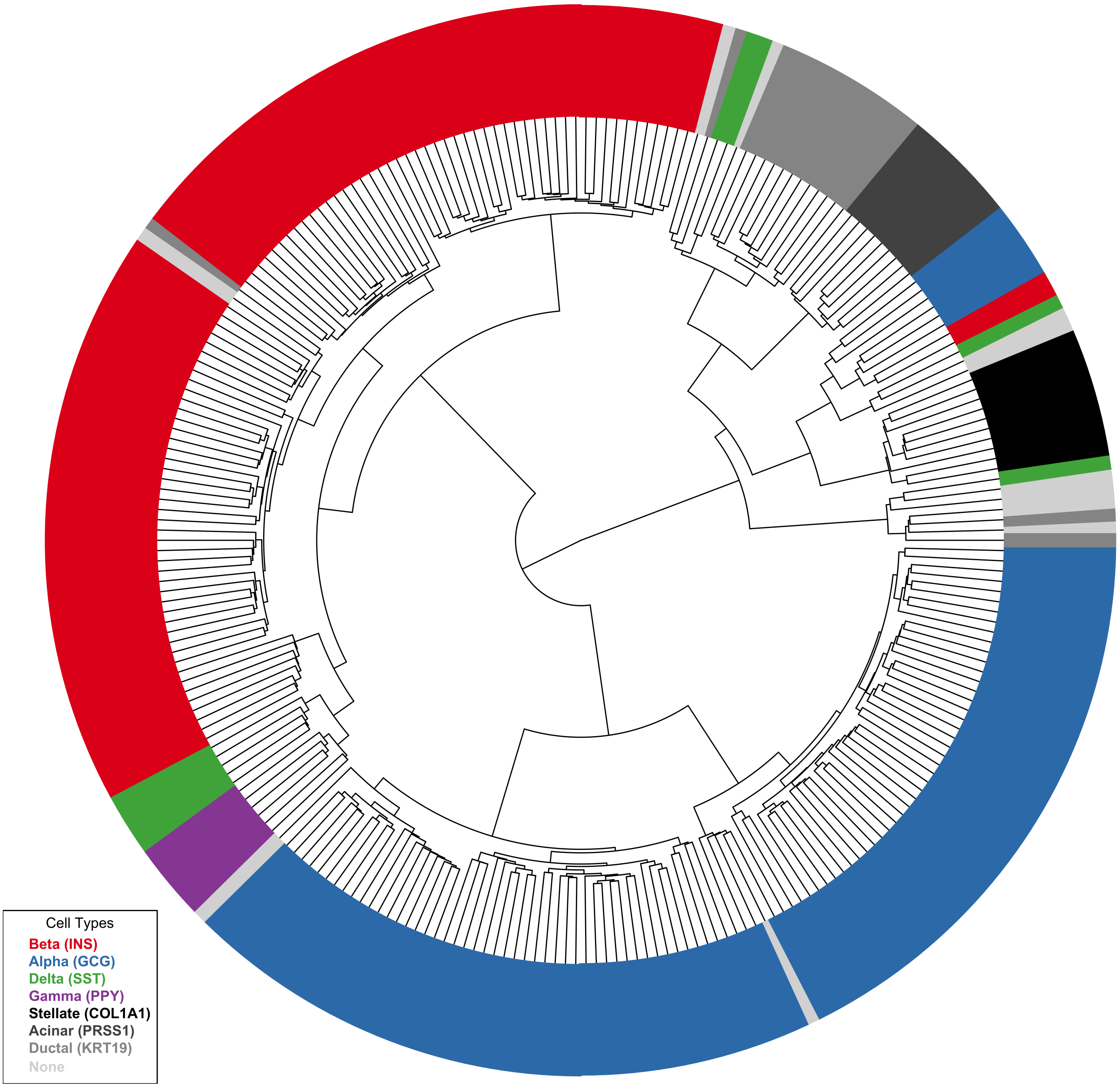
Cell Types
Beta (INS)
Alpha (GCG)
Delta (SST)
Gamma (PPY)
Stellate (COL1A1)
Acinar (PRSS1)
Ductal (KRT19)
None

Supplemental_Fig_S12: Unsupervised hierarchical clustering of Type 2 diabetic single cell transcriptomes separates samples by cell type.

Circular dendrogram of Type 2 diabetic single cell transcriptomes illustrates similar clustering by cell type as observed in non-diabetic samples. 1908 highly expressed genes with $log_2(CPM) > 10.5$ were used in the analysis. Color labels are the same as shown in Figure 3B.
