# Supplementary material for: Single-cell transcriptomes identify human islet cell signatures and reveal cell-type–specific expression changes in type 2 diabetes
Source: Genome Res. 2017 Feb;27(2):208–22. doi: 10.1101/gr.212720.116 (PMC5287227; doi:10.1101/gr.212720.116)
Supplement: Supplemental Material [file supp_gr.212720.116_Supplemental_Fig_S13.pdf]

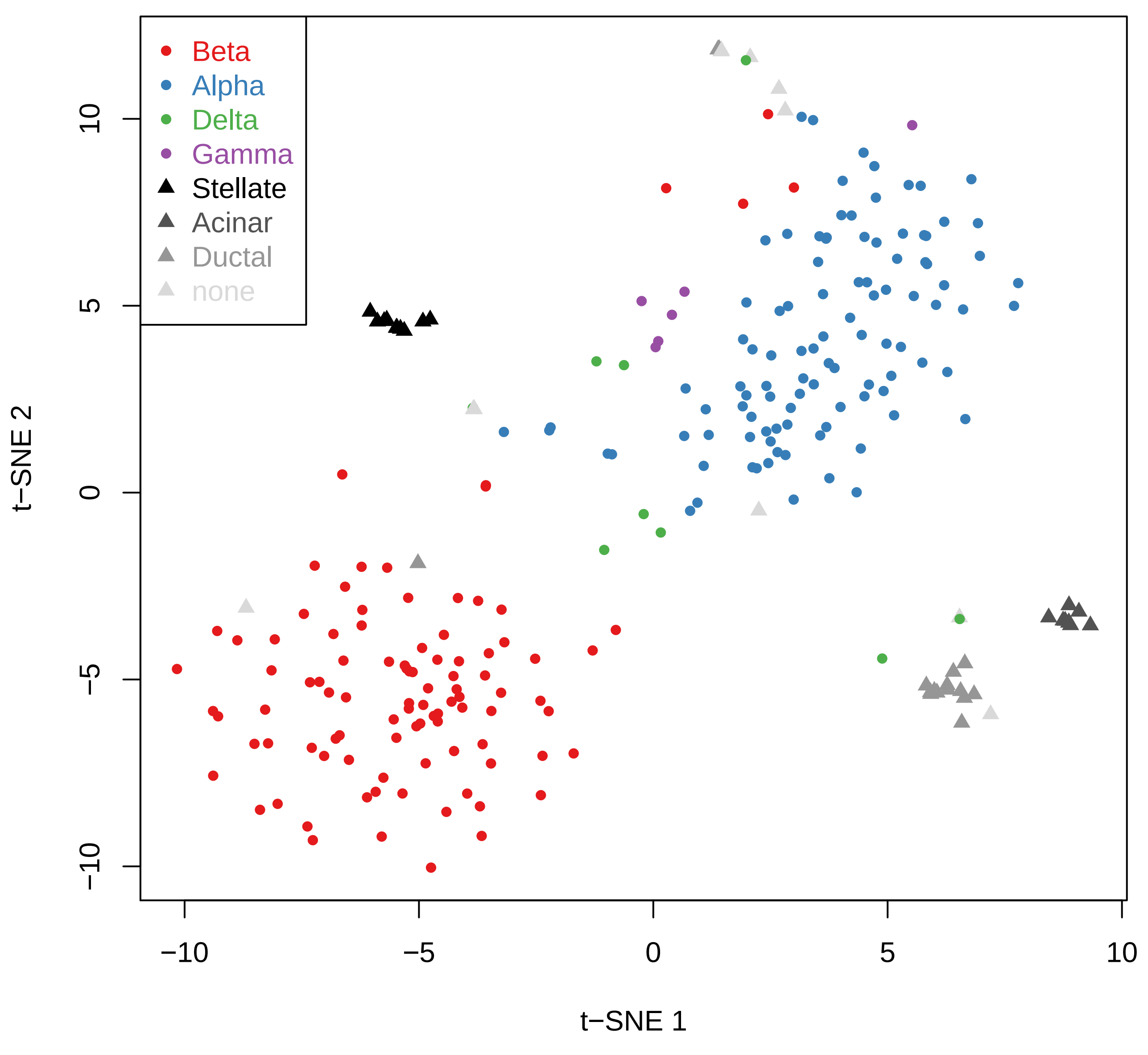

Beta
Alpha
Delta
Gamma
Stellate
Acinar
Ductal
none
t−SNE 1
t−SNE 2
−10
−5
0
5
10

Supplemental_Fig_S13: Dimension reduction of Type 2 diabetic single cell transcriptomes to two dimensions by t-SNE without marker genes still shows robust grouping of samples by cell type.

Scatter plot of Type 2 diabetic single cells after reduction to two dimensions by t-SNE without marker genes (*INS, GCG, SST, PPY, GHRL, COL1A1, PRSS1, and KRT19*). 1900 highly expressed genes with $\log_2$(CPM) > 10.5 were used in the analysis. Color labels are the same as shown in Figure 3A.
