# Supplementary material for: Single-cell transcriptomes identify human islet cell signatures and reveal cell-type–specific expression changes in type 2 diabetes
Source: Genome Res. 2017 Feb;27(2):208–22. doi: 10.1101/gr.212720.116 (PMC5287227; doi:10.1101/gr.212720.116)
Supplement: Supplemental Material [file supp_gr.212720.116_Supplemental_Fig_S15.pdf]

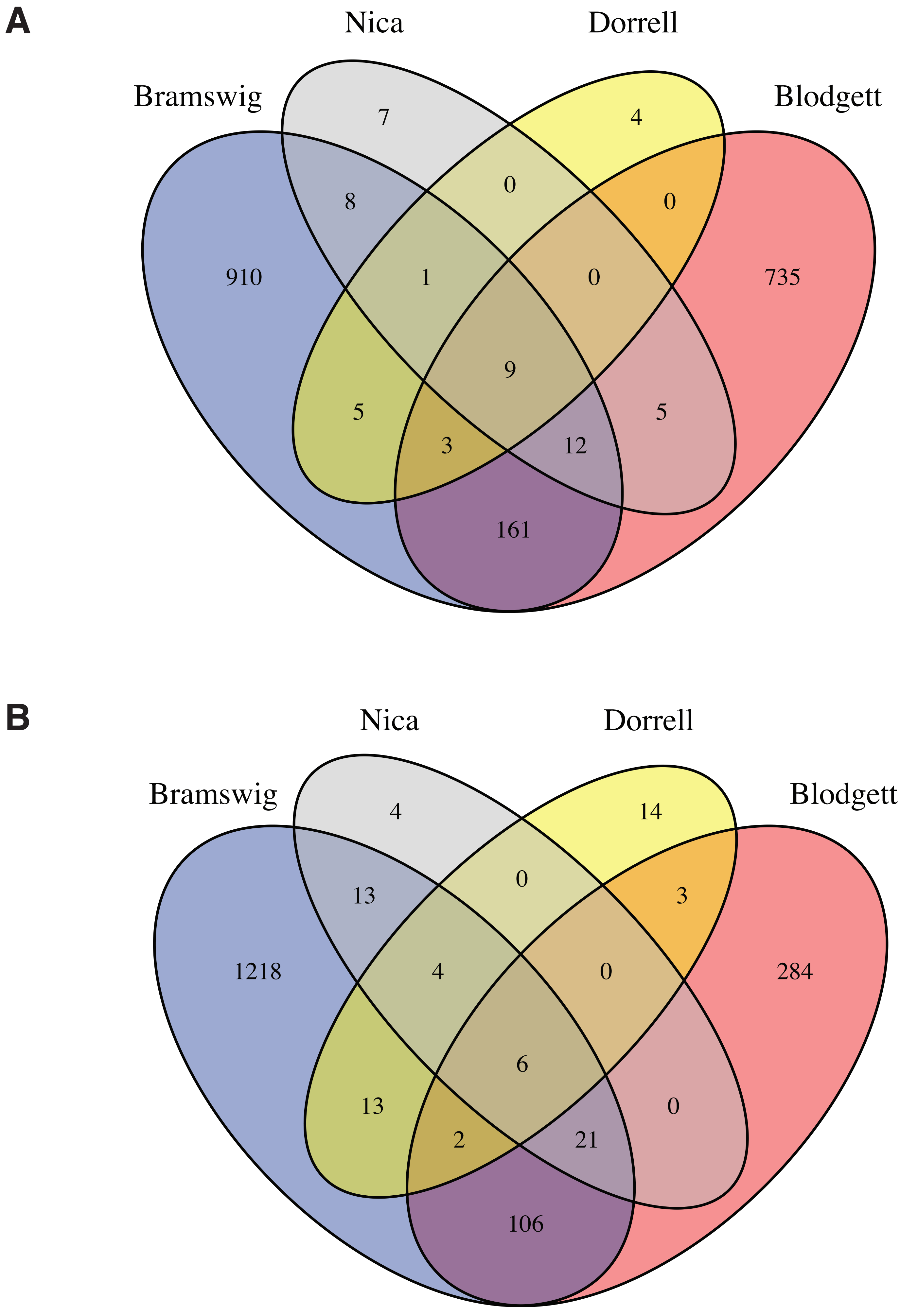
A
Nica
Dorrell
Bramswig
Blodgett
7
4
8
0
0
910
1
0
735
9
5
5
3
12
161
B
Nica
Dorrell
Bramswig
Blodgett
4
14
13
0
3
1218
4
0
284
6
13
0
2
21
106

Supplemental_Fig_S15: Intersections of lists of previously reported genes specific to islet beta or alpha cells.

Quadruple venn diagrams showing the overlap of previously reported genes specific to (A) alpha or (B) beta pancreatic islet cells from the Dorrell et al. 2011; Nica et al. 2013; Bramswig et al. 2013; and Blodgett et al. 2015 studies.
