# Supplementary material for: Single-cell transcriptomes identify human islet cell signatures and reveal cell-type–specific expression changes in type 2 diabetes
Source: Genome Res. 2017 Feb;27(2):208–22. doi: 10.1101/gr.212720.116 (PMC5287227; doi:10.1101/gr.212720.116)
Supplement: Supplemental Material [file supp_gr.212720.116_Supplemental_Fig_S16.pdf]

A

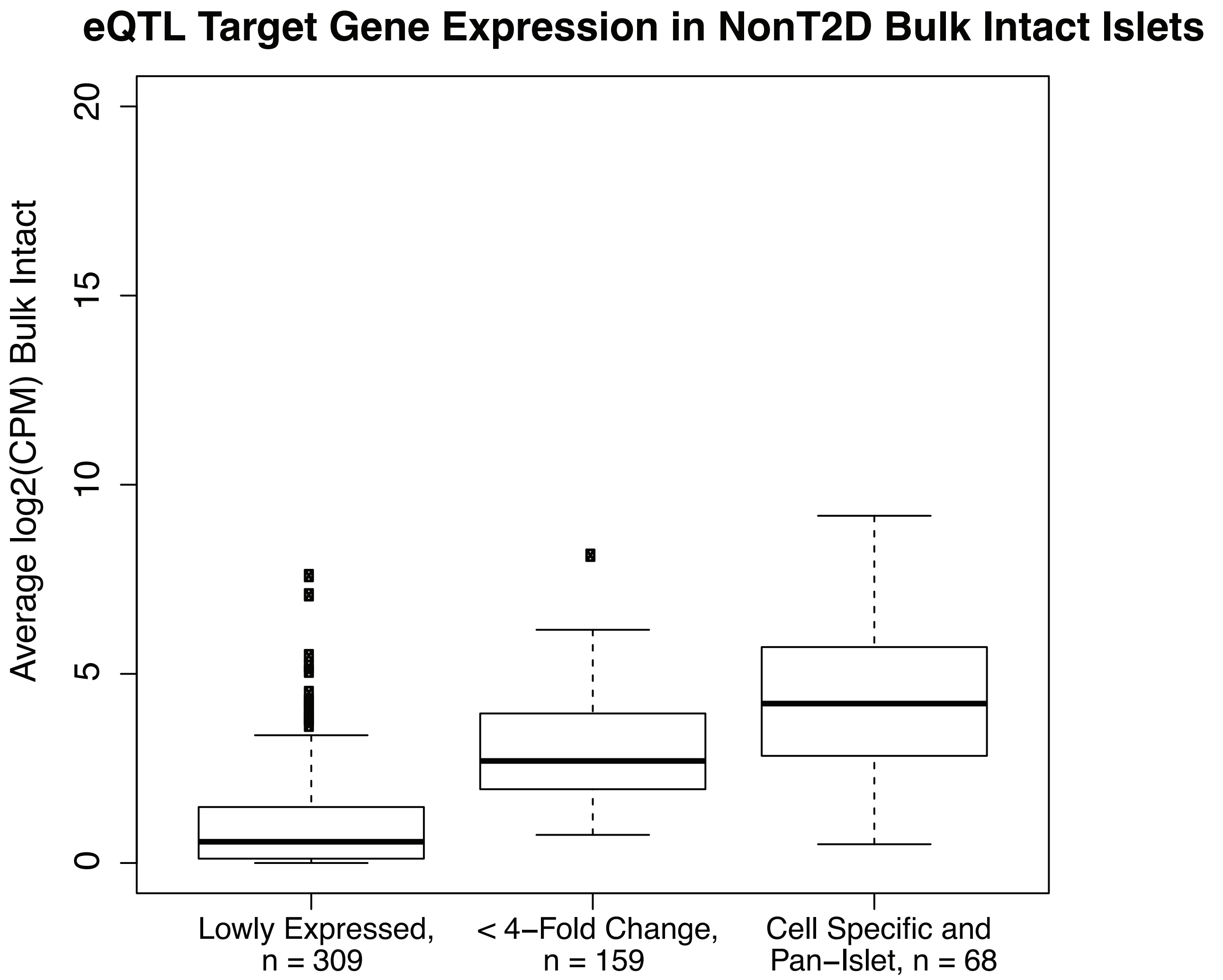
eQTL Target Gene Expression in NonT2D Bulk Intact Islets
Average log2(CPM) Bulk Intact
0
5
10
15
20
Lowly Expressed, n = 309
< 4−Fold Change, n = 159
Cell Specific and Pan−Islet, n = 68

B

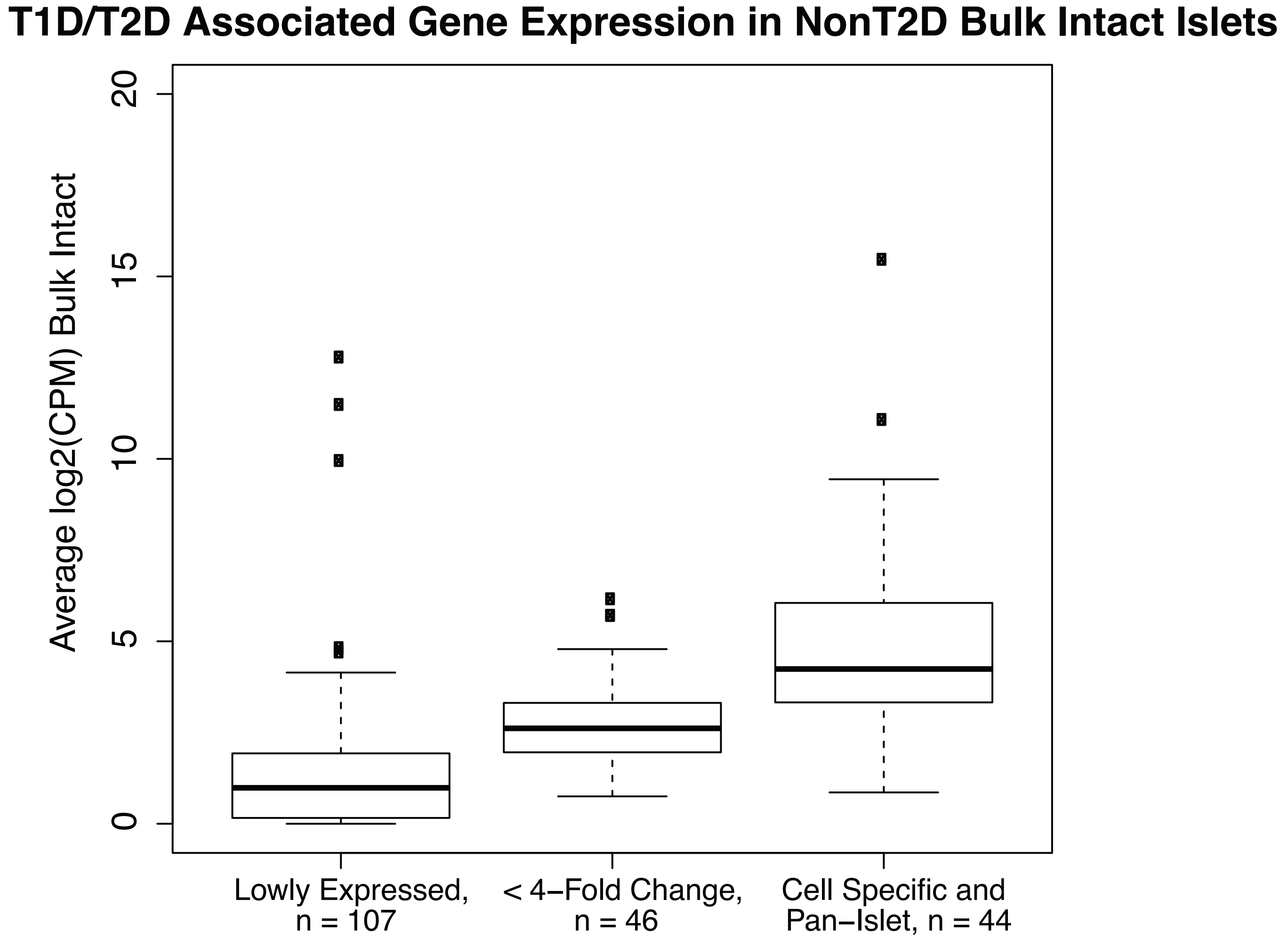
T1D/T2D Associated Gene Expression in NonT2D Bulk Intact Islets
Average log2(CPM) Bulk Intact
0
5
10
15
20
Lowly Expressed, n = 107
< 4−Fold Change, n = 46
Cell Specific and Pan−Islet, n = 44

Figure_S16: Reported Type 1 diabetes/Type 2 diabetes associated and eQTL genes that demonstrate cell specific expression patterns are more highly expressed in bulk intact islets in comparison to those that were classified as lowly expressed in our islet single cell data or not cell type-specific.

Boxplots showing the average log_2CPM expression of eQTL (A) and Type 1/Type2 diabetes-associated genes (B) lowly expressed (average log_2(CPM) < 2 in all cell types), not cell type enriched (< 4 fold change), and cell type-specific (> 4 fold change)/pan-islet (average log_2(CPM) > 4 in all islet cell types) genes in 5 non-diabetic bulk intact islets.
