# Supplementary material for: Single-cell transcriptomes identify human islet cell signatures and reveal cell-type–specific expression changes in type 2 diabetes
Source: Genome Res. 2017 Feb;27(2):208–22. doi: 10.1101/gr.212720.116 (PMC5287227; doi:10.1101/gr.212720.116)
Supplement: Supplemental Material [file supp_gr.212720.116_Supplemental_Fig_S17.pdf]

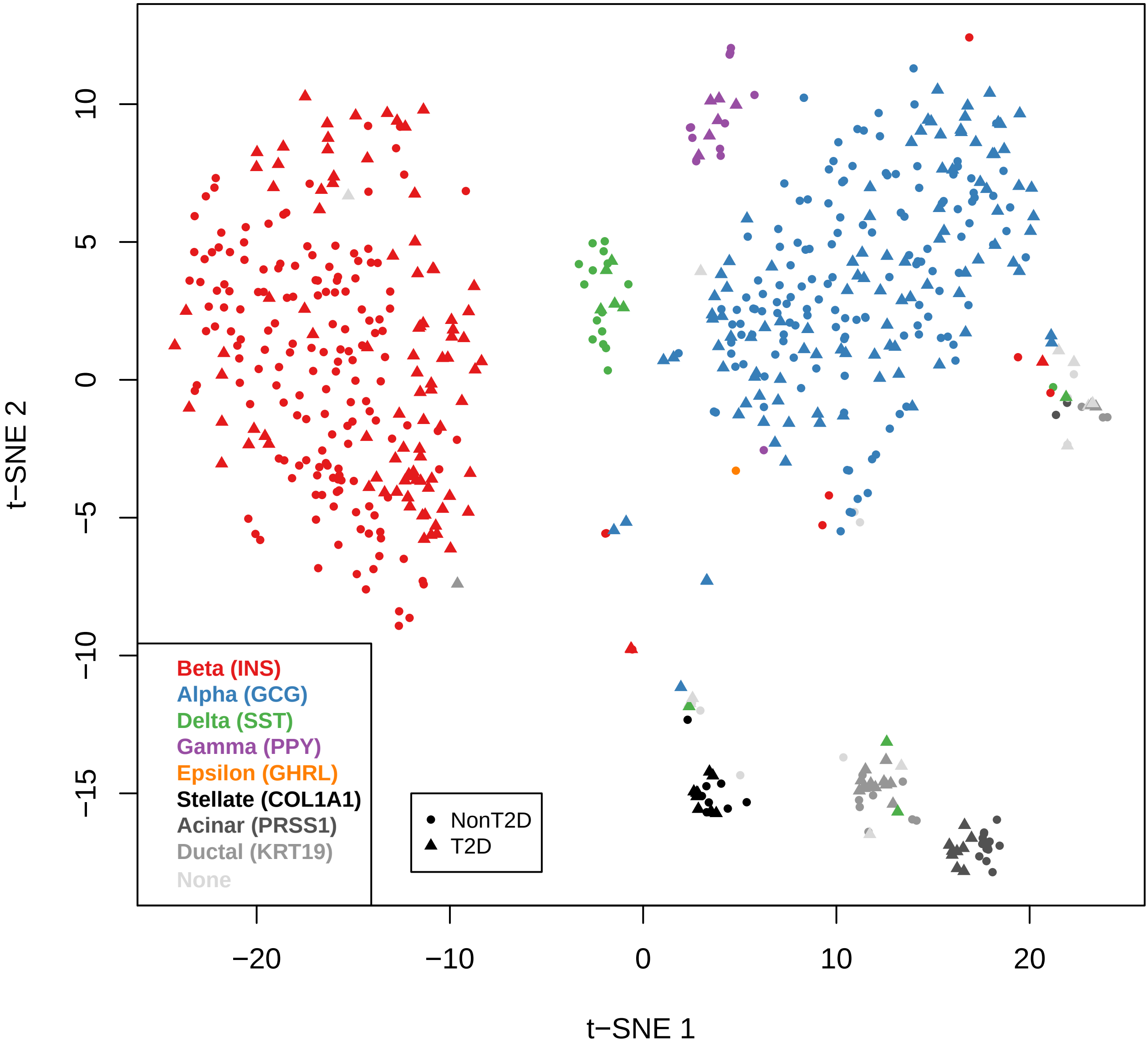

t−SNE 1
t−SNE 2
−20
−10
0
10
20
−15
−10
−5
0
5
10
Beta (INS)
Alpha (GCG)
Delta (SST)
Gamma (PPY)
Epsilon (GHRL)
Stellate (COL1A1)
Acinar (PRSS1)
Ductal (KRT19)
None
NonT2D
T2D

Supplemental_Fig_S17: Reduction of non-diabetic and Type 2 diabetic single cell transcriptomes to two dimensions via unsupervised t-SNE shows robust grouping by cell types regardless of disease state.

Scatter plot of non-diabetic and Type 2 diabetic single cell samples after t-SNE dimensionality reduction. Similar to the hierarchical clustering analysis, 2754 highly expressed genes with $\log_2(CPM) > 10.5$ were used. Cell type identities, coloring, and disease state information were labeled after the t-SNE analysis. Circular points correspond to non-diabetic single cells while triangular points correspond to Type 2 diabetic single cells.
