# Supplementary material for: Single-cell transcriptomes identify human islet cell signatures and reveal cell-type–specific expression changes in type 2 diabetes
Source: Genome Res. 2017 Feb;27(2):208–22. doi: 10.1101/gr.212720.116 (PMC5287227; doi:10.1101/gr.212720.116)
Supplement: Supplemental Material [file supp_gr.212720.116_Supplemental_Fig_S19.pdf]

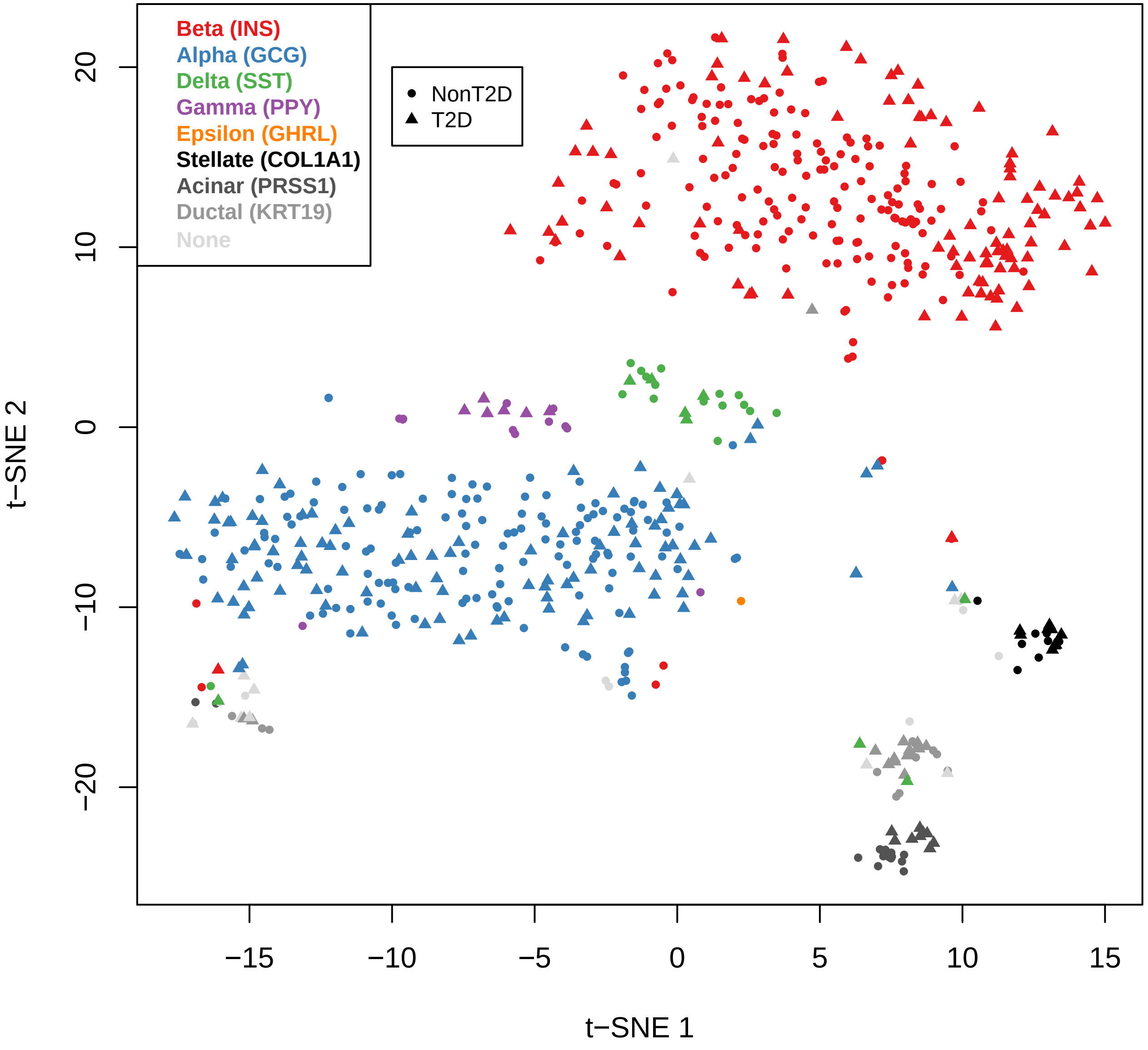

Beta (INS)
Alpha (GCG)
Delta (SST)
Gamma (PPY)
Epsilon (GHRL)
Stellate (COL1A1)
Acinar (PRSS1)
Ductal (KRT19)
None
NonT2D
T2D
20
10
0
−10
−20
−15
−10
−5
0
5
10
15
t−SNE 1
t−SNE 2

Supplemental_Fig_S19: Dimensionality reduction of non-diabetic and Type 2 diabetic single cell transcriptomes to two dimensions via unsupervised t-SNE after removing marker genes maintains clear separation of transcriptomes by cell types.

2-D Scatter plot of non-diabetic and Type 2 diabetic single cells after reduction to two dimensions by t-SNE without marker genes. 2746 highly expressed genes with $\log_2(CPM) > 10.5$ were used. Cell type identities, coloring, and disease state information were labeled after the t-SNE analysis. Circular points correspond to non-diabetic single cells while triangular points correspond to Type 2 diabetic single cells.
