# Supplementary material for: Single-cell transcriptomes identify human islet cell signatures and reveal cell-type–specific expression changes in type 2 diabetes
Source: Genome Res. 2017 Feb;27(2):208–22. doi: 10.1101/gr.212720.116 (PMC5287227; doi:10.1101/gr.212720.116)
Supplement: Supplemental Material [file supp_gr.212720.116_Supplemental_Fig_S20.pdf]

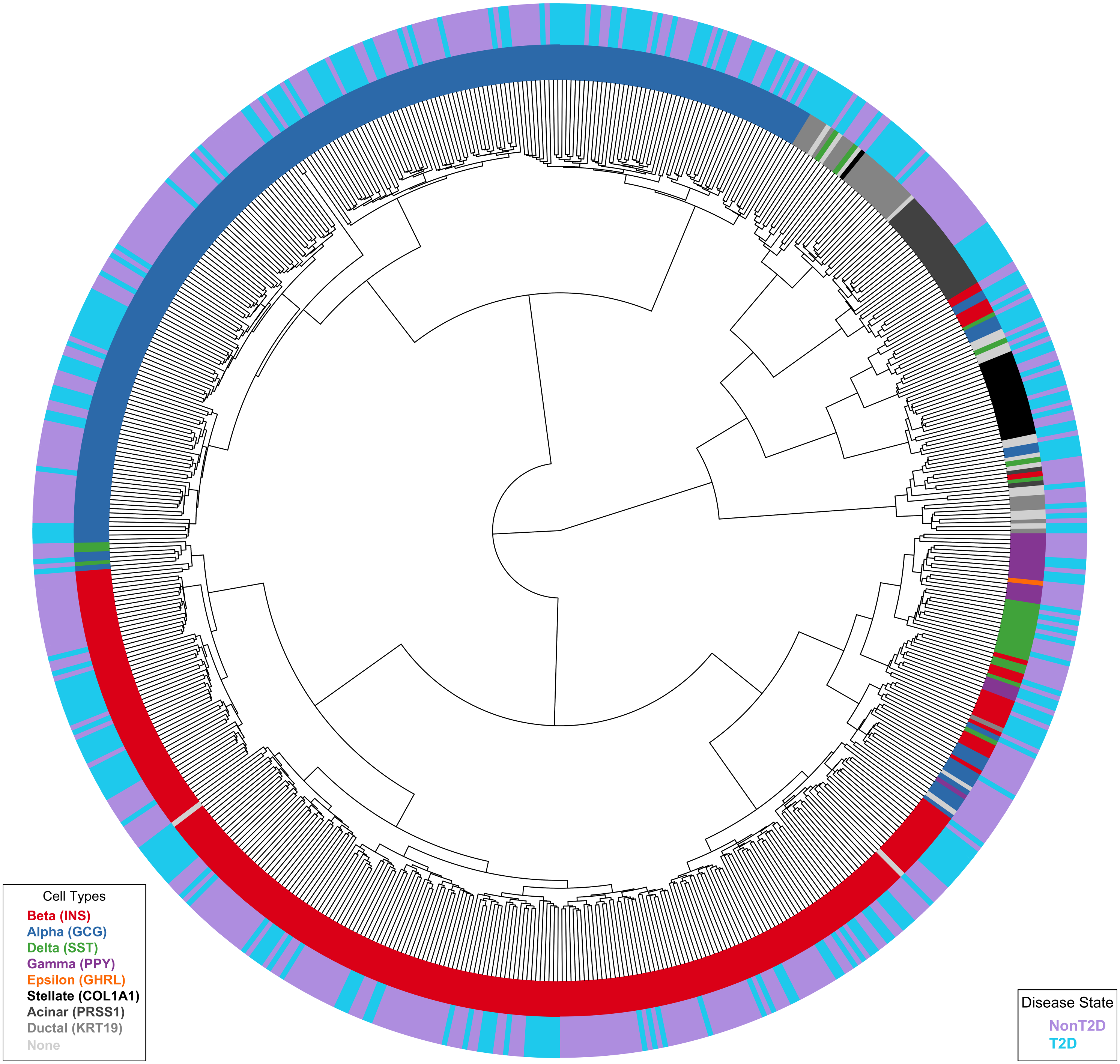

Cell Types
Beta (INS)
Alpha (GCG)
Delta (SST)
Gamma (PPY)
Epsilon (GHRL)
Stellate (COL1A1)
Acinar (PRSS1)
Ductal (KRT19)
None
Disease State
NonT2D
T2D

Supplemental_Fig_S20: Unsupervised hierarchical clustering of non-diabetic and Type 2 diabetic single cell transcriptomes after removing marker genes discretize samples by cell type and not disease state.

Circular dendrogram of single cell transcriptomes with cell type and disease status information overlain. The dendrogram was generated after performing unsupervised hierarchical clustering of both non-diabetic and Type 2 diabetic single cells without marker genes (*INS, GCG, SST, PPY, GHRL, COL1A1, PRSS1, and KRT19*) and 2746 highly expressed genes with $\log_2(CPM) > 10.5$. Appropriate labels and coloring of samples were added after the unsupervised analysis.
