# Supplementary material for: Single-cell transcriptomes identify human islet cell signatures and reveal cell-type–specific expression changes in type 2 diabetes
Source: Genome Res. 2017 Feb;27(2):208–22. doi: 10.1101/gr.212720.116 (PMC5287227; doi:10.1101/gr.212720.116)
Supplement: Supplemental Material [file supp_gr.212720.116_Supplemental_Fig_S21.pdf]

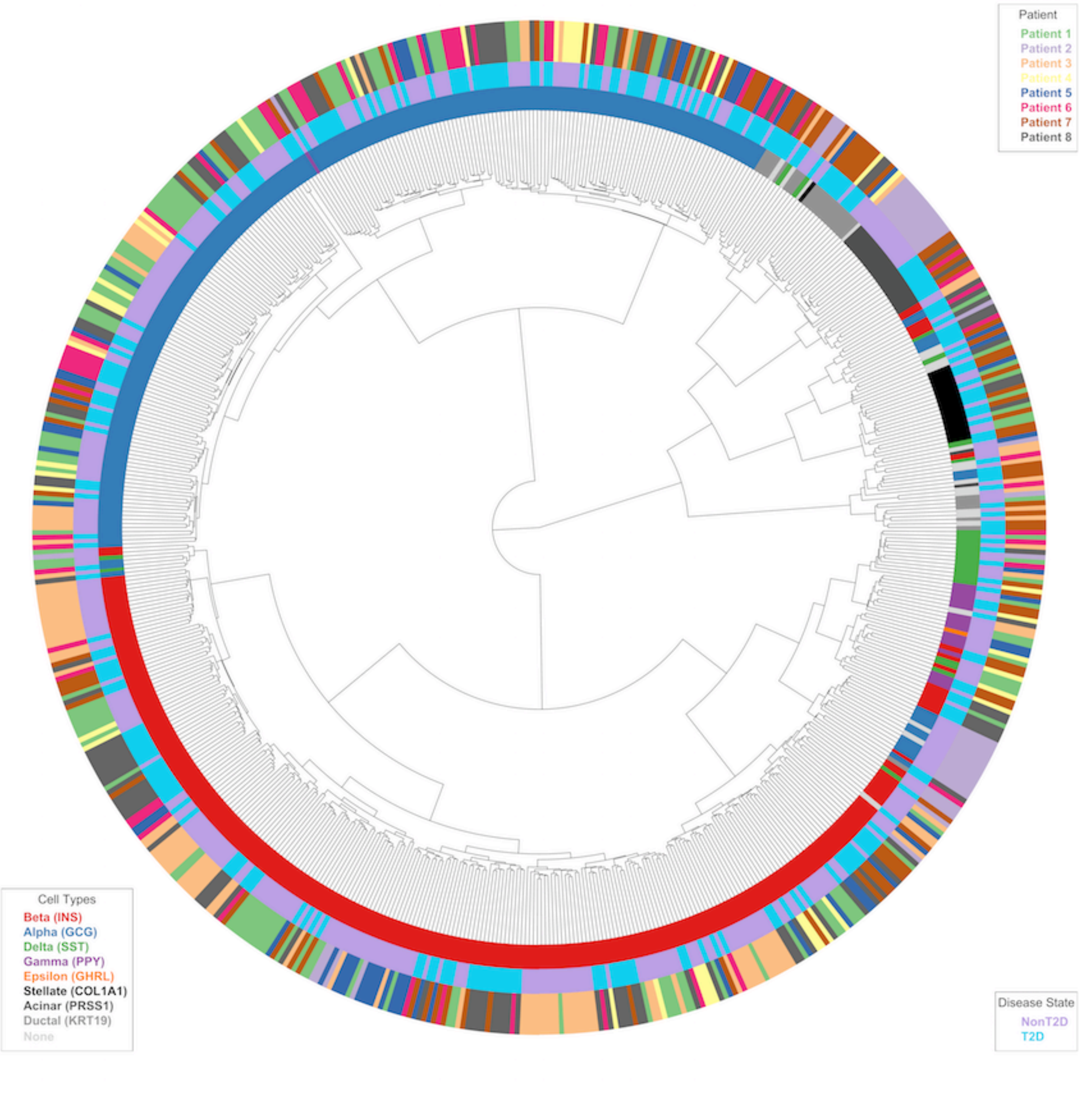
Patient
Patient 1
Patient 2
Patient 3
Patient 4
Patient 5
Patient 6
Patient 7
Patient 8
Cell Types
Beta (INS)
Alpha (GCG)
Delta (SST)
Gamma (PPY)
Epsilon (GHRL)
Stellate (COL1A1)
Acinar (PRSS1)
Ductal (KRT19)
None
Disease State
NonT2D
T2D

Supplemental_Fig_S21: Unsupervised hierarchical clustering of combined non-diabetic and Type 2 diabetic single cell samples reveals grouping of transcriptomes by cell type regardless of disease state and patient.

Circular dendrogram of single cell transcriptomes with cell type, disease status, and patient information provided. The dendrogram was generated after performing hierarchical clustering of both non-diabetic and Type 2 diabetic single cells using 2754 highly expressed genes with $\log_2(CPM) > 10.5$. Appropriate labels and coloring of samples were added after the unsupervised analysis.
