# Supplementary material for: Single-cell transcriptomes identify human islet cell signatures and reveal cell-type–specific expression changes in type 2 diabetes
Source: Genome Res. 2017 Feb;27(2):208–22. doi: 10.1101/gr.212720.116 (PMC5287227; doi:10.1101/gr.212720.116)
Supplement: Supplemental Material [file supp_gr.212720.116_Supplemental_Fig_S22.pdf]

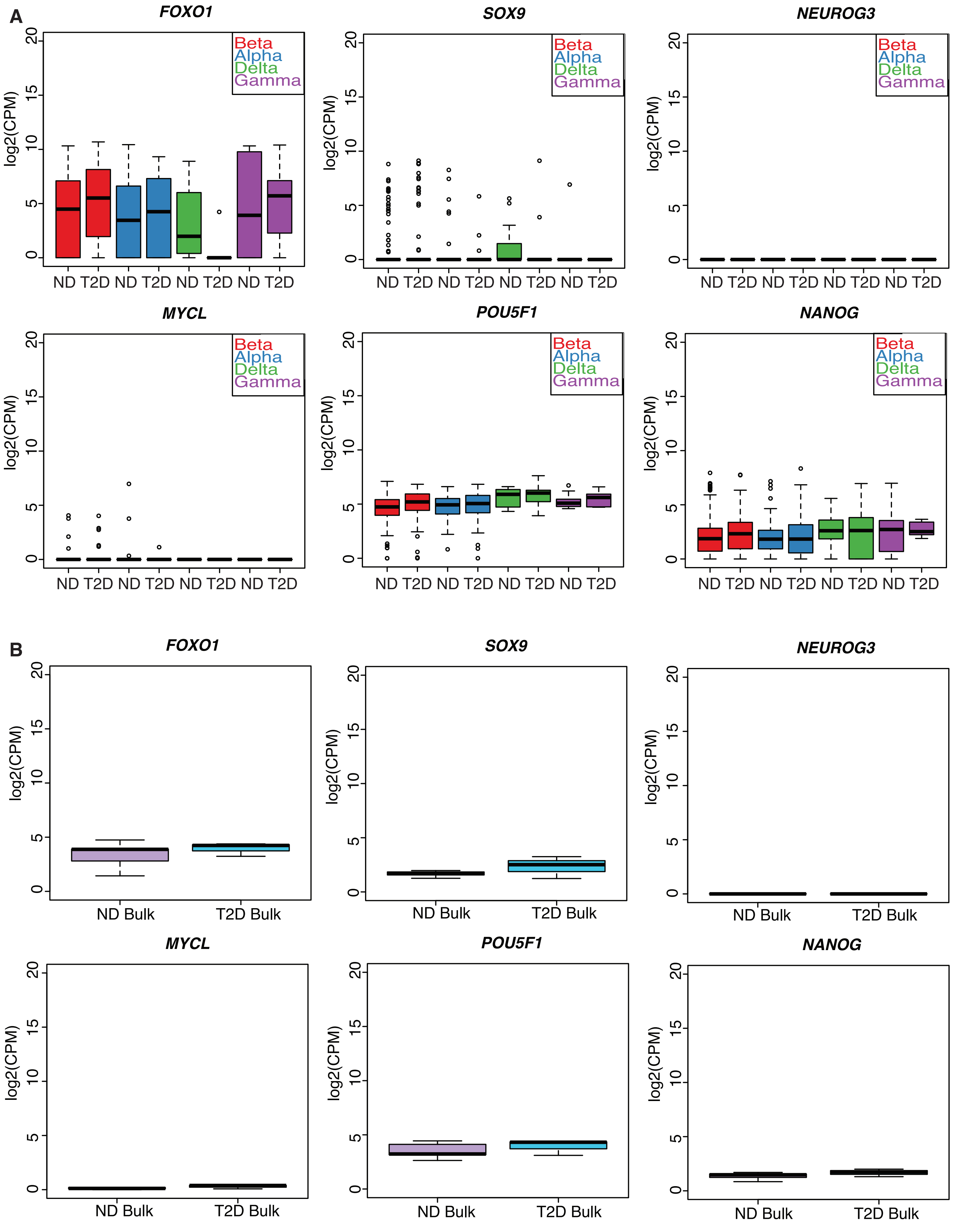
A
FOXO1
SOX9
NEUROG3
MYCL
POU5F1
NANOG
Beta
Alpha
Delta
Gamma
log2(CPM)
ND T2D ND T2D ND T2D ND T2D
B
FOXO1
SOX9
NEUROG3
MYCL
POU5F1
NANOG
log2(CPM)
ND Bulk
T2D Bulk

Supplemental_Fig_S22: Islet de-differentiation genes were not differentially expressed in single cell nor paired bulk intact islet transcriptomes.

(A) Box plots of log_2CPM expression of islet de-differentiation markers as specified in Talchai et al. 2012 in islet single cell type transcriptomes. No genes showed significant differences (p-value < 0.05) in expression between diabetic and non-diabetic cell types after one-way ANOVA. (B) Box plots of log_2CPM expression of islet de-differentiation markers in bulk intact islet transcriptomes. No genes showed significant differences (p-value < 0.05) in expression between diabetic and non-diabetic islets after one-way ANOVA. ND = Non-diabetic, T2D = type 2 diabetic, CPM = Counts per million.
