# Supplementary material for: Single-cell transcriptomes identify human islet cell signatures and reveal cell-type–specific expression changes in type 2 diabetes
Source: Genome Res. 2017 Feb;27(2):208–22. doi: 10.1101/gr.212720.116 (PMC5287227; doi:10.1101/gr.212720.116)
Supplement: Supplemental Material [file supp_gr.212720.116_Supplemental_Fig_S23.pdf]

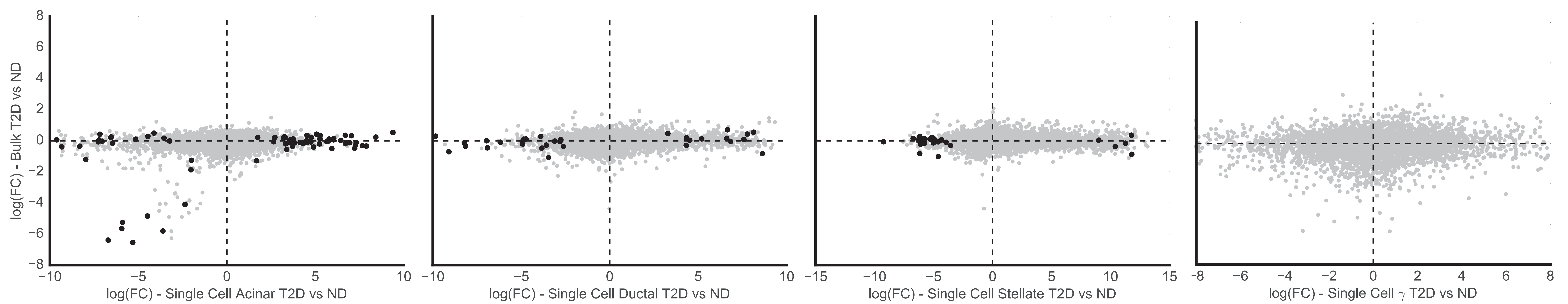
log(FC) - Bulk T2D vs ND
log(FC) - Single Cell Acinar T2D vs ND
log(FC) - Single Cell Ductal T2D vs ND
log(FC) - Single Cell Stellate T2D vs ND
log(FC) - Single Cell γ T2D vs ND

Supplemental_Fig_S23: Differential expression analysis at single cell resolution identifies expression changes in Type 2 diabetic and non-diabetic exocrine cell types.

Additional scatter plots depicting the log_2 fold change of gene expression observed in Type 2 diabetic versus non-diabetic bulk intact islet transcriptomes on the y-axis and the respective log_2 fold change values of gene expression observed in those of Type 2 diabetic versus non-diabetic single cells. Points highlighted in black represent genes that were detected to be significantly differentially expressed (FDR <0.05) in acinar, stellate, or ductal exocrine cells at single cell resolution. In all plots, points highlighted in grey were not significantly differentially expressed (FDR > 0.05). No significant changes in gene expression were found between the Type 2 diabetic and non-diabetic PP/gamma cell transcriptomes. γ = PP/gamma, FC = fold change, T2D = Type 2 diabetic, ND = non-diabetic.
