# Supplementary material for: Single-cell transcriptomes identify human islet cell signatures and reveal cell-type–specific expression changes in type 2 diabetes
Source: Genome Res. 2017 Feb;27(2):208–22. doi: 10.1101/gr.212720.116 (PMC5287227; doi:10.1101/gr.212720.116)
Supplement: Supplemental Material [file supp_gr.212720.116_Supplemental_Fig_S24.pdf]

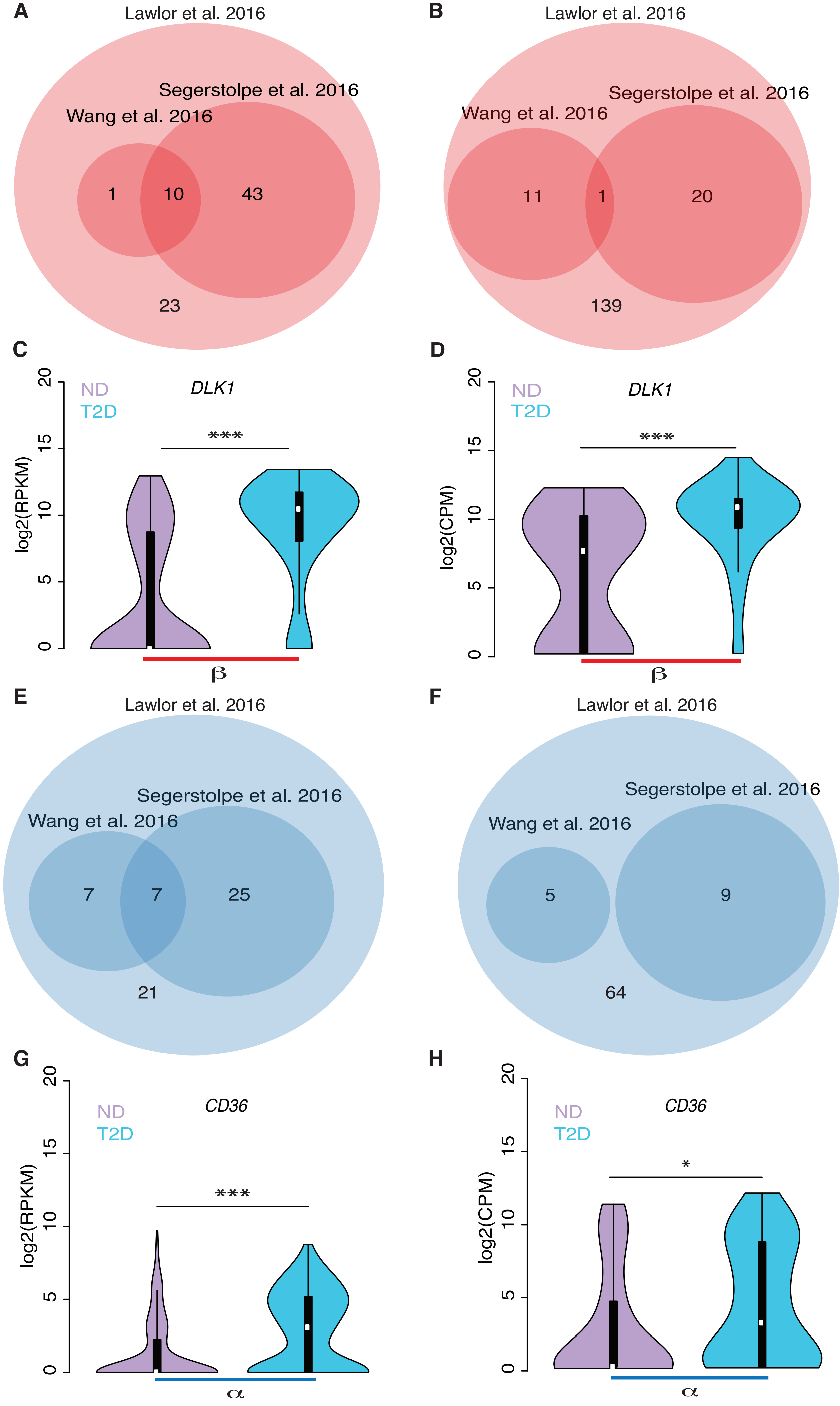
A
Lawlor et al. 2016
Segerstolpe et al. 2016
Wang et al. 2016
1
10
43
23
B
Lawlor et al. 2016
Segerstolpe et al. 2016
Wang et al. 2016
11
1
20
139
C
ND
T2D
DLK1
***
log2(RPKM)
β
D
ND
T2D
DLK1
***
log2(CPM)
β
E
Lawlor et al. 2016
Segerstolpe et al. 2016
Wang et al. 2016
7
7
25
21
F
Lawlor et al. 2016
Segerstolpe et al. 2016
Wang et al. 2016
5
9
64
G
ND
T2D
CD36
***
log2(RPKM)
α
H
ND
T2D
CD36
*
log2(CPM)
α

Supplemental_Fig_S24: Validation of genes differentially expressed in diabetic and non-diabetic cell types in independent human islet single cell transcriptomic studies.

For each cell type, we identified lists of genes that exhibited differential expression between non-diabetic and diabetic states. After two-sided Wilcoxon rank sum test of these cell type specific differentially expressed genes in the Wang et al. 2016 and Segerstolpe et al. 2016 datasets, we validated that 54/77 genes were significantly upregulated (p-value < 0.05) in diabetic beta cells from our dataset and either of the other two datasets (A). (B) 32/171 genes were significantly down-regulated in our data and the respective datasets. *DLK1* expression in diabetic and non-diabetic beta cells from (C) Segerstolpe et al. 2016 and (D) Wang et al. 2016 respectively. (E) 39/60 genes were enriched in diabetic alpha cells from our dataset and either of the two datasets. (F) 14/78 genes were verified as downregulated in diabetic alpha cells in our data and the two respective datasets. *CD36* expression in diabetic and non-diabetic alpha cells from (G) Segerstolpe et al. 2016 and (H) Wang et al. 2016 respectively. ND = non-diabetic, T2D = type 2 diabetic, RPKM = reads per kilobase of transcript per million mapped reads, CPM = counts per million, * = p-value < 0.05, *** = p-value < 0.001, β = beta cell, α = alpha cell.
